# Interventions to Enhance COVID-19 Pandemic Health Literacy in Health Professionals: Systematic Review

**DOI:** 10.2196/70400

**Published:** 2026-07-10

**Authors:** Claudia Hasenpusch, Paula Kuper, Eva-Maria Skiba, Antonia Anabella Sprenger, Uwe Matterne, Lena Kannengießer, Esther Ivanka Grätsch, Jaekyung Shin, Mengtong Li, Christian Joachim Apfelbacher

**Affiliations:** 1 Institute of Social Medicine and Health Systems Research, Medical Faculty, Otto von Guericke University Magdeburg, Leipziger Straße 44, Magdeburg, 39120, Germany, 49 3916724302

**Keywords:** pandemics, SARS-CoV-2, infection control, comparative effectiveness research, health literacy, health education

## Abstract

**Background:**

The COVID-19 pandemic has placed a significant burden on health professionals (HPs). They face higher infection risks due to the nature of their work environment and patient care responsibilities. Their ability to access and apply reliable COVID-19 information affects their own preventive behavior and that of those around them. In this context, health literacy (HL) has become increasingly important. Despite extensive research, information to foster COVID-19–related HL in HPs remains limited.

**Objective:**

This systematic review aimed to identify, appraise, and synthesize intervention studies on the effectiveness of COVID-19–related HL interventions in HPs.

**Methods:**

Five electronic databases (eg, PubMed (MEDLINE), Embase), six clinical trials registries (eg, ISRCTN registry), one preprint server (MEDRXIV), published conference proceedings, and five gray literature databases (eg, opengrey.eu, ProQuest) were searched in May 2022 and updated in August 2025. Reference lists of included studies were screened manually. Two reviewers independently screened titles, abstracts, and full-texts according to eligibility criteria and extracted data; disagreements were resolved by discussion or consultation with a third reviewer. We included randomized controlled trials (RCTs), nonrandomized studies of interventions, and uncontrolled before-and-after studies evaluating the effectiveness of any COVID-19–related HL intervention. Primary outcomes include COVID-19–related HL, its four facets (access, understand, appraise, and apply COVID-19 information), and indicators (eg, COVID-19–related knowledge), assessed at postintervention and follow-up. When studies were sufficiently similar, random-effects meta-analyses were performed; otherwise, a narrative synthesis was provided. Risk of bias was assessed using validated tools based on study design, and the overall certainty of the evidence was evaluated by the GRADE (Grading of Recommendations, Assessment, Development, and Evaluation) approach.

**Results:**

We included 15 RCTs (2034 participants), 4 nonrandomized studies of interventions (291 participants), 74 uncontrolled before-and-after studies (327,298 participants), 5 ongoing studies, and 1 study with awaiting classification. Interventions targeted a broad range of health occupational groups. Intervention type, delivery mode, methods, settings, and comparator varied widely. No outcome measure explicitly referred to an HL model. Most studies aimed to enhance COVID-19–related knowledge and skills, and had a high risk of bias. COVID-19-related interventions may increase knowledge of vaccines (standardized mean difference 1.00; 95% CI 0.33 to 1.67, *I*^2^=24%), and the infection prevention control skills, such as donning and doffing of personal protective equipment (standardized mean difference 1.95; 95% CI 1.82 to 3.09, *I*^2^=46%), but the evidence remains very uncertain.

**Conclusions:**

COVID-19–related HL interventions may promote HP’s short-term competencies in infection control. However, the evidence remains uncertain, primarily due to the low quality of studies, characterized by a high risk of bias. Interventions specifically designed to enhance the full COVID-19 HL operationalized by its four facets are lacking. High-quality RCTs with sufficient statistical power, grounded in HL theoretical principles, are needed to achieve precise understanding.

## Introduction

The occurrence of novel infectious diseases, such as COVID-19, requires a rapid implementation of prevention measures to contain the spread of the virus. Infection control measures to protect oneself and others are linked to behavior changes of individuals [1]. In the face of constantly changing recommendations and infection control measures in the early phase of the pandemic, individuals needed to recognize recommended measures such as social distancing, wearing a face mask, washing and disinfecting their hands regularly, testing for SARS-CoV-2, or getting vaccinated, and had to be capable of carrying them out [2-4]. For example, in March 2020, using a face mask was not considered an effective preventive measure for the prevention of potential transmission of SARS-CoV-2, while in June 2020, the World Health Organization (WHO) had modified their recommendation based on new evidence of its effectiveness [5]. Attainment of habituation of preventative behavior is important to consider as well if one wishes the behavior to be sustained [1].

The pandemic has not only posed unprecedented challenges for the general population, but health professionals (HPs) in particular faced a high burden, including occupational exposure, increased workload demands, rapidly changing infection-prevention requirements in patient care, and considerable psychological strain [6-8]. HPs, including physicians, nurses, midwives, physiotherapists, and other HPs [9], have a heightened risk of infection, especially during patient care and disposal of biomedical infectious waste. The risk for infection in this occupational population is 35% higher than in professions without infection-relevant working conditions [10], given the direct exposure to individuals who may be asymptomatic carriers of infectious diseases such as COVID-19. Besides their daily demands, HPs need to be cautious to prevent the spread of infections to noninfected patients, colleagues, and even their own social environment, simultaneously [7,11,12]. This requires real-time adjustments, that is, adapting to changes in governmental policies, recommendations, and adhering to guidance and clinical protocols immediately in order to ensure that care is provided adequately and safely. In addition, HPs deal with ethical responsibilities and moral obligations [7,13,14] when deciding to spend time in environments where the risk of infection is heightened. This dilemma, the responsibility to provide high-quality care to patients while simultaneously safeguarding themselves from potential harm, was intensified during the pandemic, when the demand for health care services was overwhelming [7]. Infection disease prevention competencies, such as proper hand hygiene, surface disinfection, use of personal protective equipment (PPE), biomedical waste management, and infection prevention and control practice training, are crucial in mitigating the infection risks for HPs and those around them [5,6,15].

Implementing personal protective measures in one’s everyday life depends on the ability to access, understand, appraise, and apply pandemic-relevant recommendations and information [16,17]. This ability can be defined as COVID-19–related health literacy (HL) [16,18,19]. In general, HL refers to the motivation, knowledge, and competencies of individuals (access, understand, appraise, and apply health information) to make decisions related to their health [20]. Various factors influence the health-related competencies of individuals, for instance, situational (eg, media use, social support, family, and peer influences) and personal determinants (eg, age, gender, and socioeconomic status) [20]. As the onset of the COVID-19 pandemic, and followed by its transition into an endemic, HL in the context of communicable diseases, particularly of COVID-19, has gained substantial importance [16,21,22]. It has been suggested that adequate HL may not be as prevalent among populations as needed to successfully navigate the increasing amount of both reliable information and misinformation during the onset of the pandemic, referred to as “infodemic” [21,23-26]. The *Health Literacy Survey COVID-19* found that during the first wave of the COVID-19 outbreak in Germany (spring 2020), 50.4% (n=1037) of the study population had “insufficient” COVID-19–related HL [17,22]. These findings are comparable to the results of previous studies on general HL [22,27]. However, the proportion of participants with low HL decreased by 14.9% at the second time of measurement (December 2020) [22]. Okan et al [22] assumed this can be explained by the fact that, since the beginning of the COVID-19 pandemic, information about SARS-CoV-2 and COVID-19 has been provided continuously and extensively on different information channels. Further, synthesized evidence has shown an association between HL and (nonpandemic) infectious disease prevention behavior [28]. This association may be even more relevant during a pandemic.

HL may be improved by providing valid and comprehensible information, effective communication strategies, and structured education [29]. Interventions are either tailored to the specific needs of at-risk individuals (eg, patients or predisposed individuals) or the general population [29]. HL is a crucial intervention target, as it may counteract the widespread dis- and misinformation during a pandemic [17,22,30].

There is evidence that HL can be improved by interventions. A systematic review of school-based educational interventions found higher competence in the critical appraisal of health claims shortly after intervention (eg, knowledge and skills) [31]. Another systematic review concluded that group and individual interventions in both primary health care and community settings may support sustained change in HL [32]. Further systematic reviews reported that interventions aiming to improve HPs’ mental HL [33] and eHealth literacy [34] were effective.

However, interventions aiming to improve HL should be conceptually distinguished from broader health education interventions, infection prevention, or behavioral skills training. While health education programs often focus on providing knowledge, teaching procedural skills, or promoting specific behaviors, HL interventions more specifically aim to strengthen individuals’ competencies to access, understand, appraise, and apply health information in order to make informed health decisions [35]. Although health education strategies are often used to foster HL competencies, these approaches are not conceptually synonymous and should therefore be differentiated when defining and interpreting intervention studies.

While a considerable amount of research has been undertaken on HL during the COVID-19 pandemic [17,22,35-37], evidence on interventions specifically aiming to enhance COVID-19–related HL among HPs remains limited [7,36]. Therefore, the purpose of this systematic review was to identify, appraise, and synthesize studies examining the effectiveness of interventions aiming to improve COVID-19–related HL, its dimensions (access, understand, appraise, and apply), or related competency indicators among HPs.

## Methods

### Study Design

The systematic review was reported according to the PRISMA (Preferred Reporting Items for Systematic Reviews and Meta-Analyses) 2020 Statement and Checklist (Checklist 1) and abstract checklist (Checklist 2) [38]. It was registered at PROSPERO (CRD42022301810).

### Ethics Approval and Consent to Participate

This study is a systematic review of the literature. Ethical approval was not necessary.

### Search Strategy

We searched in five electronic databases (PubMed [MEDLINE], Embase, Web of Science [Science Citation Index & Social Sciences Citation Index], PsycInfo, and Epistemonikos Database). To identify ongoing studies, we also searched five clinical trial registries (Cochrane Central Register of Controlled Trials, Cochrane COVID-19 Study Register, ISRCTN registry, Australian New Zealand Clinical Trials Registry, and EU Clinical Trials Register). Additionally, we screened preprint servers (medRxiv), published conference proceedings, and gray literature (Base, opengrey.eu, Social Science Open Access, Centers for Disease Control and Prevention WONDER, and ProQuest). We manually searched the reference lists of included studies and contacted corresponding authors to obtain unpublished study reports. The initial search was performed in May 2022, followed by a first and second update search in December 2023 and August 2025. Only studies published after the onset of COVID-19 (December 2019) were considered; a filter was applied accordingly. The search strategy was not restricted in terms of the population of interest or language. The review question was operationalized based on the PICO (Population-Intervention-Comparator-Outcome) framework, which was extended by the study design [39]. The search strategies are presented in detail in the additional file Multimedia Appendix 1 [40-42].

### Eligibility Criteria

#### Population

According to the international classification of health workers provided by the WHO (2019) [9], we included HPs, representing an occupational population, who (1) provide preventive, curative, rehabilitative, and promotional health services based on extensive theoretical and practical knowledge in diagnosing and treating diseases (eg, doctors and nurses), (2) perform technical and practical tasks and assist (eg, medical and pathology laboratory technicians, pharmaceutical technicians, community health workers, and ambulance workers), (3) provide direct personal care in health care and residential settings, assist with health care procedures, and perform routine tasks to support health services (eg, health care assistants and home-based personal care workers), (4) provide health management and support (eg, biomedical engineers, medical physicists, clinical psychologists, social workers, medical secretaries, ambulance drivers, and other professional, technical, administrative, and support staff), and (5) participate in the health labor market by providing health services, including medical interns and trainees offering clinical services as part of their education. Additionally, we also included studies on students of various health care–related fields (eg, medicine, nursing, paramedics, and health sciences).

#### Interventions

Any type of intervention was eligible if aimed at enhancing COVID-19 pandemic–related HL or at least one of the four HL components, or a related competency conceptually linked to these components. Moreover, educational or training interventions were eligible when their content or intended outcomes could be meaningfully aligned with the underlying HL framework. Therefore, we also included interventions addressing COVID-19–related knowledge, skills, and competencies as HL proxies, here referred to as indicators. Eligible intervention approaches included, for instance, digital interventions (eg, self-help tools delivered via apps and online training sessions via video), group-based or individual interventions, and educational interventions (eg, lectures and workshops) delivered by scientists, educators, or HPs. We excluded experimental interventions such as manipulations examining cognitive processes. Further, for intervention studies that target behavior changes without a discernible link to HL-related competencies, or mental health outcomes, mental HL was judged as not eligible. Interventions, which contain exposure to other epidemic or pandemic infectious disease outbreaks (eg, SARS-CoV-1 and MERS [Middle East respiratory syndrome]) or chronic infectious diseases (eg, tuberculosis and hepatitis) were also excluded.

#### Outcomes

This systematic review refers to the integrated model of HL by Sørensen et al [20]. We adapted the definition of HL in the context of COVID-19 as “knowledge and competences to access, understand, appraise, and apply COVID-19 pandemic–related health information in order to make judgments and take decisions in everyday life concerning COVID-19 infection prevention” [17,20]. Based on this definition, the primary outcomes were COVID-19–related HL as well as components and indicators (eg, knowledge and skills), assessed using either objective or subjective instruments.

As validated instruments specifically measuring COVID-19–related HL among HPs were largely unavailable during the early phase of the pandemic [43], we considered observable indicators reflecting HL competencies. These indicators were not regarded as equivalent to the full construct of HL, but as proxy measures for specific components within the underlying framework. Within the integrated HL model, knowledge and related competencies are required for processing and using health information. Knowledge tests were therefore considered proxy indicators of the “understand” component, while performance-based infection prevention skills were considered indicators of “apply” for the purpose of this review. Performance or achievement indices and COVID-19–specific knowledge tests were eligible when they could be conceptually linked to objectively assessed HL-related competencies. Subjective HL comprised self-reported judgments about how well individuals perceive themselves to know and be able to access, understand, appraise, and apply COVID-19–specific health information.

With regard to “knowledge” as an HL indicator, we distinguished between general and specific COVID-19 knowledge. General COVID-19 knowledge covered a broad range of basic themes, including, but not limited to, disease symptoms, routes of transmission, incubation periods, and infection prevention and control measures. Specific COVID-19 knowledge referred to more focused areas, such as vaccination, handling of PPE, infection management strategies, mechanisms of virus transmission, or detailed infection prevention and control measures.

Further, skills were considered as HL-related indicators only when they reflected the application of COVID-19–specific infection prevention knowledge rather than purely technical clinical procedures. In this review, the indicator “skill” referred to the ability or capacity of protecting oneself and/or others (eg, patients, colleagues, and family members) from a SARS-CoV-2 infection through correctly performing recommended prevention control measures such as donning and doffing of PPE, hand hygiene, surface disinfection, wearing a mask, managing biomedical waste, and conducting nasopharyngeal swabs.

The secondary outcomes were classified and conceptually assigned to the underlying model [20]: prevalence of infection prevention behavior and adherence to recommended infection prevention measures (health behavior), attitudes toward recommended infection prevention measures and perceived confidence and self-efficacy (determinants), usage and frequency of consultation of trusted sources of information (access), and perceived or objective health status, health-related quality of life, and frequency of health care usage (health outcomes). Behavioral outcomes were not considered as direct measures of the “apply” facet but were interpreted as behavioral outcomes potentially influenced by HL competencies.

#### Study Types

We included randomized controlled trials (RCTs, eg, individual and cluster RCTs), nonrandomized studies of interventions (NRSIs; eg, quasi-randomized and controlled trials), and uncontrolled before-and-after studies defined as one-group prepost designs in which outcomes are measured before and after an intervention in the absence of a comparison group [44]. Studies that did not report an interventional design or a before-and-after comparison were excluded.

### Data Collection and Analysis

#### Selection of Studies

All identified references were deduplicated after being uploaded into supportive software (EPPI-Reviewer [45] and Rayyan [46]). Each title and abstract was screened by two independent reviewers according to predefined eligibility criteria. Full-text papers of potentially relevant references were reviewed by the same reviewers for inclusion. Any discrepancies were solved by discussion or by consultation with a third reviewer until consensus was reached. Studies that did not meet our eligibility criteria were excluded.

When multiple records referred to the same study population (eg, study protocols with subsequent completed publications or abstracts with full-text reports), we included only the most complete and relevant version.

#### Data Extraction and Management

We designed a data extraction form in Microsoft Excel, with predefined items that were pilot tested based on 5 included studies. At least two reviewers performed data extraction. Hereby, one reviewer extracted relevant data. A second reviewer checked the data extraction table for accuracy. Disagreements were resolved by discussion or through arbitration by a third reviewer when consensus was not reached. In case of insufficient data, we contacted the authors of the studies and requested further information. We extracted in detail the following information presented in Table 1.

**Table 1.** Overview of extracted data for analysis.

Study characteristics	Authors, journal, year of publication, geographical location/country, study design, study duration (year/period of study implementation), theoretical foundation, funding sources, disclosure of conflict of interest
Intervention characteristics	Type of intervention, intervention duration, frequency, response rate, follow-up period, and delivery mode of the intervention
Participant characteristics	Age, sex, number of participants recruited/allocated/evaluated, health profession, setting (eg, hospital and community setting)
Study outcomes	Primary outcome categories: COVID-19–related health literacy, at least one facet (access, understand, appraise, or apply) or indicator (eg, knowledge and skills) Secondary outcomes categories: Prevalence of, adherence to, attitudes toward infection prevention behavior, consultation of trusted sources of information, confidence and self-efficacy, health status, and health-related quality of life

#### Assessment of Risk of Bias Within Included Studies

Risk of bias was assessed by groups of two independent reviewers (CH, AAS, PK, EIG, ML, and JS) using the Cochrane RoB 2 Tool (Cochrane Risk of Bias 2 Tool) [47] for RCTs, the ROBINS-I (Risk of Bias in Nonrandomized Studies of Interventions) [48] for NRSIs tool and the EPHPP-QAT (Quality Assessment Tool for Quantitative Studies) [49] for uncontrolled before-and-after studies. The tools consist of several domains, which are shown in Table 2. Eligible studies were appraised for each domain of the respective risk of bias tool. Based on the overall rating, the level of risk of bias for each domain was judged for the RoB 2 tool (“low risk,” “some concerns,” and “high risk”), for ROBINS-I (“low risk,” “moderate risk,” “serious risk,” “critical risk,” and “no information”) and for EPHHP-QAT tool (“strong,” “moderate,” and “weak”) [47-49]. For the overall judgment of risk of bias using the Cochrane RoB 2 Tool, an algorithm generated a proposed assessment for each domain based on the highest score on an individual domain. The overall judgment for outcome assessments with ROBINS-I was based on a mapping of domain-level ratings. Both overall judgments are informed by the responses to the signaling questions. The overall score for each domain of the EPHPP-QAT was used to determine the global rating for uncontrolled before-and-after studies, with scores ranging from 1.00 to 1.50 categorized as “weak,” 1.51 to 2.50 as “moderate,” and up to 3.00 as “strong” [49].

**Table 2.** Risk of bias assessment domains of the tools used in the systematic review.

Tools and domains	Items (n)
Cochrane RoB 2 tool^a^, Sterne et al [47], domains (n=5)	
Bias arising from the randomization process	3
Bias due to deviations from intended interventions	7
Bias due to missing outcome data	4
Bias in measurement of the outcome	5
Bias in selection of the reported result	3
ROBINS–I^b^, Sterne et al [48], domains (n=7)	
Bias due to confounding	8
Bias in selection of participants into the study	5
Bias in classification of interventions	3
Bias due to deviations from intended interventions	6
Bias due to missing data	5
Bias in measurement of outcomes	4
Bias in selection of the reported result	3
EPHPP-QAT^c^, Armijo-Olivo et al [49], domains (n=6)^d^	
Selection bias	2
Study design	4
Confounders	2
Blinding	2
Data collection	2
Withdrawals/dropouts	2

a Cochrane RoB 2 Tool: Cochrane Risk of Bias 2 Tool.

b ROBINS–I: Risk of Bias in Nonrandomized Studies of Interventions.

c EPHPP-QAT: Quality Assessment Tool for Quantitative Studies.

d The EPHPP tool comprises eight domains, with the risk of bias being assessed based on six of these [49].

#### Certainty of Evidence

The overall certainty of the evidence by outcome was determined according to the GRADE (Grading of Recommendations, Assessment, Development, and Evaluation) [50] approach by two independent reviewers (CH and EMS). The quality of the body of evidence was assessed based on four levels of certainty—“high,” “moderate,” “low,” and “very low” [50,51]. Considering the study design (RCTs or observational studies, eg, before-and-after studies), the quality of the eligible studies was either downgraded referring to five reasons (risk of bias, inconsistency, indirectness, imprecision, and publication bias) or upgraded based on three reasons (large effect, dose response, and all plausible residual confounding) [50]. If any discrepancies between the reviewer judgments occurred, a third reviewer (PK) was consulted.

#### Data Synthesis

Study characteristics and results were narratively summarized and presented in a summary of findings table. When at least two studies were judged sufficiently similar in terms of intervention, outcome measurement, and time point, a meta-analysis was performed [42]. We calculated Hedges *g* for each comparison to correct for small sample bias. If means and SDs were not reported, we converted other statistics such as medians and IQRs or odds ratios to Hedges *g* where possible [42,52,53]. To pool effect sizes, we used random effects meta-analysis with the inverse variance method, as we expected the true intervention effects to differ due to considerable remaining between-study heterogeneity. If a study contributed more than one effect size, a three-level random effects meta-analysis model was used, with effect sizes nested in studies [54]. The heterogeneity variance $\tau^{2}$ was estimated using the Restricted Maximum-Likelihood Estimator [55]. We used the Hartung-Knapp-Sidik-Jonkman method for calculating test statistics and CIs to account for uncertainty in the heterogeneity estimation, where appropriate, given the number of studies and the extent of between-study heterogeneity [56]. Statistical significance was defined at a 2-sided *P* value of less than .05. The heterogeneity variance $\tau^{2}$ and the $I^{2}$ statistic were reported along with their respective CIs as measures of heterogeneity. $I^{2}$ statistics ranging from 0% to 40% were interpreted as “might not be important,” from 30% to 60% as “moderate,” from 50% to 90% as “substantial,” and from 75% to 100% as “considerable” [42]. For values falling in overlapping ranges (eg, 55% or 80%), we interpreted them in context and used them for broad orientation rather than strict categorization. In addition, we also considered *τ*² as an absolute measure of heterogeneity to support our assessment of heterogeneity and calculated 95% prediction intervals (PIs) indicating the range in which effects of future studies are expected based on present evidence using the Nagashima method [57]. However, for analyses including only a single study, PIs were not estimated as between-study heterogeneity could not be assessed [57]. If heterogeneity was moderate to high and the analysis included more than five studies, subgroup analysis using study- or result-level variables such as risk of bias was conducted to explore heterogeneity. If the analysis included ten or more comparisons, funnel plots and the Egger test were applied to assess potential publication bias [58]. The results were visualized in forest plots where studies were ordered by weight in ascending order. Analysis was conducted in R Studio (version 2024.04.1 for Windows; R Foundation) using the packages *meta* [59] and *metafor* [60].

#### Deviation From Study Protocol

During the review process, several deviations from the original protocol were introduced in response to characteristics of the identified literature. All deviations were implemented before the final data synthesis. First, the original protocol did not restrict the target population, as we initially assumed that only a limited number of eligible studies would be available. During the review process, it became evident that a considerable proportion of potentially relevant studies targeted various populations. To ensure conceptual comparability of PICO-criteria, the population was restricted to HPs.

Second, the protocol initially did not specify language restrictions to maximize the sensitivity of the search. As a substantial number of studies originated from different linguistic contexts, a language restriction was introduced to ensure accurate assessment of eligibility and data extraction. This decision was based on the research team’s present proficiency in English, French, and German. Spanish studies were translated using the artificial intelligence translation tool DeepL by DeepL SE, and ChatGPT by OpenAI, followed by an expert verification.

Third, although gray literature and preprints were searched, only completed studies were included in the present analysis to ensure methodological rigor and robustness of the findings. Ongoing studies were documented for potential inclusion in future updates.

Fourth, the protocol did not specify an approach for assessing uncontrolled before-and-after studies. Therefore, these studies were evaluated using the EPHPP-QAT, ensuring a standardized assessment of methodological quality.

Fifth, during data extraction, we observed discrepancies between reported intervention content and measured outcomes, and combined multiple outcomes in one single measure. To adequately capture these measures and provide a more comprehensive evaluation of the interventions, studies reporting composite outcomes were considered for inclusion into the review.

Finally, while the protocol prespecified detailed procedures for addressing heterogeneity and conducting meta-analyses, substantial variability across interventions and outcome measures required a more cautious analytical approach. Therefore, the meta-analyses were conducted only when studies were deemed sufficiently comparable and were interpreted as exploratory. These listed deviations did not affect the overall conclusions of this review.

## Results

### Overview

**Figure 1.**
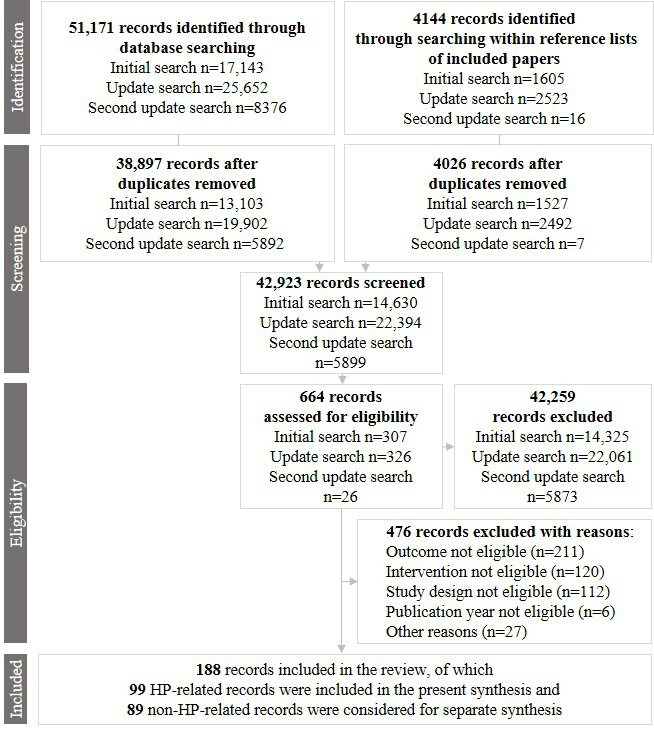
Flowchart diagram according to PRISMA (Preferred Reporting Items for Systematic Reviews and Meta-Analyses) statement 2020 [38]. Other reasons: study protocols and study registries excluded due to already included full-text publication, not eligible context or population. HP: health professional.

The results of the study search and selection process are shown in Figure 1. The search yielded a total of 51,171 papers, of which 188 studies were deemed relevant. Among these, 89 studies addressed the target population of non-HPs, while 99 focused on HPs. Given the considerable number of potentially relevant studies, the present systematic review primarily focused on the population of HPs. The results about other populations will be presented in a separate publication. For future updates, we identified ongoing studies for potential inclusion; however, only published studies have been included in the present analysis to report the outcomes. In the additional file Multimedia Appendix 2 [61-188], Table S1 shows the excluded studies with reasons provided.

### Study Characteristics

Of the included studies, there were 15 RCTs [61-74], 4 NRSIs [75-78], 74 uncontrolled before-and-after studies [79-138,188], 5 ongoing studies [139-143], and 1 study as awaiting classification [144]. The references were published between 2020 and 2025. Most studies (96/99 studies, 97%) were published in English and 3 studies were published in Spanish. In Multimedia Appendix 2, the Tables S2-S4 show the summarized characteristics of included studies.

### Population

In total, 329,623 HPs were enrolled in interventional studies, ranging from 18 to 234,643 participants per intervention. Most participants were enrolled in uncontrolled before-and-after studies (n=327,298), whereas substantially fewer participants were included in RCTs (n*=*2034) and NRSIs (n=291). Most interventions were directed toward a broad range of mixed health occupational groups, accounting for 43% of studies (40/93 studies, 298,220 participants). Interventions targeted particularly at students from various disciplines, including medicine, nursing, dentistry, or pharmacy, were examined in 29% of studies (27/93 studies, 4388 participants). Five of the 93 (5%) studies were conducted in both HPs and non-HPs (9068 participants). Table 3 shows the distribution of study populations by study design in more detail. RCTs were predominantly conducted among students and specialist medical practitioners, whereas uncontrolled before-and-after studies more frequently included mixed HP groups, nursing professionals, pharmacists, midwives, and students. Therefore, the distribution of professional groups differed across study designs.

The average age of participants was 29.9 (SD 8.8) years, as reported in 37% (34/93 studies) of studies. Further, 64% of the participants were female (65 studies). Most studies were conducted in Asia (48/93 studies, 52%), North America (17/93 studies, 18%), and Europe (15/93 studies, 16%). Overall, of the 93 included studies 39 (42%) took place in high-income countries, 27 (29%) in lower-middle countries, 26 (28%) in upper-middle-income countries, and 3 (3%) in low-income countries.

**Table 3.** Study population of included studies (N=93) by study designs.

Study population	Participants, n total	Studies, k total	RCT^a^, k (n participants)	NRSI^b^, k (n participants)	UC^c^, k (n participants)
Health professionals^d^ (n=7451, k=12)					
Generalist medical practitioners	5886	1	—^e^	—	1 (5886)
Specialist medical practitioners	126	2	2 (126)	—	—
Nursing professionals	950	7	1 (344)	—	6 (606)
Pharmacists	279	1	—	—	1 (279)
Midwives	210	1	—	—	1 (210)
Health associate professionals (n=237, k=4)					
Health care laboratory professionals	52	1	—	—	1 (52)
Environmental, occupational health, and hygiene professionals	77	1	—	—	1 (77)
Community health workers	108	2	1 (18)	—	1 (90)
Students (n=4388, k=27)					
Medical students	571	7	1 (29)	1 (129)	5 (413)
Nursing students	1537	9	—	1 (50)	8 (1487)
Paramedics students	203	2	2 (203)	—	—
Dental students	118	1	—	—	1 (118)
Pharmacy students	85	1	—	—	1 (85)
Respiratory therapist students	37	1	—	—	1 (37)
Mixed student disciplines	1837	6	2 (444)	—	4 (1393)
Mixed health professional groups (n=317,297, k=50)					
Various health professional occupation groups	298,220	40	5 (581)	2 (112)	33 (297,527)
Students and health professionals	10,009	5	1 (21)	—	4 (9988)
Health professionals and nonhealth professionals	9068	5	—	—	5 (9068)
Health professional occupation not specified (n=250, k=1)					
	—	1	1 (250)	—	—

a RCT: randomized controlled trial.

b NRSI: nonrandomized study of interventions.

c UC: uncontrolled before-and-after studies.

d Categorization in accordance with the World Health Organization classification of health workers [9].

e Not applicable.

### Interventions and Comparators

The type of intervention, delivery mode, and methods, settings, and comparator varied widely across the included studies in Table S2 in Multimedia Appendix 2. We identified three predominant intervention types: (1) educational interventions, (2) training programs, and (3) blended intervention approaches. Overall, 61 of the 93 (66%) studies applied educational interventions with traditional teaching of theoretical knowledge. Six (6%) studies provided training programs comprising demonstrations and the performance of practical, hands-on skills (eg, donning and doffing of PPE). Within 26 of the 93 (28%) studies, blended interventions were conducted, which combined both theoretical and practical approaches. While educational interventions were the most common intervention type across all study designs, they were predominantly evaluated in uncontrolled studies. Training programs were mainly evaluated in controlled study designs, whereas blended approaches were evaluated in both controlled and uncontrolled studies (Table 4). In controlled studies, comparators most commonly consisted of educational approaches delivered in formats similar to the intervention (Table 5), indicating that many studies compared alternative educational strategies rather than using a passive control group.

**Table 4.** Intervention characteristics by study designs (N=93).

Intervention characteristics	Studies, k total (%)	RCT^a^, k (%)	NRSI^b^, k (%)	UC^c^, k (%)
Type of interventions				
Educational interventions	61 (66)	9 (10)	2 (2)	50 (54)
Training programs	6 (6)	2 (2)	—^d^	4 (4)
Blended intervention approaches	26 (28)	4 (4)	2 (2)	20 (22)
Guidance				
Self-guided	16 (17)	5 (5)	1 (1)	10 (11)
Provider-guided	43 (46)	6 (6)	1 (1)	36 (39)
Self- and provider-guided	32 (34)	4 (4)	2 (2)	26 (28)
Delivery mode				
Group	50 (54)	9 (10)	2 (2)	39 (42)
One-to-one	25 (27)	7 (8)	1 (1)	17 (18)
In-person	29 (31)	3 (3)	—	26 (28)
Online distance learning	43 (46)	6 (6)	1 (1)	36 (39)
Blended learning (ie, online and in-person combined)	11 (12)	5 (5)	—	6 (6)
Technologically enhanced in-person learning	6 (6)	2 (2)	2 (2)	2 (2)
Information on delivery mode not available	7 (8)	—	—	7 (8)
Delivery method				
Technology-enhanced methods				
e-learning (through digital resources and platforms)	20 (22)	4 (4)	—	16 (17)
Virtual reality simulation	3 (3)	—	2 (2)	1 (1)
Interactive methods (eg, workshops)	3 (3)	1 (1)	—	2 (2)
Lecture-based methods	10 (11)	—	—	10 (11)
Self-directed methods	5 (5)	3 (3)	—	2 (2)
Practice-based methods (eg, demonstrations and role play)	5 (5)	—	1 (1)	4 (4)
Gamified methods				
Simulation games	1 (1)	—	1 (1)	—
Educational games	2 (2)	—	—	2 (2)
Blended methods	51 (55)	13 (14)	—	38 (41)
Information on delivery method not available	6 (6)	—	—	6 (6)

a RCT: randomized controlled trial.

b NRSI: nonrandomized study of interventions.

c UC: uncontrolled before-and-after studies.

d Not applicable.

**Table 5.** Comparator characteristics by study designs (N=93).

Type of comparator	Studies, k total (%)	RCT^a^, k (%)	NRSI^b^, k (%)	UC^c^, k (%)
Educational intervention	13 (14)	10 (11)	3 (3)	—^d^
Training programs	1 (1)	1 (1)	—	—
Blended intervention approaches	2 (2)	2 (2)	—	—
No intervention	1 (1)	—	1 (1)	—
Placebo intervention	1 (1)	1 (1)	—	—
Usual care	1 (1)	1 (1)	—	—
Waitlist	2 (2)	2 (2)	—	—
No control group	74 (80)	—	—	74 (80)

a RCT: randomized controlled trial.

b NRSI: nonrandomized study of interventions.

c UC: uncontrolled before-and-after studies.

d Not applicable.

HPs (27/93 studies, 29%), health educators (10/93 studies, 11%), trained instructors (13/93 studies, 14%), and researchers (18/93 studies, 19%) delivered the interventions. Universities (23/93 studies, 25%) and hospitals (33/93 studies, 35%) were the most prevalent settings. The duration of interventions lasted from 10 minutes to 19 weeks. Most of the interventions were provided in groups (50/93 studies, 54%), followed by one-to-one sessions (25/93 studies, 27%). Online distance learning (43/93 studies, 46%) was more prevalent than in-person teaching (29/93 studies, 31%) and hybrid learning (11/93 studies, 12%).

### Underlying Frameworks for Guiding Interventional Studies

Among 93 studies, 41 (44%) reported a theoretical framework or model for guiding processes such as development, implementation, and evaluation of the intervention. Hereby, 6 studies (6%) referred to learning models (Peyton’s approach [64], Miller’s pyramid-based training [127], cognitive learning theory [91,100,103], and simulation-based mastery learning [116]) and 2 (2%) applied health behavior change models (eg, health belief model [66] and theory of change model [112]). Further, two (2%) studies were based on Kirkpatrick’s evaluation model [110,145]. Health manuals, COVID-19 protocols, and clinical information (eg, WHO protocols and Infection Prevention Control guidelines) were applied in 27 (29%) studies. While 2 (2%) of the included studies incorporated HL as a component within the curriculum [188] or as an underlying concept for an online learning platform [121], no study explicitly referred to an HL model or provided an established definition.

### Outcomes Measurement

The vast majority of studies reported primary outcomes related to changes in COVID-19 knowledge (75/93 studies, 81%). Of the 93 studies, 20 (22%) assessed general knowledge of COVID-19, while 43 (46%) focused on specific knowledge outcomes, primarily concerning infection prevention and control measures (19 studies, 20%), PPE performance (8 studies, 9%), COVID-19 vaccine (9 studies, 10%), SARS-CoV-2 transmission (5 studies, 5%), and infection management (4 studies, 4%). In 21 (23%) studies [62-64,67-69,72,73,75,78,84,91,92,97,106,116,122,127,129,146,147] changes of COVID-19 infection prevention skills were targeted, with enhancement of PPE performance being the most frequently addressed outcome (13 studies, 14%).

In total, 38 of the 93 (41%) studies [62,65,66,74,76,78-80,83,84,86,87,90,93,97,101,104,105,108,111,112,119,123,130-132,135,146-155,188] reported secondary outcomes such as confidence and self-efficacy (19 studies, 20%), compliance toward and prevalence of infection prevention measures behavior (6 studies, 7%), attitudes (11 studies, 12%), and composite outcomes (21 studies, 23%). One study [121] developed an online learning platform aimed at fostering HL and focused in particular on knowledge, attitudes, and practices. Another study [62] reported adverse events related to motion sickness caused by the use of virtual reality simulation. Among 21 studies (23%) a discrepancy was observed between the reported outcomes and the items actually used to measure the intended construct.

Most studies (65/93 studies, 70%) measured outcomes at short-term follow-up, defined as immediately following the intervention for up to four weeks from the program’s start. Of 93 studies, 8 (9%) reported medium-term follow-up, ranging up to six months. Additionally, 9 (10%) studies determined outcomes at a second follow-up ranging from one week to eight months after completing the intervention. In 19 (20%) studies, the time point of the postintervention outcome assessment was not clearly reported.

The assessment differed across all 93 studies. The content of the assessment tools was based on hospital internal protocols [68,72,73,83], national and international guidelines, such as those provided by the WHO [84,91,100,146,156], the Centers for Disease Control and Prevention [91,116,134], or the Ministry of Health [84,153]. Further, 60 studies (65%) applied self-developed instruments, created for the study. Additionally, 26 (28%) studies reported information on the development of the assessment tool. Among 37 (40%) studies using a validated tool for outcome measurement, 11 (12%) studies did not provide details regarding the validation process and measurement properties. Most studies (82/93 studies, 88%) used participants’ reported measures with Likert scales, multiple-choice, “true”/“false” or “yes”/“no” response options. In 17 of the 93 (18%) studies [62-64,69,73,75,83,84,90-92,97,106,116,122,127,129], outcomes were assessed through performance-based evaluations conducted by an observer (eg, teacher, instructor, or member of the study team) using a checklist. In one study [188], the assessment tool included a HL-related item specifically.

Overall, the included studies indicated substantial conceptual overlap between COVID-19–related HL and educational or skills-based infection control interventions. Most studies focus on knowledge and procedural competencies, corresponding to the “understand” and “apply” facets, rather than explicitly addressing the full construct. By contrast, no study explicitly assessed COVID-19–related HL as an overall construct or examined changes in “access” and “critical appraisal” of health information (Table 6).

**Table 6.** Outcomes conceptually assigned to levels of the underlying health literacy model by study design (N=93).

Components and studies, n (%)	References
HL-related components	
Access	—^a^
Understand	
General COVID-19–related knowledge	
1 (1) RCT^b^	[66]
1 (1) NRSIs^c^	[77]
18 (19) UCs^d^	[84,89,92,93,95,102,103,110,112-115,117,137,146,152,156,188]
Specific COVID-19–related knowledge (eg, on vaccination, PPE^e^ and IPC^f^ measures)	
7 (8) RCTs	[61,64,70-73,149]
1 (1) NRSI	[78]
35 (38) UCs	[81-83,85,87,88,90,94-100,104,106-108,110,118,122,124-126,130,131,133,134,136,138,145,147,150,157,158]
Composite knowledge-related outcomes	
1 (1) NRSI	[76]
11 (12) UCs	[80,83,105,111,123,130,131,151,153-155]
Critically appraise	—
Apply	
General COVID-19–related infection prevention performance skills	
1 (1) NRSI	[78]
6 (6) UCs	[84,92,106,122,129,147]
PPE performance skills (donning and/or doffing)	
8 (9) RCTs	[63,64,67-69,71-73]
1 (1) NRSI	[75]
4 (4) UCs	[91,97,116,127]
Hand disinfection	
2 (2) RCTs	[62,64]
Nasopharyngeal swab performance	
1 (1) RCT	[62]
Composite skills-related outcomes	
1 (1) UC	[154]
HL-related factors	
Attitude	
2 (2) RCTs	[74,149]
1 (1) NRSI	[147]
8 (9) UCs	[84,92,93,109,112,146,150,152]
Confidence/self-efficacy	
7 (8) RCTs	[64-67,69-71]
1 (1) NRSI	[78]
11 (12) UCs	[83,87,96,97,101,104,105,108,119,128,188]
Prevention behavior	
Prevalence of COVID-19 IPC measures behavior	
1 RCT (1)	[66]
2 (2) UCs	[79,152]
Compliance to IPC measures	
1 (1) UC	[90]
Adherence to IPC measures	
2 (2) UC	[93,112]

a Not applicable.

b RCT: randomized controlled trial.

c NRSI: nonrandomized study of interventions.

d UC: uncontrolled before-and-after studies.

e PPE: personal protective equipment.

f IPC: infection prevention control.

### Risk of Bias Assessment

An overview of the risk of bias assessments of included studies is presented in detail in the additional file Multimedia Appendix 3 [61-138,145-158,188]. Among primary outcomes of RCTs (*k*=15) assessed with the Cochrane RoB 2 Tool, 14 of 26 (54%) compared results of primary outcomes were evaluated as “some concerns.” Primary outcomes of 6 of 26 (23%) results had “low” risk of bias, while another 6 of 26 (23%) results were rated at “high” risk of bias. Most results (16/26 studies, 62%) were rated at high risk of bias regarding the selection of the reported results due to a lack of prespecified statistical analysis plans or study protocols. Some studies [61,63] did not adequately handle missing outcome data.

Based on the ROBINS-I assessments for NRSIs, all six evaluated outcomes of 4 included studies were judged as having “serious” risk of bias. Pertaining to the risk of bias of uncontrolled before-and-after studies using EPHPP-QAT, 73 of 74 (99%) studies were rated as being “weak,” and 1 study as “moderate.” Most uncontrolled studies used a nonprobability purposive or convenience sampling technique, resulting in a higher risk of selection bias. Neither did they report information on the blinding of participants nor outcome assessors. All NRSI and uncontrolled before-and-after studies are prone to confounding (eg, preexisting COVID-19 knowledge, interest in gaining more knowledge, and socioeconomic status), but no study adjusted or controlled for potential confounding.

### Overview of Findings Pertaining to the Effectiveness of Interventions

We performed meta-analyses for changes of COVID-19–related knowledge and skills. Hereby, we restricted meta-analyses to RCTs and primary outcomes due to high heterogeneity across interventions and outcomes in both NRSIs and uncontrolled before-and-after studies and due to inconsistencies in secondary outcome definitions in RCTs. The results are presented in the summary of findings table, along with GRADE certainty of evidence ratings (Table 7). The summary of findings tables for all included studies, separated by study design, are shown in the additional file Multimedia Appendix 4 [61-138,145-158,188,189].

**Table 7.** Summary of findings. Interventions for enhancing COVID-19–related health literacy (HL) in health professionals.^a^

Outcomes	Relative effect (95% CI)	Participants, n (studies)	Certainty of the evidence (GRADE^b^)	Comments
Primary outcomes				
COVID-19–related knowledge				
General knowledge (F1)	—^c^	181 (2 nonrandomized studies) Buyego et al [76], Hu et al [77]	—^d^^,^^e^^,^^f^	Meta-analysis and the GRADE domain inconsistency were not applicable because metrics of outcomes differed widely across NRSIs^g^
Vaccine knowledge (F1)	SMD^h^ 1.00 (0.33; 1.67)	155 (1 RCT^i^) Alotaibi et al [61]	⨁◯◯◯ very low^e^^,^^j^^,^^k^	The included study contains three different intervention groups, on which the meta-analysis and the GRADE assessment were based
PPE^l^ knowledge (F1)	SMD 0.21 (−0.09, 0.51)	565 (4 RCTs) Currat et al [64], Suppan et al [70], Suppan et al [71], Xie et al [73]	⨁⨁◯◯ low^d^^,^^e^	Among 4 included studies, 1 study [70] reported outcomes in a metric that was not convertible; therefore, the meta-analysis was based on only three studies: Currat et al [64], Suppan et al [71], and Xie et al [73]
COVID-19–related infection prevention skills				
PPE performance doffing (F1)	SMD 0.69 (−0.95; 1.78)	154 (4 RCTs) Birrenbach et al [62], Christensen et al [63], Currat et al [64], Manggala et al [68]	⨁◯◯◯ very low^m^^,^^n^^,^^o^	—
PPE performing doffing (F2)	SMD 0.61 (−0.07; 1.29)	93 (2 RCTs) Birrenbach et al [62], Currat et al [64]	⨁◯◯◯ very low^p^^,^^q^^,^^m^	—
PPE performing donning (F1)	SMD 0.28 (−0.48; 1.04)	61 (2 RCTs) Christensen et al [63], Manggala et al [68]	⨁◯◯◯ very low^m^^,r^	—
PPE performing donning and doffing (F1)	SMD 1.95 (0.82; 3.09)	274 (3 RCTs) Li et al [67], Rueda-Medina et al [69], Xie et al [73]	⨁◯◯◯ very low^m^^,^^r^^,s^	—
Hand disinfection (F1)	—	93 (2 RCTs) Birrenbach et al [62], Currat et al [64]	—^p,^^q^^,^^d^	Meta-analysis and the GRADE domain inconsistency were not applicable because metrics of outcomes differed widely across RCTs
Hand disinfection (F2)	—	93 (2 RCTs) Birrenbach et al [62], Currat et al [64]	—^p^^,^^q^^,d^	Meta-analysis and the GRADE domain inconsistency were not applicable because metrics of outcomes differed widely across RCTs
Secondary outcomes				
Perceived confidence in the ability to use PPE	—	553 (4 RCTs) Currat et al [64], Li et al [67], Suppan et al [70], Suppan et al [71]	⨁◯◯◯ very low^r^^,^^d^^,t^	Meta-analysis and the GRADE domain inconsistency were not applicable because metrics of outcomes differed widely across RCTs.

a Population: health professionals of various professions; setting: various health care settings; intervention: various interventions; comparison: various comparators.

b GRADE: Grading of Recommendations, Assessment, Development, and Evaluation.

c Not applicable.

d As the sample size is very small in these studies, we rated down the quality of evidence by one level for imprecision.

e The proportion of information from studies at high risk is sufficient to affect the interpretation of the results; crucial limitation for one criterion, or some limitations for multiple criteria, sufficient to lower confidence in the estimated effect.

f As the metrics of the results differ widely across the studies, we have downgraded the quality of certainty by one level.

g NRSI: nonrandomized study of interventions.

h SMD: standardized mean difference.

i RCT: randomized controlled trial.

j A study sample based on one study and that covers only a subgroup of the population of interest.

k We rated down the quality of evidence by one level due to the very small sample size.

l PPE: personal protective equipment.

m As the sample size is very small in both studies, and the CI includes appreciable benefit and no effect, we rated down the quality of evidence by two levels for imprecision.

n Most information from results is at low risk or has some concerns, but 1 study was at high risk of bias (missing data domain). Potential limitations are likely to lower confidence in the estimated effect.

o Minor CI overlap. Wide variance of point estimates. *I*2 implies moderate to substantial heterogeneity. Heterogeneity cannot be explained.

p Most information from results is at low risk of bias or which were rated as some concerns (selection bias and performance bias). Plausible bias that raises some doubt about the results. Potential limitations are likely to lower confidence in the estimated effect.

q Very few studies that address only a subgroup of the population of interest (eg, medical students) compared according to the underlying World Health Organization classification of health workers.

r The proportion of information from studies from both at high risk and with some concerns is sufficient to affect the interpretation of the results. A crucial limitation for one criterion, or some limitations for multiple criteria, is sufficient to lower confidence in the estimated effect.

s CI overlap. Wide variance of point estimates. *I*2 implies moderate to substantial heterogeneity. Heterogeneity cannot be explained.

t As the effects differ widely across the studies, we have downgraded the quality of certainty by one level.

### Effectiveness of Interventions in Enhancing COVID-19–Related Knowledge

**Figure 2.**
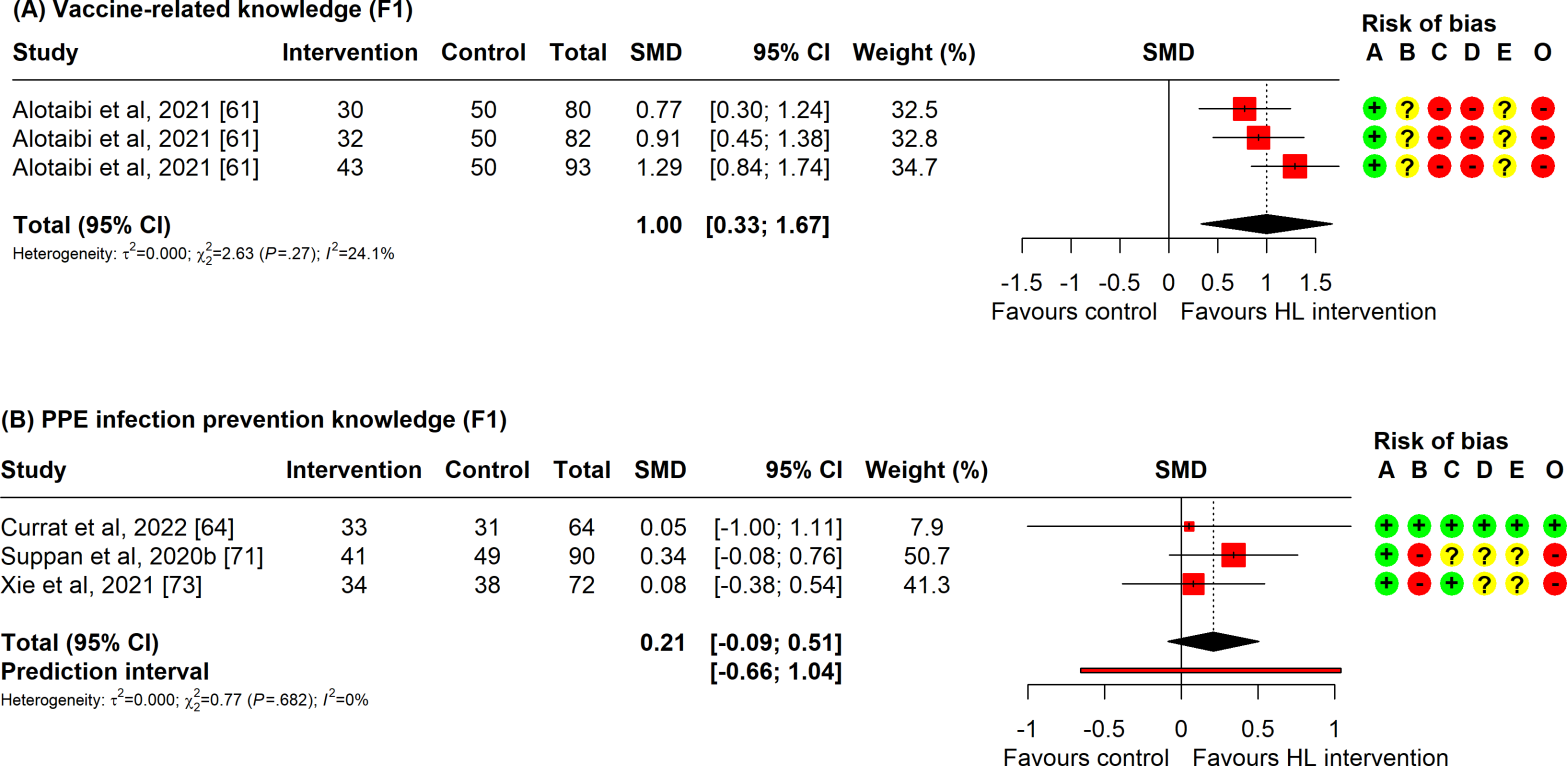
COVID-19–related knowledge. SMD: standardized mean difference.

Data from four RCTs were considered for the meta-analysis on the outcome COVID-19–related knowledge, grouped into knowledge on vaccine and PPE (Figure 2). COVID-19-relevant HL interventions may increase vaccine knowledge at postintervention (standardized mean difference [SMD] 1.00; 95% CI 0.33 to 1.67, *I*^2^=24%), but the evidence is very uncertain about the effect. Further, the evidence is uncertain and suggests that interventions may not increase PPE knowledge at postintervention (SMD 0.21; 95% CI −0.09 to 0.51, *I*^2^=0%).

### Effectiveness of Interventions in Enhancing COVID-19–Related Infection Prevention and Control Skills

**Figure 3.**
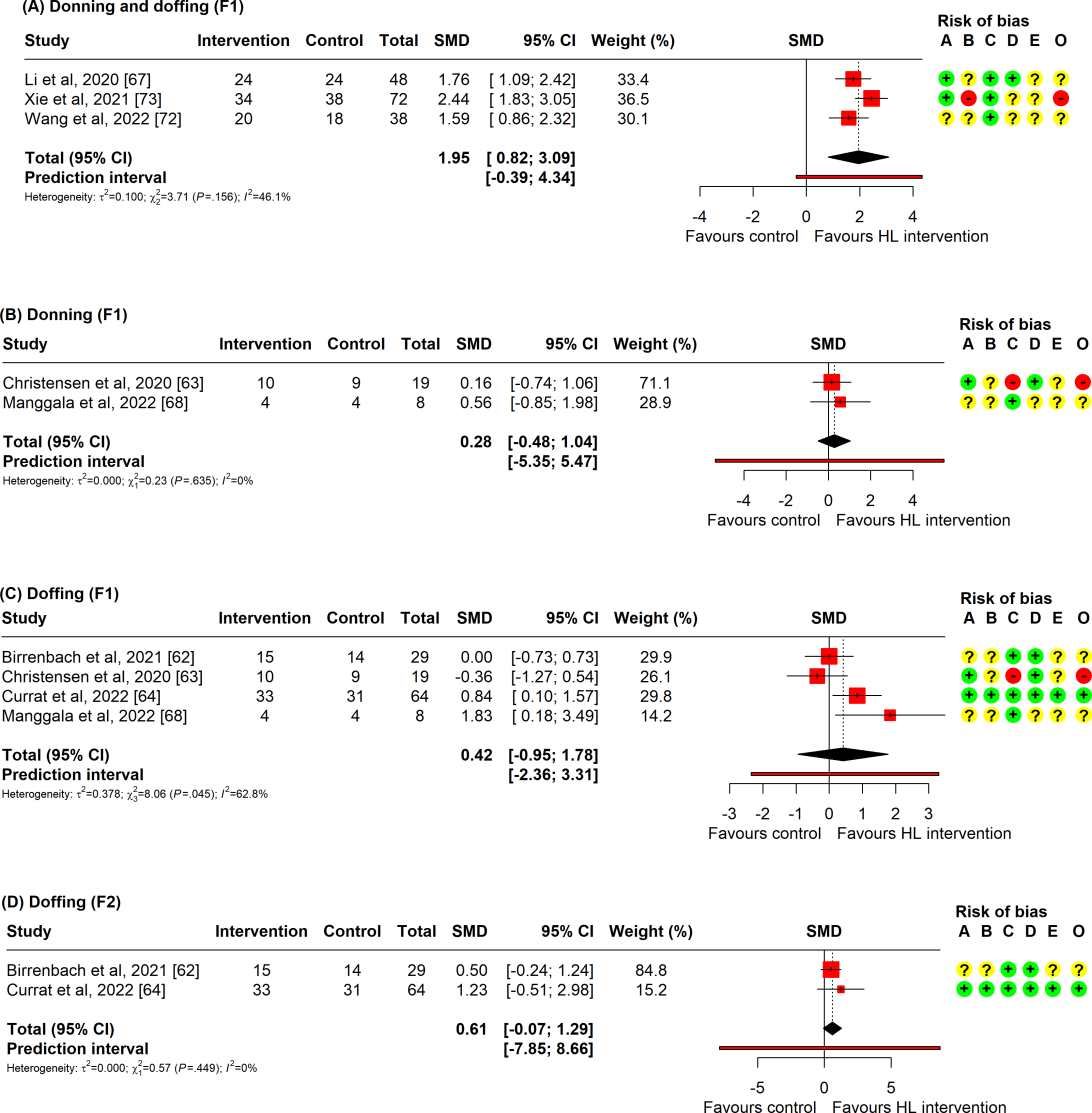
COVID-19–related PPE donning and doffing. PPE: personal protective equipment; SMD: standardized mean difference.

Of the 15 RCTs included, 7 RCTs contributed data to the meta-analysis (Figure 3). They reported on COVID-19–related infection prevention and control performance and were categorized into competencies in PPE donning, doffing, or both. The HL-relevant interventions may increase postintervention donning and doffing skills, though the evidence remains very uncertain (SMD 1.95; 95% CI 0.82 to 3.09, *I*^2^=46.1%). The evidence is very uncertain about the effect of interventions on doffing skills at postintervention (SMD 0.42; 95% CI −0.95 to 1.78, *I*^2^=62.8%) and at follow-up (SMD 0.61; 95% CI −0.07 to 1.29, *I*^2^=0%), as well as on donning skills at postintervention (SMD 0.28; 95% CI −0.48 to 1.04, *I*^2^=0%).

In addition to the findings of the meta-analysis, a narrative description of the 74 uncontrolled before-and-after studies is provided in Multimedia Appendix 5 [94,107,112].

## Discussion

### Principal Findings

This systematic review aimed to identify and critically evaluate evidence pertaining to interventions that focus on enhancing COVID-19–related HL in HPs. Meta-analysis revealed favorable changes in COVID-19–related infection prevention and control skills after the intervention, particularly in the performance of PPE donning and doffing, which are essential competencies for reducing the risk of health care–associated infections. These findings are similar to those of Saunders et al [190], who systematically investigated the impact of HL training in HP students.

However, the pooled effect sizes must be interpreted with caution. Although some effect sizes were large, they were derived from a small number of studies with limited sample sizes, which increases the risk of imprecision and unstable effect estimates. Small studies are also more susceptible to bias and may overestimate intervention effects. This is reflected in the GRADE assessment, which indicated low to very low certainty of evidence due to risk of bias, inconsistency, and imprecision. Further, many interventions targeted only a subgroup of the population of interest, for example, medical students. Whether the reported results can be applied to the population of HPs at large remains unclear [9]. Thus, the identified body of evidence can largely be characterized by high inconsistency. While the pooled effect estimate suggested a statistically significant effect for the outcomes COVID-19 vaccine-related knowledge and competencies in PPE donning and doffing at postintervention, the PI included nonsignificant and both potentially beneficial and harmful effects, indicating considerable variability and limiting the generalizability of effects across intervention approaches, HP occupational populations, settings, and outcomes. This finding is consistent with the results of similar reviews [190,191].

Moreover, the small sample sizes in most of the included studies may have led to a failure to detect meaningful differences due to insufficient statistical power. They might be attributed to the high workload of HPs, which was exacerbated during the onset of the pandemic, and associated with time constraints, limited personnel resources, and high rates of illness among infected staff, perceived exhaustion, and pandemic fatigue [6,8]. Many studies used nonprobability purposive or convenience sampling techniques, which are associated with a higher risk of bias. This might also be linked to the ad hoc nature of the interventions, which were implemented in response to the pandemic. Further, the high risk of bias in most studies weakens confidence in the findings. NRSIs are prone to confounding. For instance, participants may already possess a higher level of knowledge about COVID-19 due to extensive media coverage or a general interest in acquiring more information. In uncontrolled before-and-after studies, we cannot with certainty attribute observed effects to the intervention, as there is no control group.

None of the included studies were based on a theoretical framework of HL, aligning with findings of previous studies [7,37,191]. It appears that COVID-19 HL has more often been addressed in cross-sectional than in interventional studies [7,37]. Baumeister et al [191] concluded that the integrated HL model [20] might not be as familiar with the model as desired among researchers. More than half of the studies were conducted in Asia and North America, regions where the model might be less known, as it was developed in Europe [191,192].

Further, the identified body of evidence revealed conceptual overlap between COVID-19–related HL and educational or skills-based interventions in the context of COVID-19 prevention and infection control. Most included studies were not designed as explicit HL interventions. Instead, COVID-19–related HL was frequently operationalized indirectly through knowledge- and skills-related indicators rather than validated HL instruments. These indicators were relevant to the present review as they could be mapped onto specific HL facets, particularly “understand” and “apply.” However, they should not be interpreted as equivalent to the full construct of COVID-19–related HL. None of the studies examined all four facets of HL, and the facets relating to “access” and “critical appraisal” were not explicitly addressed. The available evidence should therefore be understood as HL-relevant, but only partly HL-specific. Nevertheless, the theoretical foundation remains essential for systematically developing, comparing, and refining interventions [193]. Most of the studies investigated socio-cognitive outcomes combined, such as knowledge, attitudes, and practical skill acquisition, which align more closely with the Knowledge, Attitudes, and Practices framework [194].

It should also be noted that the intended outcomes were primarily assessed subjectively through self-report. Self-assessments of participants pertained to self-reported judgments about how well a person perceives themselves to know COVID-19–specific health information. This often leads to over- or underestimation of their own knowledge or confidence, and can also evoke social desirability bias, which might affect confidence in the results [195]. Miller [196] highlighted the significant impact of measurement moderators on the effect sizes of HL interventions. Their findings indicated that effect sizes, and consequently the observed improvements, were greater when HL was assessed subjectively rather than through objective measures by performance or achievement indices [196].

Moreover, the constructs used in the instruments were often merged, making it unclear which specific outcome was being measured, potentially leading to over- or underestimation of the results within the study. In 21 studies, a mismatch between the reported intervention content and the measured outcomes was observed. This finding is also consistent with the results of Walters et al [193]. Therefore, uncertainty regarding composite outcomes cannot be ruled out.

In more than half of the studies (60 studies), new questionnaires were developed; however, 26 studies reported the development process. Limited information regarding the development process and the origin of the items was observed. These findings compare with those of Grepmeier et al [7]. The lack of detailed information could be attributed to the need for implementing and conducting interventions rapidly, given the urgency to quickly develop and foster pandemic-related competencies and knowledge. Instruments for assessing COVID-19–related HL in respective countries may not yet be widely available or accessible in various languages. Currently, only a few instruments address COVID-19 HL, such as the validated tools developed in Europe for the general population [17,197] and specifically for HPs, such as the “COVID-19 related health literacy in health professionals” [43].

Regarding the delivery mode, most of the interventions were conducted through online distance learning, likely due to the circumstances and contact restrictions during the pandemic. Nevertheless, this demonstrates how quickly the implementation of interventions was adapted to rapidly changing external conditions.

Although the results indicate that COVID-19 HL interventions are beneficial for selected outcomes, individuals with high levels of self-reported and observable skills may still encounter real-world challenges in applying these competencies. This can occur, for example, in unfamiliar environments or when interacting with individuals they find intimidating, such as those in hierarchically higher-ranking positions [35]. Whether HPs are able to apply what they have learned over a longer period and translate it into actual behavior cannot be confirmed based on the available data. Consistent with similar studies [190,191], the outcomes were primarily measured immediately or shortly after the intervention was conducted. No long-term follow-ups, extending up to one year, were reported. This may be due to the urgency and relevance of rapidly providing interventional effects to the scientific community. As a result, it remains unclear to what extent COVID-19–related HL interventions have long-term sustainability.

### Limitations and Strengths

This review has several limitations. Substantial heterogeneity in interventions, comparators, settings, HP occupations, and outcomes limited the comparability between studies. Therefore, we were not able to state the effect of the interventions separated by delivery mode (eg, in-person and online distance learning), and delivery methods (eg, interactive and self-directed), intervention types, or specific occupational HP groups (eg, nurses, physicians, and medical students). Given the limited comparability across studies and substantial heterogeneity, the meta-analyses were conducted in an exploratory manner. Accordingly, the observed effects should be interpreted in light of the high risk of bias and low to very low certainty of evidence according to GRADE, and therefore interpreted with caution and regarded as preliminary evidence rather than definitive conclusions.

Moreover, for certain outcomes, a meta-analysis was not considered appropriate due to substantial differences in outcome metrics across studies. Consequently, the GRADE assessment could only be applied when meta-analytical results were available, and the domain of inconsistency could not be evaluated for these outcomes. The findings for these outcomes were reported narratively. We encountered challenges in contextualizing the results and the effect sizes within the existing literature, as there are currently only a few comparable reviews that report a meta-analysis based on HL interventions [190,191,198,199].

Further, most included studies did not explicitly aim to enhance the full construct of COVID-19–related HL. Instead, most studies assessed knowledge- and skills-based outcomes that were interpreted in this review as HL-related indicators. As a result, the review captures an evidence base that is closely related to HL, but not consistently grounded in explicit HL theory or measurement. In terms of the meta-analysis, it was not possible to determine publication bias due to the small number of studies included (≤10 studies). Additionally, a considerable proportion of effect sizes required conversion, and in certain cases, the assumptions underlying conversion could only be partly verified due to limited data availability in the primary studies.

A further limitation is the use of Microsoft Excel for data extraction. Although this program is commonly used for extracting data, it lacks the automated error-checking and audit functions of specialized systematic review software. To minimize potential error, data extraction was performed by two reviewers; one reviewer extracted relevant data, and the second reviewer checked the data extraction table for accuracy.

While these limitations should be considered, the present review also offers notable strengths. To our knowledge, this is the first study systematically synthesizing and summarizing evidence on interventions aiming to increase COVID-19–related HL in HPs. We ensured a comprehensive synthesis approach by searching 15 databases and registries, and including NRSIs and uncontrolled before-and-after studies in addition to RCT evidence, while closely adhering to guidelines for systematic reviews [42]. We followed the integrated model by Sørensen et al [20] as a framework for grouping and interpreting outcomes and intervention approaches. Further, we used validated assessment tools for rating risk of bias [47-49] and conducting synthesis in light of the certainty of the evidence by using GRADE [50]. The interpretation and reporting of the meta-analytic findings throughout this review were aligned with the corresponding GRADE assessments.

### Implications

Our findings imply a need for further measures to be taken. Future intervention studies should be based on established HL concepts as a theoretical framework for designing and implementing interventions to promote COVID-19–related HL as an essential key competency in HPs on the individual level. Existing models, such as the conceptualization of HL by Sørensen et al [20], the Medical Research Council framework [200], or the Optimizing HL and Access approach [201], offer valuable foundations for the systematic development and adaptation of HL interventions. Further, as Nutbeam and Lloyd [35] concluded, a consolidated standard curriculum for enhancing HL among HPs would be essential to adapt and tailor methods in response to crises such as the pandemic. A toolbox of established targeted educational and practice-based approaches for communicable diseases might guide researchers and clinicians in developing and implementing HL interventions rapidly in acute situations. For instance, the facet “understand” was frequently assessed through performance tests or self-reported questionnaires in order to capture participants’ COVID-19 knowledge. A more effective method for assessing whether participants have understood the content of a training program might be using the widely applied communication technique “teach-back,” where participants repeat in their own words what they have understood [35]. The “apply” facet may be better evaluated through observations conducted by a trained reviewer, using a predeveloped checklist (reported in 17 studies) compared to the often used self-assessments.

Moreover, the use of validated instruments available in various languages to evaluate changes in COVID-19–related HL is emphasized. Further, to monitor changes over time and capture long-term effects, future trials should incorporate repeated follow-up assessments after the intervention for at least 12 months after the intervention.

This review focused on the individual HL of HPs. The representation of professional HL in the context of the pandemic was not within the scope of this work. Beyond individual skills, clinicians with higher COVID-19–related HL, and thus a deeper understanding of COVID-19 and its related guidelines, may be better positioned to make informed decisions regarding patient management. This includes recognizing symptoms, knowing when to increase care, advising on appropriate treatments, answering patient questions effectively, and providing clear instructions on infection prevention and control measures. This is especially critical in environments in which misinformation and frequently changing guidelines can create uncertainty for both providers and patients [30,202]. Moreover, HPs are among others the primary sources of information for patients and the public [30,203]. Therefore, the level of COVID-19–related HL among HPs may also influence their surroundings, including the behaviors and attitudes of patients.

In this context, professional HL plays a crucial role, as it extends beyond individual competencies. Professional HL refers to the ability to communicate health information to patients in an understandable manner and to act on this information [204]. Pandemic-related HL interventions should not only focus on enhancing individual skills but also address professional HL, as HPs play a key role in promoting HL within the general population. Their transferable skills can significantly contribute to improving public HL [35,204].

HL-targeted interventions may empower individuals to enhance their competencies regarding infection control measures, enabling them to navigate the vast amount of pandemic-related information more effectively. This is particularly important as the pandemic transitions into an endemic phase, where ongoing community transmission necessitates sustained health education efforts. Given the ongoing presence of SARS-CoV-2 and the anticipated infection rates due to seasonal factors [205], we assume that the evidence landscape from COVID-19–related HL interventions continues to evolve. The increased number of records identified through database searches between the initial and updated searches (Figure 1) supports this assumption. Therefore, we aim to continuously identify and integrate new evidence and thus transform this systematic review into a living systematic review.

### Conclusions

A total of 15 studies investigated the effect of interventions relevant to COVID-19–related HL among HPs. However, the identified evidence base was heterogeneous in terms of intervention content, delivery, study populations, and outcomes, limiting the comparability and the synthesis of effects. Most included studies evaluated short-term educational or skills-based infection control interventions and operationalized HL indirectly through knowledge- and skills-related indicators rather than explicit, theory-based HL measures.

Overall, the identified interventions might support HPs’ short-term competencies relevant to infection prevention and control measures. Although some interventions showed large effects on specific outcomes, the evidence remains uncertain due to small sample sizes, high risk of bias, heterogeneity, imprecision, and limited theoretical grounding. In the absence of long-term follow-up data, the sustainability of observed effects remains unclear. The available evidence is closely related to HL, but not fully representative of explicitly HL-based interventions. Interventions explicitly designed to enhance the full construct of COVID-19–related HL remain largely absent. However, high-quality RCTs with adequate statistical power are needed to advance the current understanding of how interventions can strengthen HL in the context of communicable diseases, including COVID-19.

## Supplementary material

10.2196/70400 Multimedia Appendix 1 Search strategies.

10.2196/70400 Multimedia Appendix 2 Characteristics of studies.

10.2196/70400 Multimedia Appendix 3 Risk of bias assessments.

10.2196/70400 Multimedia Appendix 4 Summary of findings tables.

10.2196/70400 Multimedia Appendix 5 Narrative analyses of primary outcomes in uncontrolled before-and-after studies.

10.2196/70400 Checklist 1 PRISMA Checklist.

10.2196/70400 Checklist 2 PRISMA Checklist Abstract.

## Abbreviations

Cochrane RoB 2 Tool: Cochrane Risk of Bias 2 Tool
EPHPP-QAT: Quality Assessment Tool for Quantitative Studies
GRADE: Grading of Recommendations, Assessment, Development, and Evaluation
HL: health literacy
HP: health professional
MERS: Middle East respiratory syndrome
NRSI: nonrandomized study of interventions
PI: prediction interval
PICO: Population-Intervention-Comparator-Outcome
PPE: personal protective equipment
PRISMA: Preferred Reporting Items for Systematic Reviews and Meta-Analyses
RCT: randomized controlled trial
ROBINS-I: Risk of Bias in Nonrandomized Studies of Interventions
SMD: standardized mean difference
WHO: World Health Organization
