# Supplementary material for: Interventions to Enhance COVID-19 Pandemic Health Literacy in Health Professionals: Systematic Review
Source: JMIR Med Educ. 2026 Jul 10;12:e70400. doi: 10.2196/70400 (PMC13360183; doi:10.2196/70400)
Supplement: Multimedia Appendix 1 — Search strategies. [file mededu-v12-e70400-s001.pdf]

## Search Strategies for each Database

### Table S1. PubMed (MEDLINE®)

| | |
|---|---|
| **Population** | ***No restrictions*** |
| **Intervention** | (health[TIAB] AND (education*[TIAB] OR prevent*[TIAB] OR promot*[TIAB] OR intervention*[TIAB] OR program*[TIAB] OR training[TIAB] OR teach[TIAB] OR course[TIAB] OR measure[TIAB] OR strateg*[TIAB]))<br>AND |
| **Comparison** | n/a[a] |
| **Outcome** | (health literacy[MeSH] OR (“health literate”) OR (health[TIAB] AND (literacy[TIAB] OR competen*[TIAB]) OR knowledge[TIAB] OR skill*[TIAB] OR abilit*[TIAB] OR capabilit*[TIAB])<br>AND |
| **Context** | (2019-nCoV OR 2019nCoV OR COVID-19 OR SARS-CoV-2 OR ((wuhan AND coronavirus) AND 2019/12[PDAT]:2030[PDAT]) OR ("Severe Acute Respiratory Syndrome"[MeSH Terms]) OR (long-COVID OR long-haul COVID OR post-acute COVID syndrome OR persistent COVID-19 OR chronic COVID syndrome))<br>AND |
| **Study design[b]** | ((“Clinical Trial”[PT] OR “Comparative Study”[PT] OR “Evaluation study”[PT] OR “Cross-Over Studies”[MeSH] OR “Clinical Trials as Topic”[MeSH] OR random*[TIAB] OR control*[TIAB] OR “intervention study”[TIAB] OR “experimental study”[TIAB] OR trial[TIAB] OR trials[TIAB] OR evaluat*[TIAB] OR repeat*[TIAB] OR compar*[TIAB] OR versus[TIAB] OR “before and after”[TIAB] OR “interrupted time series”[TIAB]) NOT (“Animals”[MeSH] NOT (Animals[MeSH] AND “Humans”[MeSH]))) |

[a] Not recommended to be specified due to lack of sensitivity [1,2]. [b] Developed by Avau et al. [3].

### Table S2. Web of Science (Science citation index, social sciences citation index)

| | |
|---|---|
| **Population** | ***No restrictions*** |
| **Intervention** | (((ALL=((health AND (education* OR prevent* OR promot* OR intervention OR program* OR training OR teach OR course OR measure OR strateg*)))) AND |
| **Comparison** | n/a[a] |
| **Outcome** | ALL=((health literacy OR (“health literate”) OR (health AND (literacy OR competen*) OR knowledge OR skill* OR abilit* OR capabilit*)) )) AND |
| **Context** | ALL=((2019-nCoV OR 2019ncov OR COVID-19 OR SARS-CoV-2 OR (wuhan AND coronavirus) OR ("Severe Acute Respiratory Syndrome") OR (long-COVID OR long-haul COVID OR post-acute COVID syndrome OR persistent COVID-19 OR chronic COVID syndrome)))) |
| **Study design** | ALL=(((“Clinical Trial” OR “Comparative Study” OR “Evaluation study” OR “Cross-Over Studies” OR “Clinical Trials as Topic” OR random* OR control* OR “intervention study” OR “experimental study” OR trial* OR evaluat* OR repeat* OR compar* OR versus OR “before and after” OR “interrupted time series”) NOT (Animals AND “Humans”))) |

[a] Not recommended to be specified due to lack of sensitivity [1,2].

Table S3. EBSCO host (APA PsychInfo)

| | |
|---|---|
| **Population** | ***No restrictions*** |
| **Intervention & Outcome** | (Health AND (education* Or prevent* OR promot* OR intervention* OR program* OR training OR teach OR course OR measure OR strateg*)) ) AND ((Health AND (education* Or prevent* OR promot* OR intervention* OR program* OR training OR teach OR course OR measure OR strateg*))) AND |
| **Comparison** | n/a[a] |
| **Context** | ((2019-nCoV OR 2019nCoV OR COVID-19 OR SARS-CoV-2 OR ((wuhan AND coronavirus) ) OR ("Severe Acute Respiratory Syndrome"[MeSH Terms]) OR (long-COVID OR long-haul COVID OR post-acute COVID syndrome OR persistent COVID-19 OR chronic COVID syndrome))) AND |
| **Study design** | ("clinical trial" OR "comparative study" OR "evaluation study OR "cross-over studies" OR "clinical trials as topic" OR random* OR control* OR "intervention study" OR experimental study" OR trial OR trials OR evaluat* OR repeat* OR compar* OR versurs OR "before and after" OR "interrupted time series") NOT ("animals" NOT (animals AND "Humans")))) |

[a] Not recommended to be specified due to lack of sensitivity [1,2].

Table S4. Embase (über Ovid)

| | |
|---|---|
| **Population** | ***No restrictions*** |
| **Intervention** | (health AND (intervention OR program OR training OR teaching)) AND |
| **Comparison** | n/a[a] |
| **Outcome** | (health literacy OR (health AND (literacy OR competence) OR knowledge OR skill OR ability OR capability)) AND |
| **Context** | (2019-nCoV OR COVID-19 OR SARS-CoV-2 OR long-COVID) AND |
| **Study design[b]** | (clinical trial OR comparative study OR evaluation study OR intervention study OR trial*) |

[a] Not recommended to be specified due to lack of sensitivity [1,2]. [b] Developed by Avau et al. [3].

Table S5. Epistemonikos Database

| | |
|---|---|
| **Population** | ***No restrictions*** |
| **Intervention** | (health AND (intervention OR program OR training OR teaching)) AND |
| **Comparison** | n/a[a] |
| **Outcome** | (health literacy OR (health AND (literacy OR competence) OR knowledge OR skill OR ability OR capability)) AND |
| **Context** | (COVID-19 OR SARS-CoV-2) AND |
| **Study design[b]** | (clinical trial OR evaluation study OR intervention study)) |

[a] Not recommended to be specified due to lack of sensitivity [1,2]. [b] Developed by Avau et al. [3].

## Search Strategies for each Clinical Trial Register

Table S6. CENTRAL (CINHAL, ClinicalTrails.gov, WHO International Clinical trails registry Platform)

| | |
|---|---|
| **Population** | ***No restrictions*** |
| **Intervention** | ((health AND (education* OR promot* OR intervention* OR program* OR training OR teach OR course OR measure OR stretg*))):ti,ab,kw AND |
| **Comparison** | n/a[a] |
| **Outcome** | ((health literacy OR (“health literate”) OR (health AND (literacy OR competen*) OR knowledge OR skill* OR abilit* OR capabilit*))):ti,ab,kw AND |
| **Context** | ((SARS-CoV-2 OR ("Severe Acute Respiratory Syndrome") OR COVID-19 OR ("long-COVID" OR "long-haul COVID" OR "post-acute COVID syndrome" OR "chronic COVID syndrome"))):ti,ab,kw AND |
| **Study design** | ((Clinical Trial OR Comparative Study OR Evaluation study OR Cross-Over Studies OR intervention study OR experimental study OR trial*)):ti,ab,kw |

[a] Not recommended to be specified due to lack of sensitivity [1,2].

Table S7. Cochrane COVID-19 Study Register

| | |
|---|---|
| **Population** | ***No restrictions*** |
| **Comparison** | n/a[a] |
| **Outcome** | (health AND (literacy OR competen*) or knowledge or skill* or abilit* or capabilit*) |

[a] Not recommended to be specified due to lack of sensitivity [1,2].

Table S8. ISRCTN registry

| | |
|---|---|
| **Population** | ***No restrictions*** |
| **Intervention** | ((health AND (education* OR promot* OR intervention* OR program* OR training OR teach OR course OR measure OR stretg*)) AND |
| **Comparison** | n/a[a] |
| **Outcome** | (health literacy OR ("health literate") OR (health AND (literacy OR competen*) OR knowledge OR skill* OR abilit* OR capabilit*))) AND |
| **Context** | ((SARS-CoV-2 OR ("Severe Acute Respiratory Syndrome") OR COVID-19 OR ("long-COVID" OR "long-haul COVID" OR "post-acute COVID syndrome" OR "chronic COVID syndrome")) AND |
| **Study design** | (Clinical Trial OR Comparative Study OR Evaluation study OR Cross-Over Studies OR intervention study OR experimental study OR trial*)) |

[a] Not recommended to be specified due to lack of sensitivity [1,2].

Table S9. Australien New Zealand Clinical Trials Registry

| | |
|---|---|
| **Population** | ***No restrictions*** |
| **Intervention & Outcome** | ((Health AND competence) OR knowledge OR skill* OR abilit*) AND (COVID 19 OR "SARS-CoV2") |

Table S10. EU Clinical Trials Register

| | |
|---|---|
| **Population** | ***No restrictions*** |
| **Comparison** | n/a[a] |
| **Outcome** | (health literacy OR ("health literate") OR (health AND (literacy OR competen*) OR knowledge OR skill* OR abilit* OR capabilit*)) AND |
| **Context** | (SARS-CoV-2 OR ("Severe Acute Respiratory Syndrome") OR COVID-19) |

## References


1. Higgins JPT, Thomas J, Chandler J, Cumpston M, Li T, Page MJ, Welch VA, editor. Cochrane Handbook for Systematic Reviews of Interventions version 6.5 (updated August 2024); 2024.
2. Hausner E, Metzendorf M-I, Richter B, Lotz F, Waffenschmidt S. Study filters for non-randomized studies of interventions consistently lacked sensitivity upon external validation. BMC Med Res Methodol 2018;18(1):171. PMID:30563471
3. Avau B, van Remoortel H, Buck E de. Translation and validation of PubMed and Embase search filters for identification of systematic reviews, intervention studies, and observational studies in the field of first aid. J Med Libr Assoc 2021;109(4):599-608. PMID:34858089
