# Supplementary material for: Interventions to Enhance COVID-19 Pandemic Health Literacy in Health Professionals: Systematic Review
Source: JMIR Med Educ. 2026 Jul 10;12:e70400. doi: 10.2196/70400 (PMC13360183; doi:10.2196/70400)
Supplement: Multimedia Appendix 2 — Characteristics of studies. [file mededu-v12-e70400-s002.pdf]

Table S1. List of excluded studies (n=75)[a]

Study	Reason for exclusion
Akugue et al. 2022 [1]	Not eligible intervention and outcomes as the study focusses on psychological outcomes in the context of the COVID-19 pandemic.
Albott et al. 2020 [2]	Not eligible intervention and outcomes as the study focusses on psychological stress responses to the COVID-19 pandemic.
Ali et al. 2021 [3]	Not eligible outcome as the study focuses on vaccine promotion efforts aimed at reducing vaccine hesitancy, which is more associated with behaviour change interventional methods rather than enhancing COVID-19 related HL directly.
Aljahany et al. 2021 [4]	Not eligible outcome. Although the intervention aims at enhancing perceived knowledge of infection prevention measures (e. g. donning & doffing of PPE) among other outcomes, the overall performance score reported relates to a wide range of outcomes demonstrating the emergency department readiness in the context of the COVID-19 pandemic.
Alroumi et al. 2021 [5]	Not eligible outcome as the study addresses rather HP-related expertise on ICU in general without explicitly referring to COVID-19.
Amsalem et al. 2022 [6]	Not eligible intervention as the study aims at increasing treatment-seeking intentions among veterans experiencing mental health disorders during the COVID-19 pandemic, which does not refer to COVID-19 related HL.
Angeloni et al. 2024 [7]	Not eligible intervention and outcomes as the study focusses on self-reported adherence to standard precautions among nursing professionals, which does not refer to COVID-19 related HL.
Azadvari et al. 2021 [8]	Not eligible intervention and outcome. The study focusses on knowledge and attitude about the necessity and the manner of rehabilitation of COVID-19 patients in general (e. g. musculoskeletal, respiratory, gastrointestinal rehabilitation).
Babu et al. 2021 [9]	Not eligible outcome as the study addresses HP-expertise on preparedness and management of respiratory infection outbreaks during the COVID-19 pandemic particularly.
Barratt et al. 2020 [10]	The intervention was implemented prior to 2019, indicating that it is not directly related to the COVID-19 pandemic.
Bayram et al. 2023 [11]	Not eligible intervention as the study aimed at enhancing tracheostomy care knowledge, which is not COVID-19 related specifically.
Beneria et al. 2020 [12]	Not eligible intervention and outcomes as the study focusses on psychological outcomes in the context of the COVID-19 pandemic.
Berry et al. 2022 [13]	Not eligible intervention as the study aims at increasing COVID-19 vaccination rates, which is more associated with interventional behaviour change rather than enhancing COVID-19 related HL directly.
Blaak et al. 2021 [14]	Not eligible intervention. The intervention encompasses virtual table top simulation for primary care in general and did not refer to COVID-19 pandemic directly.
Boggs et al. 2021 [15]	Not eligible intervention as the study investigates knowledge, confidence, and attitudes of residents toward disaster medicine education in general.
Brown et al. 2021 [16]	Not eligible intervention. The aim of the intervention is to train interprofessional approaches without referring to COVID-19 pandemic.
Camilleri et al. 2022 [17]	Not eligible outcome as the study encompasses rather HP-related expertise on ICU, which is not COVID-19 related specifically.
Camputaro et al. 2021 [18]	Not eligible outcome as the study encompasses rather HP-related expertise on ICU, which is not COVID-19 related specifically.

Study	Reason for exclusion
Castro-Sanchez et al. 2021 [19]	Not eligible outcome. Although the intervention focus on enhancing infection prevention measures (e. g. donning & doffing of PPE) among other outcomes, the intervention is more associated with behaviour change interventional methods rather than enhancing COVID-19 related HL directly.
Cerqueira-Silva et al. 2021 [20]	Not eligible outcome. The outcomes are not related to COVID-19 HL directly; they focus on the transition from face-to-face training to digital training during the COVID-19 pandemic.
Choi et al. 2022 [21]	Not eligible intervention and outcome. The outcomes and intervention are not related to COVID-19 HL; they focus on the transition from face-to-face training to digital training during the COVID-19 pandemic.
Chompff 2021 [22]	Not eligible outcome. The outcomes are not related to COVID-19 HL directly; they focus on the temporal load completing the training.
Curtin et al. 2021 [23]	Not eligible intervention and outcomes as the study focusses on psychological outcomes in the context of the COVID-19 pandemic.
Cypro et al. 2021 [24]	Not eligible intervention and outcomes as the study focusses on psychological outcomes in the context of the COVID-19 pandemic.
Daniel et al. 2021 [25]	Not eligible outcome. The reported outcome encompasses rather HP-related expertise on palliative care, which is not directly COVID-19 HL related.
Davis et al. 2022 [26]	Not eligible outcome as the study aims at increasing vaccine uptake, which is more associated with behaviour change interventional methods rather than enhancing COVID-19 related HL directly.
Doyno et al. 2022 [27]	Not eligible outcome as the study focuses on reducing vaccine hesitancy, which is more associated with behaviour change interventional methods rather than enhancing COVID-19 related HL directly.
Elgzar et al. 2020 [28]	Not eligible intervention as it encompasses an experimental design.
Engberg et al. 2021 [29]	Not eligible outcome. The reported outcome encompasses rather HP-related expertise on palliative care, which is not directly COVID-19 HL related.
Fernandez et al. 2021 [30]	Not eligible intervention and outcome. Both are not related to COVID-19 HL; they focus instead on implementation of distance-based teaching modalities during the COVID-19 pandemic.
Gálvez-Herrer et al. 2021 [31]	Not eligible intervention and outcomes as the study focusses on psychological outcomes in the context of the COVID-19 pandemic.
Gautam et al. 2021[32]	Not eligible outcome as the study encompasses rather HP-related expertise on ICU, which is not COVID-19 related specifically.
Gu et al. 2020 [33]	Not eligible intervention as the study purposes to explore management strategy effects, which does not refer to COVID-19 related HL specifically.
Hassani et al. 2021 [34]	Not eligible intervention and outcome. The outcomes and intervention are not related to COVID-19 HL; they focus on testing virtual health care training in general.
Hazwani et al. 2021 [35]	Not eligible intervention and outcome. The outcomes and intervention are not related to COVID-19 HL; they focus on testing simulation-based programm for preparedness in the COVID-19 pandemic context.
Hubele 2022 [36]	Not eligible outcome. Focus lies on empowerment of patients.
Jackson et al. 2022 [37]	Not eligible outcome as the study encompasses rather general HP-related expertise on non-invasive ventilation, which is not COVID-19 related specifically.
Jain et al.	Not eligible intervention and outcome. The study focusses on programme development and report on evaluation of the feasibility of the intervention; The reported outcomes, such as leadership skills and capacities, are more reflective of general professional competencies rather than being specifically related to COVID-19 HL.

Study	Reason for exclusion
2023 [38]	
Jarden et al. 2022 [39]	Not eligible outcome. The outcome is not directly related to COVID-19 HL, focusing instead on an evaluation of the training program in terms of participant satisfaction and app utilization.
Joosten-Hagye et al. 2020 [40]	Not eligible intervention and outcomes as the study focusses on psychological outcomes in the context of the COVID-19 pandemic.
Kabi et al. 2021 [41]	Not eligible outcome. The outcome is not directly related to COVID-19 HL, focusing instead on airway management in general.
Kasai et al. 2022 [42]	Not eligible outcome. The reported outcomes are not directly related to COVID-19 HL, they focus mainly on perceived burden of handling information on and fear of COVID-19.
Kassa et al. 2025 [43]	Not eligible outcome. Data presented are limited to an generic IPC compliance assessment.
de Las Cuevas et al. 2022 [44]	Not eligible intervention and outcomes as the study focusses on therapeutically intervention with psychological outcomes in the context of the COVID-19 pandemic.
Latif et al. 2024 [45]	Not eligible outcomes. The study is limited to the evaluation of the intervention itself and did assess the participants' satisfaction primarily.
Lee et al. 2023 [46]	Not eligible outcome. The outcome emphasizes readiness, without being directly related to COVID-19 HL.
Li et al. 2020a [47]	Not eligible outcome. The outcome is not directly related to COVID-19 HL, focusing instead on willingness of getting vaccinated.
Li et al. 2020b [48]	Not eligible outcome. The outcome is not directly related to COVID-19 HL, focusing instead on willingness of getting vaccinated.
de Melo et al. 2022 [49]	Not eligible outcome. The outcome is not directly related to COVID-19 HL. Instead, the primary focus is on mental health and children's neurodevelopment during the COVID-19 pandemic
Misra et al. 2021 [50]	Not eligible outcome. Although the intervention aims to improve nasopharyngeal swab skills, the outcomes reported relate solely to perceived comfort in performing and attitudes.
Mulaudzi et al. 2025 [51]	Not eligible intervention and outcomes as the study focusses on investigating the availability of COVID-19 infection control policies and frontline nurses' adherence to these policies.
Nguyen et al. 2022 [52]	Not eligible intervention and outcomes as the study focusses on psychological outcomes in the context of the COVID-19 pandemic.
Ogbenna et al. 2022 [53]	Not eligible intervention and outcome. The study primarily focuses on palliative patient management and care, with the reported outcome on knowledge reflecting general expertise rather than knowledge specific to COVID-19.
Osula et al. 2022 [54]	Not eligible outcome. The outcome is not directly related to COVID-19 HL, focusing primarily on respiratory care in general.
Papadakis et al. 2023 [55]	Not eligible intervention and outcome. The study primarily focuses on enhancing skills for addressing COVID-19 vaccine hesitancy.
Pasi et al. 2023 [56]	Not eligible outcome and intervention. The study focuses mainly on child care in the context of the COVID-19 pandemic; the reported outcomes encompasses generic HP related skills and knowledge such as airway management, chest compression and utilization of oxygen delivery devices.
Pelin et al. 2024 [57]	Not eligible intervention and outcomes as the study focusses on assessing participants' comfort and confidence regarding the provided training.
Penna et al.	Not eligible outcome. The study is limited to the evaluation of the intervention itself and did not assess specific COVID-19 HL outcomes.

Study	Reason for exclusion
2022 [58]	
Rodriguez-Vega et al. 2020 [59]	Not eligible intervention and outcomes as the study focusses on psychological outcomes in the context of the COVID-19 pandemic.
Rondon et al. 2022 [60]	Not eligible outcomes. The study is limited to the evaluation of the intervention itself and did not assess specific COVID-19 HL outcomes instead it centers on participants' expectations and satisfaction, among other factors.
Suarez-Balcazar et al. 2025 [61]	Not eligible intervention and outcomes. The study is limited to the impact of training on generic HL without addressing COVID-19.
Seow et al. 2021 [62]	Not eligible outcome. The outcome is not directly related to COVID-19 HL, focusing primarily on quality improvement of blended distance learning in general.
Serrano-Ripoll et al. 2021 [63]	Not eligible intervention and outcomes as the study focusses on therapeutically intervention with psychological outcomes in the context of the COVID-19 pandemic.
Singh et al. 2023 [64]	Not eligible outcome as the study addresses knowledge retention of oxygen therapy particularly.
Stevens et al. 2023 [65]	Not eligible intervention. The study aims to enhance vaccine confidence, which does not fall under the scope of COVID-19 HL as defined in our underlying definition.
Takamatsu et al. 2021 [66]	Not eligible outcome as the study aims at increasing vaccine uptake, which is more associated with behaviour change interventional methods rather than enhancing COVID-19 related HL directly.
Teerasantipun et al. 2021 [67]	Not eligible intervention. The study aims to assess the knowledge of respiratory protection in a general context.
Thakur et al. 2021 [68]	Not eligible outcome. The outcome is not directly related to COVID-19 HL, focusing primarily on virtual education that support providers in caring of patients in general.
Trottier et al. 2022 [69]	Not eligible intervention and outcomes as the study focusses on psychological outcomes in the context of the COVID-19 pandemic.
Tsuchiya et al. 2021 [70]	Not eligible outcome. The outcome is not directly related to COVID-19 HL, focusing primarily on web-based educational programme in oncology in general.
Vilendrer et al. 2021 [71]	Not eligible outcomes. The study is limited to the evaluation of the intervention itself and did not assess specific COVID-19 HL outcomes instead it focused on appropriateness and acceptability of an application.
Weiner et al. 2020 [72]	Not eligible intervention and outcomes as the study focusses on therapeutically intervention with psychological outcomes in the context of the COVID-19 pandemic.
Xin et al. 2021 [73]	Not eligible outcome. The outcomes are not related to COVID-19 HL directly; they focus on the cognitive load training.
Yoo et al. 2024 [74]	Not eligible intervention and outcomes. The study is limited to the evaluation of the intervention itself and did not assess specific COVID-19 HL outcomes instead it focused on adoption of augmented reality simulation in educational programs.
Zhang et al. 2021 [75]	Not eligible outcome. The outcomes are not related to COVID-19 HL directly; they focus on online consultations.

[a]Based on Higgins et al. [76], the table only lists the excluded studies that did not clearly fulfil the criteria for exclusion.
HL Health Literacy, **HP** Health Professionals, **ICU** Intensive Care Unit, **IPC** Infection Prevention Control

References

1. Akugue P. Evaluating the Effectiveness of BREATHE for Nurse Practitioners During COVID Pandemic. The journal for nurse practitioners : JNP 2022. doi:10.1016/j.nurpra.2022.03.007
2. Albott CS, Wozniak JR, McGlinch BP, Wall MH, Gold BS, Vinogradov S. Battle Buddies: Rapid Deployment of a Psychological Resilience Intervention for Health Care Workers During the COVID-19 Pandemic. Anesthesia & Analgesia 2020;131(1):43-54. doi:10.1213/ANE.0000000000004912
3. Ali N, Ashiru-Oredope D, Murdan S. Training university students as vaccination champions to promote vaccination in their multiple identities and help address vaccine hesitancy. PHARMACY EDUCATION 2021;21(1):407-419. doi:10.46542/pe.2021.211.407419
4. Aljahany M, Alassaf W, Alibrahim AA, Kentab O, Alotaibi A, Alresseeni A, Algarni A, Algaeed HA, Aljaber MI, Alruwaili B, Aljohani K. Use of In Situ Simulation to Improve Emergency Department Readiness for the COVID-19 Pandemic. Prehospital and Disaster Medicine 2021;36(1):6-13. doi:10.1017/S1049023X2000134X
5. Alroumi F, Cota D, Chinea J, Ravikumar N, Tiru B, Pinto-Plata V, Tidswell M. Rapid Intensive Care Unit Onboarding in Response to a Pandemic. Journal Of Medical Education And Curricular Develoment 2021;8. doi:10.1177/23821205211020741
6. Amsalem Doron, Lazarov Amit, Markowitz John C, Smith Thomas E, Dixon Lisa B, Neria Yuval. Video intervention to increase treatment-seeking by healthcare workers during the COVID-19 pandemic: Randomised controlled trial. The British Journal of Psychiatry 2022;220(1):14-20. doi:10.1192/bjp.2021.54
7. Angeloni NLN, Furlan MCR, Da Barcelos LS, Menis Ferreira A, Lopes de Sousa AF, Valim MD, Andrade D de, Oliveira LB de, Batista OMA, Santos Junior AGd. Multimodal Educational Intervention for Adherence to Standard Precautions in the COVID-19 Pandemic Among Nursing Professionals: A Quasi-Experimental Study. J Nurs Midwifery Sci 2024;11(1). doi:10.5812/jnms-143516
8. Azadvari M, Razavi S Z.E, Hosseini M, Mayeli M. Evaluation of the impact of rehabilitation training on the knowledge and attitude of caregivers of covid-19 patients in Iran. Acta Medica Iranica 2021;59(17):587-595. doi:10.18502/acta.v59i10.7764
9. V Babu M, Arumugam MK, Debnath DJ. Simulated Patient Environment: A Training Tool for Healthcare Professionals in COVID-19 Era. Advances in Medical Education and Practice 2021;12:579-585. doi:10.2147/AMEP.S297536
10. Barratt Ruth, Wyer Mary, Suyin Hor, Gilbert Gwendolyn L. Medical interns' reflections on their training in use of personal protective equipment. Durham: Research Square 2020. BMC Medical Education.
11. Bayram SB, Çaliskan N, Gülnar E. The Effect of Web-Based Tracheostomy Care Game on Nursing Students? Knowledge Levels and Their Views of the Process. CLINICAL AND EXPERIMENTAL HEALTH SCIENCES 2023;13(1):41-47. doi:10.33808/clinexphealthsci.1021950
12. Beneria A, Arnedo M, Contreras S, Perez-Carrasco M, Garcia-Ruiz I, Rodriguez-Carballeira M, Radua J, Rius JB. Impact of simulation-based teamwork training on COVID-19 distress in healthcare professionals. BMC Medical Education 2020;20(1). doi:10.1186/s12909-020-02427-4
13. Berry SD, Goldfeld KS, McConeghy K, Gifford D, Davidson HE, Han L, Syme M, Gandhi A, Mitchell SL, Harrison J, Recker A, Johnson KS, Gravenstein S, Mor V. Evaluating the Findings of the IMPACT-C Randomized Clinical Trial to Improve COVID-19 Vaccine Coverage in Skilled Nursing Facilities. JAMA Internal Medicine 2022;182(3):324-331. doi:10.1001/jamainternmed.2021.8067
14. Blaak MJ, Fadaak R, Davies JM, Pinto N, Conly J, Leslie M. Virtual tabletop simulations for primary care pandemic preparedness and response. BMJ Simulation and Technology Enhanced Learning 2021;7(6):487-493. doi:10.1136/bmjstel-2020-000854
15. Boggs K, Goodwin T, Simpson J. Disaster Training following COVID-19 for Pediatric Medical Residents: Demand and Format. Disaster Medicine and Public Health Preparedness 2021:1-9. doi:10.1017/dmp.2021.209
16. Brown DK, Sanders M, Drost J, Hazelett S, Fosnight S, Kropp D, Patton R, Kidd L. Evaluation of a virtual simulation for geriatric team-based care. Journal of the American Geriatrics Society 2021;69(SUPPL 1):S120-S121. doi:10.1111/jgs.17115
17. Camilleri M, Zhang X, Norris M, Monkhouse A, Harvey A, Wiseman A, Sinha P, Hemsley A, Tang S, Menon A, Sinmayee S, Jones M, Buckley J, Johnson R, Medici T, Corner E. Covid-19 ICU remote-learning course (CIRLC): Rapid ICU remote training for frontline health professionals during the COVID-19 pandemic in the UK. Journal of the Intensive Care Society 2022;23(2):183-190. doi:10.1177/1751143720972630
18. Camputaro LA, Quezada MBE, Arango WHA, Bortoli RG, Zelaya FRA, Moreno MDM, Delgado JDM, Magana MMS, Cuellar PVA, Sanchez MI. Intensive competency-based training strategy in a National Hospital in times of Pandemic. MEDICINE 2021;100(39). doi:10.1097/MD.0000000000027152
19. Castro-Sanchez E, Alexander CM, Atchison C, Patel D, Leung W, Calamita ME, Garcia DM, Cimpeanu C, Mumbwatasai JM, Ramid D, Doherty K, Grewal HS, Otter JA, Wells EM. Evaluation of a personal protective equipment support programme for staff during the COVID-19 pandemic in London. Journal of Hospital Infection 2021;109:68-77. doi:10.1016/j.jhin.2020.12.004
20. Cerqueira-Silva T, Carreiro R, Nunes V, Passos L, Canedo BF, Andrade S, Ramos PIP, Khouri R, Santos CBS, Nascimento JDS, Paste AA, Paiva Filho IM, Santini-Oliveira M, Cruz Á, Barral-Netto M, Boaventura V. Bridging learning in medicine and citizenship during the COVID-19 pandemic: A telehealth-based case study. JMIR public health and surveillance 2021. doi:10.2196/24795
21. Choi J, Lee SE, Choi S, Kang B, Kim SH, Bae J, Tate JA, Son YJ. Integration of visual thinking strategies to undergraduate health assessment course: A mixed-method feasibility study. Nurse Education Today 2022;113:105374. doi:10.1016/j.nedt.2022.105374
22. Chompff Rebecca M. E. The Effects of Virtual Training Methods and Temporal Load on the Donning and Doffing Performance of Personal Protective Equipment: ProQuest Dissertations and Theses. Ann Arbor; 2021.
23. Curtin M, Downs J, Hunt A, Coleman ER, Enneking BA, McNally Keehn R. INteractive Virtual Expert-Led Skills Training: A Multi-Modal Curriculum for Medical Trainees. Front Psychiatry 2021;12:671442. doi:10.3389/fpsyt.2021.671442
24. Cypro A, McGuire WC, Rolfsen M, Jones N, Shah NG, Cribbs SK, Kaul V, Bojanowski CM, Pedraza I, Lynch L, Guzman L, Larsson E, Alexander LEC. An International Virtual COVID-19 Critical Care Training Forum for Healthcare Workers. ATS Scholar 2021;2(2):278-286. doi:10.34197/ats-scholar.2020-0154IN
25. Daniel S, Venkateswaran C, Chittazhathu RK, Rana S, Leng M. Efectiveness of a palliative care resource toolkit for COVID-19 for low and middle income countries (LMICS) on health care workers knowledge and confidence levels. BMJ supportive and palliative care 2021;11:A19-. doi:10.1136/spcare-2021-PCC.49
26. Davis Colin J, Golding Matt, McKay Ryan. Efficacy information influences intention to take COVID‐19 vaccine. British Journal of Health Psychology 2022;27(2):300-319. doi:10.1111/bjhp.12546

27. Doyno CR, Fitzgerald JM, White CM, Sobieraj DM, Zacchera M. Innovative partnership in Connecticut to expand health professional eligibility to administer COVID-19 vaccines. J Am Pharm Assoc (2003) 2022;62(1):247-252. doi:10.1016/j.japh.2021.08.013
28. Elgzar WT, Al-Qahtani AM, Elfeki NK, Ibrahim HA. COVID-19 Outbreak: effect of an Educational Intervention Based on Health Belief Model on Nursing Students' Awareness and Health Beliefs at Najran University, Kingdom of Saudi Arabia. Afr J Reprod Health 2020;24(s1):78-86. doi:10.29063/ajrh2020/v24i2s.12
29. Engberg M, Bonde J, Sigurdsson ST, Moller K, Nayahangan LJ, Berntsen M, Eschen CT, Haase N, Bache S, Konge L, Russell L. Training non-intensivist doctors to work with COVID-19 patients in intensive care units. ACTA ANAESTHESIOLOGICA SCANDINAVICA 2021;65(5):664-673. doi:10.1111/aas.13789
30. Fernandez CSP, Green MA, Noble CC, Brandert K, Donnald K, Walker MR, Henry E, Rosenberg A, Dave G, Corbie-Smith G. Training "Pivots" from the Pandemic: Lessons Learned Transitioning from In-Person to Virtual Synchronous Training in the Clinical Scholars Leadership Program. JOURNAL OF HEALTHCARE LEADERSHIP 2021;13:63-75. doi:10.2147/JHL.S282881
31. Gálvez‐Herrer Macarena, Via‐Clavero Gemma, Ángel‐Sesmero José Antonio, Heras‐La Calle Gabriel. Psychological crisis and emergency intervention for frontline critical care workers during the covid‐19 pandemic. Journal of Clinical Nursing 2021. doi:10.1111/jocn.16050
32. Gautam S, Shukla A, Mishra N, Kohli M, Singh GP. Effectiveness of virtual training for medical officers and community health officers in the critical care management of COVID-19 patients in the intensive care unit. Indian journal of anaesthesia 2021;65(Suppl 4):S168-S173. doi:10.4103/ija.ija_704_21
33. Gu S, Zhang A, Huo G, Yuan W, Li Y, Han J, Shen N. Application of PDCA Cycle Management for Postgraduate Medical Students During the COVID-19 Pandemic. ResearchSquare 2020. doi:10.21203/rs.3.rs-105412/v1
34. Hassani K, McElroy T, Coop M, Pellegrin J, Wu WL, Janke RD, Johnson LK. Rapid Implementation and Evaluation of Virtual Health Training in a Subspecialty Hospital in British Columbia, in Response to the COVID-19 Pandemic. Frontiers in pediatrics 2021;9:638070. doi:10.3389/fped.2021.638070
35. Hazwani TR, Al Hassan Z, Al Zahrani A, Al Badawi A. A Simulation-Based Program for Preparedness for COVID-19 at a Pediatric Tertiary Hospital in Saudi Arabia. Cureus 2021;13(2):e13131. doi:10.7759/cureus.13131
36. Hubele Jaron Allen. Goals-of-care conversations and COVID-19: Using a brief educational module to empower generalist clinicians at the southern AZ VA health care system: Dissertation Abstracts International: Section B: The Sciences and Engineering; 2022.
37. Jackson P, Siddharthan T, Cordoba Torres IT, Green BA, Policard CJ, Degraff J, Padalkar R, Logothetis KB, Gold JA, Fort AC. Developing and Implementing Noninvasive Ventilator Training in Haiti during the COVID-19 Pandemic. ATS Scholar 2022;3(1):112-124. doi:10.34197/ats-scholar.2021-0070OC
38. Jain S, Dempsey K, Wilcox S, Bradd P, Travaglia J, Debono D, Justin L, Hor SY. Practice development amidst a pandemic: a pilot programme to develop leadership in infection prevention and control professionals. Leadership in health services (Bradford, England) 2023. doi:10.1108/LHS-06-2023-0037
39. Jarden R, Scanlon A, Bridge N, Mckeever S, Turner R, Prescott H, Thompson J, Cambridge P, Kinney S, Leong N, Gerdtz M. Coronavirus disease 2019 Critical Care Essentials course for nurses: development and implementation of an education program for healthcare professionals. Australien Journal Of Advanced Nursing 2022;39(1):34-43. doi:10.37464/2020.391.423
40. Joosten-Hagye Dawn, Katz Anne, Sivers-Teixeira Theresa, Yonshiro-Cho Jeanine. Age-friendly student senior connection: Students' experience in an interprofessional pilot program to combat loneliness and isolation among older adults during the COVID-19 pandemic. J Interprof Care;34(5):668-671. doi:10.1080/13561820.2020.1822308
41. Kabi A, Dhar M, Arora P, Bhardwaj BB, Chowdhury N, Rao S. Effectiveness of a Simulation-Based Training Program in Improving the Preparedness of Health Care Workers Involved in the Airway Management of COVID-19 Patients. Cureus 2021;13(8):e17323. doi:10.7759/cureus.17323
42. Kasai H, Saito G, Ito S, Kuriyama A, Kawame C, Shikino K, Takeda K, Yahaba M, Taniguchi T, Igari H, Sakao S, Suzuki T. COVID-19 infection control education for medical students undergoing clinical clerkship: a mixed-method approach. BMC Medical Education 2022;22(1). doi:10.1186/s12909-022-03525-1
43. Kassa G, Ogongo I, Rabkin M, Bancroft E, Mitchell R, Block L, Dennison C, Katwesigye E, Paulos M, Hokororo J, Kamau I, Herzig C. The East Africa Infection Prevention and Control (IPC) Learning Network: An Approach to Improving IPC Competencies and Practices During the COVID-19 Pandemic, 2020-2023. Clin Infect Dis 2025;81(1):41-48. PMID:40581363
44. de Las Cuevas C, Gutierrez-Rojas L, Alvarez-Mon MA, Andreu-Bernabeu A, Capitan L, Gomez JC, Grande I, Hidalgo-Mazzei D, Mateos R, Moreno-Gea P, De Vicente-Munoz T, Ferre F. Evaluating the Effect of a Telepsychiatry Educational Program on the Awareness, Knowledge, Attitude, and Skills of Telepsychiatry Among Spanish Psychiatrists during COVID-19 Pandemic. Telemedicine journal and e-health : the official journal of the American Telemedicine Association 2022. doi:10.1089/tmj.2022.0051
45. Latif A, Zaki M, Shahbaz H, Hussain SA, Daudpota AA, Imtiaz B, Asghar F, Hassan MM, Asghar MA, Aqeel M, Khan MF, Khan R, Mahmood F, Nawab S, Sabeen A, Sohaib M, Sultan SF, Tariq M, Thawer H, Ali N, Jawwad M, Niazi K, Noorali AA, Amin SK, Atiq H, Samad Z, Haider A. Mass online training of health care workers during COVID-19: Approach, impact, and outcomes for over 10,000 health care providers. Public Health 2024;233(null):193-200 URL: https://research.ebsco.com/linkprocessor/plink?id=4f5634a5-1256-3212-a6cc-1f5922520363.
46. Lee EH, Rashid A, Lawal I, Adekanye U, Adamu Y, Godfrey C, Agaba PA, Okeji N, Desai P. Protecting healthcare workers and patients during the COVID-19 pandemic: a comparison of baseline and follow-up infection prevention and control needs in Nigerian military healthcare facilities delivering HIV services. BMC Health Services Research 2023;23(1):1254. doi:10.1186/s12913-023-10289-x
47. Li J, Tang Z, Gong Z. Does the message frame promote people's willingness to vaccinate when they worry about side effects from the covid-19 vaccine? Evidence from an online survey experiment in china. Health Communication 2022. doi:10.1080/10410236.2022.2028469
48. Li J, Gong Z, Tang Z, Zhou J. How message frames promote people's willingness to get vaccinated? The mediation role of perceived net benefits. Int J Public Health 2023;67. doi:10.3389/ijph.2022.1605232
49. Melo MD de, Ferri PM, Vasconcelos MM de, Reis ZS, Godoy SC, Palmeira VA, Rocha G, Torres RM, Tupinambás U. COVID-19: Distance learning to empower educators and health assistants in rural areas. Journal of education and health promotion 2022;11(1). doi:10.4103/jehp.jehp_1303_21
50. Misra A, Carlson KJ, Barnes CA, Pate SK, Stobbe BB, Dowdall JR. A Novel Just-In-Time-Online-Training for Nasopharyngeal Swab Specimen Collection During the COVID-19 Pandemic. Cureus 2021;13(6):e15944. doi:10.7759/cureus.15944

51. Mulaudzi F, Downing C. Frontline nurses' adherence to COVID-19 policies in care delivery at a Johannesburg Academic Hospital. Curationis 2025;48(1):e1-e9. PMID:40459089
52. Nguyen B, Torres A, Sim W, Kenny D, Campbell DM, Beavers L, Lou W, Kapralos B, Peter E, Dubrowski A, Krishnan S, Bhat V. Digital Interventions to Reduce Distress Among Health Care Providers at the Frontline: Protocol for a Feasibility Trial. JMIR research protocols 2022;11(2). doi:10.2196/32240
53. Ogbenna A, Drane D, Crowe AN, Oyedele O, Hauser J, Soyannwo O, Ogunseitan A, Doobay-Persaud A. Building the Nigerian Palliative Care Workforce: An Interdisciplinary Distance Learning Training Program. Annals of global health 2022;88(1):96. doi:10.5334/aogh.3744
54. Osula VO, Sanders JE, Chakare T, Mapota-Masoabi L, Ranyali-Otubanjo M, Hansoti B, McCollum ED. COVID-19 advanced respiratory care educational training programme for healthcare workers in Lesotho: an observational study. BMJ open 2022;12(4):e058643. doi:10.1136/bmjopen-2021-058643
55. Papadakis S, Anastasaki M, Gamaletsou M, Papagiannopoulou X, Aligizakis E, Lionis C. Development of an eLearning intervention for enhancing health professionals' skills for addressing COVID-19 vaccine hesitancy. Front Med (Lausanne) 2023;10:1290288. PMID:38155659
56. Pasi R, Babu TA, Kalidoss VK. Development and validation of structured training module for healthcare workers involved in managing pediatric patients during COVID-19 pandemic using "Objective Structured Clinical Examination" (OSCE). Journal of education and health promotion 2023;12:15. doi:10.4103/jehp.jehp_578_22
57. Pelin C, Vasser M, Cavuoto Petrizzo M, Cassara M, McLeod-Sordjan R, Weiner J, Ginzburg S. Mitigating Misinformation Toolkit: A Medical Student Role-Play Curriculum on Communication Techniques to Facilitate Vaccine Misinformation Conversations. MedEdPORTAL 2024;20:11439. PMID:39193179
58. Penna AR, Hunter JC, Sanchez GV, Mohelsky R, Barnes LEA, Benowitz I, Crist MB, Dozier TR, Elbadawi LI, Glowicz JB, Jones H, Keaton AA, Ogundimu A, Perkins KM, Perz JF, Powell KM, Cochran RL, Stone ND, White KA, Weil LM. Evaluation of a Virtual Training to Enhance Public Health Capacity for COVID-19 Infection Prevention and Control in Nursing Homes. Journal of public health management and practice : JPHMP 2022;28(6):682-692. doi:10.1097/PHH.0000000000001600
59. Rodriguez-Vega B, Palao A, Munoz-Sanjose A, Torrijos M, Aguirre P, Fernandez A, Amador B, Rocamora C, Blanco L, Marti-Esquitino J, Ortiz-Villalobos A, Alonso-Sanudo M, Cebolla S, Curto J, Villanueva R, de-la-Iglesia M J, Carracedo D, Casado C, Vidal E, Trigo D, Iglesias N, Cabanas D, Mellado L, Garcia D, Fernandez-Encinas C, Navarro R, Bravo-Ortiz M F, Bayon C, Mediavilla R, Vidal-Villegas M P. Implementation of a Mindfulness-Based Crisis Intervention for Frontline Healthcare Workers During the COVID-19 Outbreak in a Public General Hospital in Madrid, Spain. Front Psychiatry 2020;11:562578. doi:10.3389/FPSYT.2020.562578
60. Rondon, J., Benkhoucha, C., Cornec, N., Fleur, L., Lhours, G., Marchais, S & Sylvain, M. [The role of a health promotion prevention and education resources center in times of health crisis]. Santé Publique 2022(6):1023-1032. doi:10.3917/spub.216.1023
61. Suarez-Balcazar Y, Allen-Meares P, Dickens C, Brazil E, Garcia-Bedoya O, Biggers A. Enhancing the Education of Community Health Workers on Health Literacy and Cultural Humility in Times of COVID-19. AMERICAN JOURNAL OF HEALTH EDUCATION 2025;56(3):256-265. doi:10.1080/19325037.2024.2366453
62. Seow YT, Teo SC, Yap W, Foo Z, Tan KH. Interactive videoconferencing in the redesign of a health-care quality improvement workshop for the coronavirus disease 2019 pandemic. PROCEEDINGS OF SINGAPORE HEALTHCARE 2021;30(3):177-184. doi:10.1177/2010105820961795
63. Serrano-Ripoll MJ, Ricci-Cabello I, Jimenez R, Zamanillo-Campos R, Yanez-Juan AM, Bennasar-Veny M, Sitges C, Gervilla E, Leiva A, Garcia-Campayo J, Garcia-Buades ME, Garcia-Toro M, Pastor-Moreno G, Ruiz-Perez I, Alonso-Coello P, Llobera J, Fiol-deRoque MA. Effect of a mobile-based intervention on mental health in frontline healthcare workers against COVID-19: Protocol for a randomized controlled trial. Journal of Advanced Nursing 2021;77(6):2898-2907. doi:10.1111/jan.14813
64. Singh D, Salhotra R, Singh A, Bajaj M, Saxena AK, Sharma SK, Yadav P. Retention of Knowledge and Efficacy of a Hands-on Training Session in Oxygen Therapy for COVID-19 among Healthcare Workers. Indian Journal of Critical Care Medicine 2023;27(2):127-131. doi:10.5005/jp-journals-10071-24327
65. Stevens G, Johnson LC, Saunders CH, Schmidt P, Sierpe A, Thomeer RP, Little NR, Cantrell M, Yen RW, Pogue JA, Holahan T, Schubbe DC, Forcino RC, Fillbrook B, Sheppard R, Wooten C, Goldmann D, O'Malley AJ, Dubé E, Durand MA, Elwyn G. The CONFIDENT study protocol: a randomized controlled trial comparing two methods to increase long-term care worker confidence in the COVID-19 vaccines. BMC Public Health 2023;23(1):384. doi:10.1186/s12889-023-15266-x
66. Takamatsu Akane, Honda Hitoshi, Kojima Tomoya, Murata Kengo, Babcock Hilary M. Promoting coronavirus disease 2019 (COVID-19) vaccination among healthcare personnel: A multifaceted intervention at a tertiary-care center in Japan. Cambridge: Cambridge University Press; 2021. Infection Control & Hospital Epidemiology.
67. Teerasantipun C, Pichetweerachai W, Pruetpongpun N, Suwannawat K, Chaiwong W, Kunanusont C. The Effect of Training on Knowledge, Perception, and Practice of Healthcare Personnel on the Use of Respiratory Protective Equipment during COVID-19 Pandemic at a Private Hospital in the Northern part of Thailand. bkkmedj 2021;17(02):110-119. doi:10.31524/bkkmedj.2021.21.004
68. Thakur A, Pereira C, Hardy J, Bobbette N, Sockalingam S, Lunsky Y. Virtual Education Program to Support Providers Caring for People With Intellectual and Developmental Disabilities During the COVID-19 Pandemic: Rapid Development and Evaluation Study. JMIR mental health 2021;8(10):e28933. doi:10.2196/28933
69. Trottier K, Monson CM, Kaysen D, Wagner AC, Liebman RE, Abbey SE. Initial findings on RESTORE for healthcare workers: an internet-delivered intervention for COVID-19-related mental health symptoms. Translational psychiatry 2022;12(1):222. doi:10.1038/s41398-022-01965-3
70. Tsuchiya M, Terazono H, Maki Y, Yoshikawa N, Kawahara Y, Nishimura K, Shinohara K, Ogawa D, Mori R, Iwamoto Y, Itagaki F, Masuko H, Yonemura M, Uchida M. Evaluation of a web-based educational programme for pharmacists during the COVID-19 pandemic in Japan. J Clin Pharm Ther 2021;46(6):1743-1749. doi:10.1111/jcpt.13526
71. Vilendrer S, Amano A, Brown Johnson CG, Favet M, Safaeinili N, Villasenor J, Shaw JG, Hertelendy AJ, Asch SM, Mahoney M. An App-Based Intervention to Support First Responders and Essential Workers During the COVID-19 Pandemic: Needs Assessment and Mixed Methods Implementation Study. Journal of medical Internet research 2021;23(5):e26573. doi:10.2196/26573
72. Weiner Luisa, Berna Fabrice, Nourry Nathalie, Severac François, Vidailhet Pierre, Mengin Amaury C. Efficacy of an online cognitive behavioral therapy program developed for healthcare workers during the COVID-19 pandemic: The REduction of STress (REST) study protocol for a randomized controlled trial. Durham: Research Square 2020. Trials.
73. Xin R, Li L, Qiaoli S, Xingyue W. Real Workload-Situated Training in COVID-19 Prevention of General Practice Residents in China: A Situated Cognition Study. Frontiers in public health 2021;9:765402. doi:10.3389/fpubh.2021.765402

74. Yoo S, Heo S, Song S, Park A, Cho H, Kim Y, Cha WC, Kim K, Son MH. Adoption of Augmented Reality in Educational Programs for Nurses in Intensive Care Units of Tertiary Academic Hospitals: Mixed Methods Study. JMIR serious games 2024;12:e54188. doi:10.2196/54188
75. Zhang D, Liao H, Jia Y, Yang W, He P, Wang D, Chen Y, Zhang YP. Effect of virtual reality simulation training on the response capability of public health emergency reserve nurses in China: a quasiexperimental study. BMJ open 2021;11(9):e048611. doi:10.1136/bmjopen-2021-048611
76. Higgins JPT, Thomas J, Chandler J, Cumpston M, Li T, Page MJ, Welch VA, editor. Cochrane Handbook for Systematic Reviews of Interventions version 6.5 (updated August 2024); 2024.

Table S2. Characteristics of included Studies

Study (Author, Year) Country	Period of time of data collection	Population	Interventions & comparators	Outcomes				Sponsorship Source	Conflicts of Interest
		Participants description, sample size (total), setting	Intervention & Control Delivery mode	Outcome	Relation to health literacy (HL)	Outcome measure	Timing of outcome measurement		
**Study Design: Randomised Controlled Trials**									
**Alotaibi et al. 2021** [1] **Kuwait**	-	302 students, who finished the first year of study from the disciplines of medicine, pharmacy, physical therapy, occupational therapy, communication disorders, nutrition, medical laboratory sciences, radiological sciences and health informatics and information management<br>Age mean (SD): 20.9y, (2.1), Range 18-35, Gender: 96.4% female, 3.6% male	**Intervention:** Online intervention programs (not specified)<br>IG1: via text (brochure)<br>IG2: via Instagram (written posts)<br>IG3: synchronous online interactive educational workshop<br>**Control:** Placebo intervention	**Primary:**<br>COVID-19 specific vaccine knowledge	**HL indicator:**<br>Knowledge<br>**HL component:**<br>Understand	5-items Likert subscale "Knowledge and beliefs about coronavirus Vaccination" of the COVID-19 Healthy Lifestyle Promotion Scale (self-developed and validated within the present study, participant-reported)	F1:<br>Two weeks after intervention	Kuwait Foundation for the Advancement of Sciences under project code: PN20-13NO-02-	None declared
**Amiri et al. 2023** [2] **Iran**	Mar 2021 - Jun 2021	90 Iranian health care providers from community health centers of Gonabad city in eastern Iran<br>Age mean (SD): 30.63y (7.79)	**Intervention:** Educational programs regarding COVID-19 management (instructions and protocols sent by the Ministry of Health to universities)<br>IG1: via multimedia (files consisting of audio, video, animation and written content), Duration: 5 files á 10 min<br>IG2: via booklet (PDF format)<br>**Control:** Educational content in a routine format provided by the centers and departments	**Primary:**<br>(a) COVID-19 specific knowledge on providing services in women during pregnancy, childbirth, and breastfeeding<br>(b) COVID-19 related performance skills in providing services in women during pregnancy, childbirth, and breastfeeding<br>**Secondary:**<br>(c) COVID-19 related attitudes towards providing services in women during pregnancy, childbirth, and breastfeeding	**HL indicators:**<br>(a) Knowledge<br>(b) Performance skills<br>**HL component:**<br>(a) Understand<br>(b) Apply<br>**HL-related factor:**<br>(c) Attitude	Questionnaires developed within the present study and previous studies; participant-reported<br>(a) 22-items Likert scale; response options: 'yes', 'no', 'I don't know'; total score range 0-44<br>(b) 22-items Likert scale; response options: 'yes', 'no'; total score range 0-22<br>(c) 22-items Likert scale; response options 'I agree', 'Disagree' and 'Have no opinion'; total score range 0-44<br>Content validity conducted with 10 experts and specialists; Content validity ratio: 0.67; Content validity index: 0.84<br>Reliability Cronbach α=0.79	F1:<br>Directly after intervention<br>F2:<br>Four weeks after intervention	Clinical Research Development Unit, Bohlool Hospital, Gonabad University of Medical Sciences, Gonabad, Iran	None declared

Study (Author, Year) Country	Period of time of data collection	Population	Interventions & comparators	Outcomes				Sponsorship Source	Conflicts of Interest
		Participants description, sample size (total), setting	Intervention & Control Delivery mode	Outcome	Relation to health literacy (HL)	Outcome measure	Timing of outcome measurement		
**Study Design: Randomised Controlled Trials**									
**Birrenbach et al. 2021** [3]<br>**Switzerland**	Sept 2020 - Dec 2020	29 medical students (years 3-6/6-year curriculum) at the emergency department of the Inselspital, University Hospital Bern<br>Age median: 23y, Range 22-25<br>Gender:<br>60% female, 40% male (IG);<br>64% female, 36% male (CG)	**Intervention:** Virtual reality (VR) educational training (the Covid-19 VR Strikes Back (CVRSB) module, version 1.1.6, ORamaVR SA, Oculus Rift S (Facebook Inc))<br>**Control:** Traditional learning methods (printed instructions, local instruction videos on COVID-19-related skills, formal videos on proper hand hygiene and on taking a nasopharyngeal sample)	**Primary:**<br>(a) COVID-19 related infection protection performance skills:<br>(i) Performance of Hand disinfection<br>(ii) Performance of nasopharyngeal swab<br>(iii) Performance of personal protective equipment (PPE)<br>**Adverse events:**<br>(b) "Visually induced motion sickness"	**HL indicator:**<br>Performance skills<br>**HL component:**<br>(a) Apply	(a) (i) Evaluated using a fluorescent marker<br>(ii) + (iii) 17-items checklist for evaluating the performance swab & PPE use<br>(assessed through observation of independent and blinded raters)<br>(b) Simulator Sickness Questionnaire (SSQ); participant-reported; adapted from Kennedy et al. [4]	F1:<br>Directly after intervention<br>F2:<br>One month after intervention	Funded in part by a grant from the Swiss National Science foundation (grant no. 31CA301966 15) for the project "Mixed-method evaluation of an online forward triage tool within the COVID-19 pandemic" to TCS and WEH	WEH, UK, MDI, AO, TCS, GP declared a conflict of interest. The other authors report no conflict of interest.
**Christensen et al. 2020** [5]<br>**Denmark**	-	21 medical students (years 3-6) and junior doctors at the Faculty of Health and Medical Sciences, University of Copenhagen, Hvidovre Hospital and Slagelse Hospital	**Intervention:** Informational videos on PPE use<br>**Control:** In-person 2-3 hour training session with a live demonstration of donning and doffing of PPE with subsequent training in groups of 1-4 people	**Primary:**<br>COVID-19 related infection protection performance skills (donning & doffing)	**HL indicator:**<br>Performance skills<br>**HL component:**<br>Apply	Adapted "2014 Donning and Doffing PPE Competency Validation Checklist" by the Association for Professionals in Infection Control and Epidemiology; clinician reported through observation	F1:<br>One month after intervention	-	None declared
**Currat et al. 2022** [6]<br>**Switzerland**	-	64 first-year students paramedics from the Colleges of Higher Education in Ambulance Care (Bern, Geneva)<br>Age median:<br>24y, Range 22-26 (IG1),<br>27y, Range 23-28 (IG2)<br>Gender:<br>58% female, 42% male (IG1);<br>61% female, 39% male (IG2)	**Intervention:** Blended learning on PPE doffing proficiency consisting of interactive gamified e-learning module (based on the SERES framework) and face-to-face learning (workshop, based on Peyton's 4-step approach)<br>**Control:** Interactive gamified e-learning module	**Primary:**<br>(a) COVID-19 related infection prevention knowledge acquisition on PPE<br>(b) COVID-19 related infection protection performance skill retention<br>(i) Correct doffing sequences performance (observer- & participant-rated)	**HL indicators:**<br>(a) Knowledge<br>(b. c) Performance skills<br>**HL component:**<br>(a) Understand<br>(b) Apply<br>**HL-related factor:**<br>(c) Confidence (self-efficacy)	(a & c) Questionnaire (participant-reported)<br>(b) (i) Independent researcher rated recorded videos with a checklist at the second session and participants electronically rebuild the doffing sequence<br>(remotely through an online platform)	F1:<br>Immediately after the intervention<br>F2:<br>4-8 weeks after intervention	Grant from the Hans Wilsdorf Foundation. Material costs (overalls, N95 respirator masks) were paid by the Colleges of Higher Education in Ambulance Care	Three Authors (LSt, LSu, and BG) receive financial compensation when serving as external teaching professional and/or exam expert for the Colleges of Higher Education in Ambulance Care in Geneva and Bern. One author (MM) is employed by the Center for

Study (Author, Year) Country	Period of time of data collection	Population	Interventions & comparators	Outcomes				Sponsorship Source	Conflicts of Interest
		Participants description, sample size (total), setting	Intervention & Control Delivery mode	Outcome	Relation to health literacy (HL)	Outcome measure	Timing of outcome measurement		
**Study Design: Randomised Controlled Trials**									
				(ii) Correct hand disinfection performance (observer-rated)<br>**Secondary:**<br>(c) Perceived confidence in the ability of using PPE					Medical Education in Bern as a teacher. The funders had no role during this study process.
**Jafree et al. 2022 [7]**<br>**Pakistan and Germany**	-	344 nurses who were employed in public or private hospitals of Lahore, Pakistan, during the COVID-19 pandemic	**Intervention:** WhatsApp-Group learning intervention over three months and literacy booklet (sent through both email and WhatsApp)<br>**Control:** Only literacy booklet (in English)<br>Duration: 12 weeks	**Secondary:**<br>*Composite outcome*<br>(a) Confidence thinking regarding infection prevention control<br>(b) COVID-19 related knowledge (not further specified)	**HL indicator:**<br>(a, b) Knowledge<br>**HL-related factor:**<br>(a) Confidence (self-efficacy)<br>**HL component:**<br>(b) Understand	Online questionnaire: 5-point Likert scale; participant-reported; with eight items; through Google Survey Form)	F1:<br>Three months after baseline	Shahid Hussain Foundation, Shalamar Hospital Finance Office	None declared
**Jeihooni et al. 2023 [8]**<br>**Iran**		250 Health medical personnel working in health treatment centers in Abadan city<br>Age mean (SD):<br>39y, 6 (IG)<br>38y, 5 (CG)<br>Gender:<br>56% female, 44% male (IG)<br>48 % female, 52% male (CG)	Educational intervention based on health-belief model<br>**Intervention:** Lectures, questions and answers, group discussion, images, and video clips (group based, blended learning)<br>**Control:** similar intervention six months later<br>Duration: Three sessions (two 50-minute sessions and one 90-minute session) and two follow-up sessions (1 month and 2 months after educational intervention)	**Primary:**<br>(a) General COVID-19 related knowledge<br>**Secondary:**<br>(b) Perceived COVID-19 related self-efficacy<br>(c) Prevalence of infection prevention behaviour	**HL indicator:**<br>(a) Knowledge<br>**HL component:**<br>(a) Understand<br>**HL-related factor:**<br>(b) Self-efficacy<br>**HL-related outcome:**<br>(c) Prevention behaviour	Research-made questionnaire containing demographic characteristics, the HBM constructs, and preventive behaviors (participant-reported)<br>(a & b)<br>5-point Likert scale<br>(c) 11 questions, nominal scale ('yes', 'no');<br>Face validity & Content validity was conducted.<br>Reliability<br>Cronbach α=0.85	F1:<br>Three months after intervention<br>(One month after two follow-up sessions)	None reported	None declared
**Li et al. 2020 [9]**<br>**China**	-	48 physicians and nurses active in the clinical front line of the Department of Anesthesiology<br>Gender: 56% female, 44% male	**Intervention:** Training in PPE donning and doffing through informational video (10-minute video, twice) & live demonstration (10 min, twice), checklist containing PPE donning and doffing steps	**Primary:**<br>(a) COVID-19 related infection protection performance skills on PPE<br>**Secondary:**	**HL indicator:**<br>(a) Performance skills<br>**HL component:**<br>(a) Apply	(a) Evaluated through observation by two trained physicians with a 29 steps/items checklist; assessed through observer	F1:<br>Post-intervention<br>(not specified)	The National Natural Science Foundation of China (No. 81673922, 81503663,81704167) and	None declared

Study (Author, Year) Country	Period of time of data collection	Population	Interventions & comparators	Outcomes				Sponsorship Source	Conflicts of Interest
		Participants description, sample size (total), setting	Intervention & Control Delivery mode	Outcome	Relation to health literacy (HL)	Outcome measure	Timing of outcome measurement		
**Study Design: Randomised Controlled Trials**									
			**Control:** Informational video (10-minute video for four times) & individual learning, checklist containing PPE donning and doffing steps	(b) Perceived confidence in the use of PPE (Donning & Doffing)	**HL-related factor:** (b) Confidence (self-efficacy)	(2) One item, 5-point Likert scale (1 'very difficult' to 5 'very easy'); participant-reported		the Project of Educational Commission of Guangdong Province of China (No.2018KTSCX037)	
**Manggala et al. 2022 [10] Indonesia**	-	40 medical doctors and nurses at the high Care Unit Cipto Mangunkusumo Hospital and Simulation-Based Medical Education and Research Center (SIMUBEAR), IMERI Universitas<br>Age median:<br>27.5y, Range 23-41 (IG1)<br>30y, Range 24-46 (CG)<br>Gender:<br>80% female, 20% male (IG1);<br>65% female, 35% male (CG)	**Intervention:** In situ high fidelity simulation-based training provided as an online interactive lecture session, two in situ simulation sessions and a debriefing at the end of each simulation session<br>**Control:** Low fidelity simulator<br>Duration: 3 days	**Primary:**<br>(a) COVID-19 related infection protection performance skills on PPE (Doffing)<br>(b) COVID-19 related infection protection performance skills on PPE (Donning)	**HL indicator:**<br>(a, b) Performance skills<br>**HL component:**<br>(a, b) Apply	Tools were developed from predefined hospital transport protocols and checklists; Each item was rated '0' for undone skill, '2' for a incompletely done skill and '5' for the skill that had been done completely.	F1: Directly after intervention (three days after baseline)	Universitas Indonesia Research Fund	None declared
**Rakhshani et al. 2024 [11] Iran**	2021	164 health care personnel, working in health healthcare services from health and treatment centers of Dehdasht City, Iran	**Intervention:**<br>Self-learned virtual learning package including various educational material (e.g., audio files, text messages, visual aids, video messages, PowePoint presentations)<br>**Control:**<br>Delayed educational program after intervention	**Secondary**<br>Attitudes towards COVID-19 infection prevention behaviors	**HL-related factor:**<br>Attitudes	Researcher-made questionnaire;<br>8-item 5-point Likert scale, ranging from 'complete agreement' to 'complete disagreement'; score range 8-40 points	F1: Three months after intervention	None	None declared
**Rueda-Medina et al. 2022 [12] Spain**	Nov 2021-Mar 2022	142 health science students from 1st, 2nd, and 3rd academic year taken from the Nursing and Physiotherapy, Degrees, of the Faculty of Health Sciences of Granada and Melilla<br>Age mean (SD): 22.54y (6.39)<br>IG: 23.44y (7.49)<br>CG: 21.61y (4.91)<br>Gender: 63.4% female, 36.6% male<br>IG: 54.4% female	**Intervention:**<br>(i) Face-to-face teaching with active training PPE<br>(ii) PPE use with active training<br>Duration: each training 60 min<br>**Control:** Non-face-to-face teaching with passive training (watching videos and PPE use protocols)<br>Afterwards for both groups: simulation scenario based on the management of a COVID-19 patient	**Primary**<br>(a) COVID-19 related performance skills on PPE (Donning & Doffing)<br>**Secondary:**<br>(b) Perceived confidence in using personal protective equipment (Donning & Doffing)	**HL indicator:**<br>(a) Performance skills<br>**HL component:**<br>(a) Apply<br>**HL-related factors:**<br>(b) Confidence (self-efficacy)	(a) Checklist - time of the procedure and total number of donning and doffing tasks was calculated; assessed by an instructor<br>(b) Questionnaire: self-assessment; respectively (5-point Likert Scale ranging from 'totally disagree' to 'totally	F1: Directly after intervention	University of Granada	None declared

Study (Author, Year) Country	Period of time of data collection	Population: Participants description, sample size (total), setting	Interventions & comparators: Intervention & Control Delivery mode	Outcomes: Outcome	Outcomes: Relation to health literacy (HL)	Outcomes: Outcome measure	Outcomes: Timing of outcome measurement	Sponsorship Source	Conflicts of Interest
**Study Design: Randomised Controlled Trials**									
		CG: 45.6% female	In both teaching and training modalities, the protocols and resources used were based on those developed by the Centers for Disease Control and Prevention (CDC) and the World Health Organization (WHO) 2020<br>Duration: 90 min (15 min simulation & 75 min debriefing)			agree'); participant-reported; used by Salway et al. [13]			
**Suppan et al. 2020a [14]**<br>**Switzerland**	April 2020	291 emergency prehospital personnel (emergency medical technicians, paramedics, emergency physicians) working in Geneva<br>Age median (Q1-Q3):<br>34 (28-40) (IG)<br>35 (30-42) (CG)<br>Gender:<br>28% female (IG)<br>32% female (CG)	**Intervention:** Blended learning intervention providing prehospital COVID-19 guideline & gamified e-learning module (created under Storyline 3 (Articulate Global)<br>**Control:** Prehospital COVID-19 guideline only (developed from the Geneva University Hospitals, version 1.11)<br>Duration: 12 days	**Primary:**<br>(a) COVID-19 related infection prevention knowledge on PPE<br>**Secondary:**<br>(b) Perceived confidence in the ability of using PPE	**HL indicator:**<br>(a) Knowledge<br>**HL component:**<br>(a) Understand<br>**HL-related factors:**<br>(b) Confidence (self-efficacy)	(a) 10-question (closed-ended) pre- & post intervention quizzes (either multiple choice or multiple answer); participant-reported<br>(b) 5-point Likert scale; participant-reported<br>(developed within the present study)	F1:<br>Post-intervention<br>(not specified)	None	None declared
**Suppan et al. 2020b [15]**<br>**Switzerland**	Enrolment date unclear; until May 2020	138 student paramedics from French-speaking paramedic schools<br>Age median (Q1-Q3):<br>26 (24-30) (IG)<br>25 (23-30) (CG)<br>Gender:<br>39% female (IG)<br>49% female (CG)	**Intervention:** Abridged version of the prehospital COVID-19 guideline & e-learning module (created under Storyline 3 (Articulate Global) on PPE knowledge<br>**Control:** Abridged version of the prehospital COVID-19 guideline only (developed from the Geneva University Hospitals, created under Visio 2013 (Microsoft Corporation)<br>Duration of completion of the module: 15 min	**Primary:**<br>(a) COVID-19 related infection prevention knowledge on PPE<br>(b) COVID-19 related infection protection performance skills of adequacy donning sequences<br>(c) COVID-19 related infection protection performance skills of adequacy doffing sequences<br>**Secondary:**<br>(d) Perceived confidence in the ability of using PPE	**HL indicator:**<br>(a) Knowledge<br>(b,c) Performance skills<br>**HL component:**<br>(a) Understand<br>(b-c) Apply<br>**HL-related factor:**<br>(d) Confidence (self-efficacy)	(a) 22-item questionnaire provided in French & English (six question were related to the correct choice of PPE); participant-reported<br>(b) Scenario-based questions with multiple choice and multiple answer options by putting elements of PPE donning in the correct order; participant-reported<br>(c) Scenario-based questions with multiple choice and multiple answer options by putting	F1:<br>Immediately after intervention	None	None declared

Study (Author, Year) Country	Period of time of data collection	Population	Interventions & comparators	Outcomes				Sponsorship Source	Conflicts of Interest
		Participants description, sample size (total), setting	Intervention & Control Delivery mode	Outcome	Relation to health literacy (HL)	Outcome measure	Timing of outcome measurement		
**Study Design: Randomised Controlled Trials**									
						elements of PPE doffing in the correct order; participant-reported<br>(d) 5-point-Likert scale; participant-reported			
**Wang et al. 2022 [16]**<br>**China**	March 2020	38 residents who receive standardized training in the obstetrics and gynecology base of Shengjing Hospital of China Medical University<br>Age mean (SD):<br>25.11y (1.12) (IG)<br>24.63y (1.48) (CG)<br>Gender (N)<br>18 females, 2 males (IG)<br>17 females, 1 male (CG)	**Intervention:** Conceive-design-implement-operate (CDIO) professional training model<br>Delivery mode: online<br>Delivery methods: Blended methods (e. g. case-based teaching, simulation training und role-playing)<br>**Control:** Traditional training based on protection theory exposition with step-by-step demonstration of the basic use of PPE and independent practice of the participants	**Primary**<br>(a) COVID-19 related performance skills on PPE<br>(b) COVID-19 related knowledge on infection prevention control measures	**HL indicator:**<br>(a) Performance skills<br>(b) Knowledge<br>**HL component:**<br>(a) Apply<br>(b) Understand	(a) Evaluation standard of the PPE protective operation as the scoring basis provided by the hospital with a maximum of 100 points (no further information)<br>(b) COVID-19 prevention and control papers with a maximum of 100 points (80 points for single-choice questions and 20 points for four short-answer questions)	F1:<br>Directly after intervention	Internal funding from Shengjing Hospital, China Medical University (SJ-M0133), and 345 Talent Project of Shengjing Hospital of China Medical University (No. M0946)	None declared
**Xie et al. 2021 [17]**<br>**China**<br>**(Preprint)**	-	72 medical residents ranging from the first to the third year in training in the department of anesthesiology in the Sichuan provincial hospital	**Intervention:** Remote skill training on donning and doffing through a multimodal teaching approach (training videos, live demonstration. standard procedure as written text and rubrics for assessing skills) with videofeedback (via WeChat group)<br>**Control:** Individual learning without video feedback	**Primary:**<br>(a) COVID-19 related infection prevention knowledge on PPE (donning & doffing)<br>(b) COVID-19 related infection protection performance skills of PPE (donning & doffing)	**HL indicator:**<br>(a) Knowledge<br>(b) Performance skills<br>**HL component:**<br>(a) Understand<br>(b) Apply	(a) Online theory test (18 Multiple-Choice-Questions + 2 fill-in-the-blank questions); participant-reported<br>(b) In-person skill assessment (not further specified); assessed through instructors	F1:<br>Two weeks after learning phase	None	None declared

Study (Author, Year) Country	Period of time of data collection	Population	Interventions & comparators	Outcomes				Sponsorship Source	Conflicts of Interes
		Participants description, sample size (total), setting	Intervention & Control Delivery mode	Outcome	Relation to health literacy (HL)	Oucome measure	Timing of outcome measurement		
	Study Design: Non-Randomised Studies of Interventions								
**Ansari et al. 2023 [18]** **Pakistan**	Jul 1st - Dec 31st, 2020	60 Health professionals (doctors, nurses, medical assistants) working in the Department of Gynaecology and Obstetrics<br>Age mean (SD):<br>33.12y (4.13) (IG)<br>35.35y (5.67) (CG)<br>Gender:<br>43.3% female, 56.6% male (IG)<br>70% female, 30% male (CG)	**Intervention:** Instructor-led training sessions with PPE demonstration followed by observation of the participants with feedback. Between weekly sessions participants have been self-practicing daily 30 minutes.<br>Duration - direct: 8h/indirect 6.9h<br>**Control:** 30-minute training videos including PPE demonstration<br>Duration: 10hr/indirect: 6.7h	**Primary:** COVID-19 related performance skills on PPE (donning, doffing)	**HL indicator:**<br>Performance skills<br>**HL component:**<br>Apply	Validated PPE competency checklist (PPE, donning, doffing, Standard Precautions & Transmission based Precautions) & recorded time spent of training<br>Donning and doffing score against the checklists was calculated; assessed through instructor<br>Maximum score:<br>8 for donning, 14 for doffing	F1:<br>One month after the intervention	None declared	None declared
**Buyego et al. 2022 [19]** **Uganda**	-	52 nurses, clinical officers, laboratory officers, medical officers, public health officers, pharmacists, Epidemiologists<br>Age median: 34.6y, Range 25-60<br>Gender: 56% female, 44% male	**Intervention:** VR-based simulations of Infection prevention and control (IPC) training, with HTC Vive Pro head-mounted display (HMD). Content was provided through (1) Enduvo (Enduvo, Inc. Peoria, IL, US), (2) Humulo (Humulo, Inc., Edge- water, MD, US) and (3) SOMA.<br>**Control:**<br>(1) Classroom instruction in a similar cohort from health workers<br>(2) No training<br>Duration: Two weeks	**Secondary:**<br>*Composite outcome* COVID-19 related knowledge (acquisition & retention)	**HL indicator:**<br>Knowledge<br>**HL component:**<br>Understand	Likert scale questionnaire with 8 items using the updated Ministry of Health COVID-19 IPC classroom/in-person course as the guide	F1:<br>Directly after the intervention (two weeks after baseline assessment)	Government of Uganda (Makerere University Research and Innovation Fund (RIF)); Supplementary support for equipment and structural support was provided by the NIH/NIAID/Office of Cyber Infrastructure and Computational Biology (OCICB), the BRECA program under NIH/FIC (1U2RTW010672–01) and the Infectious Diseases Institute in Uganda.	None declared

Study (Author, Year) Country	Period of time of data collection	Population	Interventions & comparators	Outcomes				Sponsorship Source	Conflicts of Interes
		Participants description, sample size (total), setting	Intervention & Control Delivery mode	Outcome	Relation to health literacy (HL)	Oucome measure	Timing of outcome measurement		
	**Study Design: Non-Randomised Studies of Interventions**								
**Hu et al. 2020 [20]** **China**	March - May 2020	129 medical students<br>Age mean (SD):<br>22.4y (0.9) (lecture group)<br>22.37y (0.689) (game group)<br>Gender:<br>57% female, 43% male (lecture group); 57% female, 43% male (game group)	**Intervention:** Game based intervention<br>**Control:** Online lecture<br>Duration: 3 hrs	**Primary:**<br>General COVID-19 related knowledge	**HL indicator:**<br>Knowledge<br>**HL component:**<br>Understand	Questionnaire regarding COVID-19 diagnostic criteria, PPE knowledge and patient condition evaluation (10 multiple-choice questions, participant-reported)	F1:<br>Post-intervention (not specified)<br>F2:<br>Five weeks after intervention	Novel Coronavirus Research Fund of West China Hospital and the Strategic Priority Research Program of the Chinese Academy of Science	None declared
**Yu et al. 2022 [21]** **South Korea**	Nov13th - Dec10th 2021	50 third- and fourth-year nursing students enrolled in a nursing college in "J" City in Gyeongsangnam province<br>Age mean (SD): 22.7y (2.1)<br>Gender: 90% female	**Intervention:** Virtual reality infection control simulation for children with COVID-19 (including in person pre- and debriefing with an instructor)<br>**Control:** No intervention	**Primary:**<br>(a) COVID-19 specific infection prevention knowledge on PPE performance<br>(b) Infection control measures performance<br>**Secondary:**<br>(c) Self-efficacy in infection control	**HL indicator:**<br>(a) Knowledge<br>(b) Performance skills<br>**HL component:**<br>(a) Understand<br>(b) Apply<br>**HL-related factor:**<br>Self-efficacy	Questionnaires<br>(a) 20 items, score-based; participant-reported; developed by on Choi et al. [22] (master thesis); Content validated<br>(b) 20 items, 5-point Likert scale, score-based; participant-reported; developed by Kwon et al. [23] (master-thesis) Reliability Cronbach α=0.97<br>(c) 10 items, 7-point Likert scale; participant-reported; developed by Ayres et al. [24] (dissertation)<br>Reliability Cronbach α=0.94	F1:<br>Post-intervention (not specified)<br>IG: directly after intervention<br>CG: Three weeks after intervention	National Research Foundation of Korea funded by the Ministry of Education, Science, and Technology (NRF-2018R1D1A3 B07045408)	None declared

Study (Author, Year) Country	Period of time of data collection	Population	Intervention	Outcomes				Sponsor-ship Source	Conflicts of Interest
		Participants description, Sample size (total), Setting		Outcome	Relation to health literacy (HL)	Outcome measure	Timing of outcome measurement		
**Study Design: Uncontrolled Before-After Studies**									
**Abbas et al. 2020 [25]** **Pakistan**	Jan 2020 - Apr 2020	500 health-care workers (doctors, nurses, technicians, staff members) in different cities in Pakistan; Recent medical graduates<br>Age mean (SD): 23.6y (5.3y)<br>Gender: 67 % female, 33% male	E-health education<br>Distance delivery mode: Web-based modul/online course	**Secondary:**<br>Prevalence of infection prevention behaviour	**HL-related outcome:**<br>(c) Prevention behaviour	Self-administered questionnaire regarding compliance with hand and respiratory hygiene (participant-reported)	F1:<br>One to two weeks after intervention	None reported	None declared
**Ahmed et al. 2022 [26]** **Egypt**	Dec 2020 - May 2021	159 maternity nurses working at the Women's Health Hospital, Assiut University, Egypt (Emergency ward: 50 nurses, Intensive care unit: 34 nurses, Inpatient wards: 43 nurses, Operation ward: 16 nurses, Outpatient clinics: 16 nurses)<br>Age mean (SD): 29.3y (6.7)<br>Age (%):<br>< 25y: 43.4<br>25-40y: 38.4<br>> 40y: 18.2	Educational program<br>Duration: 19 weeks, 40–60 min/session, two sessions daily	**Primary:**<br>(a) COVID-19 related knowledge on infection prevention control measures<br>(b) COVID-19 related infection protection performance skills<br>**Secondary:**<br>(c) Attitudes towards COVID-19 infection prevention measures	**HL indicator:**<br>(a) Knowledge<br>(b) Performance skills<br>**HL component:**<br>(a) Understand<br>(b) Apply<br>**HL-related factor:**<br>(c) Attitude	Self-developed questionnaire<br>(a) 20 questions; score ranging from 1 ('correct answer') to 0 ('incorrect', 'Don't know'); total score range 0-20; participant-reported; total knowledge considered efficient if ≥ 70% (14 score) and inefficient if <70% (14 score); participant-reported; items adapted from Mohammed (2021)[27]<br>(b) 23 checklist questions; scoring: 1 ('correct action done'), 2 ('action sometimes done'), 3 ('incorrect action'); total score range 0-46; total practice considered satisfactory if ≥ 70% (32 score) and unsatisfactory if <70% (32 score); participant-reported; items adapted from Nwagbara et al. (2021)[28]<br>(c) 8 questions; score range: 3 ('agree'), 2 ('unsure') and 1 ('not agree'); total score range 1-24; total attitude positive if ≥ 70%	F1:<br>Four weeks after intervention	None reported	None reported

Study (Author, Year) Country	Period of time of data collection	Population: Participants description, Sample size (total), Setting	Intervention	Outcomes: Outcome	Outcomes: Relation to health literacy (HL)	Outcomes: Outcome measure	Outcomes: Timing of outcome measurement	Sponsor-ship Source	Conflicts of Interest
**Study Design: Uncontrolled Before-After Studies**									
						(17 score) and negative if <70% (17 score); participant-reported; items adapted from Mohammed (2021)[27]<br>Content validity was conducted by panel of 3 experts<br>Reliability Cronbach α=8.809E-7			
**Altillo et al. 2021** [29]<br>**United States**		29 medical students in second, third or fourth year at the University of Texas<br>Gender: 65.5% female, 34.5% male	Medical student elective combined student-directed, faculty-supported online learning (curriculum evaluation)<br>Distance delivery mode: Via online platform (using Canvas, Instructure, Inc., Salt Lake City, UT; and Zoom®) with asynchronous modules combined field placements at health system or community partner site<br>Duration: Two weeks<br>Modules lasted 10-15h/week; Field placement lasted 25-30h/week	**Secondary**:<br>*Composite outcomes*<br>(a) General COVID-19 and public health related knowledge<br>(b) Perceived general COVID-19 and public health related knowledge	**HL indicator:**<br>(a, b) Knowledge (composite)<br>**HL component:**<br>(a, b) Understand (composite)	Questionnaire:<br>(a) Multiple choice (6 items refer to general COVID-19 related knowledge; 5 items refer to general public health related knowledge) (participant-reported)<br>(b) 4-point Likert scale (participant-reported)	F1:<br>Directly after intervention	No funding	None declared
**Aqel et al. 2023** [30]<br>**United States**	-	279 pharmacists employed at a nationwide Health Technology Company in the US, who participated in a company-sponsored continuing education session	Educational session based on continuing education (CE) presentation	**Primary:**<br>COVID-19 specific vaccine knowledge	**HL indicator:**<br>Knowledge<br>**HL component:**<br>Understand	10 items on the content of the CE presentation<br>Multiple choice (five response options), 3 items only relate to the primary outcome); self-developed questionnaire by the research team (validated, participant-reported)<br>Reliability Cronbach α = 0.65	F1:<br>Directly after intervention	None declared	External funding from various agencies and organizations outside this study is noted.

Study (Author, Year) Country	Period of time of data collection	Population	Intervention	Outcomes				Sponsor-ship Source	Conflicts of Interest
		Participants description, Sample size (total), Setting		Outcome	Relation to health literacy (HL)	Outcome measure	Timing of outcome measurement		
**Study Design: Uncontrolled Before-After Studies**									
**Aujee et al. 2022** [31] **India**	-	60 health professionals staff nurses working in labour room of selected hospitals of PCMC<br>Age:<br>26-30y: 31.7%<br>31-35y: 48.3%<br>≥ 35y: 20%	Educational intervention based on WHO COVID-19 protocols	**Primary:**<br>COVID-19 related konwledge on (WHO) protocols	**HL indicator:**<br>Knowledge<br>**HL component:**<br>Understand	Structured questionnaire (10 questions with options), Range: 0-10; poor 0-3, average 4-6, good 7-9, excellent 10, (validated by 16 experts, participant-reported)<br>Reliability: Pearson Correlation Coefficient (r) = 0.89)	F1:<br>Directly after intervention	None declared	None declared
**Bakhsh et al. 2023** [32] **Saudia-Arabia**	Completed Jun - Jul 2020	113 hospital trainees and physicians from King Abdulaziz University Hospital<br>(Out of the 113 participants enrolled in the course, only 65 consented to have their data analyzed for the study)<br>Gender: 46.2% female, 53.8% male	Educational intervention/training program in two parts:<br>(1) Virtual-based (pre-recorded webinars and lectures; developed by the Health Academy of the Saudi Comission for Health Specialities, SCFHS)<br>Duration: average 10 hrs<br>(2) Practice-based (hands-on course conducted at the King Abdulaziz University-Clinical Skills and Simulation Center)<br>Four stations:<br>i) Basic Airway Skills<br>ii) Simulation-Based Scenario<br>iii) PPE<br>iv) Mechanical Ventilation<br>Duration: 1 day (1-day focused course lasting 5-hrs), each station 45 min	**Primary:**<br>(a) COVID-19 specific infection prevention knowledge on PPE<br>**Secondary:**<br>(b) *Composite outcome*<br>COVID-19 specific infection prevention knowledge on PPE and general patient airway management<br>(c) Perceived confidence in COVID-19 critical care	**HL indicator:**<br>(a. b) Knowledge<br>**HL component:**<br>(a, b) Understand<br>**HL-related factor:**<br>(c) Confidence (self-efficacy)	(a) Questionnaire regarding knowledge (1 out of 20 multiple-choice questions referred to the primary outcome, with four options, developed by a group of active clinical faculty, participant-reported)<br>(b) Competency checklist developed by SCFHS (through observation)<br>(c) One question; self-reported confidence levels (scale 0-10)	F1:<br>Directly after intervention	Not applicable	None declared
**Bayomi et al. 2021** [33] **Egypt**	Oct - Dec 2020	286 first-year students at the Faculty of Nursing at Zagazig University<br>Age:<br>17y: 2.1%<br>18y: 69.2%<br>19y: 28.7%	1st phase:<br>Educational intervention with developed teaching guidelines (lectures with PowerPoint, brochures, images), followed by an open conversation	**Primary:**<br>(a) General COVID-19 related knowledge<br>(b) COVID-19 related infection prevention performance<br>**Secondary**:	**HL indicator:**<br>(a) Knowledge<br>(b) Performance skills<br>**HL component:**<br>(a) Understand<br>(b) Apply	(a) Interview form with 27 closed ended questions (true/false); total score 100%; Adapted from Ministry of Health guidelines 2020 [34]<br>(participant-reported)	F1:<br>Directly after the first and the second phase of Intervention	None declared	None declared

Study (Author, Year) Country	Period of time of data collection	Population: Participants description, Sample size (total), Setting	Intervention	Outcomes: Outcome	Relation to health literacy (HL)	Outcome measure	Timing of outcome measurement	Sponsor-ship Source	Conflicts of Interest
**Study Design: Uncontrolled Before-After Studies**									
		Gender: 49.7% female, 50.3% male	Duration for each session: 180 min (120 min theory & 60 min open conversation)<br>Duration: 2x/week for 3 months<br><br>2nd phase:<br>Educational intervention with demonstrations (e. g. personal hygiene, preventive control procedures); presented with PowerPoint, videos, demonstration and re-demonstration<br>Duration for each session: 240 min<br>Duration: 1x/week for 3 month	(c) Attitudes towards COVID-19 infection prevention measures	**HL-related factor:**<br>(c) Attitude	(b) 1st part: Interview form; personal hygiene and preventive measures (11 items) (partcipant-reported)<br>2nd part: observational checklist regarding technique of using mask (12 Items), and hand-washing (15 Items); Adopted from WHO, 2020 [35] (through observer)<br>(c) Interview form with 18 closed ended questions (2-point Likert Scale of agreement); Adopted from Nassef et al. 2020 [36] (participant-reported)<br>Designed tools and booklet were tested for content validity; developed by a panel of even medical surgical nursing experts<br>Reliability Cronbach α = 0.988, 0.985, and 0.977 for each one consecutively			
**Bechini et al. 2023** [37]<br>**Italy**	Jan - Feb 2022	387 students from the Medicine and Surgery degree courses, and from the pharmaceutical area<br>Gender: 64.9% female, 35.1% male	Educational intervention 'Elective Teaching Activity' (through online platform Moodle) included lessons on vaccines<br>Duration: 8 hrs	**Primary:**<br>COVID-19 related vaccine knowledge	**HL indicator:**<br>Knowledge<br>**HL component:**<br>Understand	Questionnaire (12 multiple-choice questions with questions 10 through 12 varying between medicine and pharmaceutical degrees)	F1:<br>Directly after intervention	None reported	None declared

Study (Author, Year) Country	Period of time of data collection	Population	Intervention	Outcomes				Sponsorship Source	Conflicts of Interest
		Participants description, Sample size (total), Setting		Outcome	Relation to health literacy (HL)	Outcome measure	Timing of outcome measurement		
**Study Design: Uncontrolled Before-After Studies**									
**Bieri et al. 2023** [38] **Switzerland**	Dec 13th 2021 - Jan 25th 2022	108 second-year medical students from the Faculty of Medicine in the University of Geneva, Switzerland	Educational and practical lecture with two different sessions:<br>(1) E-learning and video-based self-directed learning<br>Duration: 60 min<br>(2) In-person practice session in groups (4-6 students) using simulators (nasopharyngeal swab collection and intramuscular injection)<br>Duration: 120 min	**Secondary:**<br>*Composite outcome*<br>Perceived knowledge & confidence in performing nasopharyngeal swab	**HL component:**<br>Understand (composite)<br>**HL-related factor:**<br>Confidence (self-efficacy, composite)	3-Item questionnaire; 5-point Likert Scale; participant-reported; developed and validated by faculty members	F1:<br>One day after intervention	None reported	None declared
**Blake et al. 2022** [39] **United Kingdom**	July - Sept 2020	162 health and social care professionals, trainees or public health specialists working at public and private hospitals, public health or clinical commissioning groups or local government networks	COVID-19 Vaccine Education (digital training package)<br>Duration: eight weeks	**Primary:**<br>(a) COVID-19 related vaccine knowledge<br>**Secondary**:<br>(b) Perceived confidence in the ability to communicate COVID-19 vaccine related importance	**HL indicator:**<br>(a) Knowledge<br>**HL component:**<br>(a) Understand<br>**HL-related factor:**<br>(b) Confidence (self-efficacy)	Pre (2 items) and post (14 items) survey, which adapted from the 'Evaluation Toolkit for Reusable Learning Objects and deployment of e-Learning Resources' [40]; participant-reported	F1:<br>Within the first four weeks	The University of Nottingham Health e-Learning and Media Team supported the technological development and hosting of the reusable leaning object (RLO). The sponsors had no involvement in the study. The views expressed are those of the authors and not necessarily those of the funders.	None declared
**Boccalini et al. 2022** [41] **Italy**	Feb - Apr 2021	449 medical, hygiene, and pharmaceutical students from years 1 to 4 at the University of Florence<br>Gender: 67% female, 33% male	Elective Teaching Activity (through online platform Webex) included lessons on vaccines and vaccinations<br>Duration: 16hrs	**Primary:**<br>COVID-19 specific vaccine knowledge	**HL indicator:**<br>Knowledge<br>**HL component:**<br>Understand	Questionnaire (30 multiple-choice questions regarding the main topics of the teaching activity; 3 out of 30 questions relate to COVID-19 vaccine); participant-reported	F1:<br>Directly after intervention	No external funding	None declared

Study (Author, Year) Country	Period of time of data collection	Population	Intervention	Outcomes				Sponsor-ship Source	Conflicts of Interest
		Participants description, Sample size (total), Setting		Outcome	Relation to health literacy (HL)	Outcome measure	Timing of outcome measurement		
**Study Design: Uncontrolled Before-After Studies**									
**Bohara et al. 2021** [42] **Nepal**	11th - 14th May 2020 (1st level training)<br><br>1st Sep - 25th Oct 2020 (2nd level training)	18 medical health professionals (doctors, nurses, public health workers, medical practitioners) from five districts of Province 5, Nepal (first-level training; Master-Trainer-Program)<br>1,800 medical health professionals and allied health students (second-level training)	Two-level training curriculum program<br>First level: "Train-the-Trainer"; COVID-19 preparedness by providing information on the critical skills and knowledge<br>Distance delivery mode: via online training<br>Second level: Training provided by the previous qualified master trainers	**Secondary:**<br>*Composite Outcome*<br>COVID-19 related knowledge and awareness	**HL indicator:**<br>Knowledge (composite)<br>**HL component:**<br>Understand (composite)	39-item questionnaire based on module contents (scored 0 'incorrect', 1 'correct'; and true/false and multiple choice response formats for calculating a summation index and a total cumulative index); participant-reported	F1:<br>Post-intervention (not specified)	None reported	None reported
**Brito-Brito et al. 2021** [43] **Spain**	May 12th - July 31st 2020	766 primary healthcare professionals (healthcare and non-healthcare (area of administration, management, service); mixed population study<br>Age median: 45y, Range 28-60y<br>Gender: 79.9% female, 20.1% male	Online training program (through the Primary Care Management's Moodle online training platform)<br>Distance delivery mode: via online platform (with video library, websites, discussion forum, activities with gamified content), based on five modules<br>Duration: five weeks	**Primary:**<br>Perceived COVID-19 knowledge on infection management	**HL indicator:**<br>Knowledge<br>**HL component:**<br>Understand	Online questionnaire, 5-point Likert scale; participant-reported; based on Moorhead et al. [44]	F1:<br>Directly after intervention	No funding	None declared
**Calik et al. 2022** [45] **Turkey**	March 24th - May 26th 2021	62 senior nursing students at the Yıldırım Beyazıt University<br>Age (mean): 22.19 y<br>Gender: 91.9% female, 8.1% male	Infection and Safe Behaviors training Serious games (technology-enhanced) simulation<br>Distance delivery mode: via online training sessions (simulation game)<br>Duration: One session (á 40 min)	**Primary:**<br>General COVID-19 related knowledge	**HL indicator:**<br>Knowledge<br>**HL component:**<br>Understand	Online questionnaire consisted of "true", "false" and "I do not know" options; 10 questions; participant-reported	F1:<br>One week after intervention	No funding	None declared
**Clay et al. 2021** [46] **United States**	July - Sept 2020	76 team members of the outpatient federally qualified health center family medicine residency clinic that include medical assistants, licensed vocational nurses, and physicians (residents and attending)	Educational video	**Primary:**<br>(a) COVID-19 knowledge on infection prevention measures<br>**Secondary:**<br>(b) Perceived compliance to infection prevention measures	**HL indicator:**<br>(a) Knowledge<br>**HL component:**<br>(a) Understand<br>**HL-related outcome:**<br>(b) Compliance (behaviour)	(a-c)<br>15-item post-survey; participant-reported<br>(a) Three questions in a "check all the apply" format, scores ranged from 0 to 11 points; participant-reported<br>(b) Two questions, 5-point scale: 1 = 0-20%, 2 = 21-40%, 3 = 41-	F1:<br>Within an eight week period (not further specified)	No funding	None declared

Study (Author, Year) Country	Period of time of data collection	Population	Intervention	Outcomes				Sponsor-ship Source	Conflicts of Interest
		Participants description, Sample size (total), Setting		Outcome	Relation to health literacy (HL)	Outcome measure	Timing of outcome measurement		
**Study Design: Uncontrolled Before-After Studies**									
				(c) Compliance to infection prevention measures		60%, 4 = 61-80%, 5 = 81-100%<br>(c) Based on a tally method; observed through trained clinicians			
**Díaz-Guio et al. 2020** [47]<br>**Colombia**	Feb - March 2020	61 health care workers (physicians, respiratory therapists, nurses) from an emergency room and intensive care unit of third-level institutions of Armenia, Quindio, Colombia; mixed health professional occupation population<br>Age median (IQR). 32y (26-43y)<br>Gender: 59% female	Simulation-based educational intervention with two cases related to COVID-19 in the Emergency Room and the Intensive Care Unit (ICU)<br>In-person workshop for donning and doffing of PPE	**Primary:**<br>PPE performance (donning, doffing)	**HL indicator:**<br>Performance skills<br>**HL component:**<br>Apply	Checklist (adapted from the (WHO personal protection equipment – U.S. Centers for Disease Control and Prevention, Atlanta) via the 9-point Paas scale (1= 'very', 'very bad'; 9 = 'very', 'very good'); assessed through a reviewer)	F1:<br>During the simulation cases	No funding	None declared
**Elasrag et al. 2021** [48]<br>**Egypt**	July - Nov 2020	50 nurses at Shebin El Koum teaching Hospital and Menofia University Hospital, Egypt<br>Age mean (SD): 34.80y (4.99)<br>Gender: 76% female, 24% male	Educational training program<br>In-person, group-based workshop, with demonstration and Q&A sessions<br>Duration: 4x1 hour intervention	**Primary:**<br>(a) General COVID-19 related knowledge<br>(b) COVID-19 specific infection prevention performance<br>**Secondary:**<br>(c) Attitudes towards COVID-19 infection prevention measures	**HL indicator:**<br>(a) Knowledge<br>(b) Performance skills<br>**HL component:**<br>(a) Understand<br>(b) Apply<br>**HL-related factor:**<br>(c) Attitude	Self-administered questionnaire (Arabic language), content validated within the present study, reliability Cronbach α=0.799<br>(a) Questions are adapted from Gharpure et al. 2020 [49] (correct answer score calculation)<br>(b) Questions are adapted from McIntosh et al. 2020 [50] (correct answer score calculation); assessed through observation of a research team member at training lab<br>(c) Attitude Likert Rating Scale, 9 items (adapted from Salman et al. 2020 [51])	F1:<br>Post-intervention (not specified)	Self-funding, without any external source	None declared

Study (Author, Year) Country	Period of time of data collection	Population: Participants description, Sample size (total), Setting	Intervention	Outcomes: Outcome	Outcomes: Relation to health literacy (HL)	Outcomes: Outcome measure	Outcomes: Timing of outcome measurement	Sponsorship Source	Conflicts of Interest
**Study Design: Uncontrolled Before-After Studies**									
**Espinoza-Castro et al. 2025** [52] **Ecuador**	Aug - Dec 2021	3,498 healthcare workers (doctors, nurses, medical assistants, administrative staff, porters, caterers, and maintenance and cleaning workers) from the public health sector in Ecuador; mixed health professional population<br>Gender: 58.4% female, 41.6% male	Online Massive Open Online Course on infection prevention and control (IPC) of acute respiratory infections with problem-based learning and interactive scenarios to teach IPC principles in the context of COVID-19<br>Duration: approx. 20 h	**Primary:**<br>Perceived COVID-19 specific knowledge on infection prevention control measures	**HL indicator:**<br>Knowledge<br>**HL component:**<br>Understand	26-item Questionnaire; 6-point Likert scale ranging from 'very low knowledge' to 'high knowledge'; participant-reported;	F1:<br>Directly after the intervention	Open Access funding by Projekt DEAL; part of the OH-TARGET (One Health Training and Research Global Network) project (funded by German Federal Ministry for Economic Cooperation and Development (BMZ) and the German Academic Exchange Service (DAAD))	None declared
**Etebarian et al. 2023** [53] **Iran**	Sep 2021	118 dental students who have entered the clinical phase of practice (third year and above)<br>Age mean: 24.2y, Range 21-32 (female)<br>Age mean: 25y, Range 21-40 (male)<br>Gender: 45.45% female, 54.54% male	Educational intervention regarding COVID-19 management in dentistry<br>Webinar via Sky room (as meeting platform) and use of short educational videos<br>Duration: 3 hrs	**Primary:**<br>(a) General COVID-19 related knowledge<br>**Secondary:**<br>(b) Adherence to COVID-19 infection prevention measures<br>(c) Attitude towards COVID-19 infection prevention measures	**HL indicator:**<br>(a) Knowledge<br>**HL component:**<br>(a) Understand<br>**HL-related outcome:**<br>(b) Adherence (Behavior)<br>**HL-related factor:**<br>(c) Attitude	(a-c) Questionnaires, participant-reported<br>(a) 12 items; consisting of 'true,' 'false,' and 'I do not know' options<br>(b) 15 items; 4-point Likert scale ranging from 'never' to 'always'<br>(c) 10 items; 5-point Likert scale ranging from 'completely disagree' to 'completely agree'<br>Self-developed, validated by eight content experts, pilot-tested<br>Reliability<br>Cronbach α = 0.7	F1:<br>Directly after the intervention<br>F2:<br>Two months after intervention	Alborz University of Medical Sciences	None declared

Study (Author, Year) Country	Period of time of data collection	Population	Intervention	Outcomes				Sponsor-ship Source	Conflicts of Interest
		Participants description, Sample size (total), Setting		Outcome	Relation to health literacy (HL)	Outcome measure	Timing of outcome measurement		
**Study Design: Uncontrolled Before-After Studies**									
**Fadel et al. 2025** [54] **Egypt**	Aug - Sep 2021	350 undergraduate nursing students enrolled across four academic levels at the Faculty of Nursing, Mansoura University, Egypt<br><br>Age:<br>18-19y: 52%<br>20-21y: 21.4%<br>22-24y: 26.6%<br>Gender: 68.9% female, 31.1% male	Virtual educational session on knowledge and attitudes regarding COVID-19 vaccination<br>Duration: 4h (2 sessions á 2h)	**Primary:**<br>(a) COVID-19 related vaccine knowledge<br>**Secondary:**<br>(b) Attitudes towards COVID-19 vaccination	**HL indicator:**<br>(a) Knowledge<br>**HL component:**<br>(a) Understand<br>**HL-related factors:**<br>(b) Attitude	Self-administrative structured questionnaire<br>(a) 42-items; scoring from 0 'incorrect' to 1 'correct'; total score range 0-42; pilottest with 35 students and content validity with a panel of 5 experts were conducted; participant-reported<br>Reliability Cronbach α=0.84<br>(b) Vaccination Attitudes Examination (VAX) Scale; 12 statements; 5-point Likert scale ranging from 'low' to 'very/high'; adaopted from Martin and Perie (2017)[55]; Known-group validity was conducted: inter-factor correlation 0.47-0.79<br>Reliability Cronbach α=0.87-0.93	F1: Post-intervention (not specified)	No funding	None declared
**Findyartini et al. 2021** [56] **Indonesia**	Aug - Nov 2020	5,432 newly graduated medical doctors (3,202 participants in Batch 1, Aug 2020 and 2,230 participants in Batch 2, Nov 2020) in Indonesia<br>Gender:<br>69.86% female, 30.14% male (Batch 1)<br>67.62% female, 32.38% male (Batch 2)	Modul Tanggap Pandemi COVID-19 untuk Dokter Internship Indonesia (MTPC-I) as massive Open Online Course<br>Consisted of two parts<br>Part 1: Based on fundamental knowledge of COVID-19 (mandatory)<br>Part 2: Included supplemental and enriching knowledge about COVID-19 (recommended)	**Primary:**<br>COVID-19 specific knowledge on<br>(a) Infection prevention control measures<br>(b) Epidemiology<br>(c) Virus transmission<br>(d) COVID-19 management<br>(e) COVID-19 vaccination	**HL indicator:**<br>(a-e) Knowledge<br>**HL component:**<br>(a-e) Understand	Questionnaire; participant-reported; score-based; Items were validated by the team	F1: Directly after each intervention	No funding	None declared

Study (Author, Year) Country	Period of time of data collection	Population	Intervention	Outcomes				Sponsor-ship Source	Conflicts of Interest
		Participants description, Sample size (total), Setting		Outcome	Relation to health literacy (HL)	Outcome measure	Timing of outcome measurement		
**Study Design: Uncontrolled Before-After Studies**									
**Fuentes et al. 2023 [57]** **Cuba**	Mar-Apr 2020	79 first year medical students at the University Polyclinic 'Joaquín de Agüero'	Educational intervention in-person and group-based<br>Duration: 5hrs (one sessions/week for five weeks)	**Primary:**<br>(a) General COVID-19 related knowledge<br>(b) COVID-19 specific knowledge on infection prevention control measures<br>(c) COVID-19 specific knowledge on virus transmission	**HL indicator:**<br>(a-c) Knowledge<br>**HL component:**<br>(a-c) Understand	(a-c) Questionnaire (up to 100 points); participant-reported	F1:<br>Directly after intervention	None reported	None declared
**Garcia et al. 2024 [58]** **Guatemala**	-	210 lay midwives in Guatemala across 11 locations (healthcare clinics, schools, community centers (Peten) and Refuge International healthcare clinic (Sarstun)<br>Age mean: 61y, Range 21-85y<br>Gender: 98% female, 2% male	Evidence-based educational sessions about COVID-19 for low-literacy lay midwives<br>Delivery Methods: Repetition, role plays, and storytelling, with supporting materials: Laminated COVID-19 reminder cards to encourage knowledge retention	**Secondary:**<br>*Composite Outcome*<br>COVID-19 knowledge on symptoms, infection prevention control measures, vaccination and breastfeeding	**HL indicator:**<br>Knowledge (composite)<br>**HL component:**<br>Understand (composite)	12 questions, including multiple-choice, fill-in-the-blank, and yes/no items, scoring: total of 24 correct answers; participant-reported; Face validity was conducted, Fog index: 0.35	F1:<br>Directly after intervention	Intramural grant from the University of Utah College of Nursing and the American College of Nurse Midwives Foundation	None declared
**Girard et al. 2022 [59]** **Switzerland**	Jan - Feb 2021	230 health professionals (nursing, medical, medico-therapeutic, administration, logistic, and kitchen staff) working in two inpatients (acute and post-acute) units of the service of Geriatric medicine and geriatric rehabilitation of the University of Lausanne Medical Center, Switzerland<br>Age:<br>20–29y: 36.1%<br>30–39y: 18.6%<br>40–49y: 19.6%<br>50+ y: 25.8%<br>Gender: 78.4% female	Information session conducted by a clinical nurse specialist and a senior geriatrician<br>Two segments:<br>(1) Video including frequently asked questions on COVID-19 vaccines' efficacy and safety (15 min)<br>(2) Question-answer discussion (15 min)<br>Duration: 420 min (30 min/session, 14x)	**Primary:**<br>(a) Perceived COVID-19 related vaccine knowledge<br>**Secondary:**<br>(b) Confidence in providing information about COVID-19 vaccine	**HL indicator:**<br>(a) Knowledge<br>**HL component:**<br>(a) Understand<br>**HL-related factor:**<br>(b) Confidence (self-efficacy)	Self-administered questionnaire (participant-reported)<br>(a) Respond categories: 'high', 'intermediate', 'low', 'no knowledge'<br>(b) Respond categories: 'completely', 'relatively', 'not really', 'not at all confident')	F1:<br>Directly after intervention	None declared	None declared
**Greaves et al. 2023 [60]** **United States**	Jun 2020	48 first-year resident physicians who needed American Heart Association life support certifications (advanced cardiac life support, basic life support, etc.) (24 internal medicine, 12 general surgery, 6	Formative training program utilising modified CDC guidelines on procedures of PPE; step-by-step simulation-based review of proper PPE using sequence with deliberate practice and feedback	**Primary:**<br>(a) COVID-19 specific infection prevention knowledge on PPE (donning & doffing)	**HL indicator:**<br>(a, b) Knowledge<br>(c) Performance skills<br>**HL component:**<br>(a, b) Understand	(a, b & d) Questionnaire with 5-point Likert Scale; participant-reported<br>(c)	F1:<br>Directly after intervention	None declared	None declared

Study (Author, Year) Country	Period of time of data collection	Population	Intervention	Outcomes				Sponsor-ship Source	Conflicts of Interest
		Participants description, Sample size (total), Setting		Outcome	Relation to health literacy (HL)	Outcome measure	Timing of outcome measurement		
**Study Design: Uncontrolled Before-After Studies**									
		emergency medicine, 3 neurology, 3 psychiatry)	1. Debrief and Reflection<br>2. PPE Video Instruction<br>3. Simulated CDC PPE<br>4. Summary and Review<br>Duration: 30 min	(b) COVID-19 specific knowledge on virus transmission<br>(c) PPE performance (donning & doffing)<br>**Secondary:**<br>*Composite outcome*<br>(d) Perceived confidence and knowledge in using PPE (donning & doffing)	(c) Apply<br>(d) Understand (composite)<br>**HL-related factors:**<br>(d) Confidence (self-efficacy, composite)	I. Video records; scored by two of five randomly assigned independent raters;<br>i. Donning PPE (1-5 points)<br>ii. Cardiopulmonary resuscitation and positive pressure ventilation using a bag valve mask on manikins (no rating)<br>iii. Doffing PPE (1-7 points)<br>II. Contamination check; scored by two independent assessors;<br>Topical non-toxic invisible highlighter fluid as tracer;<br>Number of contaminated areas was recorded for each specific body area<br>Inter-rater reliability (intraclass correlation coefficient, ICC) = 0.96			
**Gupta et al. 2023** [61]<br>**India**	May 5th - 14th 2020	100 healthcare workers (medical personnel, medical students, and laboratory staff) fromLok Nayak Jai Prakash Hospital in Delhi<br><br>Age mean (SD): 33.07y (8.6)<br>Gender: 61.25% female, 38.75% male	Short video-based educational intervention<br>Duration: 8 min	**Primary:**<br>(a) General COVID-19 related knowledge<br>(b) COVID-19 specific infection protection performance skills in laboratory practice<br>**Secondary:**<br>(c) Attitudes towards COVID-19 (not further specified)	**HL indicator:**<br>(a) Knowledge<br>(b) Performance skills<br>**HL component:**<br>(a) Understand<br>(b) Apply<br>**HL-related factors:**<br>(c) Attitude	Questionnaire<br>(a) 12 questions<br>(b) 18 questions<br>(c) 5 questions, scored 1 'correct', 0 'incorrect'<br>Development based on frequently asked question from the World Health Organization (WHO) Web site (no specific reference re-	F1:<br>Directly after intervention (lasted 5 days)	No funding	None declared

Study (Author, Year) Country	Period of time of data collection	Population	Intervention	Outcomes				Sponsorship Source	Conflicts of Interest
		Participants description, Sample size (total), Setting		Outcome	Relation to health literacy (HL)	Outcome measure	Timing of outcome measurement		
**Study Design: Uncontrolled Before-After Studies**									
						ported); Participant-reported; Content validity was conducted with 10 participants; Reliability Cronbach α=0.731			
**Halemani et al. 2020 [62] India**	-	40 final year BSc nursing students at the College of Nursing in Sanjay Gandi Postgraduate Institute of Medical Sciences<br>Age:<br>< 22y (75%)<br>22-26y (25%)<br>Gender: 55% female, 45% male	Training program<br>Delivery mode: In-person<br>Delivery method: lecture & discussions<br>Duration: 1 d (6 hrs)	**Primary:**<br>COVID-19 related knowledge on infection prevention control measures	**HL indicator:**<br>Knowledge<br>**HL component:**<br>Understand	Questionnaire with multiple options with one correct answer; participant-reported; validated within the present study, reliability of the tool has checked by 10% of the study population, r=0.8	F1:<br>Post-intervention (not specified)<br>F2:<br>One week after intervention	No funding	None declared
**Han et al. 2022[63] China**	Mar 9th – 18th 2020	5,425 medical staff (1,669 doctors, 3,129 nurses, and 627 medical technicians) from the First Affiliated Hospital of Nanjing Medical University for IPC training covered all clinical and medical technical departments (Radiology, Pharmacy, Medical Laboratory Science, Pathology, Ultrasound Diagnosis, Blood Transfusion, Nuclear Medicine, Nutrition, Forensic Science, etc.), while training for other staff (administrative, property, logistical support, etc.) was not mandatory<br>Age:<br><30y.: 44.56%<br>30–39y: 36.79%<br>40–49y: 11.23%<br>50+ y: 7.42%<br>Gender: 80.69% female 19.31% male	Four stage training assessment<br>Stage 1: Pre-assessment<br>Stage 2 (March 12-14): Releasing electronical manual of IPC "Should Be Known and Done", using WeChat group and hospital intranet OA system for dissemination<br>Stage 3 (March 15-16): Online practicing via training question bank; 166 questions (66 on COVID-19 knowledge prevention & control)<br>Stage 4: Post-assessment<br>Duration: 10 d	**Primary:**<br>COVID-19 related knowledge on infection prevention control measures	**HL indicator:**<br>Knowledge<br>**HL component:**<br>Understand	Assessment comprises 20 questions randomly issued from the question bank, each worth 5 points, for a total of 100 points; participant-reported	F1:<br>One or two days after the intervention	National Key R & D Program Fund (2020YFC0848100), the Research Foundation for Infection Prevention and Control of Chinese Geriatrics Association (GRYJ-LRK2018016), and the Jiangsu Provincial Hospital Management Innovation Research Fund (JSYGY-3-2019-484) supported this project. The funders were pivotal in all aspects of the study,	None declared

Study (Author, Year) Country	Period of time of data collection	Population: Participants description, Sample size (total), Setting	Intervention	Outcomes: Outcome	Outcomes: Relation to health literacy (HL)	Outcomes: Outcome measure	Outcomes: Timing of outcome measurement	Sponsorship Source	Conflicts of Interest
**Study Design: Uncontrolled Before-After Studies**									
								from its design to publication.	
**Hwang et al. 2021** [64] **South Korea**	Sept 10th 2020 - June 27th 2021	78 nursing-students from nursing college in South Korea<br>Age mean (SD): 22.2y (1.20)<br>Gender: 85.9% female, 14.1% male	**Intervention 1:**<br>Pre-training (educational lectures)<br>**Intervention 2:**<br>Disaster nursing simulation training program (developed by Park [65]) according to the Jeffries simulation model<br>Duration of simulation: 40min	**Primary:**<br>COVID-19 specific knowledge of clinical presentation of SARS-CoV-2	**HL indicator:**<br>Knowledge<br>**HL component:**<br>Understand	Questionnaire; participant-reported; dichotomous scale; 9 items; extracted from a WHO survey [66])	F1:<br>After first intervention<br>F2:<br>After second intervention	Internal funding for customized study by Kyung Hee University	None declared
**Instrum et al. 2022** [67] **Canada**	Oct & Dec 2020	62 frontline workers<br>(52% nurses, 16 % paramedics)	Nasopharyngeal swab trainer simulation, self-evaluation of swab accuracy by using an integrated video recording before and after use of the trainer and standardized instructional videos (training module) provided by the CDC and New England Journal of Medicine<br>Delivery mode: Self-directed technologically enhanced in-person learning	**Secondary:**<br>Perceived confidence in performing nasopharyngeal swab	**HL-related factors:**<br>Confidence (self-efficacy)	Questionnaire (5-point Likert scale 'not confident' to 'extremely confident'; participant-reported)	F1:<br>Directly after intervention	Funding was received from the Peter Cheski Innovative Research Fund (non-profit)	One author (R.W.K.) is President and CEO of Ahead Simulations. He contributes to the development and provision of simulators free of charge, to study design, and approved the manuscript. He was not involved with data collection or analysis to limit the potential for bias. Other authors have none declared.
**Kasapoglu et al. 2023** [68] **Turkey**	Completed Sep 2021	463 students in Elderly Care, Home Patient<br>Care, and First and Emergency Aid programs<br>Age mean: 20.6y, Range 18 - 42<br>Gender: 73.9% female, 26.1% male	Online COVID-19 Patient Care and Emergency Intervention Interprofessional Training in two online sessions by a trainer<br>Duration: 300 minutes	**Primary:**<br>General COVID-19 related knowledge	**HL indicator:**<br>Knowledge<br>**HL component:**<br>Understand	Questionnaire "Interprofessional Information Form About COVID-19", true/false statement about COVID, developed by	F1:<br>Directly after intervention	None declared	None declared

Study (Author, Year) Country	Period of time of data collection	Population: Participants description, Sample size (total), Setting	Intervention	Outcomes: Outcome	Outcomes: Relation to health literacy (HL)	Outcomes: Outcome measure	Outcomes: Timing of outcome measurement	Sponsorship Source	Conflicts of Interest
**Study Design: Uncontrolled Before-After Studies**									
						the researcher; participant-reported			
**Kaufman et al. 2024 [69]**<br>**Fiji**	Jul 2022	35 community health workers, doctors, nurses, faith organisation leaders and representatives from Fiji Red Cross, Ministry of Health (MoH), village headman, public health students and sports representatives (mixed-population)<br>Gender: 77% female, 23% male	Vaccine Champions programme training<br>Using vaccine communication research, Motivational Interviewing, Role-play activities<br>Duration: 2 days	**Primary:**<br>COVID-19 specific vaccine knowledge on side effects	**HL indicator:**<br>Knowledge<br>**HL component:**<br>Understand	3-items; developed by the research team; participant-reported	F1:<br>Directly after intervention	The Australian Department of Foreign Affairs and Trade through the Australian Regional Immunisation Alliance–Regional Immunisation Support and Engagement (ARIA-RISE) scheme (grant number: n/a)	JF, JT, RD, LiV: employed by Fiji MoH; involved in vaccine program delivery during study. Fiji MoH reviewed manuscript prior to publication. HS: received investigator-driven research funding from industry (incl. Moderna) within past 3 yrs; no funding used for this study. All other authors: no competing interests
**Kharel et al. 2022 [70]**<br>**United States**	Jan 2021	4,291 clinicians, public health workers, community health workers, policy makers, hospital administrators worldwide of 55 countries in all six WHO regions (Africa, Amerika, Eastern Mediterranean, Europe, South East Asia, Western Pacific); mixed health professional occupation population	Virtual training-of-trainers program via Zoom®<br>**Primary training**<br>Distance delivery mode with blended methods:<br>(i) interactive didactic modules<br>(ii) case-based learning<br>(iii) video simulations	**Primary:**<br>General COVID-19 related knowledge	**HL indicator:**<br>Knowledge<br>**HL component:**<br>Understand	10-question survey; summed correct responses; score-based; participant-reported	F1:<br>Immediately after the 4th day of training	The program development and implement-ation were funded by Project HOPE®. The paper	None declared

Study (Author, Year) Country	Period of time of data collection	Population	Intervention	Outcomes				Sponsorship Source	Conflicts of Interest
		Participants description, Sample size (total), Setting		Outcome	Relation to health literacy (HL)	Outcome measure	Timing of outcome measurement		
**Study Design: Uncontrolled Before-After Studies**									
			**Secondary training**<br>In person, conducted by the primary training participants using the program materials (e. g. presentations, teaching manuals, video simulation, feedback forms)<br>Duration: 3 hrs/d for 4 days<br>Provided language: English and Spanish					and analysis were not funded.	
**Khari et al. 2022** [71] **Iran**	2021	100 seventh- and eighth-semester nursing students from the School of Nursing and Midwifery<br>Age mean (SD): 22y (1.25)<br>Gender: 61% female	Online curriculum for COVID-19 patient care<br>Delivery mode: audio files, videos, photos, educational files, Q & A about the trained items<br>Duration: 360 min (three sessions of 2h for two weeks)	**Primary:**<br>General COVID-19 related knowledge	**HL indicator:**<br>Knowledge<br>**HL component:**<br>Understand	Questionnaire, 30 questions in the form of 10 items; three response options 'agree', 'disagree', 'I do not know'; score based (range 0-30); participant-reported; developed by Khari et al. 2022 [71]<br>Validated by ten researchers<br>Reliability Cronbach α=0.80	F1: Directly after the intervention<br>F2: Four weeks after intervention	Shahid Beheshti University of Medical Sciences	None declared
**Kobayashi et al. 2023** [72] **Japan**	Apr 2021 - Mar 2022	224 health professionals in psychiatric institutes, located in five prefectures in Japan (Tokyo, Chiba, Aichi, Ehime, Fukuoka) and public health care centers<br>Age mean (SD): 47.4y (9.5)<br>Gender: 46% female, 54% male	Online training video on COVID-19 IPC<br>Duration: 30 minutes	**Primary:**<br>(a) COVID-19 specific knowledge on infection control measures<br>**Secondary:**<br>(b) Perceived confidence in COVID-19 specific infection prevention measures (participant-reported)<br>(c) Perceived confidence in PPE performance (donning &	**HL indicator:**<br>(a) Knowledge<br>**HL component:**<br>(a) Understand<br>**HL-related factors:**<br>(b, c) Confidence (self-efficacy)	(a) Questionnaire (quizzes about COVID-19 IPC) based on multiple choice; participant-reported<br>(b & c) Questionnaire based on 7-point Likert Scale ("very weak" to "very confident")<br>Developed by the core members of the study; Contextually validated by outside experts from the study	F1: Directly after the intervention<br>F2: Three months after intervention	2021 Project for the Promotion of Comprehensive Welfare for Persons with Disabilities.	None declared

Study (Author, Year) Country	Period of time of data collection	Population	Intervention	Outcomes				Sponsor-ship Source	Conflicts of Interest
		Participants description, Sample size (total), Setting		Outcome	Relation to health literacy (HL)	Outcome measure	Timing of outcome measurement		
**Study Design: Uncontrolled Before-After Studies**									
				doffing, participant reported)					
**Kufel et al. 2022** [73] **United States**	Feb 2021	85 pharmacy students from Binghamton University and Saint John Fisher College, New York	Interactive didactic class with active learning<br>Duration: 180 min (including 10 min presentation journal club summary for each group & 60 min interactive lecture on COVID-19 with clinical scenarios based on vignettes)	**Secondary:**<br>*Composites outcomes*<br>(a) COVID-19 knowledge on infection prevention and management<br>(b) Perceived confidence infection prevention and management	**HL indicator:**<br>(a) Knowledge (composite)<br>**HL component:**<br>(a) Understand (composite)<br>**HL-related factors:**<br>(b) Confidence (self-efficacy)	Questionnaires (participant-reported):<br>(a & b)<br>10 questions, based on a scale from 1 'strongly disagree' to 5 'strongly agree'<br>Reliability Cronbach α=0.93 (post-intervention scale)<br>Pilot tested by an infectious disease pharmacist and pharmacy resident	F1:<br>Directly after the intervention	No external funding	One author (WK) has received research grants from Merck and Melinta Therapeutics, and served on the advisory board for Theratechnologies, Inc. One author's (LA) husband is an employee of Merck. All other contributors reported no conflicts of interest.
**Lalitha et al. 2024** [74] **India**	-	100 dental professionals (academicians, practitioners and students);<br>Two interventions conducted:<br>Intervention 1: Webinar (80 participants) Intervention 2: Information Education Communication (IEC) Materials (20 participants)<br><br>Age mean (SD)<br>34.94y (7.87) (Total)<br>35.81y (7.97) (Webinar)<br>31.45y (6.52) (IEC)<br><br>Gender<br>56% female, 44% male (Total)<br>58.75% female, 41.25% male (Webinar)<br>45% female, 55% male (IEC)	Intervention 1: Webinar with recorded video<br>Interventions 2: IEC with poster-based material<br>Both interventions were supervised by two experts	**Primary:**<br>COVID-19 specific knowledge on oral health	**HL indicator:**<br>Knowledge<br>**HL component:**<br>Understand	Self-administered questionnaire; 10 items (9 close-ended and 1 open-ended); developed and validated by experts	F1:<br>Post-intervention (not specified)	None reported	None reported

Study (Author, Year) Country	Period of time of data collection	Population: Participants description, Sample size (total), Setting	Intervention	Outcomes: Outcome	Outcomes: Relation to health literacy (HL)	Outcomes: Outcome measure	Outcomes: Timing of outcome measurement	Sponsorship Source	Conflicts of Interest
**Study Design: Uncontrolled Before-After Studies**									
**Li et al. 2020 [75]**<br>**China**	-	35 nurses from the emergency surgery department<br>No sociodemographic characteristics reported	Online training curriculum program<br>Delivery mode: Online distance learning via various platforms (WeChat® group Tencent, Shenzhen, China), WeChat official accounts, the Aesculap Academy, (Center Valley, PA), and DingTalk (Alibaba Group, Hangzhou, China))<br>Duration:<br>in total 300 min.(20 periods, 15 min. per period)	**Primary:**<br>(a) COVID-19 specific knowledge on infection prevention control measures<br>(b) COVID-19 specific infection protection performance skills	**HL indicator:**<br>(a) Knowledge<br>(b) Performance skills<br>**HL component:**<br>(a) Understand<br>(b) Apply	(a) Questionnaire, participant-reported (no further information reported)<br>(b) Evaluated (randomly) through observation of seven performance steps (donning/doffing medical mask, protective suit and hand hygiene)<br>(a & b)<br>Graded by the same standard, by 100 scores	F1:<br>Post-intervention (not specified)	No funding	No declared
**Macht et al. 2022 [76]**<br>**Kosovo**	Sep 2020 – Mar 2021	25 nurses and doctors from all state clinics (seven regional hospitals and the University Clinic Pristina)	Video conference-based sessions including practical exercises (e.g., correct performance of hygienic hand disinfection, correct donning and doffing of PPE), information materials and Q & A<br>Duration: 900 min<br>(10 sessions x 90 min)	**Primary:**<br>COVID-19 specific knowledge on infection prevention control measures	**HL indicator:**<br>Knowledge<br>**HL component:**<br>Understand	Paper-pencil questionnaire; participant-reported; consists of 12 image vignettes; each image was presented for 30 seconds;<br>Based on the test by Heiniger et al. 2021 [77], translated into Albanian guided by a linguist and cultural scientist, translation's translatability and cultural relevance based on 'Translation guidelines and translation documentation of the European Social Survey'; The image vignettes were checked by hygiene experts	F1:<br>Directly after the intervention	This manuscript was funded by GIZ (project no. 1210038.105.86) under the program "Special competition COVID-19 Response: Together against the pandemic	None declared
**Mark et al. 2020 [78]**<br>**United States**		62 health care worker of emergency department, hospital wards, and clinics where COVID-19 testing regularly takes place	Simulation-based training<br>Delivery mode: In person, goup-based<br>Delivery method: lecture & simulation on a high-fidelity airway model	**Primary:**<br>(a) COVID-19 specific knowledge on performing nasopharyngeal swab	**HL indicator:**<br>(a) Knowledge<br>**HL component:**<br>(a) Understand<br>**HL-related factors:**	(a) Questionnaire (multiple-choice, participant-reported)<br>(b) Nasopharyngeal Swab Self-Assessment: standardised clinical	F1:<br>Directly after intervention	No funding	Non declared

Study (Author, Year) Country	Period of time of data collection	Population	Intervention	Outcomes				Sponsorship Source	Conflicts of Interest
		Participants description, Sample size (total), Setting		Outcome	Relation to health literacy (HL)	Outcome measure	Timing of outcome measurement		
**Study Design: Uncontrolled Before-After Studies**									
			Duration: 3d, 6 teaching sessions (á 20 min)	**Secondary:**<br>(b) Perceived confidence in COVID-19 specific knowledge on performing nasopharyngeal swab	(b) Confidence (self-efficacy)	competency questionnaires, 5-point Likert scale ranging from ''No knowledge', up to ''Highly knowledgeable and confident, independent'			
**McConnell et al. 2024 [79]**<br>**United Kingdom**	Jan 4th, 2021 - Jan 28th, 2022	412 first year of a BSc honours degree in professional nursing program across four fields (adult, mental health, learning disabilities, and children) in one university in Northern Ireland	Digital serious game intervention 'Coronavirus—know the facts'<br>HTML5-based COVID-19 game including true/false questions on facts and myths about COVID-19; immediate feedback provided; randomly generated questions; multiple attempts allowed<br>Duration: < 5 min; accessible over 30 days	**Primary:**<br>General COVID-19 knowledge	**HL indicator:**<br>Knowledge<br>**HL component:**<br>Understand	25 items (true/false) based on the World Health Organisation's list of top COVID-19 myths[80]; participant-reported; No identical items between the serious game and the questionnaire	F1:<br>Directly after intervention	No external funding	None declared
**Mektirat et al. 2021 [81]**<br>**Thailand**<br>**(Only abstract)**	Jun 2019	94 undergraduate health science students (22 nursing, 21 dentistry, 21 medical technology, 19 pharmacy and 12 veterinary)	Project-based interprofessional education curriculum	**Primary:**<br>(a) COVID-19 knowledge (not specified)<br>**Secondary:**<br>(b) Attitudes towards COVID-19 infection prevention measures	**HL indicator:**<br>Knowledge<br>**HL component:**<br>(a) Understand<br>**HL-related factors:**<br>(b) Attitude	No information	F1:<br>Post-intervention (not specified)	None reported	None reported
**Mohamed et al. 2023 [82]**<br>**Papua New Guinea**	Round 1: 18th Jun - 4th Sep 2020<br><br>Round 2: 18th Sep - 18th Dec 2020	364 frontline health workers and health management staff in Papua New Guinea<br>Gender: 55% female, 39% male	Blended intervention approach:<br>(i) Online training platform with 14 self-paced modules related to COVID-19<br>Duration: Approx. 420 min (14 modules × ~30 min each)<br>(ii) Lectures by health experts via video conferencing<br>Duration: 1hr/lecture, 1-2x weekly<br>(iii) Downloadable resources (including local and international guidelines, checklists, and posters)	**Primary:**<br>COVID-19 specific knowledge on<br>(a) General COVID-19<br>(b) Infection Control<br>(c) Outbreak control<br>(d) Infection Control Management	**HL indicator:**<br>(a-d) Knowledge<br>**HL component:**<br>(a-d) Understand	Questionnaire (participant-reported, pre-post knowledge quiz, multiple choice and short answer questions in online platform)<br>4 of 14 modules are directed to the primary outcome; Module 2 was only in Round 2	F1:<br>Post-intervention (not specified)	The Australian Government's Department of Foreign Affairs and Trade.<br>The funder had no role in the design of the study; collection, analysis, and interpretation of data; or in writing the manuscript.	CoHELP was funded by the Australian Government in partnership with the Government of Papua New Guinea, and implemented by the PNGAus Partnership, the PNG National Department of Health, WHO, Johnstaff International

Study (Author, Year) Country	Period of time of data collection	Population	Intervention	Outcomes				Sponsor-ship Source	Conflicts of Interest
		Participants description, Sample size (total), Setting		Outcome	Relation to health literacy (HL)	Outcome measure	Timing of outcome measurement		
**Study Design: Uncontrolled Before-After Studies**									
			(iv) Access to recorded online training sessions<br>(v) An online closed discussion board to enable clinicians to ask questions of external experts						Development and the Burnet Institute. Hinabokiole Kama, Norah Hau'ofa, Carmellina Amol, and Madi Jones are employed by the company Johnstaff International Development
**Nassar et al. 2024** [83]<br>**Yemen**	Apr - Dec 2021	186 healthcare workers working at COVID-19 isolation centers and caring for COVID-19 patients; mixed health professional occupation population<br>Age median (IQR): 33y (29-40)<br>Gender: 21% female, 79% male	Training couses based on interactive adult-learning theory; delivered through Power-Point, case studies, group works, and role-plays<br>Developed by experts based on WHO guidelines and the National Training Manual for IPC<br><br>First phase: train the trainers<br>Second phase: train the remaining healthcare workers across Yemen	**Primary:**<br>(a) General COVID-19 related knowledge<br>**Secondary:**<br>(b) Attitudes towards COVID-19<br>(c) Prevalence of infection prevention behaviour	**HL indicator:**<br>(a) Knowledge<br>**HL component:**<br>(a) Understand<br>**HL-related factors:**<br>(b) Attitude<br>**HL-related outcome:**<br>(c) Prevention behaviour	Predesigned structured questionnaire<br>(a)16 items; multiple-choice (maximum total score: 40 points)<br>(b) 10 items; 5-point Likert Scale (1 'strongly disagree' to 5 'strongly agree'; maximum total score: 50 points)<br>(a) & (b) Reliability Cronbach α=0.63<br><br>(c)10 items; 5-point Likert Scale (1 'never' to 5 'always', m aximum total score: 50 points)<br>Reliability Cronbach α=0.72<br><br>Items adopted from Al-rubaiee et al. 2020 [84], Ye et al. 2021 [85], Huynh al. 2020 [86], Tadesse et al. 2020 [87], WHO; Validated by three experts; participant-reported	F1:<br>Directly after intervention	The Task Force for Global Health. However, the authors declare that the funder had no role in the conceptualization, design, data collection, analysis, decision to publish, or preparation of the manuscript.	None declared

Study (Author, Year) Country	Period of time of data collection	Population	Intervention	Outcomes				Sponsor-ship Source	Conflicts of Interest
		Participants description, Sample size (total), Setting		Outcome	Relation to health literacy (HL)	Outcome measure	Timing of outcome measurement		
**Study Design: Uncontrolled Before-After Studies**									
**Naz et al. 2022** [88] **India**	1st Oct - 31st Dec 2020	421 health professionals (nurses, house-keeping, security staffs, technicians and attendants of Shri Mahant Indiresh Hospital, Dehradun)<br>Age:<br>20-24y: 33.5%<br>25-29y: 34.9%<br>30-34y: 13.4%<br>35-39y: 9.7%<br>40-44y: 4.5%<br>≥ 45y: 4%<br>Gender: 82.2% female, 17.8% male	Health education sessions (two-way communication between the research team and respondents, using audio visuals aids)	**Secondary:**<br>*Composite outcome*<br>COVID-19 specific knowledge on infection prevention control measures and management	**HL indicator:**<br>Knowledge (composite)<br>**HL component:**<br>Understand (composite)	Self-administered and validated questionnaire (20 multiple-choice questions with one correct answer, translated in English and Hindi)	F1:<br>Directly after intervention	No funding	None declared
**Odusanya et al. 2022** [89] **Nigeria**		63 health professionals at primary health care facilities in two local government areas of Lagos State (Ikeja, Alimosho)<br>Age mean (SD): 39.2 y. (9.9)<br>≤ 30y: 22.2%<br>≥ 30y: 77.8%<br>Gender: 85.7% female, 14.3% male	Virtual training on COVID-19 including five main modules based on the theory of change model (epidemiology, risk communication, community engagement, prevention of COVID-19, and vaccination against COVID-19)<br>Delivery mode: self-developed website<br>Blended delivery methods (e.g. videos, lectures, practical demonstrations, assignments, Q & A sessions)<br>Duration of modules:<br>125 min (5 modules, 25 min each)	**Primary:**<br>(a) General COVID-19 related knowledge<br>**Secondary:**<br>(b) Adherence to COVID-19 infection prevention measures<br>(c) Attitude towards COVID-19 infection prevention measures	**HL indicator:**<br>(a) Knowledge<br>**HL component:**<br>(a) Understand<br>**HL-related outcome:**<br>(b) Adherence (Behaviour)<br>**HL-related factor:**<br>(c) Attitude	Questionnaire (participant-reported)<br>(a) 11 items; based on 'yes', 'no' and 'don't know' (correct answer = 1-point, max. score = 33)<br>(b) 9 items; based on 4-point Likert Scale 'always' to 'never' (Range: 1-4, max. score: 36)<br>(c) 9 items; 5-point Likert Scale: 'strongly disagree' to 'strongly agree' (Range: 1-5, max. score 45)<br>Development based on Honarvar et al. 2020 [90] and Paul et al. 2020 [91]<br>Face and content validity were conducted by the research team	F1:<br>Directly after intervention	One author (OOO) received a research grant from Lagos State Research and Innovation Council. (https://lasric.lagosstate.gov.ng) The funders had no role in study process.	None declared

Study (Author, Year) Country	Period of time of data collection	Population: Participants description, Sample size (total), Setting	Intervention	Outcomes: Outcome	Relation to health literacy (HL)	Outcome measure	Timing of outcome measurement	Sponsorship Source	Conflicts of Interest
**Study Design: Uncontrolled Before-After Studies**									
						Reliability<br>(a) Cronbach α=0.63<br>(b) Cronbach α=0.86<br>(c) Cronbach α=0.60			
**Otu et al. 2021a** [92]<br>**Nigeria**	March 30th<br>-<br>June 20th 2020	1,051 community health extension workers, doctors, laboratory technicians, nurses, pharmacists and other (environmental health officers and biomedical scientists) from 275 health facilities in 25 states across Nigeria<br>Age:<br>20-29y: 13%<br>30-39y: 32%<br>40-49%: 33%<br>50-59y: 21%<br>≥ 60y: 1%<br>Gender: 50% female, 47% male (3% missing)	E-health learning with the In-Strat COVID-19 tutorial application (using the MediXcel Lite health technology platform)<br>The education module was based on content developed by the Foundation for Healthcare Innovation and Development<br>Distance delivery mode via tutorial application<br>Duration: >60min<br>Languages:<br>English, Hausa, Igbo and Yoruba	**Primary:**<br>General COVID-19 related knowledge	**HL indicator:**<br>Knowledge<br>**HL component:**<br>Understand	7-item questionnaire (multiple-choice, participant-reported)	F1:<br>Directly after intervention	The authors disclosed receipt of the following financial support for the research, authorship, and/or publication of this article: They are appreciative of the intellectual contributions from the Foundation for Healthcare Innovation and Development and the UKaid/DFID funded COMDIS-HSD research program consortia.	None declared
**Otu et al. 2021b** [93]<br>**Nigeria**	March 3rd - April 12th 2020	311 health workers (Community Health Extension Workers, nurses, medical records officers, community health officers and laboratory scientists) in primary health care facilities in all 20 local government areas in Ogun State<br>First round: 82.8 % female (n=29)<br>Second round: 63% female (n=282)	E-health learning with the In-Strat COVID-19 tutorial application (using the MediXcel Lite health technology platform)<br>The education module was based on content developed by the Foundation for Healthcare Innovation and Development<br>Distance delivery mode via application tutorial<br>Languages: English, Hausa, Igbo and Yoruba	**Primary:**<br>General COVID-19 related knowledge	**HL indicator:**<br>Knowledge<br>**HL component:**<br>Understand	10-item Questionnaire (multiple choice, participant-reported)	Not reported	Not reported	One author (OO) is employed by In-Strat Limited Liability Company, Montclair, NJ USA. InStrat LLC, Montclair, NJ USA. One author (NN) is employed by Plus91 Technologies Pvt

Study (Author, Year) Country	Period of time of data collection	Population	Intervention	Outcomes				Sponsorship Source	Conflicts of Interest
		Participants description, Sample size (total), Setting		Outcome	Relation to health literacy (HL)	Outcome measure	Timing of outcome measurement		
**Study Design: Uncontrolled Before-After Studies**									
									Ltd., Pune, India; the remaining authours declared no conflicts of interest
**Perera et al. 2022 [94]**<br>**Sri Lanka**	Mid-Mar - Apr 2021	296 public health staff (physicians, nurses and public health mid-wives) and support workers (cleaning staff and transport staff) in Sri Lanka<br>Age mean (SD): 40.7y (10.2) (Range 21-63)<br>Gender: 90.4% female	For public health staff:<br>Combined training sessions (based on public health guidance, IPC guidelines);<br>Delivery mode: Multimedia presentations (PowerPoint & videos on handwashing, donning, doffing)<br>For support workers:<br>Separate training sessions as hospital physicians and nurses<br>Duration: 6h (6 sessions x 1h each)	**Primary:**<br>General COVID-19 related knowledge	**HL indicator:**<br>Knowledge<br>**HL component:**<br>Understand	Questionnaire (30 questions, max. score 30 points), 4 different questionnaires for the professions; participant-reported<br>Piloted among 10 health care workers; reviewed and by an research team in Toronto and Sri Lanka for validity and suitability, translated into Sinhala	F1:<br>Directly after each intervention	Canadian Institute of Health Research and International Development Research Centre (439835)	None declared
**Pokrajac et al. 2020 [95]**<br>**United States**	March 31st - April 8th 2020	117 clinical faculty members and emergency medicine resident physicians from emergency department, at a large, academic tertiary care hospital	Simulation-based mastery learning with a simulated encounter of a patient with COVID-19<br>Combined delivery mode:<br>Distance learning via video and supplemental material and<br>In-person training with demonstration<br>Duration: 1 d	**Primary:**<br>PPE performance (donning & doffing)	**HL indicator:**<br>Performance skills<br>**HL component:**<br>Apply	21-item checklist of steps for donning and doffing PPE (developed within the present study, adapted from existing best-practices guidelines on PPE use from the US Centers for Disease Control and Prevention (Atlanta, GA) and Stanford University (Palo Alto, CA); assessed through trained reviewers	F1:<br>Directly after intervention	No funding	None declared
**Puga et al. 2023 [96]**<br>**Cuba**	Apr - May 2023	20 medical students (fifth year) at the University of Medical Sciences of Camagüey<br>Age mean (SD): 26.3y (4.07)<br>21-25y: 50%<br>26-30y: 35%	Educational intervention during the rotation of the subject Public Health (focus on complications of COVID-19)<br>Duration: 8 weeks	**Primary:**<br>General COVID-19 related knowledge	**HL indicator:**<br>Knowledge<br>**HL component:**<br>Understand	Questionnaire (20 items; 5-point scale (max. score 100 points); participant-reported;	F1:<br>Post-intervention (not specified)	None reported	None declared

Study (Author, Year) Country	Period of time of data collection	Population	Intervention	Outcomes				Sponsorship Source	Conflicts of Interest
		Participants description, Sample size (total), Setting		Outcome	Relation to health literacy (HL)	Outcome measure	Timing of outcome measurement		
**Study Design: Uncontrolled Before-After Studies**									
		≥31y: 15%<br>Gender: 65% female, 35% male				Self-developed by expert's criteria in an interdisciplinary team; Validated by a group of experts			
**Qasmi et al. 2021 [97]**<br>**Pakistan, China**	5th - 13th Apr 2021	52 life sciences and healthcare laboratory professionals<br>Gender: 42.31% female, 57.69% male	International Virtual Training on Biorisk Management (Biosafety & Biosecurity) in laboratories supporting SARS-CoV-2 diagnostics<br>Duration: 21h (7 sess. × 3h)	**Primary:**<br>COVID-19 specific knowledge on biorisk management	**HL indicator:**<br>Knowledge<br>**HL component:**<br>Understand	Questionnaire (multiple-choice, participant-reported)	F1:<br>Directly after intervention	Health Security Partners, USA	None declared (Two reviewers shared an affiliation with one of the authors, but this was disclosed to the handling editor.)
**Roberts EN et al. 2022 [98]**<br>**United States**	Jul 2020 - Feb 2021	7,988 health care providers including nurse practitioners (most in primary care setting)	Continuing education program 'A Case in the Life: COVID-19' included<br>(i) live Q&A sessions<br>(ii) case studies<br>(iii) three webinars (live & on-demand)<br>Webinar 1 (Jul 2020): based on a literature review, national and international guidelines, and expert faculty opinions<br>Webinar 2 (Oct 2020) and 3 (Feb 2021): based on an updated literature review, expert faculty recommendations, new national and international guidelines, and participant feedback from the previous webinar<br>Duration: 2.5-2.67 hrs	**Secondary:**<br>(a) *Composite outcome*<br>COVID-19 related knowledge and competencies<br>(b) Perceived confidence in identifying populations at risk for severe COVID-19	**HL indicator:**<br>(a) Knowledge (composite)<br>**HL component:**<br>(a) Understand (composite)<br>**HL-related factor:**<br>(b) Confidence (self-efficacy)	(a & b) Questionnaire, based on outcome framework recommendations by Moore et al. 2009 [99], participant-reported<br>(a) Webinars 1 and 2: eight multiple-choice knowledge questions, including four (webinar 1) or five (webinar 2) case-based questions<br>Webinar 3: nine knowledge questions, six of which were case based<br>(b) Webinar 1-3: Likert scale	F1:<br>Directly after each intervention	None reported	None declared
**Roberts KJ et al. 2022 [100]**<br>**United States**	Feb - May 2021	37 respiratory therapy students who have completed previous clinical rotations in the ICUs at the Hospital of the University of Pennsylvania or Penn Presbyterian Medical Center in Philadelphia, PA<br>Gender: 67% female, 33% male	Clinical tele-ICU rotations using 'Penn Medicine's' audiovisual platform<br>Duration:<br>8hrs (2 rotations x 4 hrs each)	**Secondary:**<br>COVID-19 related knowledge<br>(not further specified)	**HL indicator:**<br>Knowledge<br>**HL component:**<br>Understand	Questionnaire<br>(participant-reported, scale from 1 'strongly disagree' to 100 'strongly agree')	F1:<br>Directly after intervention	None reported	None declared

Study (Author, Year) Country	Period of time of data collection	Population: Participants description, Sample size (total), Setting	Intervention	Outcomes: Outcome	Outcomes: Relation to health literacy (HL)	Outcomes: Outcome measure	Outcomes: Timing of outcome measurement	Sponsorship Source	Conflicts of Interest
**Study Design: Uncontrolled Before-After Studies**									
**Rosas-Magallanes et al. 2022** [101] **Mexico**	Mar - Oct 2020 and Jan 1st - Oct 31st, 2020	234,634 health professionals	Four e-learning courses through online platform 'CLIMSS' (Massive Online Course organised by the Mexican Social Security Institute) Duration: 37h	**Secondary:** *Composite Outcome* COVID-19 disease specific competencies on knowledge, practice, attitudes	**HL indicator:** Mixed composite outcome based on the knowledge, attitude and practice framework	Assessment, participant-reported (no further information available)	F1: Directly after each intervention	None reported	None declared
**Saati et al. 2022** [102] **Saudia-Arabia**	Oct 2020 - Nov 2021	177 medical undergraduates at Al-Qunfudah College of Medicine, Umm Al-Qura University, Saudi-Arabia<br>Age mean (SD):<br>Posttest: 22.03 (1.34) (Range 20-25)<br>Gender:<br>Posttest: 61.9% female, 38.1% male	Infection control training course based on the WHO guide for infection control included:<br>Blenden delivery methods: Mini-lecture, practical training sessions, demonstration by trainers, performance in a group and individual with feedback<br>Duration:<br>1x/week for 6 consecutive weeks	**Primary:**<br>(a) COVID-19 specific knowledge on infection prevention control measures<br>(b) COVID-19 specific infection preventions performance skills	**HL indicator:**<br>(a) Knowledge<br>(b) Perfoemance skills<br>**HL component:**<br>(a) Understand<br>(b) Apply	(a) Questionnaire, (participant-reported, score-based, self-developed based on literature (not further specified)<br>(b) Checklist with three sections (assessed through reviewers)<br>(i) 10 steps of correct hand hygiene,<br>(ii) 5 steps of putting on PPE,<br>(iii) 5 steps of putting off PPE<br>Internal consistency: Reliability Cronbach α=0.82	F1: Six months after intervention	None reported	None declared
**Sabandüzen et al. 2024** [103] **Turkey**	Dec 2021 - Feb 2022	158 undergraduate nursing students at the nursing department of the faculty of health sciences in a private university in Turkey<br>Age mean (SD): 20.6y (1.69) (Range 18-28)<br>Gender: 72.8% female, 27.2% male	Training on home care of infected or suspected COVID-19 patients<br>Duration: 80 min	**Secondary:** *Composite outcome* General and COVID-19-related knowledge on transmission and home care of infected or suspected COVID-19 patients	**HL indicator:** Knowledge<br>**HL component:** Understand	10 multiple-choice questions (5 options each); developed by the researchers and reviewed by two experts; based on several references [103]; participant-reported	F1: One week after intervention	No funding	None declared
**Said et al. 2021** [104] **Saudi-Arabia**	Jun - Sep 2020	70 staff nurses from General Farasan Hospital, Saudi Arabia<br>Age mean (SD): 27.34y (5.44)<br>< 20y: 8.6%<br>20-30y: 74.3%<br>> 31y: 17.1%	Educational intervention<br>Duration: 16-weeks with 4 scheduled sessions; 30-60 min / sess.<br>Methods: modified lecture, group discussion, power point and brainstorming	**Secondary:** *Composite score*<br>(a) General and COVID-19-related knowledge on nursing care and healthy life style	**HL indicator:**<br>(a) Knowledge (composite)<br>(b) Performance skills (composite)<br>**HL component:**	A structured self-administered questionnaire; close and open-ended questions<br>(a) 12 items; total score of 24; Each item scored	F1: Post-intervention (not specified)	Jazan University	None declared

Study (Author, Year) Country	Period of time of data collection	Population	Intervention	Outcomes				Sponsor-ship Source	Conflicts of Interest
		Participants description, Sample size (total), Setting		Outcome	Relation to health literacy (HL)	Outcome measure	Timing of outcome measurement		
**Study Design: Uncontrolled Before-After Studies**									
			Materials: educational booklet, audio-visual aids, videos	(b) COVID-19 performance skills on COVID-19 patient care and infection protection	(a) Understand (composite)<br>(b) Apply (composite)	2 (correct) or 0 (incorrect/do not know)<br>(b) 20 items; 3-point scale (1 'not done', 2 'incompeten done', 3 'competent done')<br>Designed by the researcher; Content validity was conducted<br>(a) Reliability Cronbach *α*=0.92<br>(b) Reliability Cronbach *α*=0.87			
**Salehi et al. 2023** [105] **Ghana**	E-learning: Oct 26th 2021 – Feb 22nd 2022<br>In-person: Dec 29th 2021 - Apr 2nd 2022	9,966 health workers including nurses, midwives, physicians from the Ghana College of Nurses and Midwives and the SickKids Centre for Global Child Health<br>Age (in %)[a]<br><24y: 1.8%<br>25-34y. 73.2%<br>35-44y. 18.8%<br>45-54y. 1.1%<br>55+y: 0.5%<br>Gender: 71.2% female, 28.1% male	E-learning & in-person courses combined<br>Three e-learning courses included narrative descriptions, interactive activities, and case studies<br>One in-person course included multi-modal teaching and learning strategies (e. g. skills stations, role play, case presentations)<br>Duration: Two days (in-person)	**Secondary:**<br>*Composite outcome*<br>COVID-19 related knowledge on prevention and health promotion	**HL indicator:**<br>Knowledge<br>**HL component:**<br>Understand	Questionnaire (multiple-choice; participant-reported; score-based, range 0-100)	F1:<br>Directly after each intervention	Government of Canada (Global Affairs Canada) with in-kind contributions from the Ghana	None declared
**Sangwan et al. 2022** [106] **India**	Apr - Oct 2020	560 frontline health professionals (Senior Residents, Junior Residents, Interns, nursing staff) in COVID-19 hospital of Shaheed Hasan Khan Mewati Government Medical College<br>Age (mean): 30.5y	15 training sessions included live demonstration of PPE use and hand hygiene steps by trained staff under supervision of the Infection Control Officer	**Primary:**<br>COVID-19 specific knowledge on infection prevention control measures	**HL indicator:**<br>Knowledge<br>**HL component:**<br>Understand	Questionnaire (12 out of 21 items referred to the primary outcome; multiple-choice; participant-reported)<br>Pre-validation based on Thomas et al. 2015 [107]	F1:<br>Directly after intervention	None reported	None declared
**Shahrin et al. 2022** [108] **Bangladesh**	Jul 1st 2020 - Jun 30th 2021	755 health care workers (physicians, nurses clinical support staff) from four district-level hospitals and one specialized hospital;<br>Age mean (SD):	Modules on IPC and case management modules<br>Training curriculum and materials differed for doctors and nurses	**Primary:**<br>COVID-19 specific knowledge on	**HL indicator:**<br>(a-d) Knowledge<br>**HL component:**<br>(a-d) Understand	(a & b) Questionnaire (semi-structured, multiple-choice, 15 items; Six out of 20 items referred to the primary outcome; score-based	F1:<br>Post-intervention<br>(not specified)	Collaboration Government People's Republic of Bangladesh and the development	None declared

Study (Author, Year) Country	Period of time of data collection	Population	Intervention	Outcomes				Sponsorship Source	Conflicts of Interest
		Participants description, Sample size (total), Setting		Outcome	Relation to health literacy (HL)	Outcome measure	Timing of outcome measurement		
**Study Design: Uncontrolled Before-After Studies**									
		Doctors: 32.82y (4.99)<br>Nurses: 35.46y (8.31)<br>Gender: 71.7% female	Blended delivery methods: theoretical sessions, practical demonstrations and interactive discussions<br>Duration: Two-day session (for doctors); one-day session (for nurses and other support staff members)	(a) on infection prevention control measures<br>(b) of epidemiology<br>(c) of clinical presentation of SARS-CoV-2<br>(d) on infection management		(0-5: poor, 6-10: average, 11-15: fair), participant-reported)<br>Validated by trainee physicians of Dhaka Hospital		partners. This entire project has been funded by the Global Affairs of Canada (Gr-01686).	
**Sharma et al. 2021** [109]<br>**India**	April - May 2020	968 health care workers (nursing officers, technicians, and residents) working at 900 bedded tertiary care institute in the Northern part of India<br>Age mean (SD): 27.14y (3.07)<br>Gender: 44.9% female, 55.1% male	Virtual live & video-assisted IPC group training (via Zoom®)<br>Duration: 1 hr training per group (20-25 participants)	**Primary:**<br>COVID-19 specific knowledge on infection prevention control measures (participant-reported)	**HL indicator:**<br>Knowledge<br>**HL component:**<br>Understand	Questionnaire (semi-structured, score-based) included five sections (Do's and Don'ts, Hand Hygiene, PPE, cleaning and disinfection, and bio-medical waste management) through Google Questionnaire form presented	F1:<br>Directly after intervention	No funding	None declared
**Singh et al. 2021** [110]<br>**India**	April - May 2020	478 faculty members (not specified), senior and junior residents, MBBS students/interns, and nursing officers working at tertiary care institute in the northern part of India; mixed population<br>Age mean (SD): 27.65y (3.73)<br>Gender: 43.7% female, 56.3% male	Video-assisted IPC training modules<br>Four in-house prepared video modules (about do's & don'ts, hand hygiene, donning and doffing of PPE and disinfection) were demonstrated in training sessions conducted for group of 15–20 participants at a time;<br>Training was conducted in assistance of trainers<br>Duration: Each training lasted 2 h, 7-9min. / each video<br>Language: Hindi	**Primary:**<br>COVID-19 specific knowledge on infection prevention control measures	**HL indicator:**<br>Knowledge<br>**HL component:**<br>Understand	Questionnaire (semi-structured, score-based, participant-reported) included five sections (Do's and Don'ts, Hand Hygiene, PPE, cleaning and disinfection, and biomedical waste management) through Google Questionnaire form	F1:<br>Directly after intervention	Nil	None declared
**Smith et al. 2023** [111]<br>**United States**	Group 1: Jun 2020<br>Group 2: Jun 2020 - Feb 2021	54 house staff who were to provide care throughout the hospital, including in the operating rooms (Anesthesiology interns and first year clinical anesthesia residents)	Miller's pyramid-based training on PPE use with simulation of COVID-19 contamination through fluorescent marker<br>Consisted of multiple steps:	**Primary:**<br>PPE performance (donning & doffing)	**HL indicator:**<br>Performance skills<br>**HL component:**<br>Apply	Checklists rated by an independent observer blinded to education training video filming;	F1:<br>Directly after the intervention<br>F2:	None declared	None declared

Study<br>(Author, Year)<br>Country	Period of time of data collection	Population	Intervention	Outcomes				Sponsor-ship Source	Conflicts of Interest
		Participants description,<br>Sample size (total), Setting		Outcome	Relation to health literacy (HL)	Outcome measure	Timing of outcome measurement		
**Study Design: Uncontrolled Before-After Studies**									
		Gender: 37% female, 63% male	(1) Pre-intervention: contamination simulation before and after donning & doffing sequence and recording body areas under black-light with verbal feedback by instructors<br>(2) Watching pre-recorded video of PPE correct steps produced by an infectious disease physicians expert from infection control reinforced with face-to-face demonstration<br>(3) Post-intervention: repetition of (1) without assistance			the number of contaminated sites was noted	Eight month after intervention		
**Strehlow et al. 2024** [112]<br>**United States**	Jul 17th - Sep 24th 2020	30,859 learners, predominantly healthcare workers, enrolled in the course; mixed health professional occupation population<br>Age (10,323 participants)<br>18-39y: 71%<br>40-59y: 24%<br>≥ 60y: 5%<br>Gender (10,587 participants):<br>58% female, 41% male, 1% other	Massive opne online course;<br>15 short (≤10 min) video-based lectures in 6 modules; Online via Coursera, EdX platforms and Digital Medic mobile app<br>Duration: ~3 hours	**Secondary:**<br>*Composite outcome*<br>COVID-19 knowledge on PPE, clinical assessment, diagnostic and treatment	**HL indicator:**<br>Knowledge (composite)<br>**HL component:**<br>Understand (composite)	60 items (module quizzes: 4 per module) + 20 items (final exam); multiple-choice (1 'correct', 0 'incorrect'); participant-reported	F1:<br>Directly after each intervention<br>F2:<br>Post-intervention (not specified)	No funding	None declared
**Ta'an et al. 2023** [113]<br>**Jordan**	Apr 1st - Dec 20th 2021	87 health professionals in one of the four field hospitals in Jordan<br>Age mean (SD): 41.53y (6.34)<br>Gender: 51.7% female, 48.3% male	Competency Outcomes and Performance Assessment based training program<br>Delivered in sessions for 20 participants per group (5 groups in total)<br>Duration: 5hrs (2 sessions per group × 2,5 hrs./session)	**Secondary:**<br>(a) Perceived COVID-19 competencies on hand and respiratory hygiene<br>(b) Perceived COVID-19 competencies in using PPE<br>(c) Perceived competencies in COVID-19 specific waste management	**HL-related factor:**<br>(a-c) Confidence (self-efficacy)	(a) Self-reported competency checklist;<br>18 items, 5-point Likert Scale ranging from 'strongly disagree' to 'strong agreement'; Developed by the research team members;<br>Face and content validity checked by team members<br>Internal consistency: 0.79	F1:<br>Directly after intervention	Jordan University of Science and Technology-Deanship of Scientific Research (53/139/2021)	None declared

Study (Author, Year) Country	Period of time of data collection	Population	Intervention	Outcomes				Sponsor-ship Source	Conflicts of Interest
		Participants description, Sample size (total), Setting		Outcome	Relation to health literacy (HL)	Outcome measure	Timing of outcome measurement		
**Study Design: Uncontrolled Before-After Studies**									
**Tan et al. 2020** [114] **China**	-	301 doctors, nurses, and other personnel (pharmacists, technicians, researchers) at the tertiary hospital in Beijing, China; clinical skill training center of the hospital has simulated wards specialized for infectious disease and complete sets of training equipment.<br>Gender: 91.7% female	Emergency training program of PPE<br>Combined delivery methods as a three-stage training program:<br>(i) Lecture and demonstration (face-to-face)<br>(ii) Simulation exercise (observed by the teachers)<br>(iii) Test and evaluation	**Primary**<br>COVID-19 related infection prevention control performance skills	**HL indicator:**<br>Performance skills<br>**HL component:**<br>Apply	Checklist (40 items, score-based 0-100, pass line score of 90; assessed by teachers)<br>Assessment criteria were developed by the clinical skill training center	F1:<br>Directly after intervention	Supported by the Fifth Medical Center of Chinese PLA General Hospital. The authors did not receive any commercial supports.	None declared
**Thakre et al. 2020a** [115] **India**	May 21st - May 30th 2020	232 nurses working in outpatient departments, wards and laboratory services specifically dedicated to COVID-19 at a tertiary health care centre/hospital<br>Age mean (SD): 38.95y (4.5)<br>Gender: 90% female	COVID-19 training program with lectures<br>In-person lectures (epidemiology, microbiology, prevention) consisted of 10 batches á 20-25 participants<br>Duration: 1 d	**Primary:**<br>COVID-19 specific knowledge of<br>(a) Epidemiology<br>(b) Infection prevention control measures<br>(c) Microbiology<br>**Secondary:**<br>*Composite Outcome*<br>(d) COVID-19 related knowledge on epidemiology, prevention and microbiology	**HL indicator:**<br>(a-c) Knowledge<br>(d) Knowledge (composite)<br>**HL component:**<br>(a-c) Understand<br>(d) Understand (composite)	Predesigned validated 20-item questionnaire (open-ended, score-based, participant-reported);<br>Reliability: based on Kappa analysis for each item, were conducted by three subject; agreement value: 65%	F1:<br>Post-intervention (not specified)	No funding	None declared
**Thakre et al. 2020b** [116] **India**	March 26th - April 24th 2020	486 health care workers such as professors, associate professors, assistant professors, residents and interns at a tertiary health care institution in Nagpur; and medical officers of public health	COVID-19 training program with lectures and demonstrations<br>In-person combined delivery mode:<br>(I) lectures (epidemiology, microbiology, prevention & treatment) and<br>(ii) demonstrations (intubation)<br>Duration: 1 d	**Primary:**<br>COVID-19 specific knowledge of<br>(a) Epidemiology<br>(b) Infection prevention control measures<br>(c) Microbiology<br>**Secondary:**<br>*Composite Outcome*<br>(d) COVID-19 related knowledge on epidemiology, prevention,	**HL indicator:**<br>(a-c) Knowledge<br>(d) Knowledge (composite)<br>**HL component:**<br>(a-c) Understand<br>(d) Understand (composite)	Predesigned and validated 30-item questionnaire (score-based, participant-reported)	F1:<br>Directly after intervention	No funding	None declared

Study (Author, Year) Country	Period of time of data collection	Population	Intervention	Outcomes				Sponsor-ship Source	Conflicts of Interest
		Participants description, Sample size (total), Setting		Outcome	Relation to health literacy (HL)	Outcome measure	Timing of outcome measurement		
**Study Design: Uncontrolled Before-After Studies**									
				treatment, microbiol-ogy and stages of COVID-19					
**Tsiouris et al. 2022** [117] **Worldwide**	June - October 2020	8,797 physicians, nurses, other health fa-cility-based staff, community health work-ers, health managers and public health practitioners working in health facilities, where COVID-19 cases were expected in 11 countries; And non-clinical health worker, those not affiliated with health fa-cilities included staff at district or regional health offices, community health care workers, and staff from non-governmental organizations, and ministries of health<br>Country/Regions from which participants were recruited: Africa (Angola, Burundi, Eswatini, Kenya, Lesotho, Malawi, Mozambique, Rwanda, Sierra Leone, South Sudan, and Zambia)<br>Gender: 57% female, 43% male	Emergency COVID-19 training<br>Blended delivery mode: In-person, virtual (synchronous, via Zoom® or asynchronous using PowerPoint on-demand or the Articulate™ online training platform or hybrid (10 modules)<br>Blenden delivery methods: lec-tures, case studies, knowledge-check questions, simulation of PPE donning & doffing)<br>Duration: 1-8 d (median: 3 d)	**Secondary:**<br>*Composite outcome*<br>COVID-19 practical knowledge and skills relating to infection prevention measures	**HL indicator:**<br>Mixed composite out-come based on the knowledge and perfor-mance skills<br>**HL component:**<br>Understand and apply (composite)	25-items online post-training test survey us-ing Qualtrics™; partici-pant-reported; trans-lated into French and Portuguese; Number of questions differs be-tween regions; score-based (range 24-40), passing score of 70%)	F1:<br>Directly after intervention<br>F2:<br>Directly after intervention	No finding	None declared
**Wang et al. 2022** [118] **Ethiopia and United States**	May-Jun 2022	77 surveillance officers in nine regions<br>Age mean (SD): 27y (4.25)<br>Gender: 13% female, 87% male	Virtual COVID-19 training and field deployment<br>Module content: background on SARS-CoV-2, contact investiga-tion, and communications<br>Delivery mode: Virtual via Zoom®<br>Delivery method: Q&A, didactic and demonstration sessions<br>Developed by experts from EPHI, CDC Ethiopia, CDC USA, OSU, and John Snow, Inc.<br>Duration: 3 days (8.5 hrs/day)	**Primary:**<br>COVID-19 specific knowledge on<br>(a) infection preven-tion control measures<br>(b) virus transmission	**HL indicator:**<br>(a,b) Knowledge<br>**HL component:**<br>(a, b) Understand	Post-tests using online Qualtrics™ survey soft-ware; participant-re-ported	F1:<br>Post-interven-tion (not speci-fied)	CDC Foun-dation	None declared
**Wu et al. 2023** [119] **United States**	Nov 2021–Jan 2022	18 community health navigators repre-senting Asian American, Arab American, Black/African American, and His-panic/Latinx communities<br>Age<br>21-30y: 33.33%	'Community Health Navigator Training on COVID-19: Vaccina-tion, Prevention, and Contact Trac-ing (CHN)'<br>Training curriculum with 6 topics:<br>(i) COVID-19, (ii) quarantine, isola-tion, testing, treatment, contact	**Primary:**<br>(a) General COVID-19 related knowledge<br>**Secondary:**<br>(b) Confidence in edu-cating community members regarding	**HL indicator:**<br>(a) Knowledge<br>**HL component:**<br>(a) Understand<br>**HL-related factors:**	(a & b) Questionnaires<br>(a) 9 items with true/false response op-tions, 11 multiple-choice response op-tions; participant-re-ported	F1:<br>One week after intervention	U.S. Depart-ment of Health and Human Ser-vices, Ad-vancing Health Liter-acy (AHL) grant, #1	None declared

Study (Author, Year) Country	Period of time of data collection	Population	Intervention	Outcomes				Sponsor-ship Source	Conflicts of Interest
		Participants description, Sample size (total), Setting		Outcome	Relation to health literacy (HL)	Outcome measure	Timing of outcome measurement		
**Study Design: Uncontrolled Before-After Studies**									
		31-40y: 33.33%<br>41-50y: 11.11%<br>51-60y: 16.67%<br>≥ 61y: 5.56%<br>Gender: 94.44% female, 5.56% male	tracing, (iii) COVID-19 prevention/protection, (iv) COVID-19 vaccination,<br>(v) COVID-19 vaccination during pregnancy and breastfeeding and other special populations, (vi) health literacy and communication strategies for misinformation and vaccine hesitancy<br>Delivery mode: virtual (synchronous via Zoom® or asynchronous recorded session on-demand)<br>Delivery method: role-plays with scenarios, not further specified<br>Duration: 3.5 hrs (60 min listening session/focus group, 2 hrs CHN training, 10-15 min each for pretest and post-test)	COVID-19 vaccination and prevention	(b) Confidence (self-efficacy)	(b) 5-point Likert scale; participant-reported		CPIMP21130 1-01-00	
**Zafar et al. 2020** [120]<br>**Pakistan**	-	400 frontline healthcare professionals (junior doctors, nurses and non-clinical hospital staff) from the three major government hospitals of Rawalpindi; Benazir Bhutto Hospital, Holy Family Hospital, and District Headquarters Hospital<br>Age mean (SD): 27.09y (3.7)<br>Gender: 52% female, 48% male	Written material (circulated through social media)<br>Material as reference guide and learning tool was extracted from CDC's 'interim infection prevention and control recommendations for COVID-19 in health care settings', the Government of Pakistan COVID-19 website for the local guidelines, and the WHO	**Primary:**<br>(a) COVID-19 specific knowledge of clinical presentation of SARS-CoV-2<br>(b) COVID-19 specific knowledge on infection prevention control measures<br>(i) Usage of sanitizer/ hand washing<br>(ii) Diagnostic testing<br>(iii) PPE performance (donning & doffing)	**HL indicator:**<br>(a-b) Knowledge<br>**HL component:**<br>(a-b) Understand	(a & b) Semi-structured questionnaire; 31 items; participant-reported; based on 'Centers for Disease Control and Prevention's checklist for healthcare personnel preparedness for transport and arrival of patients with confirmed or possible COVID-19' [121]; languages; Urdu, English; Five out of 22 reported results were related to eligible outcomes<br>Validity: reviewed by a consultant of Infectious Diseases Department of Holy Family Hospital	F1: Two weeks after intervention	None reported	None declared

Study (Author, Year) Country	Period of time of data collection	Population	Intervention	Outcomes				Sponsorship Source	Conflicts of Interest
		Participants description, Sample size (total), Setting		Outcome	Relation to health literacy (HL)	Outcome measure	Timing of outcome measurement		
**Study Design: Uncontrolled Before-After Studies**									
**Zhao et al. 2022** [122] **Canada**	Jul 2020 - Feb 2021	114 Health care worker (family medicine and specialist physicians, physician assistants, nurse practitioners, registered nurses, registered practical nurses, pharmacists, and other allied health professionals) in Ontario<br>Age<br>20–39y: 41.2%<br>40–59y: 35.1%<br>≥60y: 13.2%<br>Unknown: 10.5%<br>Gender 86.8% female	Telementoring education program within the 'Extension for Community Healthcare Outcomes (ECHO)' study program<br>Delivery method: didactic lecture and patient presentation; recording sessions for review afterwards<br>Based on available resources from the international ECHO (MetaECHO) community<br>Duration:<br>24hrs (24 weekly session x 1 hr/session)	**Secondary:**<br>*Composite outcome*<br>Perceived COVID-19 related self-efficacy, knowledge & confidence	Mixed composite outcome based on the knowledge and self-efficacy<br>**HL component:**<br>Understand (composite)<br>**HL-related factors:**<br>Confidence (self-efficacy, composite)	Questionnaire (participant-reported; 17-items; 7-point Likert Scale ranging from 'strongly disagree' to 'strongly agree', administered through Survey Monkey®<br>Adapted from previous ECHO program	F1:<br>Post-intervention (not specified)	Ontario Ministry of Health and Long-Term Care.	None declared

ECHO Extension for Community Healthcare Outcomes Program, **CDC** Centers for Disease Control and Prevention, **F** Follow-up, **IG** Intervention group, **IPC** Infection prevention and control, **PPE** Personal protective equipment, **SCFHS** Saudi Comission for Health Specialities, **VR** Virtual reality. [a] Frequencies (in %) were calculated by the review authours

References

1. Alotaibi N, Al-Sayegh N, Nadar M, Shayea A, Allafi A, Almari M. Investigation of Health Science Students' Knowledge Regarding Healthy Lifestyle Promotion During the Spread of COVID-19 Pandemic: A Randomized Controlled Trial. Frontiers in public health 2021;9:774678. doi:10.3389/fpubh.2021.774678
2. Amiri, B, Khajavian, N, Rahmani, R, Bilandi RR. Comparing the Impact of Multimedia and Educational Brochures on Knowledge, Attitude and Work Performance of Healthcare about COVID-19 Management in Pregnancy, Childbirth, and Breastfeeding. Iranian Red Crescent Medical Journal 2023;25(11). doi:10.32592/ircmj.2023.25.11.2498
3. Birrenbach T, Zbinden J, Papagiannakis G, Exadaktylos AK, Muller M, Hautz WE, Sauter TC. Effectiveness and Utility of Virtual Reality Simulation as an Educational Tool for Safe Performance of COVID-19 Diagnostics: Prospective, Randomized Pilot Trial. JMIR serious games 2021;9(4):e29586. doi:10.2196/29586
4. Kennedy RS, Lane NE, Berbaum KS, Lilienthal MG. Simulator Sickness Questionnaire: An Enhanced Method for Quantifying Simulator Sickness. The International Journal of Aviation Psychology 1993;3(3):203-220. doi:10.1207/s15327108ijap0303_3
5. Christensen L, Rasmussen CS, Benfield T, Franc JM. A Randomized Trial of Instructor-Led Training Versus Video Lesson in Training Health Care Providers in Proper Donning and Doffing of Personal Protective Equipment. Disaster Medicine and Public Health Preparedness 2020;14(4):514-520. doi:10.1017/dmp.2020.56
6. Currat L, Suppan M, Gartner BA, Daniel E, Mayoraz M, Harbarth S, Suppan L, Stuby L. Impact of Face-to-Face Teaching in Addition to Electronic Learning on Personal Protective Equipment Doffing Proficiency in Student Paramedics: Randomized Controlled Trial. International journal of environmental research and public health 2022;19(5). doi:10.3390/ijerph19053077
7. Jafree SR, Zakar R, Rafiq N, Javed A, Durrani RR, Burhan SK, Hasnain Nadir SM, Ali F, Shahid A, Momina AU, Wrona KJ, Mahmood QK, Fischer F. WhatsApp-Delivered Intervention for Continued Learning for Nurses in Pakistan During the COVID-19 Pandemic: Results of a Randomized-Controlled Trial. Frontiers in public health 2022;10:739761. doi:10.3389/fpubh.2022.739761
8. Jeihooni AK, Namdari A, Kashfi SM, Kamyab A, Harsini PA, Rakhshani T. Effects of an educational intervention based on the health belief model on COVID-19 preventive behaviors among health personnel in Abadan, Iran. Journal of Public Health 2023. doi:10.1007/s10389-023-02053-6
9. Li Y, Wang Y, Li Y, Zhong M, Liu H, Wu C, Gao X, xia Z, Ma W. Comparison of Repeated Video Display vs Combined Video Display and Live Demonstration as Training Methods to Healthcare Providers for Donning and Doffing Personal Protective Equipment: A Randomized Controlled Trial. Risk Management and Healthcare Policy 2020;13:2325-2335. doi:10.2147/RMHP.S267514
10. Manggala SK, Tantri AR, Sugiarto A, Sianipar IR, Prasetyono TOH. In situ simulation training for a better interprofessional team performance in transferring critically ill patients with COVID-19: a prospective randomised control trial. Postgraduate medical journal 2022. doi:10.1136/postgradmedj-2021-141426
11. Rakhshani T, Dolatkhah SM, Kashfi SM, Khani Jeihooni A. The effect of a self-learned virtual learning package on knowledge, attitude, and self-care behaviors of COVID-19 in people referred to health and treatment centers. BMC Public Health 2024;24(1):1710. doi:10.1186/s12889-024-19233-y
12. Rueda-Medina B, Aguilar-Ferrándiz ME, Esteban-Burgos AA, Tapia Haro RM, Casas-Barragán A, Velando-Soriano A, Gil-Gutiérrez R, Correa-Rodríguez M. Impact of Non-Face-to-Face Teaching with Passive Training on Personal Protective Equipment Use in Health Science Students: A Randomized Controlled Trial. International journal of environmental research and public health 2022;19(19). doi:10.3390/ijerph191912981
13. Salway RJ, Williams T, Londono C, Roblin P, Koenig K, Arquilla B. Comparing Training Techniques in Personal Protective Equipment Use. Prehospital and Disaster Medicine 2020;35(4):364-371. PMID:32390583
14. Suppan L, Abbas M, Stuby L, Cottet P, Larribau R, Golay E, Iten A, Harbarth S, Gartner B, Suppan M. Effect of an E-Learning Module on Personal Protective Equipment Proficiency Among Prehospital Personnel: Web-Based Randomized Controlled Trial. Journal of medical Internet research 2020;22(8):e21265. doi:10.2196/21265
15. Suppan L, Stuby L, Gartner B, Larribau R, Iten A, Abbas M, Harbarth S, Suppan M. Impact of an e-learning module on personal protective equipment knowledge in student paramedics: a randomized controlled trial. Antimicrobial Resistance & Infection Control 2020;9(1):185. doi:10.1186/s13756-020-00849-9
16. Wang XX, Zhou YZ, Song ZX, Wang YT, Chen XT, Zhang DD. Practical COVID-19 Prevention Training for Obstetrics and Gynecology Residents Based on the Conceive-Design-Implement-Operate Framework. Frontiers in public health 2022;10. doi:10.3389/fpubh.2022.808084
17. Xie M, Zhou Q, Kang Y, Qing P, Guo Y, Wei X, Cai B, Zeng J, Huang J. The Skill Training of Resident Anesthesiologists During the Outbreak Of COVID-19. ResearchSquare 2021. doi:10.21203/rs.3.rs-144144/v1
18. Ansari A., Urooj U., Waseem M., Ihtasham A. Video based learning vs instructor led training for optimising personal protective equipment use to prevent Covid-19 infection-a comparative study. J Pak Med Assoc 2022;72(5):807-810. doi:10.47391/JPMA.3359
19. Buyego P, Katwesigye E, Kebirungi G, Nsubuga M, Nakyejwe S, Cruz P, McCarthy MC, Hurt D, Kambugu A, Arinaitwe JW, Ssekabira U, Jjingo D. Feasibility of virtual reality based training for optimising COVID-19 case handling in Uganda. BMC Medical Education 2022;22(1):274. doi:10.1186/s12909-022-03294-x
20. Hu H, Xiao YY, Li H. The Effectiveness of a Serious Game Versus Online Lectures for Improving Medical Students' Coronavirus Disease 2019 Knowledge. Games For Health Journal 2021;10(2):139-144. doi:10.1089/g4h.2020.0140
21. Yu M, Yang M. Effectiveness and Utility of Virtual Reality Infection Control Simulation (VRICS) for Children with COVID-19: A quasi experimental study. JMIR serious games 2022. doi:10.2196/36707
22. Choi J. Nurse's Knowledge, Attitude and Use of Personal Protective Equipment Related to Acute Respiratory Infections (master's thesis). Seoul; 2016.
23. Kwon M. Knowledge, Perception, and Performance Related to Personal Protective Equipment among Nurses in Specialized Infectious Disease Hospitals (master's thesis). Pusan; 2021.
24. Ayres H. Factors Related to Motivation to Learn and Motivation to Transfer Learning in a Nursing Population (dissertation). North Carolina; 2005.
25. Abbas K, Nawaz SMA, Amin N, Soomro FM, Abid K, Ahmed M, Sayeed KA, Ghazanfar S, Qureshi N. A web-based health education module and its impact on the preventive practices of health-care workers during the COVID-19 pandemic. Health Education Research 2020;35(5):353-361. doi:10.1093/her/cyaa034

26. Ahmed NH, Tosson MM, Badia TS. Effect of educational program on maternity nurses' knowledge, attitude and practice of preventive measures towards COVID-19. Assiut Scientific Nursing Journal 2022;0(0):0. doi:10.21608/asnj.2022.116049.1301
27. Mohammed AS. Knowledge, Attitudes, and Practices towards COVID-19 among Health Care Workers in Primary Health Care Units Dar El Salam, Suhag, Egypt. Sohag Medical Journal 2021;25(1):50-58. doi:10.21608/smj.2020.47286.1209
28. Nwagbara UI, Osual EC, Chireshe R, Bolarinwa OA, Saeed BQ, Khuzwayo N, Hlongwana KW. Knowledge, attitude, perception, and preventative practices towards COVID-19 in sub-Saharan Africa: A scoping review. PLOS ONE 2021;16(4):e0249853. PMID:33872330
29. Altillo BSA, Gray M, Avashia SB, Norwood A, Nelson EA, Johnston C, Bhavnani D, Patel H, Allen CH, Adeni S, Phelps ND, Mercer T. Global health on the front lines: an innovative medical student elective combining education and service during the COVID-19 pandemic. BMC Medical Education 2021;21(1):186. doi:10.1186/s12909-021-02616-9
30. Aqel O, Alqadheeb B, Felix M, Amundson C, Bingham JM, Meyer K, Warholak T, Axon DR. Cultivating COVID-19 Vaccine Confidence in Pharmacy Professionals. Pharmacy 2023;11(2). doi:10.3390/pharmacy11020050
31. Aujee D.S., Aghamkar J., Yangad S., Salvi R. "Assessment of Educational Intervention On Knowledge Regarding World Health Organization Covid-19 Protocols During Delivery AmongStaff Nurses Working In Labour Room of Selected Hospitals. Journal of Pharmaceutical Negative results 2022;13:4717-4721. doi:10.47750/pnr.2022.13.S08.613
32. Bakhsh A, Asiri R, Alotaibi H, Alsaeedi R, Shahbar R, Boker A. Rapid cycle training for non-critical care physicians to meet intensive care unit staff shortage at an academic training center in a developing country during the COVID-19 pandemic. BMC Medical Education 2023;23(1):493. doi:10.1186/s12909-023-04478-9
33. Bayomi R, taha N. Effect of Teaching Guidelines on Knowledge, Attitudes, and Practices Regarding COVID19 among the First Year Nursing Students. Assiut Scientific Nursing Journal 2021;9(25):38-47. doi:10.21608/asnj.2021.72355.1156
34. Ministry of Health Guidelines. Guidelines on Prevention and Control COVID19 2020 URL: https://www.health.go.ug/covid/project/guidelines/.
35. World Health Organization. Rational use of personal protective equipment for coronavirus disease (COVID-19) and considerations during severe shortages: interim guidance 2020 URL: https://apps.who.int/iris/bitstream/handle/ 10665/331215/WHO-2019-nCov-IPCPPE use-2020.1-eng pdf.
36. Nassef H. Knowledge, Attiudes, and Practices about Coronavirus disease (COVID-19) among Birzeit University Students: A cross-sectional study. Research Square 2020 URL: http://doi.org/10.21203/65.3.rs-83268.
37. Bechini A, Vannacci A, Salvati C, Crescioli G, Lombardi N, Chiesi F, Shtylla J, Del Riccio M, Bonanni P, Boccalini S. Knowledge and training of Italian students in Healthcare Settings on COVID-19 vaccines and vaccination strategies, one year after the immunization campaign. Journal of preventive medicine and hygiene 2023;64(2):E152-E160. doi:10.15167/2421-4248/jpmh2023.64.2.2934
38. Bieri J, Tuor C, Nendaz M, L Savoldelli G, Blondon K, Schiffer E, Zamberg I. Implementation of a Student-Teacher-Based Blended Curriculum for the Training of Medical Students for Nasopharyngeal Swab and Intramuscular Injection: Mixed Methods Pre-Post and Satisfaction Surveys. JMIR Medical Education 2023;9:e38870. doi:10.2196/38870
39. Blake H, Fecowycz A, Starbuck H, Jones W. COVID-19 Vaccine Education (CoVE) for Health and Care Workers to Facilitate Global Promotion of the COVID-19 Vaccines. International journal of environmental research and public health 2022;19(2). doi:10.3390/ijerph19020653
40. Wharrad HJ, Morales R, Windle R, Bradley CA. A toolkit for a multilayered, cross-institutional evaluation strategy. In: World Conference on Educational Multimedia, Hypermedia and Telecommunications. Chesapeake, WV, USA: Association for the Advancement of Computing in Education (AACE); 2008.
41. Boccalini S, Vannacci A, Crescioli G, Lombardi N, Del Riccio M, Albora G, Shtylla J, Masoni M, Guelfi MR, Bonanni P, Bechini A. Knowledge of University Students in Health Care Settings on Vaccines and Vaccinations Strategies: Impact Evaluation of a Specific Educational Training Course during the COVID-19 Pandemic Period in Italy. Vaccines 2022;10(7). doi:10.3390/vaccines10071085
42. Bohara A, Thapa S, Yilmaz SK, McBee SH. An Impact Evaluation of COVID-19 Training Program: Knowledge and Awareness of Public Health Professionals of Province Five, Nepal; 2021.
43. Brito-Brito PR, Fernandez-Gutierrez DA, Martinez-Alberto CE, Saez-Rodriguez MJ, Nunez-Marrero J, Garcia-Hernandez AM. Use of the Nursing Outcomes Classification (NOC) to measure perceived knowledge about the control of SARS-CoV-2 infection: The impact of a training program in primary healthcare professionals. International journal of nursing knowledge 2021. doi:10.1111/2047-3095.12356
44. Moorhead S., Swanson E., Johnson M., Maas M. Nursing Outcome Classification (NOC) (6th ed.): Measurement of Health Outcomes. Oxford: Elsevier Health Sciences; 2018. ISBN:9780323497640.
45. Calik A, Cakmak B, Kapucu S, Inkaya B. The effectiveness of serious games designed for infection prevention and promotion of safe behaviors of senior nursing students during the COVID-19 pandemic. American journal of infection control 2022. doi:10.1016/j.ajic.2022.02.025
46. Clay J, Morton K, Franz D, Jaqua E, Nguyen V. Quality Improvement for Outpatient COVID-19 Infection Control. Cureus 2021;13(7):e16373. doi:10.7759/cureus.16373
47. Díaz-Guio DA, Ricardo-Zapata A, Ospina-Velez J, Gómez-Candamil G, Mora-Martinez S, Rodriguez-Morales AJ. Cognitive load and performance of health care professionals in donning and doffing PPE before and after a simulation-based educational intervention and its implications during the COVID-19 pandemic for biosafety. Le Infezioni in Medicina 2020:111-117.
48. Elasrag GAEA, Elsabagh NE, Abdelmonem AF, Ahmed A. Impact of Educational Intervention on Nurses' Knowledge, Practice and Attitude Related Prevention Measures of COVID 19. IJFMT 2021. doi:10.37506/ijfmt.v15i3.15751
49. Gharpure R, Hunter CM, Schnall AH, Barrett CE, Kirby AE, Kunz J, Berling K, Mercante JW, Murphy JL, Garcia-Williams AG. Knowledge and Practices Regarding Safe Household Cleaning and Disinfection for COVID-19 Prevention - United States, May 2020. MMWR. Morbidity and Mortality Weekly Report 2020;69(23):705-709. PMID:32525852
50. McIntosh K, Hirsch MS, Bloom A. Coronavirus disease 2019 (COVID-19): Epidemiology, virology, and prevention. The Lancet Infectious Diseases 2020;1:2019-2020.
51. Salman M, Mustafa Z, Asif N, Zaidi HA, Shehzadi N, Khan TM, Saleem Z, Hussain K. Knowledge, attitude and preventive practices related to COVID-19 among health professionals of Punjab province of Pakistan. The Journal of Infection in Developing Countries 2020;14(7):707-712. PMID:32794458
52. Espinoza-Castro B, Encina V, Garrido MA, Vinueza FI, Piedra JP, Garzon-Villalba X, Radon K. Online learning for crisis response: evaluating reach and perceived knowledge gains from the MOOC "Infection, Prevention, and Control of Acute Respiratory Infections

for Healthcare Workers in Low- and Middle-Income Countries (IPC MOOC)". BMC Medical Education 2025;25(1):1150. doi:10.1186/s12909-025-07661-2
53. Etebarian A, Tusi SK, Momeni Z, Hejazi K. Impact of educational intervention regarding COVID-19 on knowledge, attitude, and practice of students before dental school re-opening. BMC Oral Health 2023;23(1):1-6. doi:10.1186/s12903-023-02845-y
54. Fadel EA, Alshawish E, El-Shaboury RHR, Khalil DE, Mahmoud FZ, El-Feshawy NI. Effect of Implementing Virtual Educational Sessions on Nursing Students' Knowledge, Attitude and Hesitancy Regarding COVID-19 Vaccination. Inquiry: a journal of medical care organization, provision and financing 2025;62:469580251339114. doi:10.1177/00469580251339114
55. Martin LR, Petrie KJ. Understanding the Dimensions of Anti-Vaccination Attitudes: the Vaccination Attitudes Examination (VAX) Scale. Ann Behav Med 2017;51(5):652-660. PMID:28255934
56. Findyartini A, Greviana N, Hanum C, Husin JM, Sudarsono NC, Krisnamurti DGB, Rahadiani P. Supporting newly graduated medical doctors in managing COVID-19: An evaluation of a Massive Open Online Course in a limited-resource setting. PLOS ONE 2021;16(9). doi:10.1371/journal.pone.0257039
57. Fuentes GM, Carbajales León EB, Carbajales León AI. Educative intervention about COVID-19 in the Medicine students from Joaquín de Agüero Policlinic. Revista Electrónica Medimay 2020;27(3):366-376.
58. Garcia KS, Rodriguez A, Gonzalez Z, Armstrong C, Iacob E, Flynn EE, Simmons M. Pretest-post-test evaluation with lay midwives in remote Guatemala after educational activities about COVID-19. Rural and Remote Health 2024;24(3):8387. doi:10.22605/RRH8387
59. Girard H., Bosshard W., Krief H., Bula C.J. AO - Bula, Christophe J. Effectiveness of Information Sessions About COVID-19 Vaccines in Healthcare Professionals Working in Geriatrics. Gerontology and Geriatric Medicine 2022;8. doi:10.1177/23337214221115235
60. Greaves SW, Alter SM, Ahmed RA, Hughes KE, Doos D, Clayton LM, Solano JJ, Echeverri S, Shih RD, Hughes PG. A Simulation-based PPE orientation training curriculum for novice physicians. Infection Prevention in Practice 2023;5(1). doi:10.1016/j.infpip.2022.100265
61. Gupta S, Goswami B, Madhu SV. Effectiveness of Video-Based Educational Intervention on Knowledge, Attitude, and Practice (KAP) of COVID-19 Health Care Workers: Lesson for Future Pandemic Preparedness. ANAMS 2023;59:233-236. doi:10.1055/s-0043-1772218
62. Halemani K, Cheema M, Khatun S, Yadidya, Singh B, Gupta V K, Sharma A. An effectiveness of training program on COVID-19 among healthcare students: A cross section study. International Journal of Research in Pharmaceutical Sciences 2020;11(13):1250-1254. doi:10.26452/ijrps.v11iSPL1.3613
63. Han B, Zang F, Liu J, Li S, Zhang W, Zhang Y, Li Z. Effect Analysis of "Four-Step" Training and Assessment Tool in the Prevention and Control of COVID-19. Infection and drug resistance 2022;15:1247-1257. doi:10.2147/IDR.S346559
64. Hwang WJ, Lee J. Effectiveness of the Infectious Disease (COVID-19) Simulation Module Program on Nursing Students: Disaster Nursing Scenarios. Journal of Korean Academy of Nursing 2021;51(6):648-660. doi:10.4040/jkan.21164
65. Park YM. The development and effects of a simulation-based disaster nursing education program for nursing students using standardized patients. [Dissertation]. Seoul; 2020.
66. Worl Health Organization. COVID-19 strategic preparedness and response plan: Operational planning guideline: Licence: CC BY-NC-SA 3.0 IGO. Geneva: World Health Organization; 2021.
67. Instrum RS, Koch RW, Rocha T, Rohani SA, Ladak H, Agrawal SK, Sowerby LJ. Improving Nasopharyngeal Swab Technique via Simulation for Frontline Workers. The Laryngoscope 2022. doi:10.1002/lary.30034
68. Kasapoglu ES, Yildiz YS, Saldamli A, Karaçetin F. The effect of COVID-19 patient care and emergency response interprofessional training on COVID-19 knowledge, perception, behavior and readiness for care. WORK-A JOURNAL OF PREVENTION ASSESSMENT & REHABILITATION 2023;75(3):767-778. doi:10.3233/WOR-220227
69. Kaufman J, Overmars I, Fong J, Tudravu J, Devi R, Volavola L, Vodonaivalu L, Jenkins K, Leask J, Seale H, Mohamed Y, Joshi K, Datt H, Sagan S, Dynes M, Hoq M, Danchin M. Training health workers and community influencers to be Vaccine Champions: a mixed-methods RE-AIM evaluation. BMJ Global Health 2024;9(9). PMID:39251236
70. Kharel R, Baird J, Vaishnav H, Chillara N, Lee JA, Genisca A, Hayward A, Uzevski V, Elbenni A, Levine AC, Aluisio AR. Development and assessment of novel virtual COVID-19 trainer-of trainers course implemented by an academic-humanitarian partnership. Global health action 2022;15(1):2010391. doi:10.1080/16549716.2021.2010391
71. Khari S, Pazokian M, Abadi AS, Zarmehrparirouy M, Ahmadvand Y. The Effect of E-Learning Program for COVID-19 Patient Care on the Knowledge of Nursing Students: A Quasi-Experimental Study. SAGE Open Nursing 2022;8. doi:10.1177/23779608221124421
72. Kobayashi D, Mami K, Fujishiro S, Nukanobu N, Ueno SI, Kuwakado S, Koyama T, Kuga H. Online training of Covid-19 infection prevention and control for healthcare workers in psychiatric institutes. BMC Psychiatry 2023;23(1). doi:10.1186/s12888-023-04826-5
73. Kufel WD, Blaine BE, Avery LM. Pharmacy students' knowledge and confidence of COVID-19 following an interactive didactic class. Journal Of The American College Of Clinical Pharmacy 2022;5(10):1082-1087. doi:10.1002/jac5.1678
74. Lalitha ND, Bhadauria US, Agarwal D, Purohit BM, Priya H, Nilima N, Duggal R, Mathur VP, Logani A. Comparing the effectiveness of two educational methods for oral health management in COVID-19 pandemic among dental professionals. Przegl Epidemiol 2024;78(1):90-93. PMID:38904315
75. Li Z, Cheng J, Zhou T, Wang S, Huang S, Wang H. Evaluating a Nurse Training Program in the Emergency Surgery Department Based on the Kirkpatrick's Model and Clinical Demand During the COVID-19 Pandemic. Telemedicine journal and e-health : the official journal of the American Telemedicine Association 2020;26(8):985-991. doi:10.1089/tmj.2020.0089
76. Macht L, Worlitzsch D, Braijoshri N, Bequiri P, Zudock J, Zilezinski M, Stoevesandt D, Smith J, Hofstetter S. COVID-19: Development and implementation of a video-conference-based educational concept to improve the hygiene skills of health and nursing professionals in the Republic of Kosovo. GMS Hygiene and Infection Control 2022;17. doi:10.3205/dgkh000412
77. Heininger SK, Baumgartner M, Zehner F, Burgkart R, Söllner N, Berberat PO, Gartmeier M. Measuring hygiene competence: the picture-based situational judgement test HygiKo. BMC Medical Education 2021;21(1):410. PMID:34330263
78. Mark ME, LoSavio P, Husain I, Papagiannopoulos P, Batra PS, Tajudeen BA. Effect of Implementing Simulation Education on Health Care Worker Comfort With Nasopharyngeal Swabbing for COVID-19. Otolaryngology-head and neck surgery: Official journal of American Academy of Otolaryngology-Head and Neck Surgery 2020;163(2):271-274. doi:10.1177/0194599820933168
79. McConnell H, Duncan D, Stark P, Anderson T, McMahon J, Creighton L, Craig S, Carter G, Smart A, Alanazi A, Mitchell G. Enhancing COVID-19 Knowledge among Nursing Students: A Quantitative Study of a Digital Serious Game Intervention. Healthcare (Basel) 2024;12(11). doi:10.3390/healthcare12111066

80. World Health Organization. Coronavirus disease (COVID-19) advice for the public: Mythbusters 2022 URL: https://www.who.int/emergencies/diseases/novel-coronavirus-2019/advice-for-public/myth-busters [accessed 2025-10-22].
81. Mektrirat R, Sathanawongs A, Tiwananthagorn S, Chaisowwong W, Peansukmanee S, Naksen W, Thongprachum A. Achieving Interprofessional Education on Collaborative Problem-Solving for COVID-19 Using Project-Based Approach. International Journal of Infectious Diseases 2021;116:S67-. doi:10.1016/j.ijid.2021.12.158
82. Mohamed Y, Hezeri P, Kama H, Mills K, Walker S, Hau'ofa N, Amol C, Jones M, du Cros P, Lin YD. Evaluation of an Online Training Program on COVID-19 for Health Workers in Papua New Guinea. Tropical medicine and infectious disease 2023;8(6). doi:10.3390/tropicalmed8060327
83. Nassar AAH, Al Serouri AA, Al-Shahethi AH, Almoayed KA. Effectiveness of training on health care workers' knowledge, attitude and practice regarding COVID-19 infection prevention and control, Yemen, 2021. BMC Health Services Research 2024;24(1):1411. doi:10.1186/s12913-024-11927-8
84. Alrubaiee GG, Al-Qalah TAH, Al-Aawar MSA. Knowledge, attitudes, anxiety, and preventive behaviors towards COVID-19 among health care providers in Yemen: an online cross-sectional survey; 2020.
85. Ye Y, Shi P, Gui Y, Li AM, Huang G, Xu H, Lu Q, Hong J, Gu Y, Hu X, Liu G, Wang C, Huang Q, Zhang X. Point-of-care training program on COVID-19 infection prevention and control for pediatric healthcare workers: a multicenter, cross-sectional questionnaire survey in Shanghai, China. Translational Pediatrics 2021;10(1):44-53. PMID:33633936
86. Huynh G, Nguyen TH, Tran V, Vo K, Vo V, Pham L. Knowledge and attitude toward COVID-19 among healthcare workers at District 2 Hospital, Ho Chi Minh City. Asian Pac J Trop Med 2020;13(6):260. doi:10.4103/1995-7645.280396
87. Tadesse DB, Gebrewahd GT, Demoz GT. Knowledge, attitude, practice and psychological response toward COVID-19 among nurses during the COVID-19 outbreak in northern Ethiopia, 2020. New Microbes New Infect 2020;38:100787. PMID:33072339
88. Naz F, Ohri P, Sharma A, Spandana BS, Gupta K. Impact of Training on Awareness of COVID-19 among The Health Care Workers in A Tertiary Care Hospital of Dehradun. Indian Journal of Community Health 2022;34(1):20-25. doi:10.47203/IJCH.2022.v34i01.005
89. Odusanya OO, Adeniran A, Bakare OQ, Odugbemi BA, Enikuomehin OA, Jeje OO, Emechebe AC. Building capacity of primary health care workers and clients on COVID-19: Results from a web-based training. PLOS ONE 2022;17(10):e0274750. doi:10.1371/journal.pone.0274750
90. Honarvar B, Lankarani KB, Kharmandar A, Shaygani F, Zahedroozgar M, Rahmanian Haghighi MR, Ghahramani S, Honarvar H, Daryabadi MM, Salavati Z, Hashemi SM, Joulaei H, Zare M. Knowledge, attitudes, risk perceptions, and practices of adults toward COVID-19: a population and field-based study from Iran. Int J Public Health 2020;65(6):731-739. PMID:32583009
91. Paul A, Sikdar D, Hossain MM, Amin MR, Deeba F, Mahanta J, Jabed MA, Islam MM, Noon SJ, Nath TK. Knowledge, attitudes, and practices toward the novel coronavirus among Bangladeshis: Implications for mitigation measures. PLOS ONE 2020;15(9):e0238492. PMID:32877449
92. Otu A, Okuzu O, Effa E, Ebenso B, Ameh S, Nihalani N, Onwusaka O, Tawose T, Olayinka A, Walley J. Training health workers at scale in Nigeria to fight COVID-19 using the InStrat COVID-19 tutorial app: an e-health interventional study. Therapeutic advances in infectious disease 2021;8:20499361211040704. doi:10.1177/20499361211040704
93. Otu A, Okuzu O, Ebenso B, Effa E, Nihalani N, Olayinka A, Yaya S. Introduction of Mobile Health Tools to Support COVID-19 Training and Surveillance in Ogun State Nigeria. Front. Sustain. Cities 2021;3. doi:10.3389/frsc.2021.638278
94. Perera N, Haldane V, Ratnapalan S, Samaraweera S, Karunathilake M, Gunarathna C, Bandara P, Kawirathne P, Wei XL. Implementation of a coronavirus disease 2019 infection prevention and control training program in a low-middle income country. JBI Evidence Implementation 2022;20(3):228-235. doi:10.1097/XEB.0000000000000307
95. Pokrajac N, Schertzer K, Poffenberger CM, Alvarez A, Marin-Nevarez P, Winstead-Derlega C, Gisondi MA. Mastery Learning Ensures Correct Personal Protective Equipment Use in Simulated Clinical Encounters of COVID-19. West J Emerg Med 2020;21(5):1089-1094. doi:10.5811/westjem.2020.6.48132
96. Puga RR, Cardoso AL, Rodríguez OL. Educational intervention in medical students on post-COVID-19 disabling sequelae from the Public Health subject. Revista Cubana De Reumatologia 2023;25(3).
97. Qasmi SA, Standley C, Mohsin S, Sarwar S, Malik L, Aziz F. Effectiveness of international virtual training on biorisk management in the context of COVID-19. Frontiers in public health 2022;10. doi:10.3389/fpubh.2022.888097
98. Roberts EN, Smithing RT, Tucker P. Measuring the impact of a COVID-19 continuing education program. Journal of the American Association of Nurse Practitioners 2022;34(6):835-843. doi:10.1097/JXX.0000000000000715
99. Moore DE, Green JS, Gallis HA. Achieving desired results and improved outcomes: integrating planning and assessment throughout learning activities. J Contin Educ Health Prof 2009;29(1):1-15. PMID:19288562
100. Roberts KJ, Zumstein KK, Lamphere TR, Williams M, Powell SA, Moran A, Kellar B, Solly WR, Pierce M. Improving Students' Knowledge and Skills Through a Tele-ICU Clinical Rotation. Respiratory Care 2022;67(7):789-794. doi:10.4187/respcare.09896
101. Rosas-Magallanes C, Basto-Abreu A, Barrientos-Gutiérrez T, Ramírez-Martínez JL, Tamayo-Ortiz M, Gutiérrez-Díaz HO, Magaña-Valladares L, Cordera DB, Santamaría-Guasch CM, Hernández-Avila M. CLIMSS online platform as a health literacy tool during the health crisis of Covid-19. Salud Publica De Mexico 2022;64(3):320-327. doi:10.21149/13103
102. Saati AA, Alkalash SH. Promotion of knowledge, attitude, and practice among medical undergraduates regarding infection control measures during COVID-19 pandemic. Frontiers in public health 2022;10. doi:10.3389/fpubh.2022.932465
103. Sabandüzen H, Kavaklı Ö. Evaluation of the effectiveness of the training on "Home care of COVID-19 positive/suspicious patients" given to nursing students: A quasi-experimental study. Journal of education and health promotion 2024;13:250. doi:10.4103/jehp.jehp_1574_23
104. Said AR. Knowledge and Practices of Nurses Regarding Corona Virus (COVID-19): An Educational Intervention. MLU 2021;21(2):36-47. doi:10.37506/mlu.v21i2.2642
105. Salehi R, Young S de, Asamoah A, Aryee SE, Eli R, Couper B, Smith B, Djokoto C, Agyeman YN, Zakaria AF, Butt N, Boadu A, Nyante F, Merdiemah G, Oliver-Commey J, Ofori-Boadu L, Akoriyea SK, Parry M, Fiore C, Okae F, Adams A, Acquah H. Evaluation of a continuing professional development strategy on COVID-19 for 10 000 health workers in Ghana: a two-pronged approach. Human resources for health 2023;21(1). doi:10.1186/s12960-023-00804-w
106. Sangwan J, Lathwal S, Lohan K, Yadav K, Adlakha N, Mane P, Gole S. Impact of training on Knowledge, Attitude and Perceived Barriers for Compliance Regarding use of Protective Equipment Kit among Frontline Healthcare Workers during COVID-19 Pandemic. J Clin Diagn Res 2022;16(1):JC17-JC21. doi:10.7860/JCDR/2022/50845.15913

107. Tomas ME, Kundrapu S, Thota P, Sunkesula VCK, Cadnum JL, Mana TSC, Jencson A, O'Donnell M, Zabarsky TF, Hecker MT, Ray AJ, Wilson BM, Donskey CJ. Contamination of Health Care Personnel During Removal of Personal Protective Equipment. JAMA Internal Medicine 2015;175(12):1904-1910. PMID:26457544
108. Shahrin L, Parvin I, Sarmin M, Abbassi NA, Ackhter MM, Alam T, Mamun GMS, Rahman A, Shaima SN, Shikha SS, Jeorge DH, Nahar MA, Sharifuzzaman, Saha H, Rahman ASMMH, Shahid ASMSB, Faruque ASG, Ahmed T, Chisti MJ. In-person training on COVID-19 case management and infection prevention and control: Evaluation of healthcare professionals in Bangladesh. PLOS ONE 2022;17(10):e0273809. doi:10.1371/journal.pone.0273809
109. Sharma R, Mohanty A, Singh V, S VA, Gupta PK, Jelly P, Gupta P, Rao S. Effectiveness of Video-Based Online Training for Health Care Workers to Prevent COVID-19 Infection: An Experience at a Tertiary Care Level Institute, Uttarakhand, India. Cureus 2021;13(5):e14785. doi:10.7759/cureus.14785
110. Singh V, Supehia S, Gupta PK, Narula H, Sharma M, Devi K, Bhute AR. Effectiveness of video modules in infection control trainings during COVID-19 pandemic: A quasi-experimental study in tertiary care institute. Journal of education and health promotion 2021;10(1):183. doi:10.4103/jehp.jehp_1009_20
111. Smith CR, Vasilopoulos T, am Frantz, LeMaster T, Martinez RA, am Gunnett, Fahy BG. Staying proper with your personal protective equipment: How to don and doff. Journal Of Clinical Anesthesia 2023;86. doi:10.1016/j.jclinane.2023.111057
112. Strehlow MC, Johnston JS, Aluri KZ, Prober CG, Acker PC, Patil AS, Mahadevan A, Mahadevan SV. Evaluation of a massive open online course for just-in-time training of healthcare workers. Frontiers in public health 2024;12:1395931. doi:10.3389/fpubh.2024.1395931
113. Ta'an WF, Al-Hammouri MM, Al-Faouri I, Suliman MM. The effectiveness of COPA-based training program on the infection- control competencies of newly hired healthcare professionals. Teaching and Learning in Nursing 2023;18(1):160-165. doi:10.1016/j.teln.2022.06.009
114. Tan W, Ye Y, Yang Y, Chen Z, Yang X, Zhu C, Chen D, Tan J, Zhen C. Whole-Process Emergency Training of Personal Protective Equipment Helps Healthcare Workers Against COVID-19: Design and Effect. J Occup Environ Med 2020;62(6):420-423. doi:10.1097/JOM.0000000000001877
115. Thakre SS, Jadhao AR, Dhoble MA, Dass R, Thakre SB, Somani A. Evaluation of Effectiveness of Covid-19 Training and Assessment of Anxiety among Nurses of a Tertiary Health Care Center during the Corona Virus Pandemic-An Experimental Study. Journal Of Clinical and Diagnostic Research 2020;14(11):LC34-LC37. doi:10.7860/JCDR/2020/45464.14301
116. Thakre SS, Thakre SB, Jadhao A, Dass R, Dhoble MA, Tiwari PN. Evaluation of effectiveness of COVID-19 training of tertiary health care workers. Int J Community Med Public Health 2020;7(7):2635. doi:10.18203/2394-6040.ijcmph20202989
117. Tsiouris F, Hartsough K, Poimbouef M, Raether C, Farahani M, Ferreira T, Kamanzi C, Maria J, Nshimirimana M, Mwanza J, Njenga A, Odera D, Tenthani L, Ukaejiofo O, Vambe D, Fazito E, Patel L, Lee C, Michaels-Strasser S, Rabkin M. Rapid scale-up of COVID-19 training for frontline health workers in 11 African countries. Human resources for health 2022;20(1):43. doi:10.1186/s12960-022-00739-8
118. Wang SH, Yimer G, Bisesi M, Lisawork L, Sugerman D, Alayu M, Wossen M, Abayneh SA, Gallagher K, Endashaw T, Kubinson H, Kanter T, Gallagher K, Gebreyes W. Rapid virtual training and field deployment for COVID-19 surveillance officers: experiences from Ethiopia. Pan African medical journal 2022;43:23. doi:10.11604/pamj.2022.43.23.28787
119. Wu TY, Hoffman JL, Chow CM, Hartl B. Training community health navigators in the public health workforce to respond during the COVID-19 pandemic. Z Gesundh Wiss 2023:1-8. doi:10.1007/s10389-022-01812-1
120. Zafar N, Jamal Z, Mujeeb Khan M. Preparedness of the Healthcare Personnel Against the Coronavirus Disease 2019 (COVID-19) Outbreak: An Audit Cycle. Frontiers in public health 2020;8:502. doi:10.3389/fpubh.2020.00502
121. Centers for Disease Control and Prevention. Healthcare professional preparedness checklist for transport and arrival of patients with Confirmed or Possible COVID-19 2020 URL: https://www.cdc.gov/ coronavirus/2019-ncov/hcp/hcp-personnel-checklist.html.
122. Zhao J, Rozenberg D, Kaul R, Sanh M, Luther R, Orchanian-Cheff A, Nourouzpour S, de Peiza P, Agbeyaka S, Gebara N, Doumouras AM, Draper H, Barber M, Lau J, Furlan A. The positive impact of a telemedicine education program on healthcare workers during the COVID-19 pandemic in Ontario, Canada. The Annals of Family Medicine 2022(20). doi:10.1370/afm.20.s1.3260

Table S3. Characteristics of Ongoing Studies

Study (Author, time of registration)	Study design	Population	Interventions & comparators	Outcomes	Notes
**Stengel et al. [1] (09/2023)**	Pilot-RCT	Mixed occupation groups: health professionals & medical students	**Intervention:** Online-on-demand training on post-COVID/post-infectious syndromes<br>**Control:** Delayed intervention (participants receive same training after the end of the study)	**Primary:**<br>Changes in post-COVID-19 knowledge (means)	German Clinical Trials Register ID: DRKS00032630<br>Current status: Recruiting
**Leung et al. [2] (05/2021)**	Cluster-RCT	Staff in residential care homes (RCHs): e. g. doctors, nurses, occupational therapists, physiotherapists, health workers, and personal care workers who are working in the RCHs	**Intervention:** Blended Gaming COVID-19 Training (2 weeks)<br>**Control:** Usual care: infection control briefing given by the Infection Control Officer of the RCHs to all staff	**Primary:**<br>Infection control performance & knowledge<br>**Secondary:**<br>Attitudes towards infection control	ClinicalTrials.gov ID NCT04783025
**Johnston et al. [3] (11/2021)**	RCT	Health professionals	**Intervention:** COVID-19 Vaccine Interactive Voice Response Training<br>**Control:** Delayed intervention (participants will receive intervention one-month after intervention group)	**Primary:**<br>COVID-19 vaccine knowledge	ClinicalTrials.gov ID NCT05107479
**Pala et al. [4] (12/2021)**	RCT	Pre-hospital emergency health worker (paramedic or emergency medical technicians)	**Intervention:** Video training<br>**Control:** Classical method (not specified)	**Primary:**<br>COVID-19 knowledge & practice<br>**Secondary:**<br>COVID-19 infection control attitudes	ClinicalTrials.gov ID NCT05192967
**Yang et al. [5] (04/2024)**	RCT	Bachelor nursing students (year one)	**Intervention:**<br>IG1: Hand scanner instant feedback<br>IG2: Video training<br>IG3: Hand scanner & video training<br>**Control:** No intervention	**Primary:**<br>Hand hygiene performance & knowledge	ClinicalTrials.gov ID NCT05872581

RCT Randomised controlled trial, **RCH** Residential Care Homes

References

1. Stengel S. Piloting interventions (case conferences, training network) for adaptive, cross-sectoral health care Long-/Post-COVID in Baden-Württemberg - SEVEN - PCS: DRKS00032630 2023 URL: https://www.cochranelibrary.com/central/doi/10.1002/central/CN-02604635/full.
2. Leung A. A Blended Gaming COVID-19 Training System (BGCTS) With WHO Guidelines for Staff in Residential Care Homes: NCT04783025 2021 URL: https://www.cochranelibrary.com/central/doi/10.1002/central/CN-02249570/full.
3. Johnston J. Effectiveness of Interactive Voice Response for COVID-19 Vaccination Training in the Democratic Republic of the Congo: NCT05107479 2021 URL: https://www.cochranelibrary.com/central/doi/10.1002/central/CN-02352592/full.
4. Seval Calıskan Pala. Development and Efficiency Evaluation of a Respiratory Disease Pandemics Preparedness Training Program for Pre-Hospital Emergency Healthcare Workers. clinicaltrials.gov 2021 URL: https://clinicaltrials.gov/show/NCT05192967.
5. Yang L. Education Program of Hand Hygiene for Nursing Students: NCT05872581 2023 URL: https://www.cochranelibrary.com/central/doi/10.1002/central/CN-02564780/full.

Table S4. Characteristics of Studies Awaiting Classification

Study (Author, time of registration)	Study design	Population	Interventions & comparators	Outcomes	Notes
**Hanson et al. 2020** [1]	Uncontrolled before-after study	Mixed health professionals occupation groups: e.g. infectious disease specialists, pulmonary medicine specialists, emergency room practitioners, advanced practitioners, nurses, & other healthcare professional	**Intervention:** Intra-activity Q&A, evaluations, 60- to 90-day follow-on assessments, technology enhances learning mode (virtual live learning platforms, mobile websites/apps, 3D animations & podcasts)	**Primary**: Changes in COVID-19 knowledge & competence	Only Abstract

References

1. Hanson B, Welch L, Frese M, Gallo C. Innovative cme tools in the teaching of evolving strategies in the management and prevention of COVID-19. Open forum infectious diseases 2020;Conference Infectious Diseases Week, IDWeek 2020. Virtua:S344. doi:10.1093/ofid/ofaa439.752
