# Supplementary material for: Interventions to Enhance COVID-19 Pandemic Health Literacy in Health Professionals: Systematic Review
Source: JMIR Med Educ. 2026 Jul 10;12:e70400. doi: 10.2196/70400 (PMC13360183; doi:10.2196/70400)
Supplement: Multimedia Appendix 3 — Risk of bias assessments. [file mededu-v12-e70400-s003.pdf]

Table S1. Risk of Bias Assessments of Randomised Controlled Trials with Risk of Bias 2 Tool – Primary Outcomes

Study (Author, Year)	Outcome	Results	Risk of bias					
			D1	D2	D3	D4	D5	Overall risk of bias
**Alotaibi et al. 2021** [1]	COVID-19 related vaccination knowledge	COVID-19 related vaccination knowledge (Intervention groups: brochure, Instagram, workshop)[a] (Follow Up 1)	Low	Some concerns	High	High	Some concerns	High
**Amiri et al. 2023** [2]	COVID-19 specific knowledge on proving services in women	COVID-19 specific knowledge on providing services in women during pregnancy, childbirth, and breastfeeding (Follow Up 1 & 2)	Some concerns	Low	Low	Some concerns	Some concerns	Some concerns
	COVID-19 related performance skills in providing services in women	COVID-19 related performance skills in providing services in women during pregnancy, childbirth, and breastfeeding (Follow Up 1 & 2)	Some concerns	Low	Low	Some concerns	Some concerns	Some concerns
**Birrenbach et al. 2021** [3]	COVID-19 related infection protection performance skills	Hand disinfection performance (Follow Up 1 & 2)[a]	Some concerns	Some concerns	Low	Low	Some concerns	Some concerns
		Nasopharyngeal Swab performance (Follow Up 1 &2)[a]	Some concerns	Some concerns	Low	Low	Some concerns	Some concerns
		Personal protective equipment (PPE) performance (Doffing, Follow Up 1 & 2)[a]	Some concerns	Some concerns	Low	Low	Some concerns	Some concerns

Study (Author, Year)	Outcome	Results	Risk of bias					
			D1	D2	D3	D4	D5	Overall risk of bias
**Christensen et al. 2020** [4]	COVID-19 related infection protection performance skills	PPE performance (Donning, Doffing)[a]	Low	Some concerns	High	Low	Some concerns	High
**Currat et al. 2022** [5]	COVID-19 related infection prevention knowledge on PPE	Knowledge acquisition in contaminated zone & non-contaminated zone (Follow Up 1)[a]	Low	Low	Low	Low	Low	Low
		Knowledge acquisition (Follow Up 1, Full sequence)	Low	Low	Low	Low	Low	Low
		Knowledge retention in contaminated zone & non-contaminated zone (Follow Up 2)[a]	Low	Low	Low	Low	Low	Low
		Knowledge retention (Follow Up 2, Full sequence)	Low	Low	Low	Low	Low	Low
	COVID-19 related infection protection performance skills	Skill retention (Doffing, Follow Up 1 & 2)	Low	Low	Low	Low	Low	Low
		Hand disinfection correctly (Knowledge acquisition (Follow Up 1), retention (Follow Up2))[a]	Low	Low	Low	Low	Low	Low
**Jeihooni et al. 2023** [6]	General COVID-19 related knowledge (Follow Up 1)		Low	Low	Low	Some concerns	Some concerns	Some concerns
**Li et al. 2020** [7]	COVID-19 related infection protection performance skills	PPE performance (Donning & Doffing, Follow Up 1)	Low	Some concerns	Low	Low	Some concerns	Some concerns
**Manggala et al. 2020** [8]	COVID-19 related infection protection performance skills	PPE performance (Donning & Doffing, Follow Up 1)[a]	Some concerns	Some concerns	Low	Some concerns	Some concerns	Some concerns

Study (Author, Year)	Outcome	Results	Risk of bias					
			D1	D2	D3	D4	D5	Overall risk of bias
**Rueda-Medina et al. 2022** [9]	COVID-19 related infection protection performance skills	PPE performance (Donning & Doffing, Follow Up 1)	Low	Low	Low	Some concerns	Some concerns	Some concerns
**Suppan et al. 2020a** [10]	COVID-19 related infection prevention knowledge on PPE (Follow Up 1)		Low	Low	Some concerns	Some concerns	Some concerns	Some concerns
**Suppan et al. 2020b** [11]	COVID-19 related infection prevention knowledge on PPE (Follow Up 1)		Low	High	Some concerns	Some concerns	Some concerns	High
	COVID-19 related infection protection performance skills	PPE performance (Donning & Doffing, Follow Up 1)[a]	Low	High	Some concerns	Some concerns	Some concerns	High
**Wang et al. 2022** [12]	COVID-19 related performance skills on PPE	PPE performance (Follow Up 1)	Some concerns	Some concerns	Low	Some concerns	Some concerns	Some concerns
	COVID-19 related knowledge on infection prevention control measures (Follow Up 1, Outcome not further specified)		Some concerns	Some concerns	Low	Some concerns	Some concerns	Some concerns
**Xie et al. 2021** [13]	COVID-19 related infection prevention knowledge on PPE	PPE performance (Donning & Doffing, Follow Up 1)	Low	High	Low	Some concerns	Some concerns	High
	COVID-19 related infection protection performance skills	PPE performance (Donning & Doffing, Follow Up 1)[a]	Low	High	Low	Some concerns	Some concerns	High

a The risk of bias assessment for each result is summarised, as there are no differences between them
D1 Bias arising from the randomisation process, **D2** Bias due to deviations from the intended interventions, **D3** Bias due to missing outcome data, **D4** Bias in measurement of the outcome, **D5** Bias in selection of the reported result,
PPE Personal protective eqiupment

Table S2. Risk of Bias Assessments of Randomised Controlled Trials with Risk of Bias 2 Tool – Secondary Outcomes

Study (Author, Year)	Outcome	Results	Risk of bias					
			D1	D2	D3	D4	D5	Overall risk of bias
**Amiri et al. 2023** [2]	COVID-19 related attitudes towards providing services in women during pregnancy, childbirth, and breastfeeding (Follow Up 1 & 2)		Some concerns	Low	Low	Some concerns	Some concerns	Some concerns
**Currat et al. 2022** [5]	Perceived confidence in COVID-19 related infection protection performance skills	Perceived confidence in using personal protective equipment (PPE)	Low	Low	Low	Some concerns	Low	Some concerns
**Jafree et al. 2022** [14]	Confidence thinking regarding infection prevention control (Follow Up 1, *composite outcome*)		Some concerns	Some concerns	Some concerns	Some concerns	Some concerns	Some concerns
	COVID-19 related knowledge and perceived abilites (Follow Up 1, *composite outcome*)		Some concerns	Some concerns	Some concerns	Some concerns	Some concerns	Some concerns
**Jeihooni et al. 2023** [6]	Perceived COVID-19 related self-efficacy (Follow Up 1)		Low	Low	Low	Some concerns	Some concerns	Some concerns
	Prevalence of infection prevention behaviour (Follow Up 1)		Low	Low	Low	Some concerns	Some concerns	Some concerns
**Li et al. 2020** [7]	Perceived confidence in COVID-19 related infection protection performance skills	Perceived confidence in using PPE (Donning & Doffing, Follow Up 1)	Low	Some concerns	Low	Some concerns	Some concerns	Some concerns

Study (Author, Year)	Outcome	Results	Risk of bias					
			D1	D2	D3	D4	D5	Overall risk of bias
**Rakhshani et al. 2024** [15]	Attitudes towards COVID-19 infection prevention behaviors (Follow Up 1)		Some concerns	Low	Low	Some concerns	Some concerns	Some concerns
**Rueda-Medina et al. 2022** [9]	Perceived confidence in COVID-19 related infection protection performance skills	Perceived confidence in using PPE (Donning & Doffing, Follow Up 1)[a]	Low	Low	Low	Some concerns	High	High
**Suppan et al. 2020a** [10]	Perceived confidence in COVID-19 related infection protection performance skills	Perceived confidence in the ability of using PPE (Follow Up 1)	Low	Low	Some concerns	Some concerns	Some concerns	Some concerns
**Suppan et al. 2020b** [11]	Perceived confidence in COVID-19 related infection protection performance skills	Perceived confidence in the ability of using PPE (Follow Up 1)	Low	High	Some concerns	Some concerns	Some concerns	High

D1 Bias arising from the randomisation process, **D2** Bias due to deviations from the intended interventions, **D3** Bias due to missing outcome data, **D4** Bias in measurement of the outcome, **D5** Bias in selection of the reported result
PPE Personal protective eqiupment

Table S3. Risk of Bias Assessment of Non-Randomised Controlled Studies with the ROBINS-I-Tool – Primary Outcomes

Study (Author, Year)	Outomes	Domain 1 Bias due to confounding	Domain 2 Bias in selection of participants into the study	Domain 3 Bias in classification of interventions	Domain 4 Bias due to deviations from the intended intervention	Domain 5 Bias due to missing data	Domain 6 Bias due to measurement of outcomes	Domain 7 Bias in selection of the reported result	Overall Risk of bias
**Ansari et al. 2022** [16]	COVID-19 related performance skills on personal protective eqiupment performance (PPE) (Donning, Follow Up 1)	Serious	Low	Low	Low	NI	Low	Moderate	Serious
**Rationale for judgement**		At least one known important domain was not appropriately measured, or not controlled for.	All participants who would have been eligible for the target trial were included in the study; and for each participant, start of follow up and start of intervention coincided.	The intervention status is well defined and the intervention definition is based solely on information collected at the time of intervention.	Any deviations from intended intervention reflected usual practice.	No information is reported about missing data or the potential for data to be missing.	The methods of outcome assessment were comparable across intervention groups. The outcome assessors were unaware of the intervention received by study participants. Any error in measuring the outcome is unrelated to intervention status.	The outcome measurements and analyses are clearly defined and both internally and externally consistent. There is no indication of selection of the reported analysis from among multiple analyses.) There is no indication of selection of the cohort or subgroups for analysis and reporting on the basis of the results.	The study is judged to be at serious risk of bias in at least one domain, but not at critical risk of bias in any domain.

Study (Author, Year)	Outomes	Domain 1	Domain 2	Domain 3	Domain 4	Domain 5	Domain 6	Domain 7	Overall Risk of bias
**Ansari et al. 2022** [16]	COVID-19 related performance skills on PPE (Doffing, Follow Up 1)	Serious	Low	Low	Low	NI	Low	Moderate	Serious
**Rationale for judgement**		At least one known important domain was not appropriately measured, or not controlled for.	All participants who would have been eligible for the target trial were included in the study; and for each participant, start of follow up and start of intervention coincided.	The intervention status is well defined and the intervention definition is based solely on information collected at the time of intervention.	Any deviations from intended intervention reflected usual practice.	No information is reported about missing data or the potential for data to be missing.	The methods of outcome assessment were comparable across intervention groups. The outcome assessors were unaware of the intervention received by study participants. Any error in measuring the outcome is unrelated to intervention status.	The outcome measurements and analyses are clearly defined and both internally and externally consistent. There is no indication of selection of the reported analysis from among multiple analyses.) There is no indication of selection of the cohort or subgroups for analysis and reporting on the basis of the results.	The study is judged to be at serious risk of bias in at least one domain, but not at critical risk of bias in any domain.
**Buyego et al. 2022** [17]	General COVID-19 related knowledge (Follow Up 1)	Serious	Serious	Low	Low	NI	NI	NI	Serious
**Rationale for judgement**		At least one known important domain was not appropriately measured, or not controlled for.	Start of follow up and start of intervention do not coincide.	The intervention status is well defined and the intervention definition is based solely on information collected at the time of intervention.	Any deviations from usual practice were unlikely to impact on the outcome.	No information is reported about missing data or the potential for data to be missing.	No information is reported about the methods of outcome assessment.	There is too little information to make a judgement.	The study is judged to be at serious risk of bias in at least one domain, but not at critical risk of bias in any domain.

Study (Author, Year)	Outomes	Domain 1	Domain 2	Domain 3	Domain 4	Domain 5	Domain 6	Domain 7	Overall Risk of bias
**Hu et al. 2021** [18]	General COVID-19 related knowledge (Follow Up 1 & 2)	Serious	Low	Low	Low	Low	Moderate	Moderate	Serious
**Rationale for judgement**		Confounding is probably present (e. g. pre-existing COVID-19 knowledge, interest to gain more knowledge, socioeconomic status) due to the nature of the study design, but no effort is reported for adjusting or controlling this kind of confounding.	All participants, who would have been eligible for the study were included in the study before the start of the intervention. For each participants, start of follow up and start of.intervention coincided.	The intervention status is well defined and the intervention definition is based solely on information collected at the time of intervention.	Any likely deviations from the intended intervention reflected usual practice and were unlikely to impact on the outcome.	Data were reasonably complete; Proportions of missing participants were similar across interventions groups.	The methods of outcome assessment were comparable across intervention groups (game vs. online lecture), the outcome measure is only minimally influenced by knowledge of the intervention received by study participants (participant-reported) and any error in measuring the outcome is probably only minimally related to intervention status.	The outcome measurements and analyses are clearly defined and both internally and externally consistent. There is no indication of selection of the reported analysis. However, no pre-registered protocol or statistical analysis plan was available.	The study is judged to be at serious risk of bias in at least one domain, but not at critical risk of bias in any domain.

Study (Author, Year)	Outomes	Domain 1	Domain 2	Domain 3	Domain 4	Domain 5	Domain 6	Domain 7	Overall Risk of bias
**Yu et al. 2022** [19]	COVID-19 related infection prevention knowledge on PPE performance (Follow Up 1)	Serious	Moderate	Low	Low	Low	Moderate	Moderate	Serious
**Rationale for judgement**		More than one known important confounding domain was not appropriately measured and not controlled for.	Start of follow up and start of intervention do not coincide for all participants. And the review authors are confident that the rate (hazard)	The intervention status is well defined and the intervention definition is based solely on information collected at the	Any deviations from usual practice were unlikely to impact on the outcome.	Data were reasonably complete, i.e. data for all participants were available.	The methods of outcome assessment were comparable across intervention groups (online vs. written	The outcome measurements and analyses are clearly defined and both internally and externally consistent. There is no indication of	The study is judged to be at serious risk of bias in at least one domain, but not at critical risk of

			ratio for the effect of intervention remains constant over time.	time of intervention.			questionnaire), the outcome measure is only minimally influenced by knowledge of the intervention received by study participants (self-reported) and any error in measuring the outcome is probably only minimally related to intervention status.	selection of the reported analysis from among multiple analyses or selection of the cohort or subgroups for analysis and reporting on the basis of the results. However, no pre-registered protocol or statistical analysis plan was available.	bias in any domain.
**Yu et al. 2022** [19]	COVID-19 related infection protection performance skills of PPE (Follow Up 1)	Serious	Low	Low	Low	Low	Moderate	Moderate	Serious
**Rationale for judgement**		At least one known important domain was not appropriately measured, or not controlled for	All participants who would have been eligible for the target trial were included in the study. For each participant, start of follow up and start of intervention coincided.	The intervention status is well defined. The intervention definition is based solely on information collected at the time of intervention.	Any deviations from intended intervention reflected usual practice	Data were reasonably complete.	The methods of outcome assessment were comparable across intervention groups and the outcome measure was subjective, i.e. outcome measure might be influenced by knowledge of the intervention received by study participants.	The outcome measurements and analyses are clearly defined and both internally and externally consistent. There is no indication of selection of the reported analysis from among multiple analyses. There is no indication of selection of the cohort or subgroups for analysis and reporting on the basis of the results.	The study is judged to be at serious risk of bias in at least one domain, but not at critical risk of bias in any domain.

PPE Personal protective eqiupment

Table S4. Risk of Bias Assessments of Uncontrolled Before-After Studies without Control Group with the Quality Assessment Tool for Quantitative Studies

Study (Author, Year)	Selection Bias	Study Design	Confounders	Blinding	Data collection methods	Withdrawals and drop-outs	Global Rating
**Population: Health Professionals**							
**Abbas et al. 2020** [20]	Moderate	Moderate	Weak	Weak	Weak	Strong	Weak
**Ahmed et al. 2022** [21]	Weak	Moderate	Weak	Weak	Strong	Weak	Weak
**Altillo et al. 2021** [22]	Weak	Moderate	Weak	Moderate	Weak	Weak	Weak
**Aqel et al. 2023** [23]	Weak	Moderate	Weak	Weak	Moderate	Weak	Weak
**Aujee et al. 2022** [24]	Weak	Moderate	Weak	Weak	Moderate	Strong	Weak
**Bakhsh et al. 2023** [25]	Moderate	Moderate	Weak	Weak	Weak	Strong	Weak
**Bayomi et al. 2021** [26]	Moderate	Moderate	Weak	Weak	Moderate	Strong	Weak
**Bechini et al. 2023** [27]	Weak	Moderate	Weak	Weak	Weak	Strong	Weak
**Bieri et al. 2023** [28]	Moderate	Moderate	Weak	Weak	Weak	Moderate	Weak
**Blake et al. 2022** [29]	Moderate	Moderate	Weak	Weak	Weak	Weak	Weak
**Boccalini et al. 2022** [30]	Weak	Moderate	Weak	Weak	Weak	Strong	Weak
**Bohara et al. 2021** [31]	Weak	Moderate	Weak	Weak	Weak	Moderate	Weak
**Calik et al. 2021** [32]	Moderate	Moderate	Weak	Moderate	Weak	Strong	Weak
**Clay et al. 2021** [33]	Weak	Moderate	Weak	Weak	Weak	Strong	Weak
**Díaz-Guio et al. 2020 [34]**	Moderate	Moderate	Weak	Weak	Weak	Weak	Weak
**Elasrag et al. 2021** [35]	Moderate	Moderate	Weak	Weak	Weak	Strong	Weak
**Etebarian et al. 2023** [36]	Moderate	Moderate	Weak	Weak	Moderate	Moderate	Weak
**Fadel et al. 2025** [37]	Weak	Moderate	Weak	Weak	Strong	Strong	Weak
**Findyartini et al. 2021** [38]	Moderate	Moderate	Weak	Weak	Weak	Strong	Weak
**Fuentes et al. 2023** [39]	Weak	Moderate	Weak	Weak	Weak	Strong	Weak
**Garcia et al. 2024** [40]	Weak	Moderate	Weak	Weak	Weak	Weak	Weak
**Girard et al. 2022** [41]	Moderate	Moderate	Weak	Weak	Weak	Weak	Weak
**Greaves et al. 2023** [42]	Moderate	Moderate	Weak	Weak	Weak	Weak	Weak
**Gupta et al. 2023** [43]	Weak	Moderate	Weak	Weak	Strong	Strong	Weak
**Halemani et al. 2020** [44]	Weak	Moderate	Weak	Weak	Strong	Strong	Weak
**Han et al. 2022** [45]	Moderate	Moderate	Weak	Weak	Weak	Strong	Weak

Study (Author, Year)	Selection Bias	Study Design	Confounders	Blinding	Data collection methods	Withdrawals and drop-outs	Global Rating
**Population: Health Professionals**							
**Hwang et al. 2021** [46]	Weak	Moderate	Weak	Weak	Weak	Strong	Weak
**Instrum et al. 2022** [47]	Weak	Moderate	Weak	Weak	Weak	Strong	Weak
**Kasapoglu et al. 2023** [48]	Weak	Moderate	Weak	Weak	Weak	Weak	Weak
**Khari et al. 2022** [49]	Moderate	Moderate	Weak	Weak	Weak	Weak	Weak
**Kobayashi et al. 2023** [50]	Moderate	Moderate	Weak	Weak	Weak	Weak	Weak
**Kufel et al. 2022** [51]	Weak	Moderate	Weak	Weak	Moderate	Moderate	Weak
**Lalitha et al. 2024** [52]	Weak	Moderate	Weak	Weak	Weak	Strong	Weak
**Li et al. 2020** [53]	Weak	Moderate	Weak	Moderate	Weak	Strong	Weak
**Macht et al. 2022** [54]	Moderate	Moderate	Weak	Moderate	Strong	Weak	Weak
**Mark et al. 2020** [55]	Moderate	Moderate	Weak	Weak	Weak	Weak	Weak
**McConnell et al. 2024** [56]	Weak	Moderate	Weak	Weak	Weak	Moderate	Weak
**Mektirat et al. 2021** [57]	Weak	Moderate	Weak	Moderate	Weak	Weak	Weak
**Mohamed et al. 2023** [58]	Moderate	Moderate	Weak	Weak	Weak	Weak	Weak
**Nassar et al. 2024** [59]	Weak	Moderate	Weak	Weak	Strong	Strong	Weak
**Naz et al. 2022** [60]	Moderate	Moderate	Weak	Weak	Weak	Strong	Weak
**Odysanya et al. 2022** [61]	Moderate	Moderate	Weak	Weak	Moderate	Strong	Weak
**Otu et al. 2021a** [62]	Moderate	Moderate	Weak	Weak	Weak	Weak	Weak
**Otu et al. 2021b** [63]	Moderate	Moderate	Weak	Moderate	Weak	Weak	Weak
**Perera et al. 2022** [64]	Moderate	Moderate	Weak	Weak	Weak	Strong	Weak
**Pokrajac et al. 2020** [65]	Moderate	Moderate	Weak	Weak	Weak	Weak	Weak
**Puga et al. 2023** [66]	Weak	Moderate	Weak	Weak	Weak	Strong	Weak
**Qasmi et al. 2022** [67]	Weak	Moderate	Weak	Weak	Weak	Moderate	Weak
**Roberts et al. 2022a** [68]	Weak	Moderate	Weak	Weak	Weak	Weak	Weak
**Roberts et al. 2022b** [69]	Moderate	Moderate	Weak	Moderate	Weak	Strong	Moderate
**Rosas-Magallanes et al. 2022** [70]	Weak	Moderate	Weak	Weak	Weak	Strong	Weak
**Saati et al. 2022** [71]	Moderate	Moderate	Weak	Weak	Weak	Strong	Weak

Study (Author, Year)	Selection Bias	Study Design	Confounders	Blinding	Data collection methods	Withdrawals and drop-outs	Global Rating
**Population: Health Professionals**							
**Sabandüzen et al. 2024** [72]	Weak	Moderate	Weak	Weak	Weak	Strong	Weak
**Said et al. 2021** [73]	Weak	Moderate	Weak	Weak	Strong	Strong	Weak
**Salehi et al. 2023** [74]	Moderate	Moderate	Weak	Weak	Weak	Strong	Weak
**Sangwan et al. 2022** [75]	Moderate	Moderate	Weak	Weak	Moderate	Strong	Weak
**Shahrin et al. 2022** [76]	Moderate	Moderate	Weak	Weak	Weak	Strong	Weak
**Sharma et al. 2021** [77]	Weak	Moderate	Weak	Moderate	Weak	Weak	Weak
**Smith et al. 2023** [78]	Moderate	Moderate	Weak	Moderate	Weak	Moderate	Weak
**Strehlow et al. 2024** [79]	Moderate	Moderate	Weak	Weak	Weak	Weak	Weak
**Ta'an et al. 2023** [80]	Strong	Moderate	Weak	Weak	Strong	Strong	Weak
**Tan et al. 2020** [81]	Moderate	Moderate	Weak	Moderate	Weak	Weak	Weak
**Thakre et al. 2020a** [82]	Moderate	Moderate	Weak	Moderate	Weak	Strong	Weak
**Thakre et al. 2020b** [83]	Moderate	Moderate	Weak	Moderate	Weak	Strong	Weak
**Tsiouris et al. 2022** [84]	Moderate	Moderate	Weak	Moderate	Weak	Weak	Weak
**Wang et al. 2022** [85]	Weak	Moderate	Weak	Weak	Weak	Strong	Weak
**Wu et al. 2023** [86]	Moderate	Moderate	Weak	Weak	Weak	Moderate	Weak
**Zafar et al. 2020** [87]	Strong	Moderate	Weak	Weak	Weak	Strong	Weak
**Zhao et al. 2022** [88]	Weak	Moderate	Weak	Weak	Weak	Weak	Weak
**Heterogeneous Population: Health Professionals and Non-Health Professionals[a]**							
**Brito-Brito et al. 2021[b] [89]**	Moderate	Moderate	Weak	Weak	Weak	Strong	Weak
**Espinoza-Castro et al. 2025[c] [90]**	Moderate	Moderate	Weak	Weak	Weak	Weak	Weak
**Kaufman et al. 2024[c] [91]**	Weak	Moderate	Weak	Weak	Weak	Weak	Weak
**Kharel et al. 2022[d] [92]**	Weak	Moderate	Weak	Moderate	Weak	Weak	Weak
**Singh et al. 2021[e] [93]**	Weak	Moderate	Weak	Moderate	Weak	Strong	Weak

[a] The Risk of bias assessment based on results for health professionals and non-health professionals together, as results were not separately reported for both population groups.
[b] We assigned this study to the heterogeneous population as findings were not reported sparately for both population groups of educators and health professions.
[c] We assigned this study to the heterogeneous population as findings were not reported sparately for both population groups of health professionals and non-health professionals.
[d] We assigned this study to the heterogeneous population as included policy makers were not further specified.
[e] We assigned this study to the heterogeneous population as the professions of faculty members were not further specified.

References

1. Alotaibi N, Al-Sayegh N, Nadar M, Shayea A, Allafi A, Almari M. Investigation of Health Science Students' Knowledge Regarding Healthy Lifestyle Promotion During the Spread of COVID-19 Pandemic: A Randomized Controlled Trial. Frontiers in public health 2021;9:774678. doi:10.3389/fpubh.2021.774678
2. Amiri, B, Khajavian, N, Rahmani, R, Bilandi RR. Comparing the Impact of Multimedia and Educational Brochures on Knowledge, Attitude and Work Performance of Healthcare about COVID-19 Management in Pregnancy, Childbirth, and Breastfeeding. Iranian Red Crescent Medical Journal 2023;25(11). doi:10.32592/ircmj.2023.25.11.2498
3. Birrenbach T, Zbinden J, Papagiannakis G, Exadaktylos AK, Muller M, Hautz WE, Sauter TC. Effectiveness and Utility of Virtual Reality Simulation as an Educational Tool for Safe Performance of COVID-19 Diagnostics: Prospective, Randomized Pilot Trial. JMIR serious games 2021;9(4):e29586. doi:10.2196/29586
4. Christensen L, Rasmussen CS, Benfield T, Franc JM. A Randomized Trial of Instructor-Led Training Versus Video Lesson in Training Health Care Providers in Proper Donning and Doffing of Personal Protective Equipment. Disaster Medicine and Public Health Preparedness 2020;14(4):514-520. doi:10.1017/dmp.2020.56
5. Currat L, Suppan M, Gartner BA, Daniel E, Mayoraz M, Harbarth S, Suppan L, Stuby L. Impact of Face-to-Face Teaching in Addition to Electronic Learning on Personal Protective Equipment Doffing Proficiency in Student Paramedics: Randomized Controlled Trial. International journal of environmental research and public health 2022;19(5). doi:10.3390/ijerph19053077
6. Jeihooni AK, Namdari A, Kashfi SM, Kamyab A, Harsini PA, Rakhshani T. Effects of an educational intervention based on the health belief model on COVID-19 preventive behaviors among health personnel in Abadan, Iran. Journal of Public Health 2023. doi:10.1007/s10389-023-02053-6
7. Li Y, Wang Y, Li Y, Zhong M, Liu H, Wu C, Gao X, xia Z, Ma W. Comparison of Repeated Video Display vs Combined Video Display and Live Demonstration as Training Methods to Healthcare Providers for Donning and Doffing Personal Protective Equipment: A Randomized Controlled Trial. Risk Management and Healthcare Policy 2020;13:2325-2335. doi:10.2147/RMHP.S267514
8. Manggala SK, Tantri AR, Sugiarto A, Sianipar IR, Prasetyono TOH. In situ simulation training for a better interprofessional team performance in transferring critically ill patients with COVID-19: a prospective randomised control trial. Postgraduate medical journal 2022. doi:10.1136/postgradmedj-2021-141426
9. Rueda-Medina B, Aguilar-Ferrándiz ME, Esteban-Burgos AA, Tapia Haro RM, Casas-Barragán A, Velando-Soriano A, Gil-Gutiérrez R, Correa-Rodríguez M. Impact of Non-Face-to-Face Teaching with Passive Training on Personal Protective Equipment Use in Health Science Students: A Randomized Controlled Trial. International journal of environmental research and public health 2022;19(19). doi:10.3390/ijerph191912981
10. Suppan L, Abbas M, Stuby L, Cottet P, Larribau R, Golay E, Iten A, Harbarth S, Gartner B, Suppan M. Effect of an E-Learning Module on Personal Protective Equipment Proficiency Among Prehospital Personnel: Web-Based Randomized Controlled Trial. Journal of medical Internet research 2020;22(8):e21265. doi:10.2196/21265
11. Suppan L, Stuby L, Gartner B, Larribau R, Iten A, Abbas M, Harbarth S, Suppan M. Impact of an e-learning module on personal protective equipment knowledge in student paramedics: a randomized controlled trial. Antimicrobial Resistance & Infection Control 2020;9(1):185. doi:10.1186/s13756-020-00849-9
12. Wang XX, Zhou YZ, Song ZX, Wang YT, Chen XT, Zhang DD. Practical COVID-19 Prevention Training for Obstetrics and Gynecology Residents Based on the Conceive-Design-Implement-Operate Framework. Frontiers in public health 2022;10. doi:10.3389/fpubh.2022.808084
13. Xie M, Zhou Q, Kang Y, Qing P, Guo Y, Wei X, Cai B, Zeng J, Huang J. The Skill Training of Resident Anesthesiologists During the Outbreak Of COVID-19. ResearchSquare 2021. doi:10.21203/rs.3.rs-144144/v1
14. Jafree SR, Zakar R, Rafiq N, Javed A, Durrani RR, Burhan SK, Hasnain Nadir SM, Ali F, Shahid A, Momina AU, Wrona KJ, Mahmood QK, Fischer F. WhatsApp-Delivered Intervention for Continued Learning for Nurses in Pakistan During the COVID-19 Pandemic: Results of a Randomized-Controlled Trial. Frontiers in public health 2022;10:739761. doi:10.3389/fpubh.2022.739761
15. Rakhshani T, Dolatkhah SM, Kashfi SM, Khani Jeihooni A. The effect of a self-learned virtual learning package on knowledge, attitude, and self-care behaviors of COVID-19 in people referred to health and treatment centers. BMC Public Health 2024;24(1):1710. doi:10.1186/s12889-024-19233-y
16. Ansari A., Urooj U., Waseem M., Ihtasham A. Video based learning vs instructor led training for optimising personal protective equipment use to prevent Covid-19 infection-a comparative study. J Pak Med Assoc 2022;72(5):807-810. doi:10.47391/JPMA.3359
17. Buyego P, Katwesigye E, Kebirungi G, Nsubuga M, Nakyejwe S, Cruz P, McCarthy MC, Hurt D, Kambugu A, Arinaitwe JW, Ssekabira U, Jjingo D. Feasibility of virtual reality based training for optimising COVID-19 case handling in Uganda. BMC Medical Education 2022;22(1):274. doi:10.1186/s12909-022-03294-x
18. Hu H, Xiao YY, Li H. The Effectiveness of a Serious Game Versus Online Lectures for Improving Medical Students' Coronavirus Disease 2019 Knowledge. Games For Health Journal 2021;10(2):139-144. doi:10.1089/g4h.2020.0140
19. Yu M, Yang MR. Effectiveness and Utility of Virtual Reality Infection Control Simulation for Children With COVID-19: Quasi-Experimental Study. JMIR serious games 2022;10(2):e36707. doi:10.2196/36707
20. Abbas K, Nawaz SMA, Amin N, Soomro FM, Abid K, Ahmed M, Sayeed KA, Ghazanfar S, Qureshi N. A web-based health education module and its impact on the preventive practices of health-care workers during the COVID-19 pandemic. Health Education Research 2020;35(5):353-361. doi:10.1093/her/cyaa034
21. Ahmed NH, Tosson MM, Badia TS. Effect of educational program on maternity nurses' knowledge, attitude and practice of preventive measures towards COVID-19. Assiut Scientific Nursing Journal 2022;0(0):0. doi:10.21608/asnj.2022.116049.1301
22. Altillo BSA, Gray M, Avashia SB, Norwood A, Nelson EA, Johnston C, Bhavnani D, Patel H, Allen CH, Adeni S, Phelps ND, Mercer T. Global health on the front lines: an innovative medical student elective combining education and service during the COVID-19 pandemic. BMC Medical Education 2021;21(1):186. doi:10.1186/s12909-021-02616-9
23. Aqel O, Alqadheeb B, Felix M, Amundson C, Bingham JM, Meyer K, Warholak T, Axon DR. Cultivating COVID-19 Vaccine Confidence in Pharmacy Professionals. Pharmacy 2023;11(2). doi:10.3390/pharmacy11020050

24. Aujee D.S., Aghamkar J., Yangad S., Salvi R. ”Assessment of Educational Intervention On Knowledge Regarding World Health Organization Covid-19 Protocols During Delivery AmongStaff Nurses Working In Labour Room of Selected Hospitals. Journal of Pharmaceutical Negative results 2022;13:4717-4721. doi:10.47750/pnr.2022.13.S08.613
25. Bakhsh A, Asiri R, Alotaibi H, Alsaeedi R, Shahbar R, Boker A. Rapid cycle training for non-critical care physicians to meet intensive care unit staff shortage at an academic training center in a developing country during the COVID-19 pandemic. BMC Medical Education 2023;23(1):493. doi:10.1186/s12909-023-04478-9
26. Bayomi R, taha N. Effect of Teaching Guidelines on Knowledge, Attitudes, and Practices Regarding COVID19 among the First Year Nursing Students. Assiut Scientific Nursing Journal 2021;9(25):38-47. doi:10.21608/asnj.2021.72355.1156
27. Bechini A, Vannacci A, Salvati C, Crescioli G, Lombardi N, Chiesi F, Shtylla J, Del Riccio M, Bonanni P, Boccalini S. Knowledge and training of Italian students in Healthcare Settings on COVID-19 vaccines and vaccination strategies, one year after the immunization campaign. Journal of preventive medicine and hygiene 2023;64(2):E152-E160. doi:10.15167/2421-4248/jpmh2023.64.2.2934
28. Bieri J, Tuor C, Nendaz M, L Savoldelli G, Blondon K, Schiffer E, Zamberg I. Implementation of a Student-Teacher-Based Blended Curriculum for the Training of Medical Students for Nasopharyngeal Swab and Intramuscular Injection: Mixed Methods Pre-Post and Satisfaction Surveys. JMIR Medical Education 2023;9:e38870. doi:10.2196/38870
29. Blake H, Fecowycz A, Starbuck H, Jones W. COVID-19 Vaccine Education (CoVE) for Health and Care Workers to Facilitate Global Promotion of the COVID-19 Vaccines. International journal of environmental research and public health 2022;19(2). doi:10.3390/ijerph19020653
30. Boccalini S, Vannacci A, Crescioli G, Lombardi N, Del Riccio M, Albora G, Shtylla J, Masoni M, Guelfi MR, Bonanni P, Bechini A. Knowledge of University Students in Health Care Settings on Vaccines and Vaccinations Strategies: Impact Evaluation of a Specific Educational Training Course during the COVID-19 Pandemic Period in Italy. Vaccines 2022;10(7). doi:10.3390/vaccines10071085
31. Bohara A, Thapa S, Yilmaz SK, McBee SH. An Impact Evaluation of COVID-19 Training Program: Knowledge and Awareness of Public Health Professionals of Province Five, Nepal; 2021.
32. Calik A, Cakmak B, Kapucu S, Inkaya B. The effectiveness of serious games designed for infection prevention and promotion of safe behaviors of senior nursing students during the COVID-19 pandemic. American journal of infection control 2022. doi:10.1016/j.ajic.2022.02.025
33. Clay J, Morton K, Franz D, Jaqua E, Nguyen V. Quality Improvement for Outpatient COVID-19 Infection Control. Cureus 2021;13(7):e16373. doi:10.7759/cureus.16373
34. Díaz-Guio DA, Ricardo-Zapata A, Ospina-Velez J, Gómez-Candamil G, Mora-Martinez S, Rodriguez-Morales AJ. Cognitive load and performance of health care professionals in donning and doffing PPE before and after a simulation-based educational intervention and its implications during the COVID-19 pandemic for biosafety. Le Infezioni in Medicina 2020:111-117.
35. Elasrag GAEA, Elsabagh NE, Abdelmonem AF, Ahmed A. Impact of Educational Intervention on Nurses’ Knowledge, Practice and Attitude Related Prevention Measures of COVID 19. IJFMT 2021. doi:10.37506/ijfmt.v15i3.15751
36. Etebarian A, Tusi SK, Momeni Z, Hejazi K. Impact of educational intervention regarding COVID-19 on knowledge, attitude, and practice of students before dental school re-opening. BMC Oral Health 2023;23(1):1-6. doi:10.1186/s12903-023-02845-y
37. Fadel EA, Alshawish E, El-Shaboury RHR, Khalil DE, Mahmoud FZ, El-Feshawy NI. Effect of Implementing Virtual Educational Sessions on Nursing Students’ Knowledge, Attitude and Hesitancy Regarding COVID-19 Vaccination. Inquiry: a journal of medical care organization, provision and financing 2025;62:469580251339114. doi:10.1177/00469580251339114
38. Findyartini A, Greviana N, Hanum C, Husin JM, Sudarsono NC, Krisnamurti DGB, Rahadiani P. Supporting newly graduated medical doctors in managing COVID-19: An evaluation of a Massive Open Online Course in a limited-resource setting. PLOS ONE 2021;16(9). doi:10.1371/journal.pone.0257039
39. Fuentes GM, Carbajales León EB, Carbajales León AI. Educative intervention about COVID-19 in the Medicine students from Joaquín de Agüero Policlinic. Revista Electrónica Medimay 2020;27(3):366-376.
40. Garcia KS, Rodriguez A, Gonzalez Z, Armstrong C, Iacob E, Flynn EE, Simmons M. Pretest-post-test evaluation with lay midwives in remote Guatemala after educational activities about COVID-19. Rural and Remote Health 2024;24(3):8387. doi:10.22605/RRH8387
41. Girard H., Bosshard W., Krief H., Bula C.J. AO - Bula, Christophe J. Effectiveness of Information Sessions About COVID-19 Vaccines in Healthcare Professionals Working in Geriatrics. Gerontology and Geriatric Medicine 2022;8. doi:10.1177/23337214221115235
42. Greaves SW, Alter SM, Ahmed RA, Hughes KE, Doos D, Clayton LM, Solano JJ, Echeverri S, Shih RD, Hughes PG. A Simulation-based PPE orientation training curriculum for novice physicians. Infection Prevention in Practice 2023;5(1). doi:10.1016/j.infpip.2022.100265
43. Gupta S, Goswami B, Madhu SV. Effectiveness of Video-Based Educational Intervention on Knowledge, Attitude, and Practice (KAP) of COVID-19 Health Care Workers: Lesson for Future Pandemic Preparedness. ANAMS 2023;59:233-236. doi:10.1055/s-0043-1772218
44. Halemani K, Cheema M, Khatun S, Yadidya, Singh B, Gupta V K, Sharma A. An effectiveness of training program on COVID-19 among healthcare students: A cross section study. International Journal of Research in Pharmaceutical Sciences 2020;11(13):1250-1254. doi:10.26452/ijrps.v11iSPL1.3613
45. Han B, Zang F, Liu J, Li S, Zhang W, Zhang Y, Li Z. Effect Analysis of “Four-Step” Training and Assessment Tool in the Prevention and Control of COVID-19. Infection and drug resistance 2022;15:1247-1257. doi:10.2147/IDR.S346559
46. Hwang WJ, Lee J. Effectiveness of the Infectious Disease (COVID-19) Simulation Module Program on Nursing Students: Disaster Nursing Scenarios. Journal of Korean Academy of Nursing 2021;51(6):648-660. doi:10.4040/jkan.21164
47. Instrum RS, Koch RW, Rocha T, Rohani SA, Ladak H, Agrawal SK, Sowerby LJ. Improving Nasopharyngeal Swab Technique via Simulation for Frontline Workers. The Laryngoscope 2022. doi:10.1002/lary.30034
48. Kasapoglu ES, Yildiz YS, Saldamli A, Karaçetin F. The effect of COVID-19 patient care and emergency response interprofessional training on COVID-19 knowledge, perception, behavior and readiness for care. WORK-A JOURNAL OF PREVENTION ASSESSMENT & REHABILITATION 2023;75(3):767-778. doi:10.3233/WOR-220227
49. Khari S, Pazokian M, Abadi AS, Zarmehrparirouy M, Ahmadvand Y. The Effect of E-Learning Program for COVID-19 Patient Care on the Knowledge of Nursing Students: A Quasi-Experimental Study. SAGE Open Nursing 2022;8. doi:10.1177/23779608221124421
50. Kobayashi D, Mami K, Fujishiro S, Nukanobu N, Ueno SI, Kuwakado S, Koyama T, Kuga H. Online training of Covid-19 infection prevention and control for healthcare workers in psychiatric institutes. BMC Psychiatry 2023;23(1). doi:10.1186/s12888-023-04826-5

51. Kufel WD, Blaine BE, Avery LM. Pharmacy students' knowledge and confidence of COVID-19 following an interactive didactic class. Journal Of The American College Of Clinical Pharmacy 2022;5(10):1082-1087. doi:10.1002/jac5.1678
52. Lalitha ND, Bhadauria US, Agarwal D, Purohit BM, Priya H, Nilima N, Duggal R, Mathur VP, Logani A. Comparing the effectiveness of two educational methods for oral health management in COVID-19 pandemic among dental professionals. Przegl Epidemiol 2024;78(1):90-93. PMID:38904315
53. Li Z, Cheng J, Zhou T, Wang S, Huang S, Wang H. Evaluating a Nurse Training Program in the Emergency Surgery Department Based on the Kirkpatrick's Model and Clinical Demand During the COVID-19 Pandemic. Telemedicine journal and e-health : the official journal of the American Telemedicine Association 2020;26(8):985-991. doi:10.1089/tmj.2020.0089
54. Macht L, Worlitzsch D, Braijoshri N, Bequiri P, Zudock J, Zilezinski M, Stoevesandt D, Smith J, Hofstetter S. COVID-19: Development and implementation of a video-conference-based educational concept to improve the hygiene skills of health and nursing professionals in the Republic of Kosovo. GMS Hygiene and Infection Control 2022;17. doi:10.3205/dgkh000412
55. Mark ME, LoSavio P, Husain I, Papagiannopoulos P, Batra PS, Tajudeen BA. Effect of Implementing Simulation Education on Health Care Worker Comfort With Nasopharyngeal Swabbing for COVID-19. Otolaryngology-head and neck surgery: Official journal of American Academy of Otolaryngology-Head and Neck Surgery 2020;163(2):271-274. doi:10.1177/0194599820933168
56. McConnell H, Duncan D, Stark P, Anderson T, McMahon J, Creighton L, Craig S, Carter G, Smart A, Alanazi A, Mitchell G. Enhancing COVID-19 Knowledge among Nursing Students: A Quantitative Study of a Digital Serious Game Intervention. Healthcare (Basel) 2024;12(11). doi:10.3390/healthcare12111066
57. Mektrirat R, Sathanawongs A, Tiwananthagorn S, Chaisowwong W, Peansukmanee S, Naksen W, Thongprachum A. Achieving Interprofessional Education on Collaborative Problem-Solving for COVID-19 Using Project-Based Approach. International Journal of Infectious Diseases 2021;116:S67-. doi:10.1016/j.ijid.2021.12.158
58. Mohamed Y, Hezeri P, Kama H, Mills K, Walker S, Hau'ofa N, Amol C, Jones M, du Cros P, Lin YD. Evaluation of an Online Training Program on COVID-19 for Health Workers in Papua New Guinea. Tropical medicine and infectious disease 2023;8(6). doi:10.3390/tropicalmed8060327
59. Nassar AAH, Al Serouri AA, Al-Shahethi AH, Almoayed KA. Effectiveness of training on health care workers' knowledge, attitude and practice regarding COVID-19 infection prevention and control, Yemen, 2021. BMC Health Services Research 2024;24(1):1411. doi:10.1186/s12913-024-11927-8
60. Naz F, Ohri P, Sharma A, Spandana BS, Gupta K. Impact of Training on Awareness of COVID-19 among The Health Care Workers in A Tertiary Care Hospital of Dehradun. Indian Journal of Community Health 2022;34(1):20-25. doi:10.47203/IJCH.2022.v34i01.005
61. Odusanya OO, Adeniran A, Bakare OQ, Odugbemi BA, Enikuomehin OA, Jeje OO, Emechebe AC. Building capacity of primary health care workers and clients on COVID-19: Results from a web-based training. PLOS ONE 2022;17(10):e0274750. doi:10.1371/journal.pone.0274750
62. Otu A, Okuzu O, Effa E, Ebenso B, Ameh S, Nihalani N, Onwusaka O, Tawose T, Olayinka A, Walley J. Training health workers at scale in Nigeria to fight COVID-19 using the InStrat COVID-19 tutorial app: an e-health interventional study. Therapeutic advances in infectious disease 2021;8:20499361211040704. doi:10.1177/20499361211040704
63. Otu A, Okuzu O, Ebenso B, Effa E, Nihalani N, Olayinka A, Yaya S. Introduction of Mobile Health Tools to Support COVID-19 Training and Surveillance in Ogun State Nigeria. Front. Sustain. Cities 2021;3. doi:10.3389/frsc.2021.638278
64. Perera N, Haldane V, Ratnapalan S, Samaraweera S, Karunathilake M, Gunarathna C, Bandara P, Kawirathne P, Wei XL. Implementation of a coronavirus disease 2019 infection prevention and control training program in a low-middle income country. JBI Evidence Implementation 2022;20(3):228-235. doi:10.1097/XEB.0000000000000307
65. Pokrajac N, Schertzer K, Poffenberger CM, Alvarez A, Marin-Nevarez P, Winstead-Derlega C, Gisondi MA. Mastery Learning Ensures Correct Personal Protective Equipment Use in Simulated Clinical Encounters of COVID-19. West J Emerg Med 2020;21(5):1089-1094. doi:10.5811/westjem.2020.6.48132
66. Puga RR, Cardoso AL, Rodríguez OL. Educational intervention in medical students on post-COVID-19 disabling sequelae from the Public Health subject. Revista Cubana De Reumatologia 2023;25(3).
67. Qasmi SA, Standley C, Mohsin S, Sarwar S, Malik L, Aziz F. Effectiveness of international virtual training on biorisk management in the context of COVID-19. Frontiers in public health 2022;10. doi:10.3389/fpubh.2022.888097
68. Roberts EN, Smithing RT, Tucker P. Measuring the impact of a COVID-19 continuing education program. Journal of the American Association of Nurse Practitioners 2022;34(6):835-843. doi:10.1097/JXX.0000000000000715
69. Roberts KJ, Zumstein KK, Lamphere TR, Williams M, Powell SA, Moran A, Kellar B, Solly WR, Pierce M. Improving Students' Knowledge and Skills Through a Tele-ICU Clinical Rotation. Respiratory Care 2022;67(7):789-794. doi:10.4187/respcare.09896
70. Rosas-Magallanes C, Basto-Abreu A, Barrientos-Gutiérrez T, Ramírez-Martínez JL, Tamayo-Ortiz M, Gutiérrez-Díaz HO, Magaña-Valladares L, Cordera DB, Santamaría-Guasch CM, Hernández-Avila M. CLIMSS online platform as a health literacy tool during the health crisis of Covid-19. Salud Publica De Mexico 2022;64(3):320-327. doi:10.21149/13103
71. Saati AA, Alkalash SH. Promotion of knowledge, attitude, and practice among medical undergraduates regarding infection control measures during COVID-19 pandemic. Frontiers in public health 2022;10. doi:10.3389/fpubh.2022.932465
72. Sabandüzen H, Kavaklı Ö. Evaluation of the effectiveness of the training on "Home care of COVID-19 positive/suspicious patients" given to nursing students: A quasi-experimental study. Journal of education and health promotion 2024;13:250. doi:10.4103/jehp.jehp_1574_23
73. Said AR. Knowledge and Practices of Nurses Regarding Corona Virus (COVID-19): An Educational Intervention. MLU 2021;21(2):36-47. doi:10.37506/mlu.v21i2.2642
74. Salehi R, Young S de, Asamoah A, Aryee SE, Eli R, Couper B, Smith B, Djokoto C, Agyeman YN, Zakaria AF, Butt N, Boadu A, Nyante F, Merdiemah G, Oliver-Commey J, Ofori-Boadu L, Akoriyea SK, Parry M, Fiore C, Okae F, Adams A, Acquah H. Evaluation of a continuing professional development strategy on COVID-19 for 10 000 health workers in Ghana: a two-pronged approach. Human resources for health 2023;21(1). doi:10.1186/s12960-023-00804-w
75. Sangwan J, Lathwal S, Lohan K, Yadav K, Adlakha N, Mane P, Gole S. Impact of training on Knowledge, Attitude and Perceived Barriers for Compliance Regarding use of Protective Equipment Kit among Frontline Healthcare Workers during COVID-19 Pandemic. J Clin Diagn Res 2022;16(1):JC17-JC21. doi:10.7860/JCDR/2022/50845.15913
76. Shahrin L, Parvin I, Sarmin M, Abbassi NA, Ackhter MM, Alam T, Mamun GMS, Rahman A, Shaima SN, Shikha SS, Jeorge DH, Nahar MA, Sharifuzzaman, Saha H, Rahman ASMMH, Shahid ASMSB, Faruque ASG, Ahmed T, Chisti MJ. In-person training on COVID-19

case management and infection prevention and control: Evaluation of healthcare professionals in Bangladesh. PLOS ONE 2022;17(10):e0273809. doi:10.1371/journal.pone.0273809
77. Sharma R, Mohanty A, Singh V, S VA, Gupta PK, Jelly P, Gupta P, Rao S. Effectiveness of Video-Based Online Training for Health Care Workers to Prevent COVID-19 Infection: An Experience at a Tertiary Care Level Institute, Uttarakhand, India. Cureus 2021;13(5):e14785. doi:10.7759/cureus.14785
78. Smith CR, Vasilopoulos T, am Frantz, LeMaster T, Martinez RA, am Gunnett, Fahy BG. Staying proper with your personal protective equipment: How to don and doff. Journal Of Clinical Anesthesia 2023;86. doi:10.1016/j.jclinane.2023.111057
79. Strehlow MC, Johnston JS, Aluri KZ, Prober CG, Acker PC, Patil AS, Mahadevan A, Mahadevan SV. Evaluation of a massive open online course for just-in-time training of healthcare workers. Frontiers in public health 2024;12:1395931. doi:10.3389/fpubh.2024.1395931
80. Ta'an WF, Al-Hammouri MM, Al-Faouri I, Suliman MM. The effectiveness of COPA-based training program on the infection- control competencies of newly hired healthcare professionals. Teaching and Learning in Nursing 2023;18(1):160-165. doi:10.1016/j.teln.2022.06.009
81. Tan W, Ye Y, Yang Y, Chen Z, Yang X, Zhu C, Chen D, Tan J, Zhen C. Whole-Process Emergency Training of Personal Protective Equipment Helps Healthcare Workers Against COVID-19: Design and Effect. J Occup Environ Med 2020;62(6):420-423. doi:10.1097/JOM.0000000000001877
82. Thakre SS, Jadhao AR, Dhoble MA, Dass R, Thakre SB, Somani A. Evaluation of Effectiveness of Covid-19 Training and Assessment of Anxiety among Nurses of a Tertiary Health Care Center during the Corona Virus Pandemic-An Experimental Study. Journal Of Clinical and Diagnostic Research 2020;14(11):LC34-LC37. doi:10.7860/JCDR/2020/45464.14301
83. Thakre SS, Thakre SB, Jadhao A, Dass R, Dhoble MA, Tiwari PN. Evaluation of effectiveness of COVID-19 training of tertiary health care workers. Int J Community Med Public Health 2020;7(7):2635. doi:10.18203/2394-6040.ijcmph20202989
84. Tsiouris F, Hartsough K, Poimbouef M, Raether C, Farahani M, Ferreira T, Kamanzi C, Maria J, Nshimirimana M, Mwanza J, Njenga A, Odera D, Tenthani L, Ukaejiofo O, Vambe D, Fazito E, Patel L, Lee C, Michaels-Strasser S, Rabkin M. Rapid scale-up of COVID-19 training for frontline health workers in 11 African countries. Human resources for health 2022;20(1):43. doi:10.1186/s12960-022-00739-8
85. Wang SH, Yimer G, Bisesi M, Lisawork L, Sugerman D, Alayu M, Wossen M, Abayneh SA, Gallagher K, Endashaw T, Kubinson H, Kanter T, Gallagher K, Gebreyes W. Rapid virtual training and field deployment for COVID-19 surveillance officers: experiences from Ethiopia. Pan African medical journal 2022;43:23. doi:10.11604/pamj.2022.43.23.28787
86. Wu TY, Hoffman JL, Chow CM, Hartl B. Training community health navigators in the public health workforce to respond during the COVID-19 pandemic. Z Gesundh Wiss 2023:1-8. doi:10.1007/s10389-022-01812-1
87. Zafar N, Jamal Z, Mujeeb Khan M. Preparedness of the Healthcare Personnel Against the Coronavirus Disease 2019 (COVID-19) Outbreak: An Audit Cycle. Frontiers in public health 2020;8:502. doi:10.3389/fpubh.2020.00502
88. Zhao J, Rozenberg D, Kaul R, Sanh M, Luther R, Orchanian-Cheff A, Nourouzpour S, de Peiza P, Agbeyaka S, Gebara N, Doumouras AM, Draper H, Barber M, Lau J, Furlan A. The positive impact of a telemedicine education program on healthcare workers during the COVID-19 pandemic in Ontario, Canada. The Annals of Family Medicine 2022(20). doi:10.1370/afm.20.s1.3260
89. Brito-Brito PR, Fernandez-Gutierrez DA, Martinez-Alberto CE, Saez-Rodriguez MJ, Nunez-Marrero J, Garcia-Hernandez AM. Use of the Nursing Outcomes Classification (NOC) to measure perceived knowledge about the control of SARS-CoV-2 infection: The impact of a training program in primary healthcare professionals. International journal of nursing knowledge 2021. doi:10.1111/2047-3095.12356
90. Espinoza-Castro B, Encina V, Garrido MA, Vinueza FI, Piedra JP, Garzon-Villalba X, Radon K. Online learning for crisis response: evaluating reach and perceived knowledge gains from the MOOC "Infection, Prevention, and Control of Acute Respiratory Infections for Healthcare Workers in Low- and Middle-Income Countries (IPC MOOC)". BMC Medical Education 2025;25(1):1150. doi:10.1186/s12909-025-07661-2
91. Kaufman J, Overmars I, Fong J, Tudravu J, Devi R, Volavola L, Vodonaivalu L, Jenkins K, Leask J, Seale H, Mohamed Y, Joshi K, Datt H, Sagan S, Dynes M, Hoq M, Danchin M. Training health workers and community influencers to be Vaccine Champions: a mixed-methods RE-AIM evaluation. BMJ Global Health 2024;9(9). PMID:39251236
92. Kharel R, Baird J, Vaishnav H, Chillara N, Lee JA, Genisca A, Hayward A, Uzevski V, Elbenni A, Levine AC, Aluisio AR. Development and assessment of novel virtual COVID-19 trainer-of trainers course implemented by an academic-humanitarian partnership. Global health action 2022;15(1):2010391. doi:10.1080/16549716.2021.2010391
93. Singh V, Supehia S, Gupta PK, Narula H, Sharma M, Devi K, Bhute AR. Effectiveness of video modules in infection control trainings during COVID-19 pandemic: A quasi-experimental study in tertiary care institute. Journal of education and health promotion 2021;10(1):183. doi:10.4103/jehp.jehp_1009_20
