# Supplementary material for: Interventions to Enhance COVID-19 Pandemic Health Literacy in Health Professionals: Systematic Review
Source: JMIR Med Educ. 2026 Jul 10;12:e70400. doi: 10.2196/70400 (PMC13360183; doi:10.2196/70400)
Supplement: Multimedia Appendix 4 — Summary of findings tables. [file mededu-v12-e70400-s004.pdf]

Table S1. Summary of Findings in Randomised Controlled Trials – Primary Outcomes

**Interventions for enhancing COVID-19 related health literacy in health professionals**				
**Population: Health professionals of various professions**<br>**Setting: Various health care settings**<br>**Intervention: Various interventions**<br>**Comparison: Various comparator**				
**Study (Year)**	**Intervention**	**Comparator**	**Relative effect**	**No. of Participants[a] (n Studies)**
**Primary Outcomes aimed at enhancing COVID-19 related knowledge**				
**General COVID-19 related knowledge (Follow Up 1)**				**250 (1 Study)**
**Jeihooni et al. 2023** [1]	Health belief model-oriented training package	Delayed intervention<br>(six month later)	**Mean Score (SD)**<br>***IG*** 37.13 (1.98)<br>***CG*** 16.25 (1.38)<br>*P*=.001	250<br>(*IG* 125, *CG* 125)
**COVID-19 specific vaccine knowledge (Follow Up 1)**				**155 (1 Study)**
**Alotaibi et al. 2021** [2]	**Intervention 1**<br>Written information (Brochure)<br>**Intervention 2**<br>Written information via Instagram<br>**Intervention 3**<br>Synchronous online interactive educational workshop	Placebo	**Mean Score (SD)**<br>***Brochure*** 20.5 (4.1), *P*=.001<br>***Instagram*** 21.1 (4.1), *P*=.001<br>***Workshop*** 22.5 (3.8), *P*=.0001<br>***CG*** 17.2 (4.3), *P*=.004<br>**Effect size within groups**<br>***Brochure*** 1.13<br>***Instagram*** 1.23<br>***Workshop*** 1.47<br>***CG*** 0.60	155<br>(*Brochure* 30, *Instagram* 32, *Workshop* 43, *CG* 50)
**COVID-19 specific infection prevention knowledge on PPE (Follow Up 1)**				**399 (4 Studies)**
**Currat et al. 2022** [3]	Interactive gamified e-learning module & face-to-face workshop	Interactive gamified e-learning module	**Proportion of correct performances**<br>***IG*** 66.7% (95% CI 48.2-82.0)<br>***CG*** 64.5% (95% CI 45.4-80.8)<br>*P*=1	64<br>(*IG* 33, *CG* 31)
**Suppan et al. 2020a** [4]	Gamified e-learning module & COVID-19 Guideline	COVID-19 guidelines only	**Median of difference in proportions of correct choice at pre and post (Fisher exact test)**<br>***IG*** 17% (IQR 8-33)<br>***CG*** 8% (IQR 8-33)<br>*P*=.27	173<br>(*IG* 88, *CG* 85)
**Suppan et al. 2020b** [5]	Gamified e-learning module & COVID-19 Guideline	COVID-19 guidelines only	**Median of difference in proportions of correct choice at pre and post (Fisher exact test)**<br>***IG*** 33% (IQR 0-58)<br>***CG*** 17% (IQR -17-42)<br>*P*=.087	90<br>(*IG* 41, *CG* 49)
**Xie et al. 2021** [6]	Live streaming demonstration & Video feedback	Independent learning	**Mean Score (SD)**<br>***IG*** 81.88 (11.25)<br>***CG*** 80.95 (11.64)<br>*P*=.731	72<br>(*IG* 34, *CG* 38)
**COVID-19 related knowledge on IPC measures (not further specified, Follow Up 1)**				**38 (1 Study)**
**Wang et al. 2022** [7]	Conceive-design-implement-operate professional training model	Traditional Training based on protection theory exposition	**Mean Score (SD)**<br>***IG*** 92.76 (4.38), ***CG*** NA<br>*t*=5.861, *P*<.001	38<br>(*IG* 20, *CG* 18)
**COVID-19 specific knowledge on providing services in women during pregnancy, childbirth, and breast-feeding (Follow Up 1)**				**90 (1 Study)**
**Amiri et al. 2023** [8]	Educational programs for healthcare providers regarding COVID-19 management (instructions and protocols sent by the	Educational content in a routine format	**Mean Score (SD)**<br>***IG1*** 18.43 (1.98),<br>***IG2*** 16.47 (3.37),<br>***CG*** 10.8 (4.89)<br>*P*<.001	90<br>(*IG1* 30, *IG2* 30, *CG* 30)

Interventions for enhancing COVID-19 related health literacy in health professionals				
**Population: Health professionals of various professions**<br>**Setting: Various health care settings**<br>**Intervention: Various interventions**<br>**Comparison: Various comparator**				
**Study (Year)**	**Intervention**	**Comparator**	**Relative effect**	**No. of Participants[a] (n Studies)**
	Ministry of Health to universities)<br>**Intervention 1**: via multimedia (audio, video, written content, animation)<br>**Intervention 2**: via booklet (PDF format)			
**COVID-19 specific knowledge on providing services in women during pregnancy, childbirth, and breast-feeding (Follow Up 2)**				**90 (1 Study)**
**Amiri et al. 2023 [8]**	Educational programs for healthcare providers regarding COVID-19 management (instructions and protocols sent by the Ministry of Health to universities)<br>**Intervention 1**: via multimedia (audio, video, written content, animation)<br>**Intervention 2**: via booklet (PDF format)	Educational content in a routine format	**Mean Score (SD)**<br>***IG1*** 18.43 (1.98),<br>***IG2*** 16.47 (3.37),<br>***CG*** 10.8 (4.89)<br>*P*<.001	90<br>(*IG1* 30, *IG2* 30, *CG* 30)
**Primary outcomes aimed at enhancing COVID-19 related infection prevention performance skills**				
**PPE performance of Donning & Doffing (Follow Up 1)**				**300 (4 Studies)**
**Li et al. 2020 [9]**	Repeated Video Display (*Group A*)<br>Video Display & Live Demonstration (*Group B*)	None	**Mean Score (SD)**<br>***Group A*** 86.63 (6.34)<br>***Group B*** 94.92 (1.72)<br>*P*<.001	48<br>(*IG* 24, *CG* 24)
**Rueda-Medina et al. 2022 [10]**	Face-to-Face Teaching with Active Training	Non-Face-to-Face Teaching with Passive Training	**Mean Score (SD)**<br>***IG*** 1.53 (1.78)<br>***CG*** 2.23 (1.99)<br>*P*=.029	142<br>(*IG* 72, *CG* 70)
**Wang et al. 2022 [7]**	Conceive–design–implement–operate (CDIO) professional training model	Traditional Training based on protection theory exposition	**Mean Score (SD)**<br>***IG*** 87.33 (4.90)<br>***CG*** 80.84 (2.65)<br>*t*=4.996, *P*<.001	38<br>(*IG* 20, *CG* 18)
**Xie et al. 2021 [6]**	Live streaming demonstration & Video feedback	Independent learning	**Mean Score (SD)**<br>***IG*** 86.94 (4.19)<br>***CG*** 66.84 (10.49)<br>*P*<.001	72<br>(*IG* 34, *CG* 38)
**PPE performance of Donning (Follow Up 1)**				**117 (3 Studies)**
**Christensen et al. 2020 [11]**	Informational Video Training	Live Demonstration	**Mean Score (SD)**<br>***IG*** 88.0%<br>***CG*** 84.8%<br>95% CI -7.7to -9.5), *P*=.54	19<br>(*IG* 10, *CG* 9)
**Manggala et al. 2022 [12]**	Simulation based training (High fidelity)	Simulation based training (Low fidelity)	**Mean Score (SD)**<br>***IG*** 86.54 (13.92)<br>***CG*** 77.95 (12.49), *P*=.287	8<br>(*IG* 4, *CG* 4)
**Suppan et al. 2020b [5]**	Gamified e-learning module & COVID-19 Guideline	COVID-19 guidelines only	**Median of difference in proportions of correct answers at pre and post**<br>***IG*** 7.7% (95% CI 2.2-13.2)<br>***CG*** similar rate (not explicitely reported)<br>*P*=.571	90<br>(*IG* 41, *CG* 49)

Interventions for enhancing COVID-19 related health literacy in health professionals				
**Population: Health professionals of various professions**<br>**Setting: Various health care settings**<br>**Intervention: Various interventions**<br>**Comparison: Various comparator**				
**Study (Year)**	**Intervention**	**Comparator**	**Relative effect**	**No. of Participants[a] (n Studies)**
**PPE performance of Doffing (Follow Up 1)**				**210 (5 Studies)**
**Birrenbach et al. 2021 [13]**	VR Simulation	Traditional learning methods	**Median of number of contaminated areas**<br>***IG*** 2 (IQR 2-4)<br>***CG*** 3 (IQR 1-4), *P*=.64	29<br>(*IG* 15, *CG* 14)
**Christensen et al. 2020 [11]**	Informational Video Training	Live Demonstration	**Mean Score (SD)**<br>***IG*** 73.9%<br>***CG*** 79.1%<br>(95% CI -7.6 to -18.0), *P*=.54	19<br>(*IG* 10, *CG* 9)
**Currat et al. 2022 [3]**	Interactive gamified e-learning module & face-to-face workshop	Interactive gamified e-learning module	**Proportion of correct performances**<br>***IG*** 33.3% (95% CI 18.0-51.8.)<br>***CG*** 9.7% (95% CI 2.0-25.8)<br>*P*=.03	64<br>(IG 33, CG 31)
**Manggala et al. 2022 [12]**	Simulation based training (High fidelity)	Simulation based training (Low fidelity)	**Mean Score (SD)**<br>***IG*** 85.83 (8.01)<br>***CG*** 48.33 (23.80)<br>*P*=.010	8<br>(*IG* 4, *CG* 4)
**Suppan et al. 2020b [5]**	Gamified e-learning module & COVID-19 Guideline	COVID-19 guidelines only	Study reported that no participant was able to correctly describe the doffing sequence	90<br>(*IG* 41, *CG* 49)
**PPE performance of Doffing (Follow Up 2)**				**93 (2 Studies)**
**Birrenbach et al. 2021 [13]**	VR Simulation	Traditional learning methods	**Median of number of contami-nated areas**<br>***IG*** 1 (IQR 0-2)<br>***CG*** 0 (IQR 0-1)<br>*P*=.18	29<br>(*IG* 15, *CG* 14)
**Currat et al. 2022 [3]**	Interactive gamified e-learning module & face-to-face workshop	Interactive gamified e-learning module	**Proportion of correct performances**<br>***IG*** 33.3% (95%CI 18.0-51.8)<br>***CG*** 9.7% (95%CI 2.0-25.8)<br>*P*=.03	64<br>(*IG* 33, *CG* 31)
**Hand desinfection performance (Follow Up 1)**				**93 (2 Studies)**
**Birrenbach et al. 2021 [13]**	VR Simulation	Traditional learning methods	**Median of number of mistakes**<br>***IG*** 7 (IQR 4-14)<br>***CG*** 10 (IQR 6-14)<br>*P*=.34	29<br>(*IG* 15, *CG* 14)
**Currat et al. 2022 [3]**	Interactive gamified e-learning module & face-to-face workshop	Interactive gamified e-learning module	**Proportion of correct performances**<br>***IG*** 63.6% (95% CI 45.1-79.6)<br>***CG*** 45.2% (95% CI 27.3-64.0)<br>*P*=.21	64<br>(*IG* 33, *CG* 31)
**Hand desinfection performance (Follow Up 2)**				**93 (2 Studies)**
**Birrenbach et al. 2021 [13]**	VR Simulation	Traditional learning methods	**Median of number of mistakes**<br>***IG*** 14 (IQR 8-17)<br>***CG*** 11 (IQR 7-16)<br>*P*=.74	29<br>(*IG* 15, *CG* 14)
**Currat et al. 2022 [3]**	Interactive gamified e-learning module & face-to-face workshop	Interactive gamified e-learning module	**Proportion of correct performances**<br>***IG*** 57.6% (95% CI 39.2-74.5)<br>***CG*** 41.9% (95% CI 24.5-60.9)<br>*P*=.32	64<br>(*IG* 33, *CG* 31)

Interventions for enhancing COVID-19 related health literacy in health professionals				
**Population: Health professionals of various professions**<br>**Setting: Various health care settings**<br>**Intervention: Various interventions**<br>**Comparison: Various comparator**				
**Study (Year)**	**Intervention**	**Comparator**	**Relative effect**	**No. of Participants[a] (n Studies)**
**Nasopharyngeal swab performance (Follow Up 1)**				**29 (1 Study)**
**Birrenbach et al. 2021 [13]**	VR Simulation	Traditional learning methods	**Median score**<br>***IG*** 14 (IQR 13-15)<br>***CG*** 12 (IQR 11-14)<br>*P*=.03	29<br>(*IG* 15, *CG* 14)
**Nasopharyngeal swab performance (Follow Up 2)**				**29 (1 Study)**
**Birrenbach et al. 2021 [13]**	VR Simulation	Traditional learning methods	**Median score**<br>***IG*** 14 (IQR 14-16)<br>***CG*** 14 (IQR 14-15), *P*=.79	29<br>(*IG* 15, *CG* 14)
**COVID-19 related performance skills in providing services in women during pregnancy, childbirth, and breastfeeding (Follow Up 1)**				**90 (1 Study)**
**Amiri et al. 2023 [8]**	Educational programs for healthcare providers regarding COVID-19 management (instructions and protocols sent by the Ministry of Health to universities)<br>**Intervention 1**: via multimedia (audio, video, written content, animation)<br>**Intervention 2**: via booklet (PDF format)	Educational content in a routine format	**Mean Score (SD)**<br>***IG1*** 20.2 (1.56),<br>***IG2*** 18.57 (2.42),<br>***CG*** 15.6 (3.41)<br>*P*<.001	90<br>(*IG1* 30, *IG2* 30, *CG* 30)
**COVID-19 related performance skills in providing services in women during pregnancy, childbirth, and breastfeeding (Follow Up 2)**				**90 (1 Study)**
**Amiri et al. 2023 [8]**	Educational programs for healthcare providers regarding COVID-19 management (instructions and protocols sent by the Ministry of Health to universities)<br>**Intervention 1**: via multimedia (audio, video, written content, animation)<br>**Intervention 2**: via booklet (PDF format)	Educational content in a routine format	**Mean Score (SD)**<br>***IG1*** 20.73 (2.35),<br>***IG2*** 19.23 (3.18),<br>***CG*** 16.23 (3.65)<br>*P*<.001	90<br>(*IG1* 30, *IG2* 30, *CG* 30)

CI Confidence interval, **ICU** Intensive Care Unit, **IG** Intervention Group, **IPC** Infection prevention and control, **CG** Control Group, **PPE** Personal protective equipment, **NA** Not available, [a] Numbers of participants contributing to the analysis of each outcome

Table S2. Summary of Findings in Randomised Controlled Trials – Secondary Outcomes

Interventions for enhancing COVID-19 related health literacy in health professionals				
Population: Health professionals of various professions<br>Setting: Various health care settings<br>Intervention: Various interventions<br>Comparison: Various comparator				
**Study (Year)**	**Intervention**	**Comparator**	**Relative effect**	**Participants[a] (Studies)**
**Secondary Outcomes aimed at enhancing COVID-19 related knowledge**				
**COVID-19 related knowledge and perceived abilites (Follow up 1)** *Composite outcome*				**208 (1 Study)**
Jafree et al. 2022 [14]	Booklet & Whats App based intervention	Only booklet	**Adjusted mean difference**<br>0.02 (95% CI −1.63 to 1.69)<br>*P*=.063<br>**Mean Score (SD)**<br>***IG*** 34.97 (6.13)<br>***CG*** 33.13 (3.47)	208<br>(*IG* 106, *CG* 102)
**Secondary outcomes aimed at enhancing COVID-19 related confidence**				
**Perceived confidence in the ability of using PPE (Follow Up 1)**				**375 (4 Studies)**
Currat et al. 2022 [3]	Interactive gamified e-learning module & face-to-face workshop	Interactive gamified e-learning module	**Absolute number of participants**<br>Significantly different between groups *P*<.05	64<br>(*IG* 33, *CG* 31)
Li et al. 2020 [9]	Repeated Video Display (*Group A*)<br>Video Display & Live Demonstration (*Group B*)	None	**Median of number of participants**<br>***Group A*** 3.00 (Range 2-4)<br>***Group B*** *2* 4.00 (Range 3-5)<br>*P*<.001	48<br>(*A* 24, *B* 24)
Suppan et al. 2020a [4]	Gamified e-learning module & COVID-19 Guideline	COVID-19 guidelines only	**Differences between pre- & posttest within groups**<br>***IG*** *P*=.27<br>***CG*** *P*=.04	173<br>(*IG* 88, *CG* 85)
Suppan et al. 2020b [5]	Gamified e-learning module & COVID-19 Guideline	COVID-19 guidelines only	**Differences between pre- & posttest within groups**<br>***IG*** *P*=.666<br>***CG*** *P*=.521	90<br>(*IG* 41, *CG* 49)
**Perceived confidence in the ability of PPE Donning (Follow up 1)**				**142 (1 Study)**
Rueda-Medina et al. 2022 [10]	Face-to-Face Teaching with Active Training	Non-Face-to-Face Teaching with Passive Training	**Mean Score (SD)**<br>***IG*** 4.85 (0.39), ***CG*** 4.63 (0.73)<br>*P*=.029	142<br>(*IG* 72, *CG* 70)
**Perceived confidence in the ability of PPE Doffing (Follow up 1)**				**142 (1 Study)**
Rueda-Medina et al. 2022 [10]	Face-to-Face Teaching with Active Training	Non-Face-to-Face Teaching with Passive Training	**Mean Score (SD)**<br>***IG*** 4.83 (0.37), ***CG*** 4.64 (0.68)<br>*P*=.042	142<br>(*IG* 72, *CG* 70)
**Confidence thinking regarding infection prevention control** *Composite outcome*				**208 (1 Study)**
Jafree et al. 2022 [14]	Booklet & Whats App based intervention	Only booklet	**Adjusted mean difference**<br>1.17 (95% CI −0.17 to 2.11)<br>*P*=.021<br>**Mean Score (SD)**<br>***IG*** 34.36 (2.68), ***CG*** 33.25 (3.14)	208<br>(*IG* 106, *CG* 102)
**Secondary outcomes related to attitudes towards COVID-19 infection prevention control measures**				
**COVID-19 related attitudes towards providing services in women during pregnancy, childbirth, and breastfeeding (Follow Up 1)**				**90 (1 Study)**
Amiri et al. 2023 [8]	Educational programs for healthcare providers regarding COVID-19 management (instructions and protocols sent by the	Educational content in a routine format	**Mean Score (SD)**<br>***IG1*** 17.5 (4.78),<br>***IG2*** 16.23 (2.86),<br>***CG*** 11.3 (3.68)<br>*P*<.001	90<br>(*IG1* 30, *IG2* 30, *CG* 30)

Interventions for enhancing COVID-19 related health literacy in health professionals				
**Population: Health professionals of various professions**<br>**Setting: Various health care settings**<br>**Intervention: Various interventions**<br>**Comparison: Various comparator**				
**Study (Year)**	**Intervention**	**Comparator**	**Relative effect**	**Participants[a] (Studies)**
	Ministry of Health to universities)<br>**Intervention 1**: via multimedia (audio, video, written content, animation)<br>**Intervention 2**: via booklet (PDF format)			
**COVID-19 related attitudes towards providing services in women during pregnancy, childbirth, and breastfeeding (Follow Up 2)**				**90 (1 Study)**
Amiri et al. 2023 [8]	Educational programs for healthcare providers regarding COVID-19 management (instructions and protocols sent by the Ministry of Health to universities)<br>**Intervention 1**: via multimedia (audio, video, written content, animation)<br>**Intervention 2**: via booklet (PDF format)	Educational content in a routine format	**Mean Score (SD)**<br>***IG1*** 18.17 (5.3),<br>***IG2*** 16.9 (3.36),<br>***CG*** 12.1 (3.9)<br>*P*<.001	90<br>(*IG1* 30, *IG2* 30, *CG* 30)
**Attitudes towards COVID-19 infection prevention behaviors (Follow Up 1)**				**164 (1 Study)**
Rakhshani et al. 2024 [15]	Self-learned virtual learning package including various educational material	Delayed educational program after intervention	**Mean (SD)**<br>***IG*** 15.6 (1.22)<br>***CG*** 10.10 (1.17)<br>*P*=.001	164<br>(*IG* 82, *CG* 82)
Other secondary outcomes				
**Prevalence of infection prevention behaviour (Follow Up 1)**				**250 (1 Study)**
Jeihooni et al. 2023 [1]	Health belief model-oriented training package	Delayed intervention (six month later)	**Mean Score (SD)**<br>***IG*** 10.10 (2.74), ***CG*** 6.01 (2.96)<br>*P*=.001	250<br>(*IG* 125, *CG* 125)
**Perceived COVID-19 related self-efficacy (Follow up 1)**				**250 (1 Study)**
Jeihooni et al. 2023 [1]	Health belief model-oriented training package	Delayed intervention (six month later)	**Mean Score (SD)**<br>***IG*** 30.01 (3.42), ***CG*** 13.01 (3.75)<br>*P*=.001	250<br>(*IG* 125, *CG* 125)

IG Intervention Group, **CG** Control Group, [a] Numbers of participants contributing to the analysis of each outcome, [b] Number of participants varied between Webinars

Table S3. Summary of Findings in Non-randomised Studies of Interventions – Primary Outcomes

Interventions for enhancing COVID-19 related health literacy in health professionals

Population: Health professionals of various professions
Setting: Various health care settings
Intervention: Various interventions
Comparison: Various Comparators

Study (Year)	Intervention	Comparator	Relative effect	Participants[a] (Studies)
**Primary Outcomes aimed at enhancing COVID-19 related knowledge**				
**General COVID-19 related knowledge (Follow Up 1)**				**126 (1 Study)**
**Hu et al. 2021** [16]	Game-based intervention (Group 1)<br><br>Online lecture intervention (Group 2)	None	**Mean difference (SE)**<br>*Pretest vs. Posttest 1*<br>***IG 1*** -3.192 (0.165)<br>95% CI -4.316 to -3.508<br>*P*=.000<br>***IG 2*** -4.276 (1.161)<br>95% CI -4.673 to -3.879<br>*P*=.000	126<br>(*IG 1* 68, *IG 2* 58)
**General COVID-19 related knowledge (Follow Up 2)**				**126 (1 Study)**
**Hu et al. 2021** [16]	Game-based intervention (Group 1)<br><br>Online lecture intervention (Group 2)	None	**Mean difference (SE)**<br>*Posttest 1 vs. Posttest 2*<br>***IG*** 1.559 (0.195)<br>95% CI 1.179 to 1.938<br>*P*=.000<br>***CG*** 2.241 (0.176)<br>95% CI 1.807 to 2.676<br>*P*=.000<br><br>*Pretest vs. Posttest 2*<br>***IG*** -2.353 (0.195)<br>95% CI -2.832 to -1.874<br>*P*=.000<br>***CG*** -2.034 (0.189)<br>95% CI -2.500 to -1.569<br>*P*=.000	126<br>(*IG* 68, *CG* 58)
**COVID-19 specific infection prevention knowledge on PPE performance**				**50 (1 Study)**
**Yu et al. 2022** [17]	Participation in the virtual reality infection control simulation (VRICS) program	No participation in the VRICS program	**Mean Difference (SD)**<br>***IG*** 2.08 (1.75)<br>***CG*** 0.32 (2.08)<br><br>*t*=-3.28, *P*<.001	50<br>(*IG* 25, *CG* 25)

Interventions for enhancing COVID-19 related health literacy in health professionals				
**Population: Health professionals of various professions**<br>**Setting: Various health care settings**<br>**Intervention: Various interventions**<br>**Comparison: Various Comparators**				
**Study (Year)**	**Intervention**	**Comparator**	**Relative effect**	**Participants[a] (Studies)**
**Primary outcomes aimed at enhancing COVID-19 related infection prevention performance skills**				
**Infection control measures performance (Follow Up 1)**				**50 (1 Study)**
**Yu et al. 2022** [17]	Participation in the virtual reality infection control simulation (VRICS) program	No participation in the VRICS program	**Mean difference (SD)**<br>***IG*** 1.41 (1.09)<br>***CG*** 0.20 (0.0.58)<br>t_{48}=4.89, *P*<.001	50<br>(*IG* 25, *CG* 25)
**PPE performance of Donning & Doffing (Follow Up 1)**				**60 (1 Study)**
**Ansari et al. 2023** [18]	Instructor-led training session with demonstrations (Group A)<br><br>Video-based intervention (Group B)	None	**Score**<br>*Donning*<br>***Group A*** Range 3-8<br>***Group B*** range 5-8<br>*P*=.23<br>*Doffing*<br>***Group A*** Range 10-14<br>***Group B*** range 12-14<br>*P*=.16<br>Between group effect: *P*=.36	60<br>(*A* 30, *B* 30)

IG Intervention Group, **CG** Control Group, **PPE** Personal protective equipment

Table S4. Summary of Findings in Non-randomised Studies of Interventions – Secondary Outcomes

Interventions for enhancing COVID-19 related health literacy in health professionals				
**Population: Health professionals of various professions**<br>**Setting: Various health care settings**<br>**Intervention: Various interventions**<br>**Comparison: Various Comparators**				
**Study (Year)**	**Intervention**	**Comparator**	**Relative effect**	**Participants[a] (n Studies)**
**COVID-19 knowledge and skills acquisition (Follow Up 1)** ***Composite outcome***				**52 (2 Studies)**
**Buyego et al. 2022 [19]**	Virtual reality-based simulations	Classroom instruction training	*P* = 4.0E-09	52 (n per group not available)
**Self-efficacy in infection prevention control**				**50 (1 Study)**
**Yu et al. 2022 [17]**	Participation in the virtual reality infection control simulation (VRICS) program	No participation in the VRICS program	**Mean difference (SD)**<br>***IG*** 0.60 (0.47)<br>***CG*** -0.40 (0.90)<br>t_{362}=4.93, *P*<.001	50 (*IG* 25, *CG* 25)

IG Intervention Group, **CG** Control Group

Table S5. Summary of Findings in Uncontrolled Before-After Studies – Primary Outcomes

Interventions for enhancing COVID-19 related health literacy in health professionals			
**Population: Health professionals of various professions**<br>**Setting: Various health care settings**<br>**Intervention: Various interventions**<br>**Comparison: No comparator**			
**Study (Year)**	**Intervention**	**Relative effect**	**No. of Participants[a] (n Studies)**
**Primary Outcomes aimed at enhancing COVID-19 related knowledge**			
**General COVID-19 related knowledge (Follow Up 1)**			**3,510 (18 Studies)**
**Bayomi et al. 2021** [20]	Educational intervention with demonstrations	**Mean (SD)**<br>Pretest: 12.58 (3.20)<br>Posttest: 25.78 (2.42)<br>t=54.37, P=.000	286
**Calik et al. 2022** [21]	Serious game as technology-enhanced simulation	**Mean score difference**<br>r = 0.061, P=.001	62
**Elasrag et al. 2021** [22]	Educational training program	**Difference between pre- & post-test (%)**<br>Pretest: good: 10%, average: 20%, poor: 70%<br>Posttest: good: 76%, average: 18%, poor: 6%	50
**Etebarian et al. 2023** [23]	Educational intervention	**Mean (SD)**<br>Pretest: 7.78 (1.58)<br>Posttest: 10.66 (0.89)<br>η^2=0.789, P<.001	88
**Fuentes et al. 2023** [24]	Educational intervention	**Difference between pre- & post-test (%)**<br>Pretest: Sufficient: 15.1%, Insufficient: 84.9 %<br>Posttest: Sufficient: 92.4 %, Insufficient: 7.6 %	79
**Gupta et al. 2023** [25]	Short video-based educational intervention	**Percentage of correct responses (SD)**<br>Pretest: 63.75 (11.33)<br>Posttest: 86.25 (13.72)<br>**Mean difference (SD)**<br>22.5%, P<.001	80
**Kharel et al. 2022** [26]	Virtual training-of-trainers program (educational intervention)	**Mean (SD)**<br>Pretest: 6.9 (1.4)<br>Posttest: 8.1 (1.4)<br>**Mean difference**<br>1.2 (95% CI 1.06-1.28)<br>P<.01	1198 (Pretest)<br>706 (Posttest)
**Kasapoglu et al. 2023** [27]	Online COVID-19 Patient Care and Emergency Response Interprofessional Training	**Mean (SD)**<br>Pretest: 12.34 (3.33)<br>Posttest: 14.29 (3.29)<br>P<.001	463
**Khari et al. 2022** [28]	E-learning program for COVID-19 patient care	**Mean (SD)**<br>Pretest: 17.21 (0.48)<br>Posttest: 22.95 (0.30)<br>**Mean difference (SD)**<br>5.74 (0.53), $P\leq$.001	100
**McConnell et al. 2024** [29]	Digital serious game	**Mean (SD)**<br>Pretest: 82.64 (13.26)<br>Posttest: 92.68 (13.59)<br>$t_{(209)}$=14.55, P< .001	210
**Mohamed et al. 2023** [30]	E-learning training program on COVID-19	**Mean difference (SD)**<br>10.8 (16.0) Module 1: Introduction to COVID-19	105 (Module 1)[b]
**Nassar et al. 2024** [31]	Training couses based on interactive adult-learning theory	**Median (IQR)**<br>Pretest: 32.0 (30.0; 33.0)<br>Posttest: 35.0 (30.0; 37.0)	181

Interventions for enhancing COVID-19 related health literacy in health professionals			
**Population: Health professionals of various professions**<br>**Setting: Various health care settings**<br>**Intervention: Various interventions**<br>**Comparison: No comparator**			
**Study (Year)**	**Intervention**	**Relative effect**	**No. of Participants[a] (n Studies)**
		**Difference Median (IQR):**<br>3.0 (1.0; 6.0), *P*=.000	
**Odusanya et al. 2022** [32]	Virtual training on COVID-19	**Mean (SD)**<br>Pretest: 28.48 (2.24)<br>Posttest: 28.52 (1.92)<br>t=0.14, *P*=.446	63
**Otu et al. 2021a** [33]	E-health learning intervention with the InStrat COVID-19 tutorial application	**Mean (SD)**<br>Pretest: 53.92 (11.72)<br>Posttest: 73.51 (12.17)<br>t=-37.425, *P*<.001	627
**Otu et al. 2021b** [34]	E-health learning intervention with the InStrat COVID-19 tutorial application	**Percentage of correct responses (SD)**<br>Pretest: 47.5 (9.4)<br>Posttest: 73.1 (10)<br>95% CI 27.9 to -23.3, *P*<.001	123
**Perera et al. 2022** [35]	Training sessions based on multi-media videos and role-playing	**Mean score (%)**<br>Pretest: 77.14%<br>Posttest: 84.06%<br>*P*=.001	256
**Puga et al. 2023** [36]	Educational intervention	**Difference between pre-test and posttest**<br>Pretest: Sufficient: 5 %, Insufficient: 95 %<br>Posttest: Sufficient: 90 %, Insufficient: 10 %<br>χ^2 = 28.97, *P*<.001	18
**Wu et al. 2023** [37]	Community Health Navigator Training on COVID-19	**Mean (SD)**<br>Pretest: 17.3 (0.82)<br>Posttest: 18.0 (1.41)<br>t_5=-0.93, SE=0.72, *P*=0.39<br>Cohen's d = 0.38	18 (Pretest)<br>13 (Posttest)
**General COVID-19 related knowledge (Follow Up 2)**			**173 (2 Studies)**
**Etebarian et al. 2023** [23]	Educational intervention	**Mean (SD)**<br>Posttest: 10.66 (0.89)<br>2-month follow-up: 11.56 (0.57)<br>η^2=0.798, *P*<.001	73
**Khari et al. 2022** [28]	E-learning program for COVID-19 patient care	**Mean (SD)**<br>Posttest 1: 22.95 (0.30)<br>1-month follow-up: 22.79 (0.30)<br>**Mean difference (SD)**<br>Pretest vs. 1 month follow-up: 5.58 (0.53), *P*≤.001<br>Posttest 1 vs. 1 month follow-up: -0.16 (0.52)	100
**COVID-19 specific vaccine knowledge (Follow Up 1)**			**6,822 (6 Studies)**
**Aqel et al. 2023** [38]	Live continuing education session	**Percentage of correct responses**<br>Pretest: 47.3%<br>Posttest: 79.6%<br>*P*<.0001	42
**Bechini et al. 2023** [39]	Optional elective Teaching Activity	**Mean (SD)**<br>Pretest: 26 (4.0)<br>Posttest: 30.5 (2.6)<br>**Difference (%)**<br>+17.05%<br>*P*<.001	387

Interventions for enhancing COVID-19 related health literacy in health professionals			
**Population: Health professionals of various professions**<br>**Setting: Various health care settings**<br>**Intervention: Various interventions**<br>**Comparison: No comparator**			
**Study (Year)**	**Intervention**	**Relative effect**	**No. of Participants[a] (n Studies)**
**Blake et al. 2022** [40]	Interactive, multimedia COVID-19 Vaccine Education training	**Percentage of participants rating knowledge ≥ 8/10**<br>Pretest: 35.5%<br>Posttest: 84.6%	162
**Boccalini et al. 2022** [41]	Elective Teaching Activity on vaccinations	**Mean (SD)**<br>Pretest: 21.2 (4.6)<br>Posttest: 29.2 (3.4)<br>**Difference (%)**<br>+27.3%<br>*P*<.001	449
**Fadel et al. 2025** [42]	Virtual educational session on knowledge and attitudes regarding COVID-19 vaccination	**Mean (SD)**<br>Pretest: 13.82 (5.08)<br>Posttest: 35.59 (2.62)<br>*t*=82.426, *P*=.000	350
**Findyartini et al. 2022** [43]	Massive Open Online Course on COVID-19-related knowledge	**Median (Min-Max)**<br>*Batch 1 (August 2020)*<br>No data available<br>*Batch 2 (November 2020)*<br>Pretest: 10.00 (0.00–10.00)<br>Posttest: 10.00 (0.00–10.00)<br>*P*=.000	3,202 (Batch 1)<br>2,230 (Batch 2)
**Perceived COVID-19 related vaccine knowledge (Follow Up 1)**			**132 (2 Study)**
**Girard et al. 2022** [44]	Information Sessions about COVID-19 Vaccines	**Proportion of participants (%)**<br>Pretest:<br>High: 15.6%<br>Intermediate: 40.6%<br>Low: 40.6%<br>Posttest:<br>High: 47.4%<br>Intermediate: 50.5%<br>Low: 2.1%<br>*P*<.001	97
**Kaufman et al. 2024** [45]	Vaccine Champions programme training	**Difference (%)**<br>*Sore arm*<br>12.9 (95% CI -5.1 to 30.9), *P*=.229<br>*Headache*<br>6.5 (95% CI -12.1 to 25.0), *P*=.688<br>*Mild fever*<br>3.2 (95% CI -16.7 to 23.1), P=1.000<br>*Fatique*<br>9.7 (95% CI -14.2 to 33.6), *P*=0.549	35
**COVID-19 specific knowledge on virus transmission (Follow Up 1)**			**5,712 (5 Studies)**
**Clay et al. 2021** [46]	Educational video intervention	**Percentage of correct responses**<br>*Staff*<br>Pretest: 93%<br>Posttest: 89%<br>*P*>.05<br>*Physician*<br>Pretest: 94%<br>Posttest: 92%<br>*P*>.05	76

Interventions for enhancing COVID-19 related health literacy in health professionals			
**Population: Health professionals of various professions**<br>**Setting: Various health care settings**<br>**Intervention: Various interventions**<br>**Comparison: No comparator**			
**Study (Year)**	**Intervention**	**Relative effect**	**No. of Participants[a] (n Studies)**
**Findyartini et al. 2022** [43]	Massive Open Online Course on COVID-19-related knowledge	**Median (Min-Max)**<br>*Batch 1 (August 2020)*<br>Pretest: 10.00 (0.00–10.00)<br>Posttest: 10.00 (6.00–10.00)<br>*P*=.000<br>*Batch 2 (November 2020)*<br>Pretest: 10.00 (0.00–10.00)<br>Posttest: 10.00 (7.00–10.00)<br>*P*=.000	3,202 (Batch 1)<br>2,230 (Batch 2)
**Fuentes et al. 2023** [24]	Educational intervention	**Difference between pre- & post-test (%)**<br>Pretest: Sufficient: 26.5 %, Insufficient: 73.5 %<br>Posttest:Sufficient: 98.7 %, Insufficient: 1.3 %	79
**Greaves et al. 2023** [47]	Simulation-based training	**Percentage of correct responses**<br>Pretest: 67%<br>Posttest: 81%<br>**Difference (%)**<br>15% (95% CI -3% to 33%, *P*=.109	48
**Wang et al. 2022** [48]	Virtual COVID-19 training and field deployment	**Percentage and differences of correct responses, % (n participants)[b]**<br>*Questionnaire Item No. 2*<br>Pretest: 84% (43)<br>Posttest: 93% (70)<br>**Difference: 9%**<br>*Questionnaire Item No. 3*<br>Pretest: 90% (48)<br>Posttest: 90% (73)<br>**Difference: 0%**	77[a]
**COVID-19 specific knowledge of clinical presentation of SARS-CoV-2 (Follow Up 1)**			**828 (3 Studies)**
**Hwang et al. 2021** [49]	**Intervention 1:** Pre-training (educational lectures)<br>**Intervention 2:** Disaster nursing simulation training program	**Mean (SD)**<br>Pretest: 8.51 (0.70)<br>Midtest (after 1st intervention): 8.41(1.26)<br>**Mean difference**<br>Midtest to Pretest: -0.10<br>*F*= 0.33, *P*=.719	78
**Shahrin et al. 2022** [50]	Modules on IPC and case management modules	**Mean (%)**<br>Pretest: 52.24%<br>Posttest: 62.04%, *P*<.001	357
**Zafar et al. 2022** [51]	Written material (circulated through social media)	**Percentage of correct responses**<br>Pretest: 98.8%<br>Posttest: 100%	400 (Pretest)<br>393 (Posttest)
**COVID-19 specific knowledge of clinical presentation of SARS-CoV-2 (Follow Up 2)**			**78 (1Studies)**
**Hwang et al. 2021** [49]	**Intervention 1:** Pre-training (educational lectures)<br>**Intervention 2:** Disaster nursing simulation training program	**Mean (SD)**<br>Pretest (after 2nd intervention): 8.44 (0.75)<br>**Mean difference**<br>Posttest to Midtest: 0.03<br>*F*= 0.33, *P*=.719	78
**COVID-19 specific knowledge of epidemiology (Follow Up 1)**			**6,507 (4 Studies)**
**Findyartini et al. 2022** [43]	Massive Open Online Course on COVID-19-related knowledge	**Median (Min-Max)**<br>*Batch 1 (August 2020)*	3,202 (Batch 1)

Interventions for enhancing COVID-19 related health literacy in health professionals			
**Population: Health professionals of various professions**<br>**Setting: Various health care settings**<br>**Intervention: Various interventions**<br>**Comparison: No comparator**			
**Study (Year)**	**Intervention**	**Relative effect**	**No. of Participants[a] (n Studies)**
		Pretest: 8.57 (0.00–10.00)<br>Posttest: 10.00 (1.79–10.00)<br>*P*=.000<br>*Batch 2 (November 2020)*<br>Pretest: 8.00 (0.00–10.00)<br>Posttest: 10.00 (3.00–10.00)<br>*P*=.000	2,230 (Batch 2)
**Shahrin et al. 2022 [50]**	Modules on IPC and case management modules	**Difference between pre- & post-test score, Mean (%)**<br>Pretest: 72.36%<br>Posttest: 82.17%<br>*P*<.001	357
**Thakre et al. 2020a [52]**	COVID-19 training program with lectures	**Mean (SD)**<br>Pretest: 3.37 (1.62)<br>Posttest: 5.53 (1.49)<br>*P*<.001	232
**Thakre et al. 2020b [53]**	COVID-19 training program with lectures and demonstrations	**Mean (SD)**<br>Pretest: 5.97 (1.78)<br>Posttest: 7.11 (1.57)<br>*P*=.000	486
**COVID-19 specific knowledge of microbiology (Follow Up 1)**			**718 (2 Studies)**
**Thakre et al. 2020a [52]**	COVID-19 training program with lectures	**Mean (SD)**<br>Pretest: 0.46 (0.49)<br>Posttest: 0.75 (0.43)<br>*P*<.001	232
**Thakre et al. 2020b [53]**	COVID-19 training program with lectures and demonstrations	**Mean (SD)**<br>Pretest: 2.00 (0.71)<br>Posttest: 2.21 (0.96)<br>*P*=.000	486
**COVID-19 specific knowledge on biorisk management (Follow Up 1)**			**36 (1 Study)**
**Qasmi et al. 2021 [54]**	Educational intervention	**Mean (SD)**<br>Pretest: 18.69 (0.560)<br>Posttest: 24.00 (0.676)<br>**Mean difference (SD)**<br>-5.306 (2.896), 95% CI -6.286 to -4.326<br>t_{35}=-10.990, *P*=.000	36
**COVID-19 specific knowledge on infection management (Follow Up 1)**			**5,834 (3 Studies)**
**Findyartini et al. 2021 [43]**	Massive Open Online Course on COVID-19-related knowledge	**Median (Min-Max)**<br>*Batch 1 (August 2020)*<br>Pretest: 9.00 (2.00–10.00)<br>Posttest: 10.00 (0.00–10.00), *P*=.000<br>*Batch 2 (November 2020)*<br>Pretest: 8.00 (0.00–10.00)<br>Posttest: 10.00 (3.00–10.00), *P*=.000	3,202 (Batch 1)<br>2,230 (Batch 2)
**Mohamed et al. 2023 [30]**	E-learning training program on COVID-19	**Mean difference (SD)**<br>6.9 (20.6) Module 4: Infection Control Management	45 (Module 4)[b]
**Shahrin et al. 2022 [50]**	Modules on IPC and case management modules	**Mean difference score (%)**<br>Pretest: 56.44%<br>Posttest: 67.65%<br>*P*<.001	357

Interventions for enhancing COVID-19 related health literacy in health professionals			
**Population: Health professionals of various professions**<br>**Setting: Various health care settings**<br>**Intervention: Various interventions**<br>**Comparison: No comparator**			
**Study (Year)**	**Intervention**	**Relative effect**	**No. of Participants[a] (n Studies)**
**COVID-19 related knowledge on pandemic WHO-protocols (Follow Up 1)**			**60 (1 Study)**
**Aujee et al. 2022** [55]	Educational Intervention	**Mean (SD)**<br>Pretest: 6.83 (1.56)<br>Posttest: 9.82 (0.39)<br>t_{59}=14.08, *P*=.000	60
**COVID-19 specific knowledge on infection prevention control measures (Follow Up 1)**			**15,166 (18 Studies)**
**Ahmed et al. 2022** [56]	Educational program	**Mean (SD)**<br>Pretest: 13.89 (3.1)<br>Posttest: 18.45 (0.890)<br>*P*=.001	159
**Findyartini et al. 2021** [43]	Massive Open Online Course on COVID-19-related knowledge	**Median (Min-Max)**<br>*Batch 1 (August 2020)*<br>Pretest: 8.00 (0.00–10.00)<br>Posttest: 10.00 (2.00–10.00)<br>*P*=.000<br>*Batch 2 (November 2020)*<br>Pretest: 8.00 (0.00–10.00)<br>Posttest: 10.00 (2.00–10.00)<br>*P*=.000	3,202 (Batch 1)<br>2,230 (Batch 2)
**Fuentes et al. 2023** [24]	Educational intervention	**Difference between pre- and post-test (%)**<br>Pretest: Sufficient: 34.1 %, Insufficient: 65.9 %<br>Posttest: Sufficient: 96.2 %, Insufficient: 3.8 %	79
**Halemani et al. 2020** [57]	Training program	**Mean (SD)**<br>Pretest: 11.90 (2.16)<br>Posttest 1: 15.82 (1.39)<br>**Mean difference**<br>3.92<br>*P*=.00	40
**Han et al. 2022** [58]	"Four-Stage" Training and Assessment Tool	**Correct Rate (%)**<br>Stage 1 (Pretest): 86.58%<br>Stage 4 (Posttest): 90.35%<br>χ^2 = 313.339, *P*<.001	5325 (Stage 1)<br>5577 (Stage 4)
**Li et al. 2020** [59]	Online training curriculum program	**Mean (SD)**<br>Pretest: 72.69 (4.38)<br>Posttest: (89.54 (4.77)<br>*P*<.001	35
**Kobayashi et al. 2023** [60]	Online training video on COVID-19 IPC	**Correct response rate**<br>**Mean (SD)**<br>Pretest: 57.0 (13.9)<br>Posttest: 88.3 (12.9)<br>**Mean difference (SD)**<br>+31.1 (15.7), *P*<.01	224 (Pretest)<br>190 (Posttest)
**Macht et al. 2022** [61]	Video conference-based sessions	**Mean (SD)**<br>Pretest: 2.58 (1.31)<br>Posttest: 3.50 (2.56)<br>t_{20}=-1.171, *P*=.255	12 (Pretest)<br>14 (Posttest)
**Mohamed et al. 2023** [30]	E-learning training program on COVID-19	**Mean difference (SD)**<br>5.0 (13.4) Module 2: Infection Control Basics<br>8.8 (11.4) Module 3: Principles of Outbreak Control	40 (Module 2)<br>59 (Module 3)[b]

Interventions for enhancing COVID-19 related health literacy in health professionals			
**Population: Health professionals of various professions**<br>**Setting: Various health care settings**<br>**Intervention: Various interventions**<br>**Comparison: No comparator**			
**Study (Year)**	**Intervention**	**Relative effect**	**No. of Participants[a] (n Studies)**
**Saati et al. 2022** [62]	Training course based on the WHO guide for infection control	**Mean (SD)**<br>Pretest: 7.79 (2.10)<br>Posttest: 11.06 (1.27)<br>p = 0.001 (paired t-test)	177 (Pretest)<br>176 (Posttest)
**Sangwan et al. 2022** [63]	Training sessions with live demonstrations	**Mean Score**<br>*Doctors*<br>Pretest: 5.7<br>Posttest: 11<br>*P*<.05<br>*Nursing staff*<br>Pretest: 4.2<br>Posttest: 10.7<br>*P*<.05	560
**Shahrin et al. 2022** [50]	Modules on IPC and case management modules	**Mean (%)**<br>Pretest: 53.61%<br>Posttest: 59.98%<br>*P*<.001	357
**Sharma et al. 2021** [64]	Virtual live & video-assisted IPC group training	**Level of knowledge (%)**<br>Pretest: Good (>70%): 38.8%, Poor (≤ 70%): 61.2%<br>Posttest: Good (>70%): 66.1%, Poor (≤ 70%): 33.9%<br>**Mean (SD)**<br>Pretest: 12.78 (3.13)<br>Posttest: 14.89 (3.32)<br>*P*<.001<br>**Relative Gain (%)**<br>17.05%	968 (Pretest)<br>888 (Posttest)
**Singh et al. 2021** [65]	Video-assissted IPC training modules	**Mean (SD)**<br>Pretest: 9.8 (2.62)<br>Posttest: 12.8 (2.9)<br>*P*=.001	407
**Thakre et al. 2020a** [52]	COVID-19 training program with lectures	**Mean (SD)**<br>Pretest: 2.91 (1.59)<br>Posttest: 7.26 (1.96)<br>*P*<.001	232
**Thakre et al. 2020b** [53]	COVID-19 training program with lectures and demonstrations	**Mean (SD)**<br>Pretest: 4.07 (1.87)<br>Posttest: 5.73 (1.66)<br>*P*=.000	486
**Wang et al. 2022** [48]	Virtual COVID-19 training and field deployment	**Percentage of and difference in correct responses, % (n participants)[b]**<br>*Questionnaire Item No. 4*<br>Pretest: 65% (48)<br>Posttest: 88% (73)<br>Difference: 23%<br>*Questionnaire Item No. 5*<br>Pretest: 17% (46)<br>Posttest: 46% (72)<br>Difference: 29%<br>*Questionnaire Item No. 6*<br>Pretest: 68% (47)<br>Posttest: 73% (73)<br>Difference: 5%	77[a]

Interventions for enhancing COVID-19 related health literacy in health professionals			
**Population: Health professionals of various professions**<br>**Setting: Various health care settings**<br>**Intervention: Various interventions**<br>**Comparison: No comparator**			
**Study (Year)**	**Intervention**	**Relative effect**	**No. of Participants[a] (n Studies)**
		Questionnaire Item No. 11<br>Pretest: 48% (46)<br>Posttest: 66% (73)<br>Difference: 18%	
**Zafar et al. 2020** [51]	Written material (circulated through social media)	**Percentage of correct responses (%)**<br>*Usage of sanitizer/ hand washing*<br>Pretest: 97.5%<br>Posttest: 100%<br><br>*Diagnosting testing*<br>Pretest: 76%<br>Posttest: 98%	400 (Pretest)<br>393 (Posttest)
**COVID-19 specific knowledge on infection prevention control measures (Follow Up 2)**			**171 (2 Studies)**
**Halemani et al. 2020** [57]	Training program	**Mean (SD)**<br>Pretest: 11.90 (2.16)<br>Posttest 1: 15.82 (1.39)<br>Posttest 2: 16.92 (1.24)<br>**Mean difference**<br>Pretest vs. Posttest 2: 5.02<br>Posttest 1 vs. Posttest 2: 1.1<br>*P*=.00	40
**Kobayashi et al. 2023** [60]	Online training video on COVID-19 IPC	**Correct response rate**<br>**Mean (SD)**<br>Pretest: 57.0 (13.9)<br>Posttest: 88.3 (12.9)<br>3-month follow-up: 72.5 (14.3)<br>**Mean difference (SD)**<br>Pretest vs. 3-month follow-up: +14.9 (16.8), *P*<.01<br>Posttest vs. 3-month follow-up: -16.1 (16.7), *P*<.01	224 (Pretest)<br>190 (Postest)<br>131 (3-month follow-up)
**Perceived COVID-19 specific knowledge on infection prevention control measures (Follow Up 1)**			**809 (1 Study)**
**Espinoza-Castro et al. 2025** [66]	Online Massive Open Online Course	**Median score**<br>*Manual workers*<br>Pretest: 3.41<br>Posttest: 4.08<br>*Intellectual workers*<br>Pretest: 3.57<br>Posttest: 4.14	809
**Perceived COVID-19 specific knowledge on infection management (Follow Up 1)**			**705 (1 Study)**
**Brito-Brito et al. 2021** [67]	Online training program	**Mean score (%)**<br>Pretest: 40% (95% CI 29%-53%)<br>Posttest: 53% (95% CI 39%-60%)<br>*P*<.001	705
**COVID-19 specific knowledge on performing nasopharyngeal swab (Follow Up 1)**			**46 (1 Study)**
**Mark et al. 2020** [68]	Simulation-based training with lectures	**Mean (SD)**<br>Pretest: 3.13 (4.54)<br>Posttest: 1.09 (0.59)<br>**Mean change**<br>1.41 (95% CI 1.10-1.73)<br>*P*< .0001	46

Interventions for enhancing COVID-19 related health literacy in health professionals			
**Population: Health professionals of various professions**<br>**Setting: Various health care settings**<br>**Intervention: Various interventions**<br>**Comparison: No comparator**			
**Study (Year)**	**Intervention**	**Relative effect**	**No. of Participants[a] (n Studies)**
**COVID-19 specific infection prevention knowledge on personal protective equipment (PPE) (Follow Up 1)**			**506 (3 Studies)**
**Bakhsh et al. 2023 [69]**	COVID-19 critical care crash course (virtual and practical)	**Mean (SD)**<br>Pretest: No data available<br>Posttest: 2.80 (0.40)	65
**Greaves et al. 2023 [47]**	Simulation-based training	**Percentage of correct responses**<br>*Donning sequence*<br>Pretest: 81%<br>Posttest: 92%<br>**Difference (%)**<br>10% (95% CI -3% to 24%), *P*=.133<br>*Doffing sequence*<br>Pretest: 52%<br>Posttest: 94%<br>**Difference (%)**<br>42% (95% CI 27%-56%), *P*<.001	48
**Zafar et al. 2020 [51]**	Written material (circulated through social media)	**Percentage of correct responses**<br>*Donning sequence*<br>Pretest: 50.3%<br>Posttest: 92%<br>*Doffing sequence*<br>Pretest: 47.8%<br>Posttest: 65%	400 (Pretest)<br>393 (Posttest)
**COVID-19 specific knowledge on oral health (Follow Up 1)**			**100 (1 Study)**
**Lalitha et al. 2024 [70]**	Intervention 1: Webinar	**Mean (SD)**<br>Pretest: 7.61 (0.15)<br>Posttest: 7.61 (0.163)	80
**Lalitha et al. 2024 [70]**	Intervention 2: Information Education Communication Materials	**Mean (SD)**<br>Pretest: 6.6 (0.28)<br>Posttest: 9.05 (0.25)	20
**COVID-19 related knowledge (not further specified, Follow Up 1)**			**127 (2 Study)**
**Mektirat et al. 2021 [71] (Only abstract)**	Project-based interprofessional education curriculum	**Median, % (IQR)**<br>Pretest: 60% (53.33 - 73.33)<br>Posttest: 80% (70.00 - 86.67)<br>*P*<.01	94
**Roberts et al. 2022b [72]**	Clinical rotations in the tele-ICU	**Median (IQR)**<br>Pre-rotation: 50.0 (11.5–65.7)<br>Post-rotation: 80.0 (58.5–100)<br>*P*=.001	33
**Primary outcomes aimed at enhancing COVID-19 related infection prevention performance skills**			
**General COVID-19 related infection prevention performance skills (Follow Up 1)**			**744 (6Studies)**
**Ahmed et al. 2022 [56]**	Educational program	**Mean (SD)**<br>Pretest: 37.67 (5.37)<br>Posttest: 43.20 (1.98)<br>*P*=.001	159

Interventions for enhancing COVID-19 related health literacy in health professionals			
**Population: Health professionals of various professions**<br>**Setting: Various health care settings**<br>**Intervention: Various interventions**<br>**Comparison: No comparator**			
**Study (Year)**	**Intervention**	**Relative effect**	**No. of Participants[a] (n Studies)**
**Bayomi et al. 2021** [20]	Educational intervention with demonstrations	**Mean (SD)**<br>Pretest: 12.77 (5.66)<br>Posttest: 37.25 (2.50)<br>*t*=65.54, *P*=.000	286
**Elasrag et al. 2021** [22]	Educational training program	**Percentage of correct responses**<br>Pretest: 36%<br>Posttest: 90%<br>*t*=17.05, *P*<.01	50
**Li et al. 2020** [59]	Online training curriculum program	**Mean (SD)**<br>Pretest: 71.86 (7.68)<br>Posttest: 88.31 (4.91)<br>*P*<.001	35
**Saati et al. 2020** [62]	Training course based on the WHO guide for infection control	**Mean (SD)**<br>Pretest: 4.56 (2.58)<br>Posttest: 15.68 (1.90)<br>*P*=.0001	177 (Pretest)<br>176 (Posttest)
**Tan et al. 2020** [73]	Emergency training program of PPE	**Median (IQR)[d]**<br>Pretest: 95.97 (76.01-93.08)<br>Posttest: 96.94 (93.04-99.07)<br>*P*=7.16e–15	38
**PPE performance of Donning & Doffing (Follow Up 1)**			**261 (4 Studies)**
**Diaz-Guio et al. 2020** [74]	Simulation-based educational intervention	**Mean (SD)**<br>Pretest: 2.5 (0.8)<br>Posttest: 7.9 (1.1)	61
**Greaves et al. 2023** [47]	Simulation-based training	**Percentage of correct responses**<br>*Donning score*<br>Pretest: 52%<br>Posttest: 98%<br>**Difference (%)**<br>47% (95%-CI 37%-57%), *P*<.001<br>*Doffing score*<br>Pretest: 46%<br>Posttest: 85%<br>**Difference (%)**<br>39% (95%-CI 33%-46%), *P*<.001	48
**Pokrajac et al. 2020** [75]	Simulation-based mastery learning	**Mean score (%)**<br>Pretest: 73.1% (95% CI 70.9%-75.3%)<br>Posttest: 100%, (95% CI 24.7%-29.1%)[c]<br>*P*<.001	117
**Smith et al. 2023** [76]	Miller's pyramid-based training (Simulation of COVID-19 contamination through flourescent marker)	**Contamination rate, Mean (%)**<br>Pretest: 70%<br>Posttest: 40%<br>*P*<.001	54 (Pretest)<br>35 (Posttest)
**COVID-19 specific infection protection performance skills in laboratory practice (Follow Up 1)**			**80 (1 Study)**
**Gupta et al. 2023** [25]	Short video-based educational intervention	**Percentage of correct responses (SD)**<br>Pretest: 71.8 (10.14)<br>Posttest: 91.38 (10.6)<br>**Mean difference (SD)**<br>19.58%, *P*<.001	80

Interventions for enhancing COVID-19 related health literacy in health professionals			
**Population: Health professionals of various professions**<br>**Setting: Various health care settings**<br>**Intervention: Various interventions**<br>**Comparison: No comparator**			
**Study (Year)**	**Intervention**	**Relative effect**	**No. of Participants[a] (n Studies)**
**Perceived COVID-19 competencies on hand and respiratory hygiene (Follow Up 1)**			**87 (1 Study)**
**Ta'an et al. 2023** [77]	Competency Outcomes and Performance Assessment-based training program	**Mean difference (SD)**<br>0.95 (2.10), 95%-CI 0.51-1.40<br>t_{86}=4.25, *P*<.007	87
**Perceived COVID-19 competencies in the ability of using PPE (Follow Up 1)**			**87 (1 Study)**
**Ta'an et al. 2023** [77]	Competency Outcomes and Performance Assessment-based training program	**Mean difference (SD)**<br>1.32 (1.50), 95%-CI 1.00-1.64<br>t_{86}=8.23, *P*<.007	87
**Perceived competencies in COVID-19 specific waste management (Follow Up 1)**			**87 (1 Study)**
**Ta'an et al. 2023** [77]	Competency Outcomes and Performance Assessment-based training program	**Mean difference (SD)**<br>0.50 (2.02), 95%-CI 0.06-0.93<br>t_{86}=2.28, *P*=.03	87

CI Confidence interval, **IPC** Infection prevention and control, **PPE** Personal protective equipment, **ICU** Intensive Care Unit
[a] Numbers of participants contributing to the analysis of each outcomes; [b] Number of participants varied between single items/modules.
[c] 95% CI were calculated by the review authors based on the reported data; [d] As the reported results in the study are only available as graphed data in a box plot, we used the WebPlotDigitizer software [78] in order to obtain the data for extraction.

Table S6. Summary of Findings in Uncontrolled Before-After Studies – Secondary Outcomes

Interventions for enhancing COVID-19 related health literacy in health professionals			
**Population: Health professionals**<br>**Setting: Various settings**<br>**Intervention: Various interventions**<br>**Comparison: No comparator**			
**Study (Year)**	**Intervention**	**Relative effect**	**No. of Participants[a] (n Studies)**
**Secondary Outcomes aimed at enhancing COVID-19 related knowledge**			
**COVID-19 related knowledge on epidemiology, prevention and microbiology (Follow Up 1)** *Composite outcome*			**232 (1 Study)**
Thakre et al. 2020a [52]	COVID-19 training program with lectures	**Mean Score (SD)**<br>Pretest: 6.75 (3.08)<br>Posttest: 13.55 (3.27)<br>P<.001 (χ^2-Test)	232
**COVID-19 related knowledge on epidemiology, prevention, treatment, microbiology and stages of COVID-19 microbiology (Follow Up 1)** *Composite outcome*			**486 (1 Study)**
Thakre et al. 2020b [53]	COVID-19 training program with lectures and demonstrations	**Mean score (SD)**<br>Pretest: 17.74 (5.00)<br>Posttest: 20.93 (5.03)<br>P=.000	486
**General COVID-19 and public health related knowledge (Follow Up 1)** *Composite outcome*			**27 (1 Study)**
Altillo et al. 2021 [79]	Online learning within medical student elective	**Mean score (%)**<br>Pretest: 43.8%<br>Posttest: 60.8%<br>P<.001	27
**Perceived general COVID-19 and public health related knowledge (Follow Up 1)** *Composite outcome*			**27 (1 Study)**
Altillo et al. 2021 [79]	Online learning within medical student elective	**Mean based on 4-point Likert scale (1 'none' to 4 'high')**<br>Pretest: 2.4<br>Posttest: 3.5<br>P<.0001	27
**COVID-19 specific infection preventions knowledge on PPE and patient airway management (Follow Up 1)** *Composite outcome*			**65 (1 Study)**
Bakhsh et al. 2023 [69]	COVID-19 critical care crash course (virtual and practical)	**Mean (SD)**<br>Pretest: 14.92 (3.20)<br>Posttest: 18.81 (1.40)<br>P<.01	65
**COVID-19 specific knowledge on infection prevention control measures and management (Follow Up 1)** *Composite outcome*			**482 (2 Studies)**
Kufel et al. 2022 [80]	Interactive didactic class with active learning	**Mean (SD)**<br>Pretest: 5.9 (1.31)<br>Posttest: 8.6 (1.29)<br>P<.001	61
Naz et al. 2022 [81]	Health education sessions	**Mean (SD)**<br>*Gender: male vs. female*<br>Pretest: 7.59 (2.991) vs. 8.58 (2.923), P=.00<br>Posttest: 13.93 (3.326) vs. 14.48 (3.521), P=.00	421
**COVID-19 related knowledge and competencies (Follow Up 1)** *Composite outcome*			**2,901 (1 Study)**
Roberts et al. 2022a [82]	Continuing education program	**Mean of correct scores (%)**<br>*Webinar 1 (949 participants)*<br>Pretest: 57%<br>Posttest: 84%<br>Cohen d=1.92, P<.001<br>*Webinar 2 (1,103 participants)*	2,901[b]

Interventions for enhancing COVID-19 related health literacy in health professionals			
**Population: Health professionals**<br>**Setting: Various settings**<br>**Intervention: Various interventions**<br>**Comparison: No comparator**			
**Study (Year)**	**Intervention**	**Relative effect**	**No. of Participants[a] (n Studies)**
		Pretest: 49%<br>Posttest: 85%<br>Cohen *d*=1.85, *P*<.001<br>*Webinar 3 (849 participants)*<br>Pretest: 55%<br>Posttest: 86%<br>Cohen *d*=1.73, *P*<.001	
**COVID-19 practical knowledge & skills related to infection prevention control measures (Follow Up 1)**<br>*Composite outcome*			**2,370 (1 Study)**
Tsiouris et al. 2022 [83]	Emergency training (tailored)	**Mean (SD)**<br>Pretest: 0.59 (0.15)<br>Posttest: 0.73 (0.13)<br>**Mean differences (SD)**<br>0.15 (0.13), 95% CI 0.14-0.13<br>*t*=55.09, *P*<.0001	2,370
**COVID-19 related knowledge on prevention and health promotion (Follow Up 1)** *Composite outcome*			**7, 243 (1 Study)**
Salehi et al. 2023 [84]	E-learning & in-person courses	**Mean score (SD)**<br>Pretest: 74.8 (17.0)<br>Posttest: 85.5 (14.1)<br>Z=-46.9, r=-0.39, *P*<.01	6, 799 (Pretest)<br>7,243 (Posttest)
**COVID-19 disease specific competencies based on knowledge, practice, attitudes (Follow Up 1)**<br>*Composite outcome*			**129,530 (1 Study)**
Rosas-Magallanes et al. 2022 [85]	Massive Online Open Courses	**Mean difference**<br>26.0 (95% CI 25.9-30.1)	129,530
**COVID-19 related knowledge and awareness (Follow Up 1)** *Composite outcome*			**1,052 (1 Study)**
Bohara et al. 2021 [86]	Two-level training curriculum program	Cumulative knowledge index<br>Welch's *t* = –2.16, *P* < .05<br>Cumulative multiple choice index, *P*<.001<br>True/False index, *P*<.01	1,052
**COVID-19 knowledge on symptoms, infection prevention control measures, vaccination and breastfeeding (Follow Up 1)**<br>*Composite outcome*			**210 (1 Study)**
Garcia et al. 2024 [87]	Evidence-based educational sessions about COVID-19 for low-literacy lay midwives	**Mean (SD)**<br>Pretest: 7.09 (3.06)<br>Posttest: 15.2 (4.61)<br>*P*>.001	210
**General and COVID-19-related knowledge on transmission and home care of infected or suspected COVID-19 patients (Follow Up 1)** *Composite outcome*			**243 (1 Study)**
Sabandüzen et al. 2024 [88]	Training on home care of infected or suspected COVID-19 patients	**Mean (SD)**<br>Pretest: 7.97 (1.51)<br>Posttest: 9.11 (1.15)<br>*P*<.001	243
**General and COVID-19-related knowledge on nursing care and healthy life style (Follow Up 1)**<br>*Composite Outcome*			**70 (1 Study)**
Said et al. 2021 [89]	Educational intervention	**Mean (SD)**<br>Pretest: 14.1 (3.25)<br>Posttest: 22.52 (1.09)<br>*P*<.001	70

Interventions for enhancing COVID-19 related health literacy in health professionals			
**Population: Health professionals**<br>**Setting: Various settings**<br>**Intervention: Various interventions**<br>**Comparison: No comparator**			
**Study (Year)**	**Intervention**	**Relative effect**	**No. of Participants[a] (n Studies)**
**COVID-19 knowledge on PPE, clinical assessment, diagnostic and treatment (Follow Up 1)**<br>*Composite outcome*			**15,898 (1 Study)**
Strehlow et al. 2024 [90]	Massive open online course	**Mean score (%)**<br>*Physician*<br>Pretest: 52%<br>Posttest 1: 74%<br>Posttest 2: 78%<br>**Mean difference**<br>*Physicians*<br>Pretest-Posttest 1: 23%, *P*<.001<br>Pretest-Posttest 2: 26%, *P*<.001	10,714 (Pretest)<br>5,184 (Posttest)
**Secondary outcomes aimed at enhancing COVID-19 related performance skills**			
**COVID-19 performance skills on COVID-19 patient care and infection protection (Follow Up 1)**<br>*Composite outcome*			**70 (1 Study)**
Said et al. 2021 [89]	Educational intervention	**Mean (SD)**<br>Pretest: 10.15 (2.26)<br>Posttest: 19.91 (1.53)<br>*P*<.001	70
**Secondary outcomes aimed at enhancing COVID-19 related confidence**			
**Perceived confidence on performing nasopharyngeal swab knowledge (Follow Up 1)**			**46 (1 Study)**
Mark et al. 2020 [68]	Simulation-based training with lectures	**Mean (SD)**<br>Pretest: 3.13 (1.09)<br>Posttest: 4.54 (0.59)<br>**Mean difference**<br>1.41 (95% CI 1.10-1.73)<br>*P*<.0001	46
**Perceived confidence in performing nasopharyngeal swab (Follow Up 1)**			**135 (2 Studies)**
Bieri et al. 2023 [91]	Educational and practical lecture (Student-Teacher–Based Blended Curriculum)	**Mean (SD)**<br>Pretest: 3.59 (1.13)<br>Posttest: 4.32 (0.76)<br>*P*<.001	82 (Pretest)<br>73 (Posttest)
Instrum et al. 2022 [92]	Nasopharyngeal swab trainer simulation	**Percentage of perceived confidence level > 4 on 5-point Likert scale (1 ‘not confident’, 5 ‘extremely confident’)**<br>Pretest: 27%<br>Posttest: 71%	62
**Perceived confidence in identifying populations at risk for severe COVID-19 (Follow Up 1)**			**2,901 (1 Study)**
Roberts et al. 2022a [82]	Continuing education program	**Mean**<br>*Webinar 1 (949 participants)*<br>Pretest: 3.11<br>Posttest: 4.00<br>Cohen *d*=1.24, *P*<.001<br>*Webinar 2 (1,103 participants)*<br>Pretest: 3.24<br>Posttest: 4.15<br>Cohen *d*=1.09, *P*<.001<br>*Webinar 3 (849 participants)*	2,901[b]

Interventions for enhancing COVID-19 related health literacy in health professionals			
**Population: Health professionals**<br>**Setting: Various settings**<br>**Intervention: Various interventions**<br>**Comparison: No comparator**			
**Study (Year)**	**Intervention**	**Relative effect**	**No. of Participants[a] (n Studies)**
		Pretest: 3.50<br>Posttest: 4.22<br>Cohen *d*=0.88, *P*<.001	
**Perceived confidence in COVID-19 critical care (Follow Up 1)**			**65 (1 Study)**
Bakhsh et al. 2023 [69]	COVID-19 critical care crash course (virtual and practical)	**Mean (SD)**<br>Pretest: 4.98 (1.15)<br>Posttest: 8.76 (1.10)<br>*P*<.01	65
**Confidence in educating community members regarding COVID-19 vaccination and prevention (Follow Up 1)**			**13 (1 Study)**
Wu et al. 2023 [37]	Community Health Navigator Training on COVID-19	**Mean (SD)**<br>Pretest: 4.17 (0.73)<br>Posttest: 4.50 (0.54)<br>t_7=1.23, Cohen *d*=0.44, *P*=0.26	18 (Pretest)<br>13 (Posttest)
**Perceived confidence infection prevention and management (Follow Up 1)** *Composite outcome*			**61 (1 Study)**
Kufel et al. 2022 [80]	Interactive didactic class with active learning	**Mean (SD)**<br>Pretest: 2.66 (0.75)<br>Posttest: 4.03 (0.53)<br>*P*<.001	61
**Perceived confidence in the ability to communicate COVID-19 vaccine related importance (Follow Up 1)**			**162 (1 Study)**
Blake et al. 2022 [40]	Interactive, multimedia COVID-19 Vaccine Education training	**Percentage of participants rating confidence ≥ 8/10**<br>Pretest: 44.5%<br>Posttest: 80.2%	162
**Confidence in providing information about COVID-19 vaccine (Follow Up 1)**			**97 (1 Study)**
Girard et al. 2022 [44]	Information Sessions about COVID-19 Vaccines	**Proportion of participants (%)**<br>Pretest:<br>Completely: 9.3%<br>Relatively: 45.4%<br>Not really: 34.0%<br>Not at all: 11.3%<br>Posttest:<br>Completely: 34.0%<br>Relatively: 57.7%<br>Not really: 7.2%<br>Not at all: 1.0%<br>*P*<.001	97
**Secondary outcome related to compliance towards and prevalence of COVID-19 infection prevention control measures behaviour**			
**Observed compliance to infection prevention measures (Follow Up 1)**			**1,132 (Study)**
Clay et al. 2021 [46]	Educational video intervention	**Percentage of observed compliance**<br>**(a) Mask Use**<br>*Staff*<br>Pretest: 84.7%<br>Posttest: 94.2%<br>*P*<.001	667 (Preintervention)<br>1,132 (Postintervention)

Interventions for enhancing COVID-19 related health literacy in health professionals			
**Population: Health professionals**<br>**Setting: Various settings**<br>**Intervention: Various interventions**<br>**Comparison: No comparator**			
**Study (Year)**	**Intervention**	**Relative effect**	**No. of Participants[a] (n Studies)**
		Physician<br>Pretest: 96.1%<br>Posttest: 99%<br>*P*<.001<br>**(b) Social Distancing**<br>*Staff*<br>Pretest: 85.24%<br>Posttest: 93.8%<br>*P*<.001<br>*Physician*<br>Pretest: 96.1%<br>Posttest: 99%<br>*P*<.001	
**Perceived compliance to infection prevention measures (Follow Up 1)**			**47 (1 Study)**
Clay et al. 2021 [46]	Educational video intervention	**Rates based on 5-point Likert scale (1 '0-20%' to 4 '81-100%')**<br>**(a) Mask Use**<br>*Staff (5 participants)*<br>Pretest: 5<br>Posttest: 5<br>*Physicians (26 participants)*<br>Pretest: 4.8<br>Posttest: 4.8<br>**(b) Social Distancing**<br>*Staff (5 participants)*<br>Pretest: 4.6<br>Posttest: 4.0<br>*Physicians (26 participants)*<br>Pretest: 4.5<br>Posttest: 4.7<br>*P*<0.05	37 (Pretest)<br>47 (Posttest)
**Adherence to COVID-19 infection prevention control measures (Follow Up 1)**			**151 (2 Studies)**
Etebarian et al. 2023 [23]	Educational intervention	**Mean (SD)**<br>Pretest: 50.75 (5.79)<br>Posttest: 56.81 (3.19)<br>η^2=0.728, *P*<.001	88
Odusanya et al. 2022 [32]	Virtual training on COVID-19	**Mean (SD)**<br>Pretest: 33.95 (3.13)<br>Posttest: 34.60 (2.73)<br>*P*=.070	63
**Adherence to COVID-19 infection prevention measures (Follow Up 2)**			**73 (1 Study)**
Etebarian et al. 2023 [23]	Educational intervention	**Mean (SD)**<br>Posttest: 56.81 (3.19)<br>2-month follow-up: 59.40 (0.93)<br>η^2=0.728, *P*<.001	73
**Prevalence of infection prevention behaviour (Follow Up 1)**			**582 (2 Studies)**
Abbas et al. 2020 [93]	Web-based health education module	**Proportion of participants (%)**<br>*Daily frequency of hand washing*	401

Study (Year)	Intervention	Relative effect	No. of Participants[a] (n Studies)
**Interventions for enhancing COVID-19 related health literacy in health professionals**			
**Population: Health professionals**<br>**Setting: Various settings**<br>**Intervention: Various interventions**<br>**Comparison: No comparator**			
		< 5 times/day: 74.8% vs. 42.6%<br>> 5 times/day: 25.2% vs. 57.4%<br>*Duration of each hand washing*<br>Less than 20s: 62.6% vs. 28.9%<br>At least for 20s: 37.4% vs. 71.1%<br>*Use of alcohol-based sanitizer:*<br>*Before examining a patient*<br>Yes: 60.2% vs. 74.1%<br>No: 39.8% vs. 25.9%<br>*After examining a patient*<br>Yes: 44.4% vs. 76.6%<br>No:55.6% vs. 23.4%<br>*After contact with blood, body fluids or contaminated surfaces*<br>Yes: 60.2% vs. 71.3%<br>No: 39.8% vs. 28.7%<br>*Wash hands or use sanitizer immediately after glove removal*<br>Yes: 41.0% vs. 61.1%<br>No: 59.0% vs. 38.9%<br>*Wash hands before touching your eyes, nose and mouth*<br>Yes: 29.0% vs. 75.3%<br>No: 71.0% vs. 24.7%<br>*Wash hands after caring for a person with confirmed or suspected COVID-19 infection*<br>Yes: 63.8% vs. 76.1%<br>No: 36.2% vs. 23.9%	
Nassar et al. 2024 [31]	Training couses based on interactive adult-learning theory	**Median (IQR)**<br>Pretest: 44.0 (41.0; 47.0)<br>Posttest: 46.0 (42.0; 49.0)<br>**Difference Median (IQR):**<br>2.0 (-1.0; 5.0), *P*=.000	181
**Secondary outcomes related to attitudes towards COVID-19 infection prevention control measures**			
**Attitudes towards COVID-19 infection prevention control measures (Follow Up 1)**			**740 (6 Studies)**
Ahmed et al. 2022 [56]	Educational program	**Mean (SD)**<br>Pretest: 18.01 (2.63)<br>Posttest: 21.92 (1.64), *P*=.001	159
Bayomi et al. 2021 [20]	Educational intervention with demonstrations	**Mean (SD)**<br>Pretest: 8.84 (3.18)<br>Posttest: 17.40 (1.20)<br>t=40.95, *P*=.000	286
Elasrag et al. 2021 [22]	Educational training program	**Mean (SD)**<br>Pretest: 13.70 (3.45)<br>Posttest: 25.18 (3.66)<br>t=14.503, *P*<.01	50
Etebarian et al. 2023 [23]	Educational intervention	**Mean (SD)**<br>Pretest: 43.13 (3.86)<br>Posttest: 45.28 (2.39)<br>η^2=0.317, *P*<0.05	88

Interventions for enhancing COVID-19 related health literacy in health professionals			
**Population: Health professionals**<br>**Setting: Various settings**<br>**Intervention: Various interventions**<br>**Comparison: No comparator**			
**Study (Year)**	**Intervention**	**Relative effect**	**No. of Participants[a] (n Studies)**
Mektirat et al. 2021 [71] (Only abstract)	Project-based interprofessional education curriculum	**Median (IQR)**<br>Pretest: 4 (3.00-4.25)<br>Posttest: 4 (3.00–4.00)<br>**Average gain score (%)**<br>12.34 %<br>*P*=.37	94
Odusanya et al. 2022 [32]	Virtual training on COVID-19	**Mean (SD)**<br>Pretest: 34.44 (3.72)<br>Posttest: 34.83 (3.84)<br>*t*=0.63, *P*=.267	63
**Attitudes towards COVID-19 infection prevention control measures (Follow Up 2)**			**73 (1 Study)**
Etebarian et al. 2023 [23]	Educational intervention	**Mean (SD)**<br>Posttest: 45.28 (2.39)<br>2-month follow-up: 45.88 (1.31)<br>η^2=0.317, *P*<0.05	73
**Attitudes towards COVID-19 vaccination (Follow Up 1)**			**350 (1 Study)**
Fadel et al. 2025 [42]	Virtual educational session on knowledge and attitudes regarding COVID-19 vaccination	**Mean difference of total negative attitudes scores (%)**<br>*Low negative*<br>Pretest: 5.7, Posttest: 90.3, *P*=.000<br>*Intermediate negative*<br>Pretest: 91.7, Posttest: 9.4, *P*=.000<br>*Very negative*<br>Pretest: 2.6, Posttest: 0.3, *P*=.000<br>**Mean (SD)**<br>Pretest: 36.38 (7.03), Posttest: 17.6 (5.13)<br>*t*=59.220, *P*=.000	350
**Attitudes towards COVID-19 (not further specified, Follow Up 1)**			**261 (2 Study)**
Gupta et al. 2023 [25]	Short video-based educational intervention	**Percentage of correct responses (SD)**<br>Pretest: 79.25 (18.4)<br>Posttest: 86 (17.83)<br>**Mean difference (SD)**<br>6.75%, *P*=.017	80
Nassar et al. 2024 [31]	Training couses based on interactive adult-learning theory	**Median (IQR)**<br>Pretest: 42.0 (39.0; 45.0)<br>Posttest: 43.0 (40.5; 46.0)<br>**Difference Median (IQR):**<br>1.0 (-1.0; 4.0), *P*=.001	181
**Other secondary outcomes**			
**Perceived COVID-19 related self-efficacy, knowledge & confidence (Follow Up 1)**<br>*Composite outcomes*			**50 (1 Study)**
Zhao et al. 2022 [94]	Telementoring education program within the ‘Extension for Community Healthcare Outcomes’ study program	**Median (IQR) based on 7-point Likert scale 7 (1 ‘strongly disagree’ to 7 ‘strongly agree’)**<br>Pretest: 4.7 (4.1–5.5)<br>Posttest: 6.0 (5.5–6.4)<br>**Median difference**<br>1.3 (95% CI 0.8-1.7)<br>*P*<.0001	50

Interventions for enhancing COVID-19 related health literacy in health professionals			
**Population: Health professionals**<br>**Setting: Various settings**<br>**Intervention: Various interventions**<br>**Comparison: No comparator**			
**Study (Year)**	**Intervention**	**Relative effect**	**No. of Participants[a] (n Studies)**
**Perceived confidence & knowledge in performing nasopharyngeal swab (Follow Up 1)** *Composite outcome*			**73 (1 Study)**
Bieri et al.<br>2023 [91]	Educational and practical lecture (Student-Teacher–Based Blended Curriculum)	**Mean (SD)**<br>Pretest: 3.59 (1.13)<br>Posttest: 4.32 (0.76)<br>*P*<.001	82 (Pretest)<br>73 (Posttest)
**Perceived confidence & knowledge in using PPE of Donning & Doffing (Follow Up 1)** *Composite Outcome*			**48 (1 Study)**
Greaves et al.<br>2023 [47]	Simulation-based training	**Percentage of perceived confidence level > 4 on 5-point Likert scale (1 'no knowledge', 5 'extremely confident')**<br>Pretest: 13%, Posttest: 100%<br>**Difference (%)**<br>88% (95% CI 78-97)<br>**Median (IQR)**<br>Pretest: 3 (2-3)<br>Posttest: 5 (4-5)<br>*P*<.001	48

[a] Numbers of participants contributing to the analysis of each outcomes. [b] Number of participants varied between Webinars.

References

1. Jeihooni AK, Namdari A, Kashfi SM, Kamyab A, Harsini PA, Rakhshani T. Effects of an educational intervention based on the health belief model on COVID-19 preventive behaviors among health personnel in Abadan, Iran. Journal of Public Health 2023. doi:10.1007/s10389-023-02053-6
2. Alotaibi N, Al-Sayegh N, Nadar M, Shayea A, Allafi A, Almari M. Investigation of Health Science Students' Knowledge Regarding Healthy Lifestyle Promotion During the Spread of COVID-19 Pandemic: A Randomized Controlled Trial. Frontiers in public health 2021;9:774678. doi:10.3389/fpubh.2021.774678
3. Currat L, Suppan M, Gartner BA, Daniel E, Mayoraz M, Harbarth S, Suppan L, Stuby L. Impact of Face-to-Face Teaching in Addition to Electronic Learning on Personal Protective Equipment Doffing Proficiency in Student Paramedics: Randomized Controlled Trial. International journal of environmental research and public health 2022;19(5). doi:10.3390/ijerph19053077
4. Suppan L, Abbas M, Stuby L, Cottet P, Larribau R, Golay E, Iten A, Harbarth S, Gartner B, Suppan M. Effect of an E-Learning Module on Personal Protective Equipment Proficiency Among Prehospital Personnel: Web-Based Randomized Controlled Trial. Journal of medical Internet research 2020;22(8):e21265. doi:10.2196/21265
5. Suppan L, Stuby L, Gartner B, Larribau R, Iten A, Abbas M, Harbarth S, Suppan M. Impact of an e-learning module on personal protective equipment knowledge in student paramedics: a randomized controlled trial. Antimicrobial Resistance & Infection Control 2020;9(1):185. doi:10.1186/s13756-020-00849-9
6. Xie M, Zhou Q, Kang Y, Qing P, Guo Y, Wei X, Cai B, Zeng J, Huang J. The Skill Training of Resident Anesthesiologists During the Outbreak Of COVID-19. ResearchSquare 2021. doi:10.21203/rs.3.rs-144144/v1
7. Wang XX, Zhou YZ, Song ZX, Wang YT, Chen XT, Zhang DD. Practical COVID-19 Prevention Training for Obstetrics and Gynecology Residents Based on the Conceive-Design-Implement-Operate Framework. Frontiers in public health 2022;10. doi:10.3389/fpubh.2022.808084
8. Amiri, B, Khajavian, N, Rahmani, R, Bilandi RR. Comparing the Impact of Multimedia and Educational Brochures on Knowledge, Attitude and Work Performance of Healthcare about COVID-19 Management in Pregnancy, Childbirth, and Breastfeeding. Iranian Red Crescent Medical Journal 2023;25(11). doi:10.32592/ircmj.2023.25.11.2498
9. Li Y, Wang Y, Li Y, Zhong M, Liu H, Wu C, Gao X, xia Z, Ma W. Comparison of Repeated Video Display vs Combined Video Display and Live Demonstration as Training Methods to Healthcare Providers for Donning and Doffing Personal Protective Equipment: A Randomized Controlled Trial. Risk Management and Healthcare Policy 2020;13:2325-2335. doi:10.2147/RMHP.S267514
10. Rueda-Medina B, Aguilar-Ferrándiz ME, Esteban-Burgos AA, Tapia Haro RM, Casas-Barragán A, Velando-Soriano A, Gil-Gutiérrez R, Correa-Rodríguez M. Impact of Non-Face-to-Face Teaching with Passive Training on Personal Protective Equipment Use in Health Science Students: A Randomized Controlled Trial. International journal of environmental research and public health 2022;19(19). doi:10.3390/ijerph191912981
11. Christensen L, Rasmussen CS, Benfield T, Franc JM. A Randomized Trial of Instructor-Led Training Versus Video Lesson in Training Health Care Providers in Proper Donning and Doffing of Personal Protective Equipment. Disaster Medicine and Public Health Preparedness 2020;14(4):514-520. doi:10.1017/dmp.2020.56
12. Manggala SK, Tantri AR, Sugiarto A, Sianipar IR, Prasetyono TOH. In situ simulation training for a better interprofessional team performance in transferring critically ill patients with COVID-19: a prospective randomised control trial. Postgraduate medical journal 2022. doi:10.1136/postgradmedj-2021-141426
13. Birrenbach T, Zbinden J, Papagiannakis G, Exadaktylos AK, Muller M, Hautz WE, Sauter TC. Effectiveness and Utility of Virtual Reality Simulation as an Educational Tool for Safe Performance of COVID-19 Diagnostics: Prospective, Randomized Pilot Trial. JMIR serious games 2021;9(4):e29586. doi:10.2196/29586
14. Jafree SR, Zakar R, Rafiq N, Javed A, Durrani RR, Burhan SK, Hasnain Nadir SM, Ali F, Shahid A, Momina AU, Wrona KJ, Mahmood QK, Fischer F. WhatsApp-Delivered Intervention for Continued Learning for Nurses in Pakistan During the COVID-19 Pandemic: Results of a Randomized-Controlled Trial. Frontiers in public health 2022;10:739761. doi:10.3389/fpubh.2022.739761
15. Rakhshani T, Dolatkhah SM, Kashfi SM, Khani Jeihooni A. The effect of a self-learned virtual learning package on knowledge, attitude, and self-care behaviors of COVID-19 in people referred to health and treatment centers. BMC Public Health 2024;24(1):1710. doi:10.1186/s12889-024-19233-y
16. Hu H, Xiao YY, Li H. The Effectiveness of a Serious Game Versus Online Lectures for Improving Medical Students' Coronavirus Disease 2019 Knowledge. Games For Health Journal 2021;10(2):139-144. doi:10.1089/g4h.2020.0140
17. Yu M, Yang MR. Effectiveness and Utility of Virtual Reality Infection Control Simulation for Children With COVID-19: Quasi-Experimental Study. JMIR serious games 2022;10(2):e36707. doi:10.2196/36707
18. Ansari A., Urooj U., Waseem M., Ihtasham A. Video based learning vs instructor led training for optimising personal protective equipment use to prevent Covid-19 infection-a comparative study. J Pak Med Assoc 2022;72(5):807-810. doi:10.47391/JPMA.3359
19. Buyego P, Katwesigye E, Kebirungi G, Nsubuga M, Nakyejwe S, Cruz P, McCarthy MC, Hurt D, Kambugu A, Arinaitwe JW, Ssekabira U, Jjingo D. Feasibility of virtual reality based training for optimising COVID-19 case handling in Uganda. BMC Medical Education 2022;22(1):274. doi:10.1186/s12909-022-03294-x
20. Bayomi R, taha N. Effect of Teaching Guidelines on Knowledge, Attitudes, and Practices Regarding COVID19 among the First Year Nursing Students. Assiut Scientific Nursing Journal 2021;9(25):38-47. doi:10.21608/asnj.2021.72355.1156
21. Calik A, Cakmak B, Kapucu S, Inkaya B. The effectiveness of serious games designed for infection prevention and promotion of safe behaviors of senior nursing students during the COVID-19 pandemic. American journal of infection control 2022. doi:10.1016/j.ajic.2022.02.025
22. Elasrag GAEA, Elsabagh NE, Abdelmonem AF, Ahmed A. Impact of Educational Intervention on Nurses' Knowledge, Practice and Attitude Related Prevention Measures of COVID 19. IJFMT 2021. doi:10.37506/ijfmt.v15i3.15751
23. Etebarian A, Tusi SK, Momeni Z, Hejazi K. Impact of educational intervention regarding COVID-19 on knowledge, attitude, and practice of students before dental school re-opening. BMC Oral Health 2023;23(1):1-6. doi:10.1186/s12903-023-02845-y
24. Fuentes GM, Carbajales León EB, Carbajales León AI. Educative intervention about COVID-19 in the Medicine students from Joaquín de Agüero Policlinic. Revista Electrónica Medimay 2020;27(3):366-376.
25. Gupta S, Goswami B, Madhu SV. Effectiveness of Video-Based Educational Intervention on Knowledge, Attitude, and Practice (KAP) of COVID-19 Health Care Workers: Lesson for Future Pandemic Preparedness. ANAMS 2023;59:233-236. doi:10.1055/s-0043-1772218

26. Kharel R, Baird J, Vaishnav H, Chillara N, Lee JA, Genisca A, Hayward A, Uzevski V, Elbenni A, Levine AC, Aluisio AR. Development and assessment of novel virtual COVID-19 trainer-of trainers course implemented by an academic-humanitarian partnership. Global health action 2022;15(1):2010391. doi:10.1080/16549716.2021.2010391
27. Kasapoglu ES, Yildiz YS, Saldamli A, Karaçetin F. The effect of COVID-19 patient care and emergency response interprofessional training on COVID-19 knowledge, perception, behavior and readiness for care. WORK-A JOURNAL OF PREVENTION ASSESSMENT & REHABILITATION 2023;75(3):767-778. doi:10.3233/WOR-220227
28. Khari S, Pazokian M, Abadi AS, Zarmehrparirouy M, Ahmadvand Y. The Effect of E-Learning Program for COVID-19 Patient Care on the Knowledge of Nursing Students: A Quasi-Experimental Study. SAGE Open Nursing 2022;8. doi:10.1177/23779608221124421
29. McConnell H, Duncan D, Stark P, Anderson T, McMahon J, Creighton L, Craig S, Carter G, Smart A, Alanazi A, Mitchell G. Enhancing COVID-19 Knowledge among Nursing Students: A Quantitative Study of a Digital Serious Game Intervention. Healthcare (Basel) 2024;12(11). doi:10.3390/healthcare12111066
30. Mohamed Y, Hezeri P, Kama H, Mills K, Walker S, Hau'ofa N, Amol C, Jones M, du Cros P, Lin YD. Evaluation of an Online Training Program on COVID-19 for Health Workers in Papua New Guinea. Tropical medicine and infectious disease 2023;8(6). doi:10.3390/tropicalmed8060327
31. Nassar AAH, Al Serouri AA, Al-Shahethi AH, Almoayed KA. Effectiveness of training on health care workers' knowledge, attitude and practice regarding COVID-19 infection prevention and control, Yemen, 2021. BMC Health Services Research 2024;24(1):1411. doi:10.1186/s12913-024-11927-8
32. Odusanya OO, Adeniran A, Bakare OQ, Odugbemi BA, Enikuomehin OA, Jeje OO, Emechebe AC. Building capacity of primary health care workers and clients on COVID-19: Results from a web-based training. PLOS ONE 2022;17(10):e0274750. doi:10.1371/journal.pone.0274750
33. Otu A, Okuzu O, Effa E, Ebenso B, Ameh S, Nihalani N, Onwusaka O, Tawose T, Olayinka A, Walley J. Training health workers at scale in Nigeria to fight COVID-19 using the InStrat COVID-19 tutorial app: an e-health interventional study. Therapeutic advances in infectious disease 2021;8:20499361211040704. doi:10.1177/20499361211040704
34. Otu A, Okuzu O, Ebenso B, Effa E, Nihalani N, Olayinka A, Yaya S. Introduction of Mobile Health Tools to Support COVID-19 Training and Surveillance in Ogun State Nigeria. Front. Sustain. Cities 2021;3. doi:10.3389/frsc.2021.638278
35. Perera N, Haldane V, Ratnapalan S, Samaraweera S, Karunathilake M, Gunarathna C, Bandara P, Kawirathne P, Wei XL. Implementation of a coronavirus disease 2019 infection prevention and control training program in a low-middle income country. JBI Evidence Implementation 2022;20(3):228-235. doi:10.1097/XEB.0000000000000307
36. Puga RR, Cardoso AL, Rodríguez OL. Educational intervention in medical students on post-COVID-19 disabling sequelae from the Public Health subject. Revista Cubana De Reumatologia 2023;25(3).
37. Wu TY, Hoffman JL, Chow CM, Hartl B. Training community health navigators in the public health workforce to respond during the COVID-19 pandemic. Z Gesundh Wiss 2023:1-8. doi:10.1007/s10389-022-01812-1
38. Aqel O, Alqadheeb B, Felix M, Amundson C, Bingham JM, Meyer K, Warholak T, Axon DR. Cultivating COVID-19 Vaccine Confidence in Pharmacy Professionals. Pharmacy 2023;11(2). doi:10.3390/pharmacy11020050
39. Bechini A, Vannacci A, Salvati C, Crescioli G, Lombardi N, Chiesi F, Shtylla J, Del Riccio M, Bonanni P, Boccalini S. Knowledge and training of Italian students in Healthcare Settings on COVID-19 vaccines and vaccination strategies, one year after the immunization campaign. Journal of preventive medicine and hygiene 2023;64(2):E152-E160. doi:10.15167/2421-4248/jpmh2023.64.2.2934
40. Blake H, Fecowycz A, Starbuck H, Jones W. COVID-19 Vaccine Education (CoVE) for Health and Care Workers to Facilitate Global Promotion of the COVID-19 Vaccines. International journal of environmental research and public health 2022;19(2). doi:10.3390/ijerph19020653
41. Boccalini S, Vannacci A, Crescioli G, Lombardi N, Del Riccio M, Albora G, Shtylla J, Masoni M, Guelfi MR, Bonanni P, Bechini A. Knowledge of University Students in Health Care Settings on Vaccines and Vaccinations Strategies: Impact Evaluation of a Specific Educational Training Course during the COVID-19 Pandemic Period in Italy. Vaccines 2022;10(7). doi:10.3390/vaccines10071085
42. Fadel EA, Alshawish E, El-Shaboury RHR, Khalil DE, Mahmoud FZ, El-Feshawy NI. Effect of Implementing Virtual Educational Sessions on Nursing Students' Knowledge, Attitude and Hesitancy Regarding COVID-19 Vaccination. Inquiry: a journal of medical care organization, provision and financing 2025;62:469580251339114. doi:10.1177/00469580251339114
43. Findyartini A, Greviana N, Hanum C, Husin JM, Sudarsono NC, Krisnamurti DGB, Rahadiani P. Supporting newly graduated medical doctors in managing COVID-19: An evaluation of a Massive Open Online Course in a limited-resource setting. PLOS ONE 2021;16(9). doi:10.1371/journal.pone.0257039
44. Girard H., Bosshard W., Krief H., Bula C.J. AO - Bula, Christophe J. Effectiveness of Information Sessions About COVID-19 Vaccines in Healthcare Professionals Working in Geriatrics. Gerontology and Geriatric Medicine 2022;8. doi:10.1177/23337214221115235
45. Kaufman J, Overmars I, Fong J, Tudravu J, Devi R, Volavola L, Vodonaivalu L, Jenkins K, Leask J, Seale H, Mohamed Y, Joshi K, Datt H, Sagan S, Dynes M, Hoq M, Danchin M. Training health workers and community influencers to be Vaccine Champions: a mixed-methods RE-AIM evaluation. BMJ Global Health 2024;9(9). PMID:39251236
46. Clay J, Morton K, Franz D, Jaqua E, Nguyen V. Quality Improvement for Outpatient COVID-19 Infection Control. Cureus 2021;13(7):e16373. doi:10.7759/cureus.16373
47. Greaves SW, Alter SM, Ahmed RA, Hughes KE, Doos D, Clayton LM, Solano JJ, Echeverri S, Shih RD, Hughes PG. A Simulation-based PPE orientation training curriculum for novice physicians. Infection Prevention in Practice 2023;5(1). doi:10.1016/j.infpip.2022.100265
48. Wang SH, Yimer G, Bisesi M, Lisawork L, Sugerman D, Alayu M, Wossen M, Abayneh SA, Gallagher K, Endashaw T, Kubinson H, Kanter T, Gallagher K, Gebreyes W. Rapid virtual training and field deployment for COVID-19 surveillance officers: experiences from Ethiopia. Pan African medical journal 2022;43:23. doi:10.11604/pamj.2022.43.23.28787
49. Hwang WJ, Lee J. Effectiveness of the Infectious Disease (COVID-19) Simulation Module Program on Nursing Students: Disaster Nursing Scenarios. Journal of Korean Academy of Nursing 2021;51(6):648-660. doi:10.4040/jkan.21164
50. Shahrin L, Parvin I, Sarmin M, Abbassi NA, Ackhter MM, Alam T, Mamun GMS, Rahman A, Shaima SN, Shikha SS, Jeorge DH, Nahar MA, Sharifuzzaman, Saha H, Rahman ASMMH, Shahid ASMSB, Faruque ASG, Ahmed T, Chisti MJ. In-person training on COVID-19 case management and infection prevention and control: Evaluation of healthcare professionals in Bangladesh. PLOS ONE 2022;17(10):e0273809. doi:10.1371/journal.pone.0273809
51. Zafar N, Jamal Z, Mujeeb Khan M. Preparedness of the Healthcare Personnel Against the Coronavirus Disease 2019 (COVID-19) Outbreak: An Audit Cycle. Frontiers in public health 2020;8:502. doi:10.3389/fpubh.2020.00502

52. Thakre SS, Jadhao AR, Dhoble MA, Dass R, Thakre SB, Somani A. Evaluation of Effectiveness of Covid-19 Training and Assessment of Anxiety among Nurses of a Tertiary Health Care Center during the Corona Virus Pandemic-An Experimental Study. Journal Of Clinical and Diagnostic Research 2020;14(11):LC34-LC37. doi:10.7860/JCDR/2020/45464.14301
53. Thakre SS, Thakre SB, Jadhao A, Dass R, Dhoble MA, Tiwari PN. Evaluation of effectiveness of COVID-19 training of tertiary health care workers. Int J Community Med Public Health 2020;7(7):2635. doi:10.18203/2394-6040.ijcmph20202989
54. Qasmi SA, Standley C, Mohsin S, Sarwar S, Malik L, Aziz F. Effectiveness of international virtual training on biorisk management in the context of COVID-19. Frontiers in public health 2022;10. doi:10.3389/fpubh.2022.888097
55. Aujee D.S., Aghamkar J., Yangad S., Salvi R. "Assessment of Educational Intervention On Knowledge Regarding World Health Organization Covid-19 Protocols During Delivery AmongStaff Nurses Working In Labour Room of Selected Hospitals. Journal of Pharmaceutical Negative results 2022;13:4717-4721. doi:10.47750/pnr.2022.13.S08.613
56. Ahmed NH, Tosson MM, Badia TS. Effect of educational program on maternity nurses' knowledge, attitude and practice of preventive measures towards COVID-19. Assiut Scientific Nursing Journal 2022;0(0):0. doi:10.21608/asnj.2022.116049.1301
57. Halemani K, Cheema M, Khatun S, Yadidya, Singh B, Gupta V K, Sharma A. An effectiveness of training program on COVID-19 among healthcare students: A cross section study. International Journal of Research in Pharmaceutical Sciences 2020;11(13):1250-1254. doi:10.26452/ijrps.v11iSPL1.3613
58. Han B, Zang F, Liu J, Li S, Zhang W, Zhang Y, Li Z. Effect Analysis of "Four-Step" Training and Assessment Tool in the Prevention and Control of COVID-19. Infection and drug resistance 2022;15:1247-1257. doi:10.2147/IDR.S346559
59. Li Z, Cheng J, Zhou T, Wang S, Huang S, Wang H. Evaluating a Nurse Training Program in the Emergency Surgery Department Based on the Kirkpatrick's Model and Clinical Demand During the COVID-19 Pandemic. Telemedicine journal and e-health : the official journal of the American Telemedicine Association 2020;26(8):985-991. doi:10.1089/tmj.2020.0089
60. Kobayashi D, Mami K, Fujishiro S, Nukanobu N, Ueno SI, Kuwakado S, Koyama T, Kuga H. Online training of Covid-19 infection prevention and control for healthcare workers in psychiatric institutes. BMC Psychiatry 2023;23(1). doi:10.1186/s12888-023-04826-5
61. Macht L, Worlitzsch D, Braijoshri N, Bequiri P, Zudock J, Zilezinski M, Stoevesandt D, Smith J, Hofstetter S. COVID-19: Development and implementation of a video-conference-based educational concept to improve the hygiene skills of health and nursing professionals in the Republic of Kosovo. GMS Hygiene and Infection Control 2022;17. doi:10.3205/dgkh000412
62. Saati AA, Alkalash SH. Promotion of knowledge, attitude, and practice among medical undergraduates regarding infection control measures during COVID-19 pandemic. Frontiers in public health 2022;10. doi:10.3389/fpubh.2022.932465
63. Sangwan J, Lathwal S, Lohan K, Yadav K, Adlakha N, Mane P, Gole S. Impact of training on Knowledge, Attitude and Perceived Barriers for Compliance Regarding use of Protective Equipment Kit among Frontline Healthcare Workers during COVID-19 Pandemic. J Clin Diagn Res 2022;16(1):JC17-JC21. doi:10.7860/JCDR/2022/50845.15913
64. Sharma R, Mohanty A, Singh V, S VA, Gupta PK, Jelly P, Gupta P, Rao S. Effectiveness of Video-Based Online Training for Health Care Workers to Prevent COVID-19 Infection: An Experience at a Tertiary Care Level Institute, Uttarakhand, India. Cureus 2021;13(5):e14785. doi:10.7759/cureus.14785
65. Singh V, Supehia S, Gupta PK, Narula H, Sharma M, Devi K, Bhute AR. Effectiveness of video modules in infection control trainings during COVID-19 pandemic: A quasi-experimental study in tertiary care institute. Journal of education and health promotion 2021;10(1):183. doi:10.4103/jehp.jehp_1009_20
66. Espinoza-Castro B, Encina V, Garrido MA, Vinueza FI, Piedra JP, Garzon-Villalba X, Radon K. Online learning for crisis response: evaluating reach and perceived knowledge gains from the MOOC "Infection, Prevention, and Control of Acute Respiratory Infections for Healthcare Workers in Low- and Middle-Income Countries (IPC MOOC)". BMC Medical Education 2025;25(1):1150. doi:10.1186/s12909-025-07661-2
67. Brito-Brito PR, Fernandez-Gutierrez DA, Martinez-Alberto CE, Saez-Rodriguez MJ, Nunez-Marrero J, Garcia-Hernandez AM. Use of the Nursing Outcomes Classification (NOC) to measure perceived knowledge about the control of SARS-CoV-2 infection: The impact of a training program in primary healthcare professionals. International journal of nursing knowledge 2021. doi:10.1111/2047-3095.12356
68. Mark ME, LoSavio P, Husain I, Papagiannopoulos P, Batra PS, Tajudeen BA. Effect of Implementing Simulation Education on Health Care Worker Comfort With Nasopharyngeal Swabbing for COVID-19. Otolaryngology-head and neck surgery: Official journal of American Academy of Otolaryngology-Head and Neck Surgery 2020;163(2):271-274. doi:10.1177/0194599820933168
69. Bakhsh A, Asiri R, Alotaibi H, Alsaeedi R, Shahbar R, Boker A. Rapid cycle training for non-critical care physicians to meet intensive care unit staff shortage at an academic training center in a developing country during the COVID-19 pandemic. BMC Medical Education 2023;23(1):493. doi:10.1186/s12909-023-04478-9
70. Lalitha ND, Bhadauria US, Agarwal D, Purohit BM, Priya H, Nilima N, Duggal R, Mathur VP, Logani A. Comparing the effectiveness of two educational methods for oral health management in COVID-19 pandemic among dental professionals. Przegl Epidemiol 2024;78(1):90-93. PMID:38904315
71. Mektrirat R, Sathanawongs A, Tiwananthagorn S, Chaisowwong W, Peansukmanee S, Naksen W, Thongprachum A. Achieving Interprofessional Education on Collaborative Problem-Solving for COVID-19 Using Project-Based Approach. International Journal of Infectious Diseases 2021;116:S67-. doi:10.1016/j.ijid.2021.12.158
72. Roberts KJ, Zumstein KK, Lamphere TR, Williams M, Powell SA, Moran A, Kellar B, Solly WR, Pierce M. Improving Students' Knowledge and Skills Through a Tele-ICU Clinical Rotation. Respiratory Care 2022;67(7):789-794. doi:10.4187/respcare.09896
73. Tan W, Ye Y, Yang Y, Chen Z, Yang X, Zhu C, Chen D, Tan J, Zhen C. Whole-Process Emergency Training of Personal Protective Equipment Helps Healthcare Workers Against COVID-19: Design and Effect. J Occup Environ Med 2020;62(6):420-423. doi:10.1097/JOM.0000000000001877
74. Díaz-Guio DA, Ricardo-Zapata A, Ospina-Velez J, Gómez-Candamil G, Mora-Martinez S, Rodriguez-Morales AJ. Cognitive load and performance of health care professionals in donning and doffing PPE before and after a simulation-based educational intervention and its implications during the COVID-19 pandemic for biosafety. Le Infezioni in Medicina 2020:111-117.
75. Pokrajac N, Schertzer K, Poffenberger CM, Alvarez A, Marin-Nevarez P, Winstead-Derlega C, Gisondi MA. Mastery Learning Ensures Correct Personal Protective Equipment Use in Simulated Clinical Encounters of COVID-19. West J Emerg Med 2020;21(5):1089-1094. doi:10.5811/westjem.2020.6.48132
76. Smith CR, Vasilopoulos T, am Frantz, LeMaster T, Martinez RA, am Gunnett, Fahy BG. Staying proper with your personal protective equipment: How to don and doff. Journal Of Clinical Anesthesia 2023;86. doi:10.1016/j.jclinane.2023.111057

77. Ta'an WF, Al-Hammouri MM, Al-Faouri I, Suliman MM. The effectiveness of COPA-based training program on the infection- control competencies of newly hired healthcare professionals. Teaching and Learning in Nursing 2023;18(1):160-165. doi:10.1016/j.teln.2022.06.009
78. Drevon D, Fursa SR, Malcolm AL. Intercoder Reliability and Validity of WebPlotDigitizer in Extracting Graphed Data. Behav Modif 2017;41(2):323-339. doi:10.1177/0145445516673998
79. Altillo BSA, Gray M, Avashia SB, Norwood A, Nelson EA, Johnston C, Bhavnani D, Patel H, Allen CH, Adeni S, Phelps ND, Mercer T. Global health on the front lines: an innovative medical student elective combining education and service during the COVID-19 pandemic. BMC Medical Education 2021;21(1):186. doi:10.1186/s12909-021-02616-9
80. Kufel WD, Blaine BE, Avery LM. Pharmacy students' knowledge and confidence of COVID-19 following an interactive didactic class. Journal Of The American College Of Clinical Pharmacy 2022;5(10):1082-1087. doi:10.1002/jac5.1678
81. Naz F, Ohri P, Sharma A, Spandana BS, Gupta K. Impact of Training on Awareness of COVID-19 among The Health Care Workers in A Tertiary Care Hospital of Dehradun. Indian Journal of Community Health 2022;34(1):20-25. doi:10.47203/IJCH.2022.v34i01.005
82. Roberts EN, Smithing RT, Tucker P. Measuring the impact of a COVID-19 continuing education program. Journal of the American Association of Nurse Practitioners 2022;34(6):835-843. doi:10.1097/JXX.0000000000000715
83. Tsiouris F, Hartsough K, Poimbouef M, Raether C, Farahani M, Ferreira T, Kamanzi C, Maria J, Nshimirimana M, Mwanza J, Njenga A, Odera D, Tenthani L, Ukaejiofo O, Vambe D, Fazito E, Patel L, Lee C, Michaels-Strasser S, Rabkin M. Rapid scale-up of COVID-19 training for frontline health workers in 11 African countries. Human resources for health 2022;20(1):43. doi:10.1186/s12960-022-00739-8
84. Salehi R, Young S de, Asamoah A, Aryee SE, Eli R, Couper B, Smith B, Djokoto C, Agyeman YN, Zakaria AF, Butt N, Boadu A, Nyante F, Merdiemah G, Oliver-Commey J, Ofori-Boadu L, Akoriyea SK, Parry M, Fiore C, Okae F, Adams A, Acquah H. Evaluation of a continuing professional development strategy on COVID-19 for 10 000 health workers in Ghana: a two-pronged approach. Human resources for health 2023;21(1). doi:10.1186/s12960-023-00804-w
85. Rosas-Magallanes C, Basto-Abreu A, Barrientos-Gutiérrez T, Ramírez-Martínez JL, Tamayo-Ortiz M, Gutiérrez-Díaz HO, Magaña-Valladares L, Cordera DB, Santamaría-Guasch CM, Hernández-Avila M. CLIMSS online platform as a health literacy tool during the health crisis of Covid-19. Salud Publica De Mexico 2022;64(3):320-327. doi:10.21149/13103
86. Bohara A, Thapa S, Yilmaz SK, McBee SH. An Impact Evaluation of COVID-19 Training Program: Knowledge and Awareness of Public Health Professionals of Province Five, Nepal; 2021.
87. Garcia KS, Rodriguez A, Gonzalez Z, Armstrong C, Iacob E, Flynn EE, Simmons M. Pretest-post-test evaluation with lay midwives in remote Guatemala after educational activities about COVID-19. Rural and Remote Health 2024;24(3):8387. doi:10.22605/RRH8387
88. Sabandüzen H, Kavaklı Ö. Evaluation of the effectiveness of the training on "Home care of COVID-19 positive/suspicious patients" given to nursing students: A quasi-experimental study. Journal of education and health promotion 2024;13:250. doi:10.4103/jehp.jehp_1574_23
89. Said AR. Knowledge and Practices of Nurses Regarding Corona Virus (COVID-19): An Educational Intervention. MLU 2021;21(2):36-47. doi:10.37506/mlu.v21i2.2642
90. Strehlow MC, Johnston JS, Aluri KZ, Prober CG, Acker PC, Patil AS, Mahadevan A, Mahadevan SV. Evaluation of a massive open online course for just-in-time training of healthcare workers. Frontiers in public health 2024;12:1395931. doi:10.3389/fpubh.2024.1395931
91. Bieri J, Tuor C, Nendaz M, L Savoldelli G, Blondon K, Schiffer E, Zamberg I. Implementation of a Student-Teacher-Based Blended Curriculum for the Training of Medical Students for Nasopharyngeal Swab and Intramuscular Injection: Mixed Methods Pre-Post and Satisfaction Surveys. JMIR Medical Education 2023;9:e38870. doi:10.2196/38870
92. Instrum RS, Koch RW, Rocha T, Rohani SA, Ladak H, Agrawal SK, Sowerby LJ. Improving Nasopharyngeal Swab Technique via Simulation for Frontline Workers. The Laryngoscope 2022. doi:10.1002/lary.30034
93. Abbas K, Nawaz SMA, Amin N, Soomro FM, Abid K, Ahmed M, Sayeed KA, Ghazanfar S, Qureshi N. A web-based health education module and its impact on the preventive practices of health-care workers during the COVID-19 pandemic. Health Education Research 2020;35(5):353-361. doi:10.1093/her/cyaa034
94. Zhao J, Rozenberg D, Kaul R, Sanh M, Luther R, Orchanian-Cheff A, Nourouzpour S, de Peiza P, Agbeyaka S, Gebara N, Doumouras AM, Draper H, Barber M, Lau J, Furlan A. The positive impact of a telemedicine education program on healthcare workers during the COVID-19 pandemic in Ontario, Canada. The Annals of Family Medicine 2022(20). doi:10.1370/afm.20.s1.3260
