# Supplementary material for: Interventions to Enhance COVID-19 Pandemic Health Literacy in Health Professionals: Systematic Review
Source: JMIR Med Educ. 2026 Jul 10;12:e70400. doi: 10.2196/70400 (PMC13360183; doi:10.2196/70400)
Supplement: Multimedia Appendix 5 — Narrative analyses of primary outcomes in uncontrolled before-and-after studies. [file mededu-v12-e70400-s005.pdf]

Narrative Analyses of Primary Outcome in Uncontrolled Before-After Studies

Interventions in enhancing COVID-19-related knowledge

Among the 20 (27%) uncontrolled before after-studies (3,762 participants), addressing general COVID-19-related knowledge, 17 studies (18 %, 3,179 participants) reported effects that favour the intervention, whereof 14 studies (19 %) showed significant changes. One study [1] (63 participants) found no significant effects between pre- and post-testing. Beneficial outcome changes in (perceived) COVID-19-specific vaccine knowledge were found in six out of seven studies. One study [2] reported no changes after the intervention referring to vaccine knowledge (2,230 participants). With regard to the specific knowledge in virus transmission three of five studies showed favourable effects for the intervention, when comparing pre- and posttests (204 participants). In 16 studies (14,759 participants) out of 17 studies aimed at enhancing knowledge on infection prevention control measure, positive effects of the conducted interventions were reported. One study [3] found no significant changes (12 participants).

Interventions in enhancing COVID-19-related infection prevention control skills

A total of eleven uncontrolled before-after studies (15 %) measured COVID-19-related infection prevention performance skills. In five studies (7 %, 458 participants), interventions aimed at enhancing general infection prevention skills were reported to have a beneficial effect, when comparing pre- and post-tests results. Further four studies (5 %, 261 participants) investigated PPE performance of donning and donning and detected favourable changes after the intervention.

References

1. Odusanya OO, Adeniran A, Bakare OQ, Odugbemi BA, Enikuomehin OA, Jeje OO, Emechebe AC. Building capacity of primary health care workers and clients on COVID-19: Results from a web-based training. PLOS ONE 2022;17(10):e0274750. doi:10.1371/journal.pone.0274750
2. Findyartini A, Greviana N, Hanum C, Husin JM, Sudarsono NC, Krisnamurti DGB, Rahadiani P. Supporting newly graduated medical doctors in managing COVID-19: An evaluation of a Massive Open Online Course in a limited-resource setting. PLOS ONE 2021;16(9). doi:10.1371/journal.pone.0257039
3. Macht L, Worlitzsch D, Braijoshri N, Bequiri P, Zudock J, Zilezinski M, Stoevesandt D, Smith J, Hofstetter S. COVID-19: Development and implementation of a video-conference-based educational concept to improve the hygiene skills of health and nursing professionals in the Republic of Kosovo. GMS Hygiene and Infection Control 2022;17. doi:10.3205/dgkh000412
